# Supplementary material for: Genetic Incorporation of Diverse Noncanonical Amino Acids for Histidine Substitution
Source: J Am Chem Soc. 2026 Mar 26;148(13):13619–32. doi: 10.1021/jacs.5c19599 (PMC13067346; doi:10.1021/jacs.5c19599)
Supplement: Supplementary file 1 [file ja5c19599_si_001.pdf]

Supporting information: Genetic incorporation of diverse non-canonical amino acids for histidine substitution

Anton Natter Perdiguero[1†], Sandro Fischer[1†], Alrika R. Lischke[1], Benjamin P. Manser[1], Alexandria Deliz Liang[1]*

[1]Department of Chemistry, University of Zurich; Winterthurerstrasse 190, 8057, Zurich, Switzerland.

[†]These authors contributed equally.

*Correspondence should be addressed to A.D.L. (alexandriadeliz.liang@uzh.ch).

Table of Contents

I.	Supplementary data figures	2
II.	Synthesis	19
III.	Supplementary tables	62
IV.	DNA and Protein Sequences	69
V.	Plasmid Construction	76
VI.	References	91

I. Supplementary data figures

All figures were prepared as vector graphics or high-resolution (≥300 dpi) raster images and are intended to be viewed at 100% zoom *or higher* to ensure accurate rendering across viewing environments.

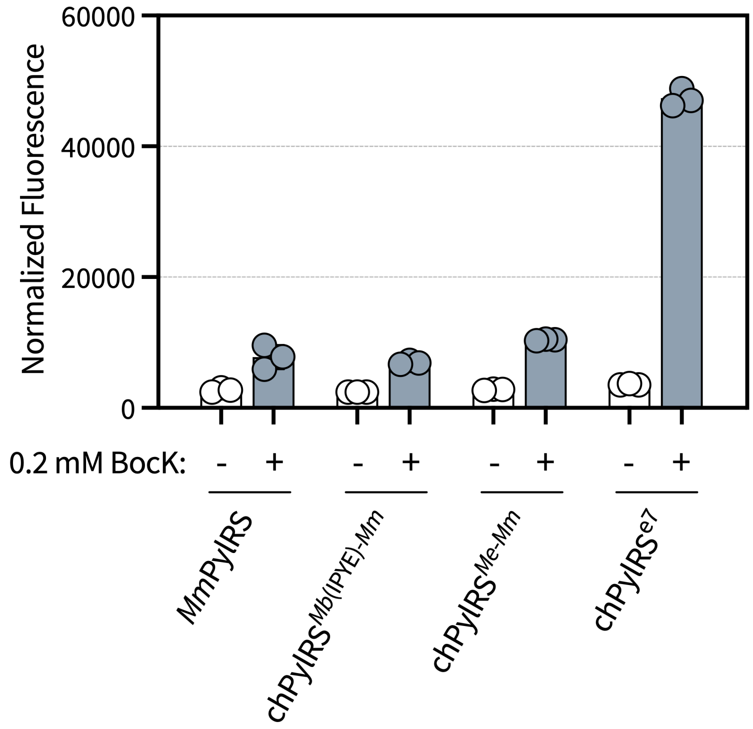

Supplementary Figure 1 | Validation of chPylRS^{E7} variants for incorporation of BocK. Suppression of sfGFP150$_{TAG}$ in NEB10β in the presence or absence of different PylRS variants with 0.2 mM BocK. The data are depicted as normalized fluorescence (excitation at 480 nm and emission at 510 nm, normalized to the optical density at 600 nm) and represent the mean and standard deviation of three biological replicates.

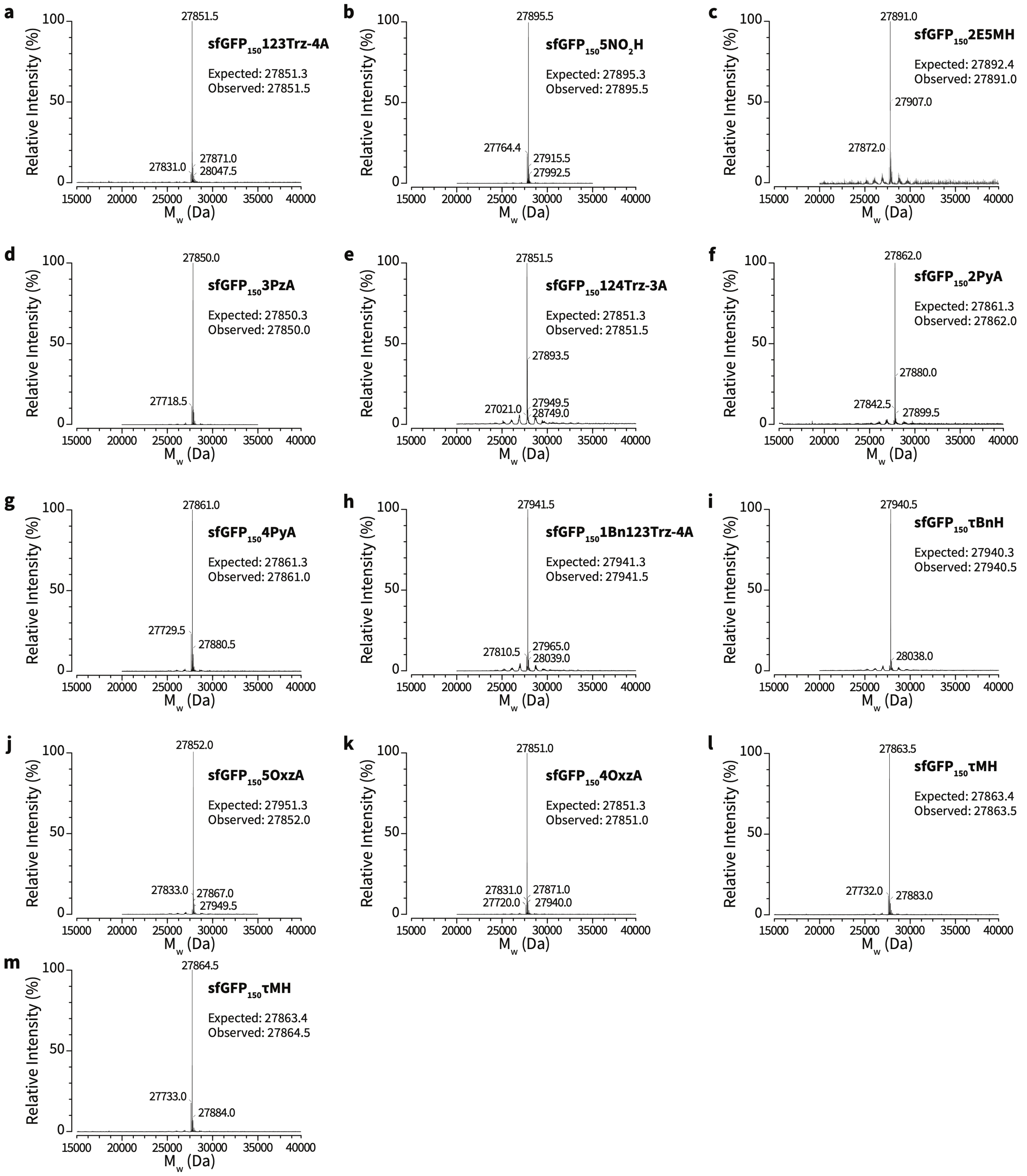

Supplementary Figure 2 | LC-MS spectra of sfGFP150$_{TAG}$ containing a single histidine-like ncAA. Intact LC-MS analysis (positive electrospray time of flight) of sfGFP150$_{TAG}$ production in the presence of different ncAAs. The expected and observed masses confirming incorporation of the desired ncAA are indicated. Additional peaks, consistent with typically observed [H_2O] elimination (-18 Da), $[Na]^+$ addition (+23 Da) or loss of N-terminal [Met] (-131 Da) were observed. Panels l and m were derived with the use of *Mb*(IPYE)PylRS^{p10} and *Mb*(IPYE)PylRS$^{\tau MH}$, respectively.

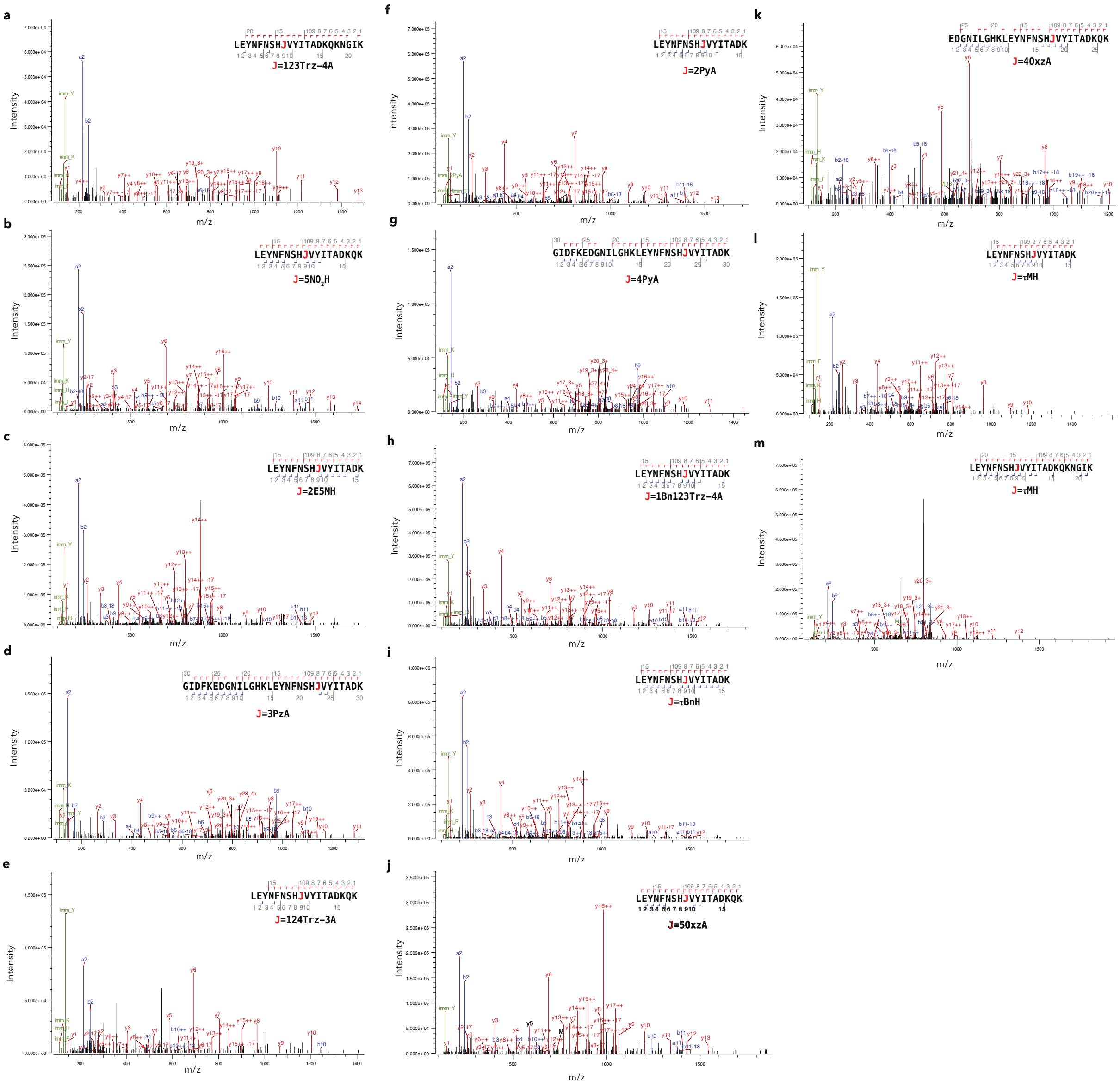

Supplementary Figure 3 | LC-MS/MS spectra of sfGFP150$_{TAG}$ containing a single histidine-like ncAA. A representative LC-MS/MS spectrum from a tryptic digest of sfGFP150$_{TAG}$ expressed with different ncAAs and their corresponding aaRS/tRNA pairs. Typically, multiple peptides containing the desired ncAA were observed and no peptides for canonical amino acid incorporation were observed. For samples of sfGFP-5NO_2H, sfGFP-τBnH, sfGFP-1Bn123Trz-4A and sfGFP-124Trz-3A, some peptides containing canonical amino acids were observed (see **Supplementary Table 9 and 10**). Panels l and m were derived with the use of *Mb*(IPYE)PylRS^{p10} and *Mb*(IPYE)PylRS^{τMH}, respectively.

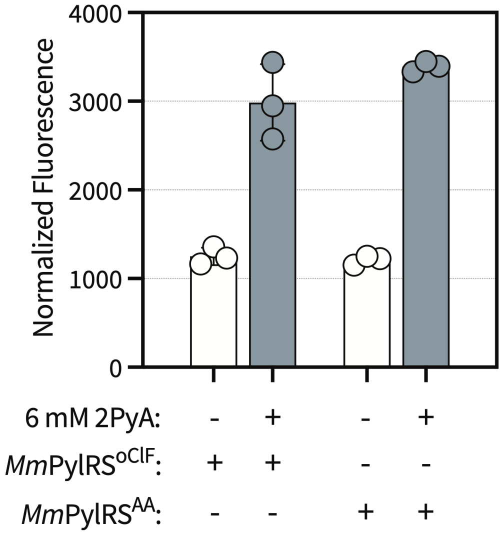

Supplementary Figure 4 | Testing of reported variants for incorporation of 2PyA. Suppression of sfGFP150$_{TAG}$ in NEB10β in the presence or absence of 6 mM 2PyA with different PylRS variants (*Mm*PylRS^{oClF} [1] and *Mm*PylRS^{AA} [2]). The data are shown as normalized fluorescence (excitation at 480 nm and emission at 510 nm, normalized to the optical density at 600 nm) and represent the mean and standard deviation of three biological replicates.

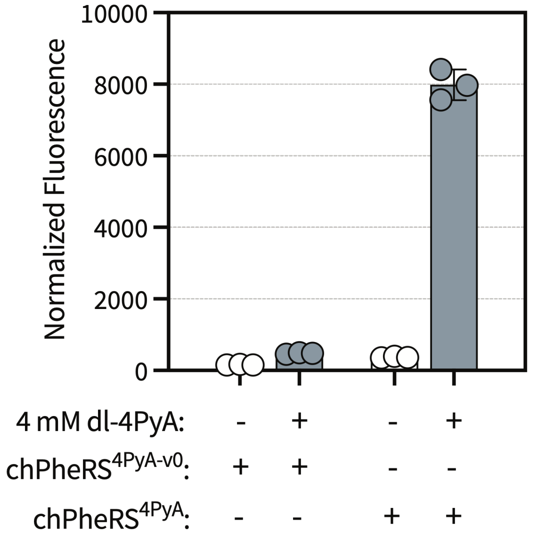

Supplementary Figure 5 | Validation of chPheRS^{4PyA} variants for incorporation of 4PyA. Suppression of sfGFP150$_{TAG}$ in NEB10β in the presence or absence of 4 mM 4PyA with different **chPheRS** variants. The data are shown as normalized fluorescence (excitation at 480 nm and emission at 510 nm, normalized to the optical density at 600 nm) and represent the mean and standard deviation of three biological replicates.

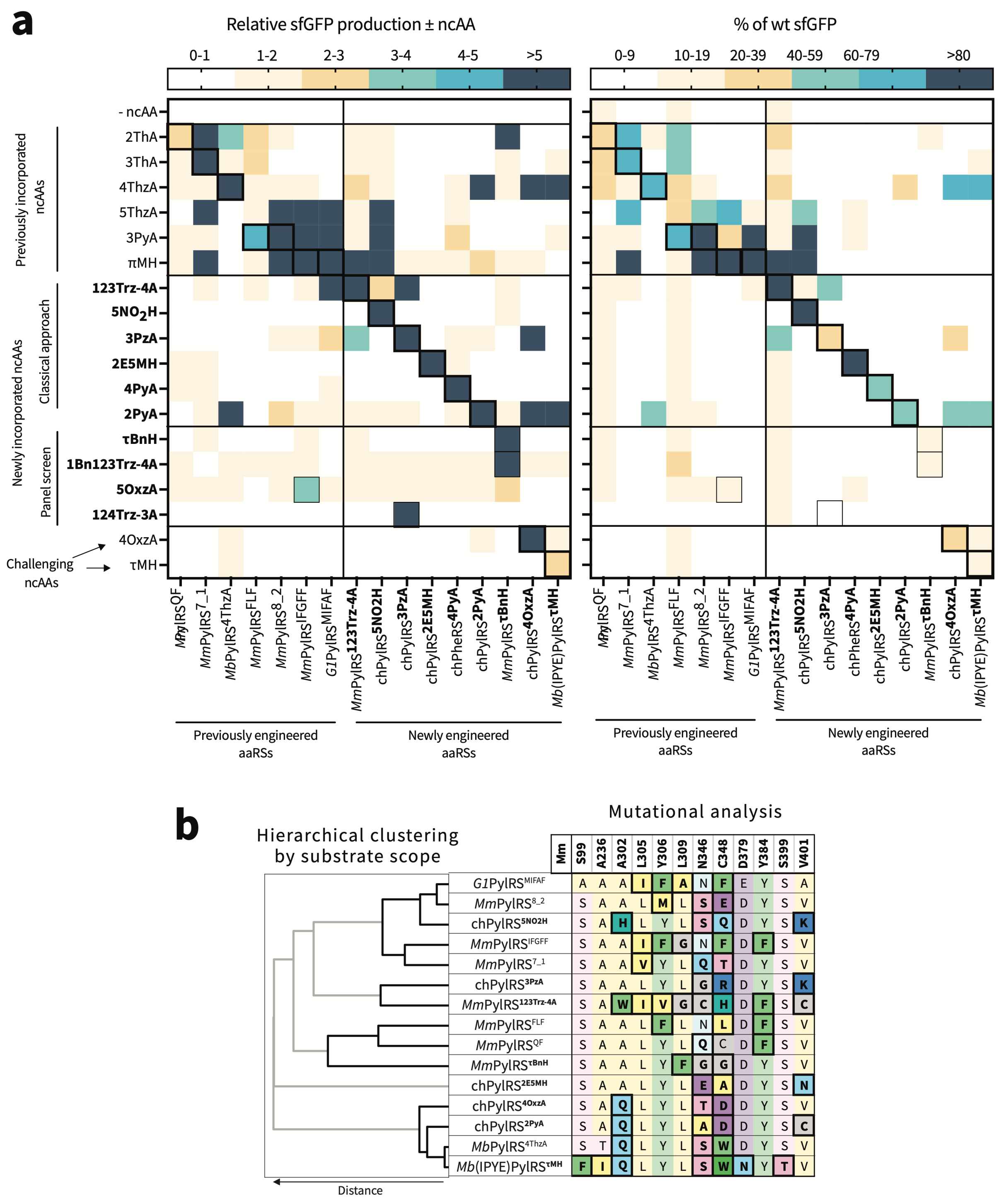

Supplementary Figure 6 | aaRS-ncAA substrate specificity profiling. a) Substrate profiling analysis with relative sfGFP production ± ncAA (reproduction from Figure 2a for clear comparison) and % of wt sfGFP. Relative sfGFP production (± ncAA) is calculated from the normalized fluorescence (excitation at 480 nm and emission at 510 nm, normalized to the optical density at 600 nm) in the presence of the ncAA divided by the normalized fluorescence in the absence of the ncAA. The data represent the mean of 2-3 biological replicates. The data for % of wt sfGFP was derived from normalized fluorescence (excitation at 480 nm and emission at 510 nm, normalized to the optical density at 600 nm) as percentage of a wt sfGFP reference. The data represent the mean of 2-3 biological replicates. b) The hierarchical clustering by substrate scope including chPylRS^{4OxzA} and *Mb*(IPYE)PylRS$^{\tau MH}$ paired with the analysis of mutations found during engineering. The numbering is given based on the *Mm*PylRS sequence numbers.

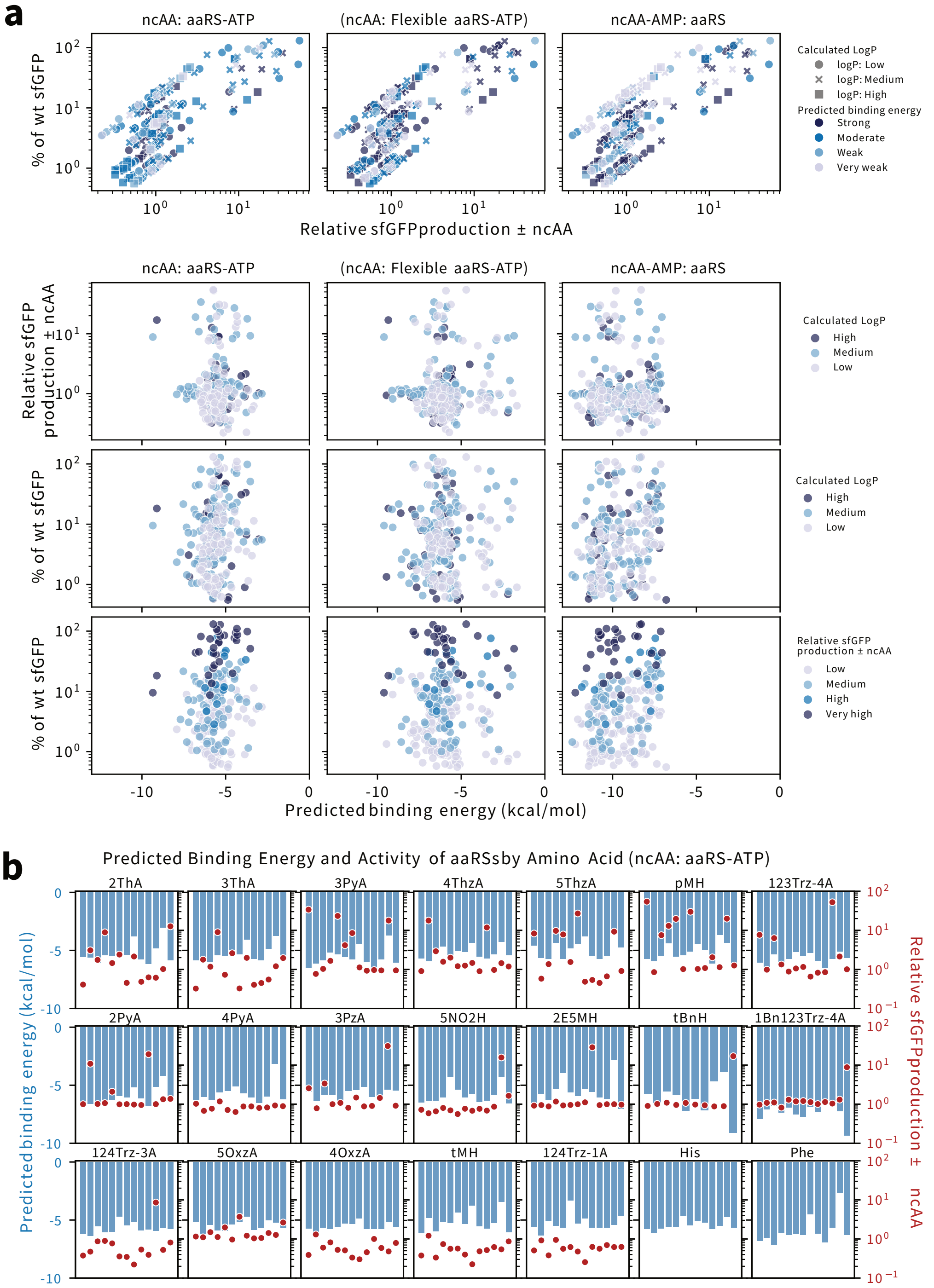

Supplementary Figure 7 | Analysis of predicted affinities and sfGFP production metrics for different PylRS variants. For each ncAA, the binding affinities of the ncAA to the aaRS-ATP adduct and the binding affinities of the ncAA-AMP adduct to the aaRS were predicted. The predictions were obtained from aaRS-ATP structure prediction with Boltz2[3] and docking of the ncAA with Gnina[4]. For binding of the ncAA to the aaRS-ATP adduct both a frozen protein configuration and a flexible protein configuration for residues within 3.5 Å of the substrate binding pocket were considered. The predicted binding affinities were visualized using several different methods: a) bulk analysis of the relationships between relative sfGFP production ± ncAA, % wt sfGFP production, calculated LogP, and the predicted binding energy and b) separate subplots for each ncAA for the ncAA:aaRS-ATP binding analysis, with bars (left y-axis) and scatter circles (right y-axis) indicating the predicted binding energies and the relative GFP production ± ncAA, respectively. No clear correlation between predicted binding energies and sfGFP production metrics could be found, even accounting for LogP.

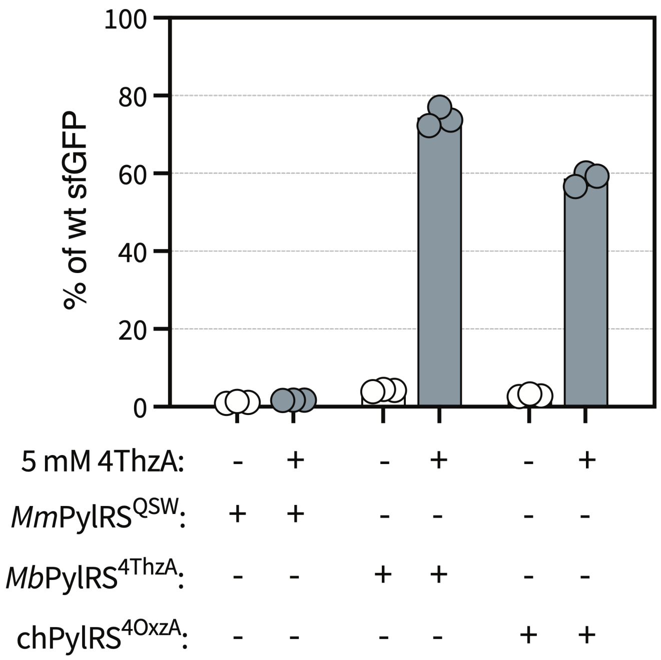

Supplementary Figure 8 | Comparison of different PylRS variants for 4ThzA incorporation. Suppression of sfGFP150$_{TAG}$ in NEB10β in the presence or absence of 5 mM 4ThzA with different PylRS variants. The data are illustrated as normalized fluorescence (excitation at 480 nm and emission at 510 nm, normalized to the optical density at 600 nm) as percentage of a wt sfGFP reference. The mean and standard deviation of three biological replicates are shown. These data together with Supplementary Figure 9 indicate the functional challenges observed upon transplanting the "QSW" mutation into other PylRS systems, as has been observed previously for other sets of mutations in PylRS[5].

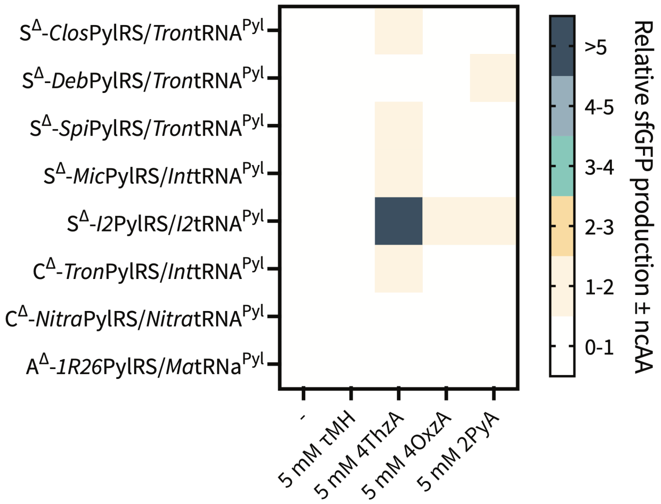

Supplementary Figure 9 | Testing of a broader range of PylRS^{QSW} variants. Suppression of sfGFP150$_{TAG}$ in NEB10β in the presence or absence of different ncAAs with different PylRS variants[6] carrying the "QSW" mutations. The data are depicted as relative sfGFP production ± ncAA, which was calculated from the normalized fluorescence (excitation at 480 nm and emission at 510 nm, normalized to the optical density at 600 nm) in the presence of the ncAA divided by the normalized fluorescence in the absence of the ncAA. The data represent the mean of three biological replicates. These data together with Supplementary Figure 8 indicate the functional challenges observed upon transplanting the "QSW" mutation into other PylRS systems, as has been observed previously for other sets of mutations in PylRS[5].

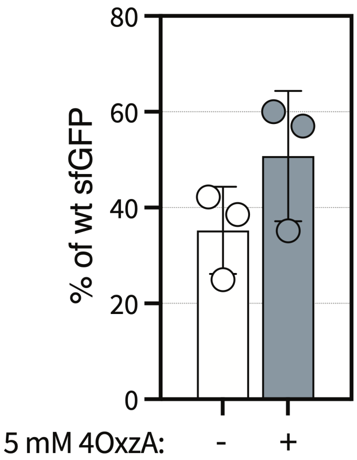

Supplementary Figure 10 | Validation of chPylRS$^{4OxzA\text{-}2}$ for 4OxzA incorporation in sfGFP150$_{TAG}$. Suppression of sfGFP150$_{TAG}$ in NEB10β in the presence or absence of 5 mM 4OxzA. The data show normalized fluorescence (excitation at 480 nm and emission at 510 nm, normalized to the optical density at 600 nm) as percentage of a wt sfGFP reference. The mean and standard deviation of three biological replicates are shown.

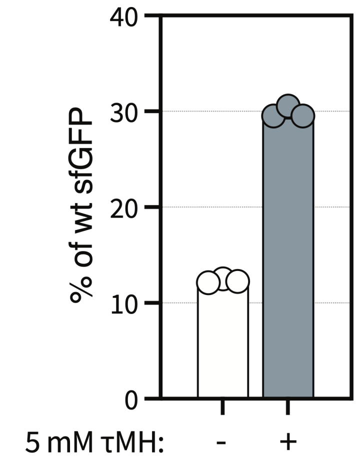

Supplementary Figure 11 | Validation of *Mb*(IPYE)PylRS^{P10} for τMH incorporation in sfGFP150$_{TAG}$. Suppression of sfGFP150$_{TAG}$ in NEB10β in the presence or absence of 20 mM τMH. The data are illustrated as normalized fluorescence (excitation at 480 nm and emission at 510 nm, normalized to the optical density at 600 nm) as percentage of a wt sfGFP reference. The mean and standard deviation of three biological replicates are shown.

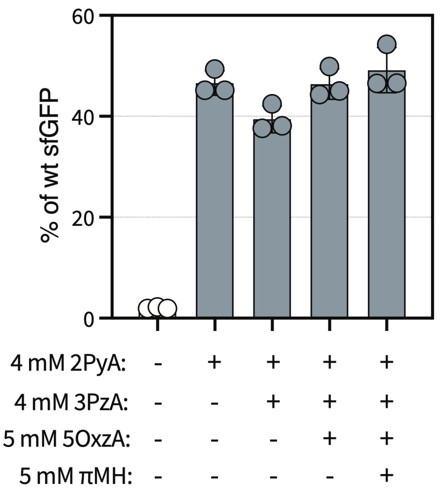

Supplementary Figure 12 | Testing of ncAA incorporation with ncAA mixtures. Suppression of sfGFP150$_{TAG}$ in NEB10β with chPylRS^{2PyA}/*Mm*tRNAPyl$_{CUA}$ in the presence or absence of different ncAAs. The data are illustrated as normalized fluorescence (excitation at 480 nm and emission at 510 nm, normalized to the optical density at 600 nm) as percentage of a wt sfGFP reference. The mean and standard deviation of three biological replicates are shown. The corresponding LC-MS/MS analysis is provided in Supplementary Figure 13.

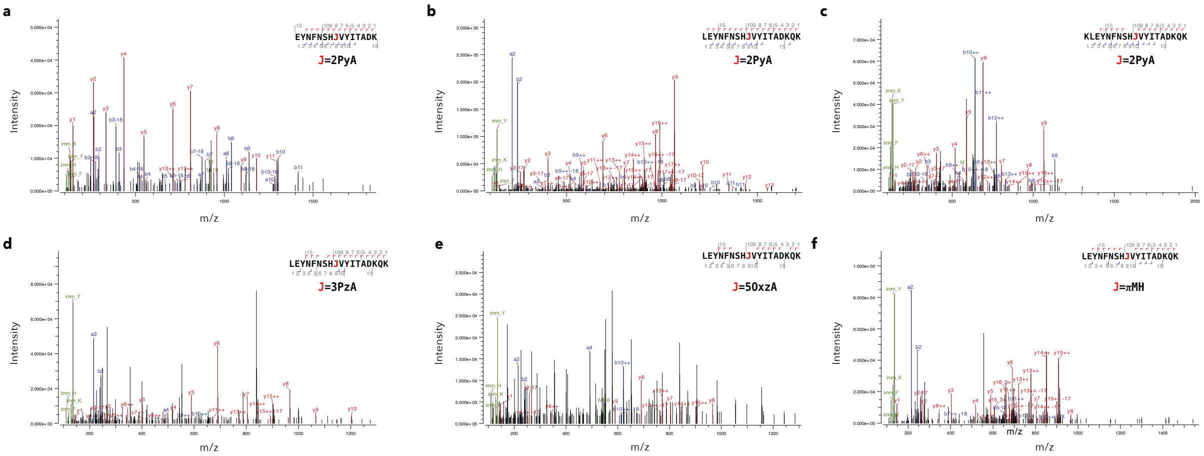

Supplementary Figure 13 | LC-MS/MS spectra of sfGFP150$_{TAG}$ expressed with chPylRS^{2PyA} in the presence of ncAA mixtures. A representative LC-MS/MS spectrum from a tryptic digest of sfGFP150$_{TAG}$ for different expression conditions is shown. Typically, multiple peptides containing 2PyA were observed (a-c). Expressions were carried out with addition of a) 2PyA and 3PzA b) 2PyA, 3PzA and 5OxzA c-f) 2PyA, 3PzA and 5OxzA and πMH. In the condition expressed with 2PyA, 3PzA, 5OxzA and πMH, additionally some peptides with 3PzA (d), 5OxzA (e) or πMH (f) were observed. However, purities were estimated to be above 96%. Thus, we observed that for an example aaRS (chPylRS^{2PyA}) the ncAA incorporation profiles observed in Figure 2a were maintained, when expressing sfGFP150$_{TAG}$ in the presence of mixtures of ncAAs.

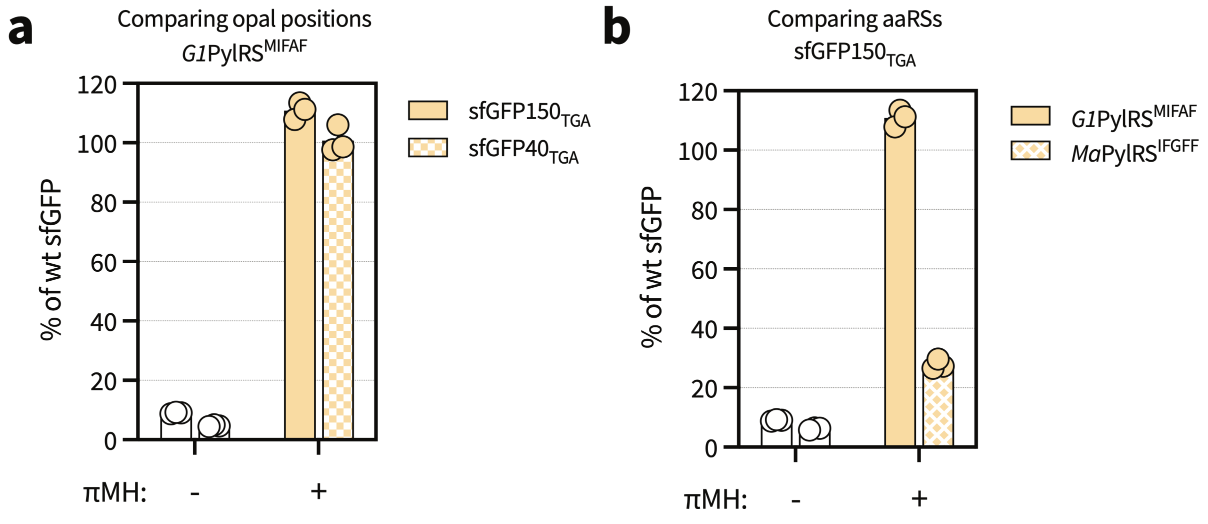

Supplementary Figure 14 | Validation of opal suppression with PylRS pairs. a) Comparison of opal suppression at sfGFP40$_{TGA}$ and sfGFP150$_{TGA}$ in NEB10β in the presence or absence of πMH with *G1*PylRS^{MIFAF}/*Ma*tRNA$^{\Delta NPyl}$(8)$_{UCA}$. b) Comparison of opal suppression with πMH (*G1*PylRS^{MIFAF} and *Ma*PylRS^{IFGFF}) for expression of sfGFP150$_{TGA}$ in NEB10β in the presence or absence of πMH. The data are presented as % of wt sfGFP (as measured by normalized fluorescence—excitation at 480 nm and emission at 510 nm, normalized to the optical density at 600 nm—as percentage of a wt sfGFP reference). The mean and standard deviation of three biological replicates are shown. The data were used to select the *G1*PylRS^{MIFAF}/*Ma*tRNA$^{\Delta NPyl}_{UCA}$ pair for dual suppression. The data also illustrate the impact of codon context dependence vs. the impact of aaRS efficiency.

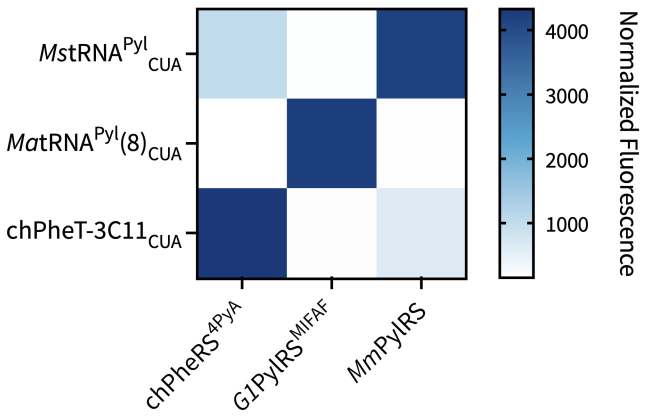

Supplementary Figure 15 | Orthogonality testing of chPheRS^{4PyA}/3C11-chPheT$_{CUA}$ construct. Suppression of sfGFP150$_{TAG}$ in NEB10β for different aaRS-tRNA combinations (chPheRS^{4PyA} with 4 mM dl-4PyA, *G1*PylRS^{MIFAF} with 2 mM πMH, *Mm*PylRS with 4 mM BocK). The data are shown as normalized fluorescence (excitation at 480 nm and emission at 510 nm, normalized to the optical density at 600 nm). The data represent the mean of three biological replicates.

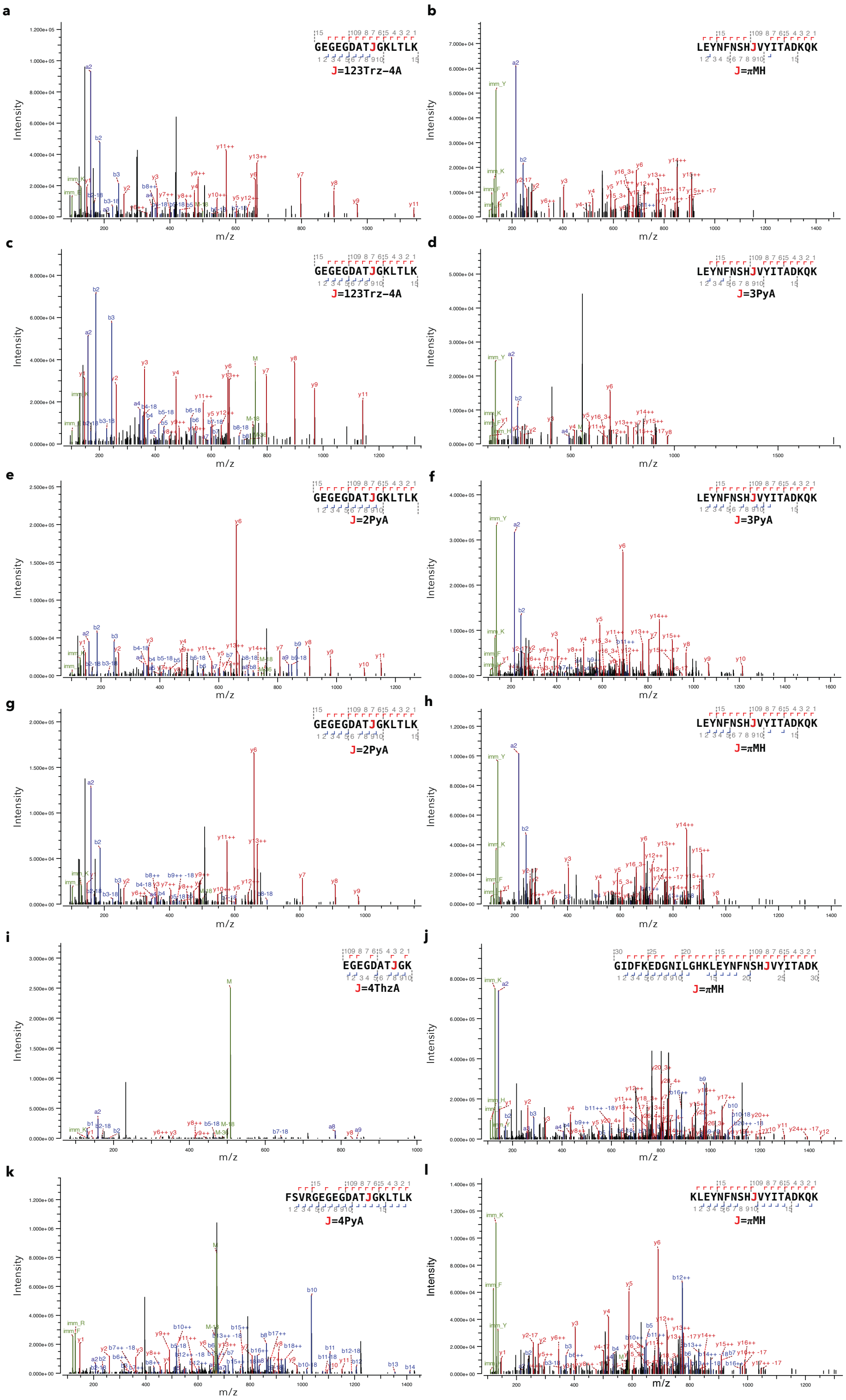

Supplementary Figure 16 | LC-MS/MS spectra of sfGFP40$_{TAG}$150$_{TGA}$ containing two histidine-like ncAA. Representative LC-MS/MS spectra from tryptic digests of sfGFP40$_{TAG}$150$_{TGA}$ expressed with different combinations of aaRS/tRNA pairs. In all cases, peptides containing the desired ncAA were observed and no peptides containing an undesired ncAA at the respective positions were detected. In the samples sfGFP40$_{123Trz\text{-}4A}$150$_{\pi MH}$, sfGFP40$_{2PyA}$150$_{3PyA}$ and sfGFP40$_{2PyA}$150$_{\pi MH}$, some Gln incorporation was detected. In the sfGFP40$_{4ThzA}$150$_{\pi MH}$, low levels of Phe incorporation were detected. a, b) sfGFP40$_{123Trz\text{-}4A}$150$_{\pi MH}$; c, d) sfGFP40$_{123Trz\text{-}4A}$150$_{3PyA}$; e, f) sfGFP40$_{2PyA}$150$_{3PyA}$; g, h) sfGFP40$_{2PyA}$150$_{\pi MH}$; i, j) sfGFP40$_{4ThzA}$150$_{\pi MH}$; k, l) sfGFP40$_{4PyA}$150$_{\pi MH}$.

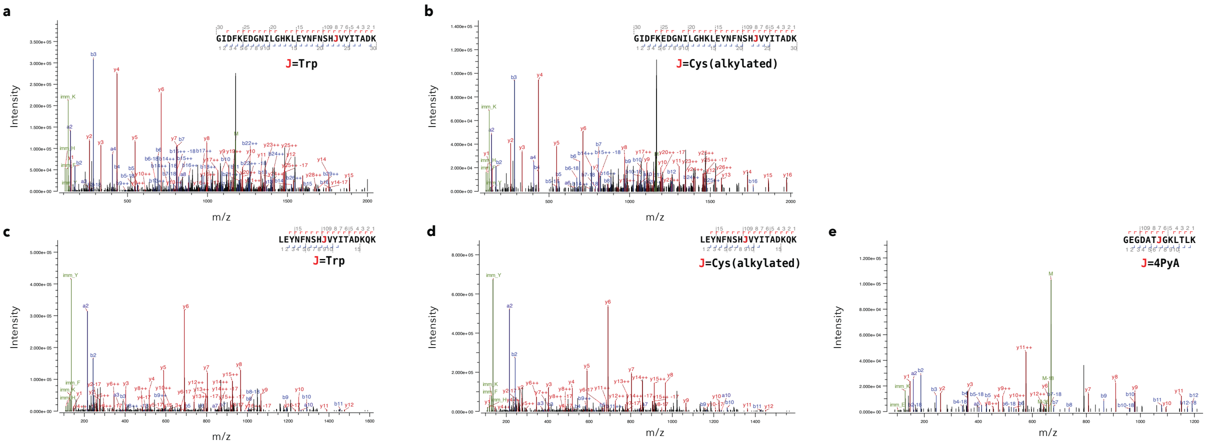

Supplementary Figure 17 | LC-MS/MS analysis of background incorporation at the opal codon. Representative LC-MS/MS spectra from a tryptic digest of sfGFP150$_{TGA}$ expressed with *G1*PylRS^{MIFAF}/*Ma*tRNA$^{\Delta NPyl}$(8)$_{UCA}$ in the absence of ncAA supplementation showing a) Trp incorporation or b) Cys incorporation at position 150. Representative LC-MS/MS spectra from a tryptic digest of sfGFP40$_{TAG}$150$_{TGA}$ expressed with *G1*PylRS^{MIFAF}/*Ma*tRNA$^{\Delta NPyl}$(8)$_{UCA}$ and chPylRS^{4PyA}/3C11-chPheT$_{CUA}$ in the presence of 4 mM 4PyA showing c) Trp incorporation at position 150 or d) Cys incorporation at the position 150 and e) 4PyA incorporation at position 40.

Comparison of codon order/context vs. aaRS-ncAA set

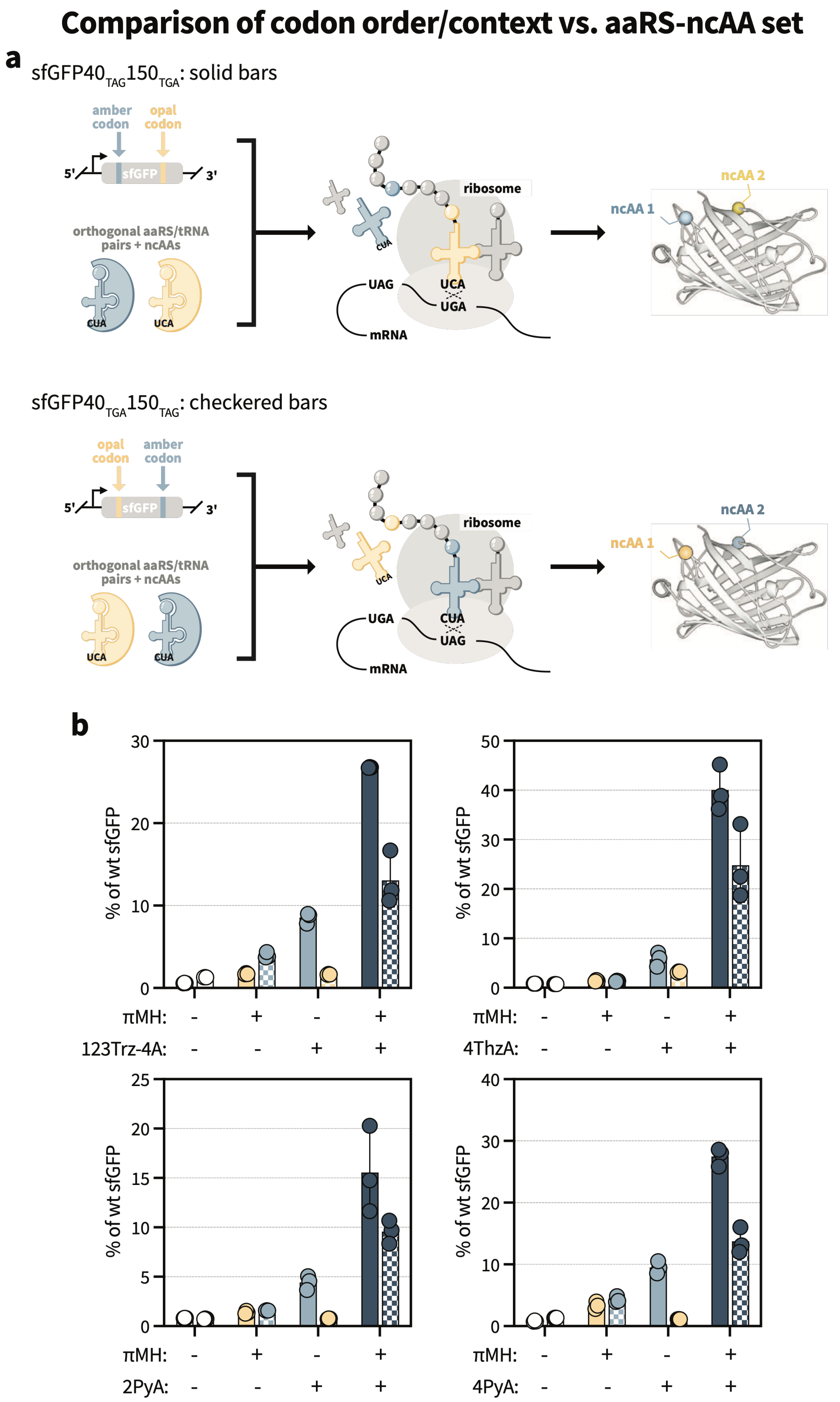

Supplementary Figure 18 | Comparison of dual suppression as a function of codon order/context. a) Schematic representing comparison of incorporation positions: sfGFP40$_{TAG}$150$_{TGA}$ and sfGFP40$_{TGA}$150$_{TAG}$. b) Data for sfGFP production are shown when providing only the amber suppression ncAA (light blue), providing only the opal suppression ncAA (yellow), and providing both ncAAs for dual suppression (dark blue). The sfGFP40$_{TAG}$150$_{TGA}$ and sfGFP40$_{TGA}$150$_{TAG}$ constructs are indicated by the solid bars and the checkered bars, respectively. The data are presented as % of wt sfGFP (as measured by normalized fluorescence—excitation at 480 nm and emission at 510 nm, normalized to the optical density at 600 nm) as percentage of a wt sfGFP reference. The data represent the mean and standard deviation of 3 biological replicates.

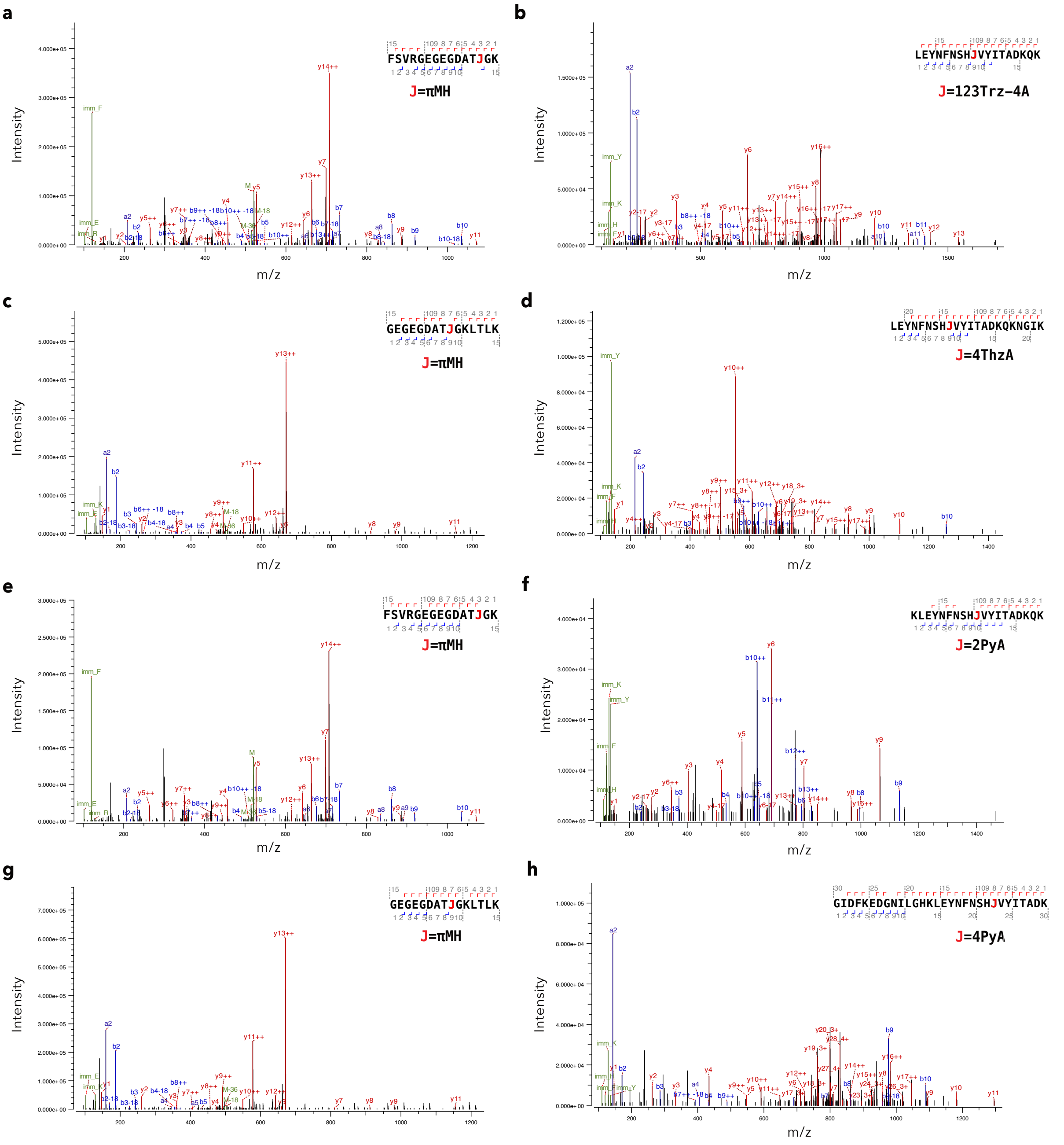

Supplementary Figure 19 | LC-MS/MS spectra of sfGFP40$_{TGA}$150$_{TAG}$ containing two histidine-like ncAAs. Representative LC-MS/MS spectra from tryptic digests of sfGFP40$_{TGA}$150$_{TAG}$ expressed with different combinations of aaRS/tRNA pairs. In all cases, several peptides containing the desired ncAA at the respective position were observed. a, b) sfGFP40$_{\pi MH}$150$_{123Trz\text{-}4A}$; c, d) sfGFP40 $_{\pi MH}$150$_{4ThzA}$; e, f) sfGFP40$_{\pi MH}$150$_{2PyA}$; g, h) sfGFP40 $_{\pi MH}$ 150$_{4PyA}$.

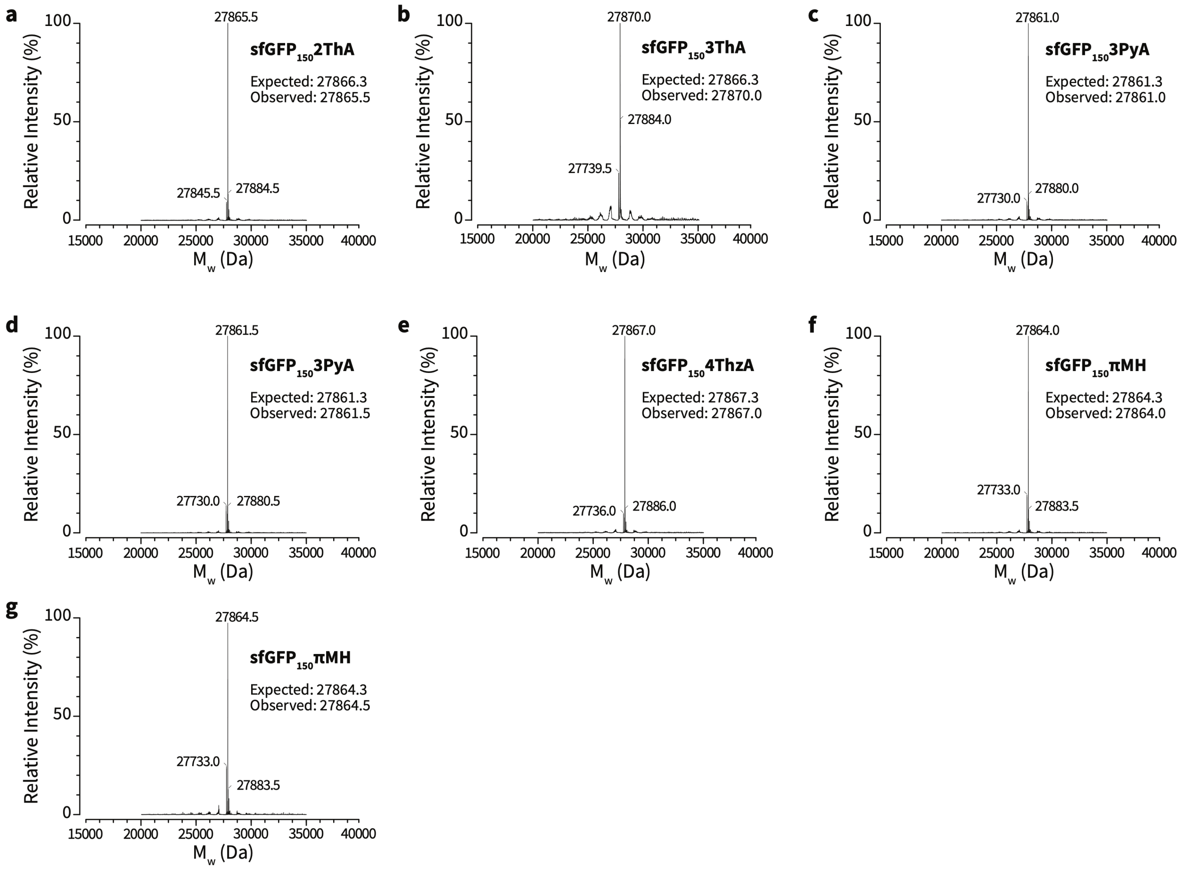

Supplementary Figure 20 | LC-MS spectra of sfGFP150$_{TAG}$ containing a single histidine-like ncAA incorporated by previously reported aaRS/tRNA pairs. Intact LC-MS analysis (positive electrospray time of flight) of sfGFP150$_{TAG}$ production in the presence of ncAAs with their cognate aaRS/tRNA pair. a) *Mb*PylRS^{QF} with 2ThA. b) *Mm*PylRS$^{7\text{-}1}$ with 3ThA. c) *Mm*PylRS^{FLF} with 3PyA. d) *Mm*PylRS$^{8\text{-}2}$ with 3PyA. e) *Mb*PylRS^{4ThzA} with 4ThzA. f) *Mm*PylRS^{IFGFF} with πMH. g) *G1*PylRS^{MIFAF} with πMH. The expected and observed masses confirming incorporation of the desired ncAA are indicated. Additional peaks, consistent with typically observed [H_2O] elimination (-18 Da), [Na]$^+$ addition (+23 Da) or loss of N-terminal [Met] (-131 Da) were observed.

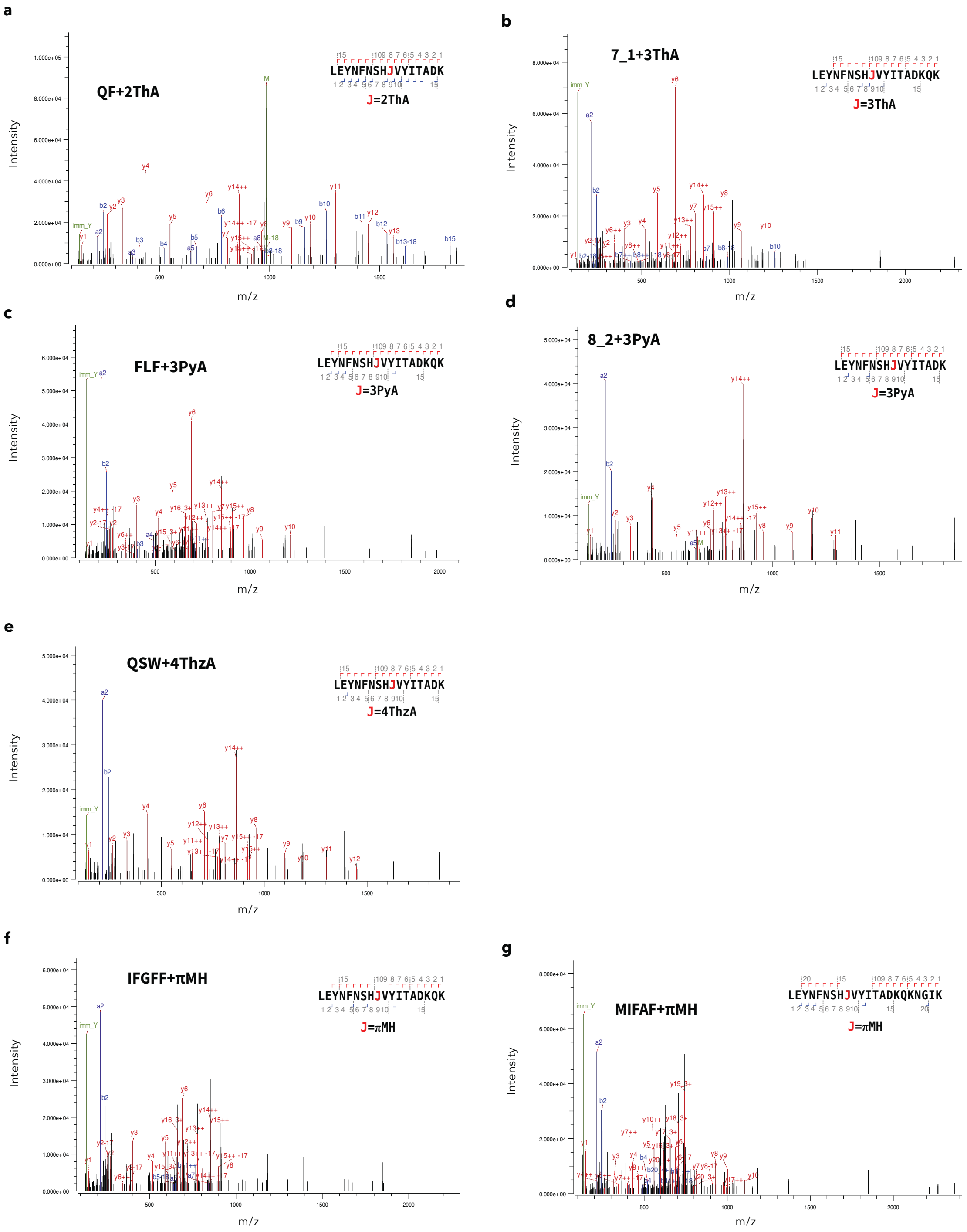

Supplementary Figure 21 | LC-MS/MS spectra of sfGFP150$_{TAG}$ containing a single histidine-like ncAA incorporated by previously reported aaRS/tRNA pairs. A representative LC-MS/MS spectrum from a tryptic digest of sfGFP150$_{TAG}$ expressed with different ncAAs and their corresponding aaRS/tRNA pairs. a) *Mb*PylRS^{QF} with 2ThA. b) *Mm*PylRS^{7_1} with 3ThA. c) *Mm*PylRS^{FLF} with 3PyA. d) *Mm*PylRS^{8_2} with 3PyA. e) *Mb*PylRS^{4ThzA} with 4ThzA. f) *Mm*PylRS^{IFGFF} with πMH. g) *G1*PylRS^{MIFAF} with πMH. Typically, multiple peptides containing the desired ncAA were observed and no peptides for canonical amino acid incorporation were observed. For samples of sfGFP150-3ThA and both aaRSs of sfGFP150-3PyA, some peptides containing canonical amino acids were observed (see **Supplementary Table 9**).

II. Synthesis

1,2,4-Triazol-3-yl-alanine hydrochloride synthesis (124Trz-3A)

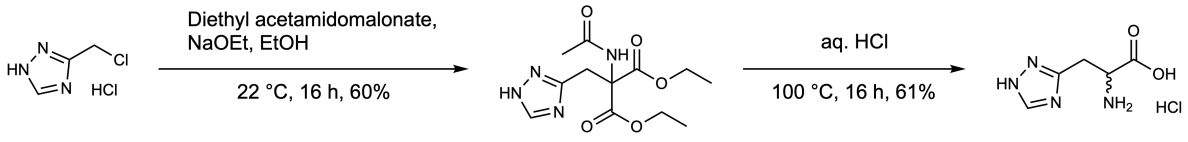

Diethyl-2-acetamido-2-(1,2,4-triazol-3-ylmethyl)malonate

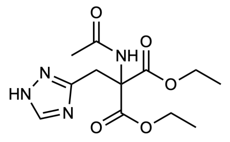

The reaction was performed under a nitrogen atmosphere. Sodium ethoxide (21% in EtOH, 3.6 mL, 9.7 mmol, 3.8 eq.) was dissolved in dry EtOH (2 mL) and cooled to 0 °C. Diethyl acetamidomalonate (1.11 g, 5.10 mmol, 2.0 eq.) was dissolved in dry EtOH (13 mL) and was added dropwise to the sodium ethoxide solution. The reaction mixture was stirred at room temperature for 2 h. 1,2,3-Triazol-3-ylmethylchloride hydrochloride (393 mg, 2.55 mmol, 1.0 eq.) was dissolved in dry EtOH (6 mL) and added to the malonate solution. The reaction mixture was stirred at room temperature for 16 h and quenched with the addition of water. The solvents were removed *in vacuo* and the crude material was dissolved in water. The solution was adjusted to pH 6 with aq. HCl (4 M) and was extracted with EtOAc (6x). The combined organic phase was dried over Na_2SO_4 and the solvent was removed *in vacuo* to yield diethyl-2-acetamido-2-(1,2,4-triazol-3-ylmethyl)malonate (456 mg, 1.53 mmol, 60%) as a yellow solid.

^{1}H-NMR (400 MHz, $CDCl_3$) δ 7.96 (s, 1H), 6.87 (s, 1H), 4.36 – 4.20 (m, 4H), 3.87 (s, 2H), 1.99 (s, 3H), 1.26 (t, $^3J_{HH}$ = 7.1 Hz, 6H).

1,2,4-Triazol-3-yl-alanine hydrochloride (124Trz-3A)

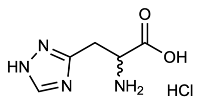

Diethyl-2-acetamido-2-(1,2,4-triazol-3-ylmethyl)malonate (2.11 g, 7.06 mmol, 1.0 eq.) was suspended in aq. HCl (4 M, 36 mL) and stirred at 100 °C for 16 h. The solvent was removed *in vacuo*, the crude product was dissolved in EtOH (14 mL) and aniline (700 µL) was added to precipitate 1,2,4-triazol-3-yl-alanine hydrochloride (836 mg, 4.28 mmol, 61%) as a beige solid with aniline as an impurity.[7]

^{1}H-NMR (400 MHz, D_2O) δ 8.37 (s, 1H), 4.15 (dd, $^3J_{HH}$ = 7.9, 5.0 Hz, 1H), 3.44 (dd, $^2J_{HH}$ = 16.0 Hz, $^3J_{HH}$ = 5.0 Hz, 1H), 3.33 (dd, $^2J_{HH}$ = 16.0 Hz, $^3J_{HH}$ = 7.9 Hz, 1H).

^{13}C NMR (101 MHz, D_2O) δ 172.7, 156.4, 145.9, 53.2, 28.0.

MS (ESI): calc. for $[M+H]^+$: 157.06, obs.: 157.1.

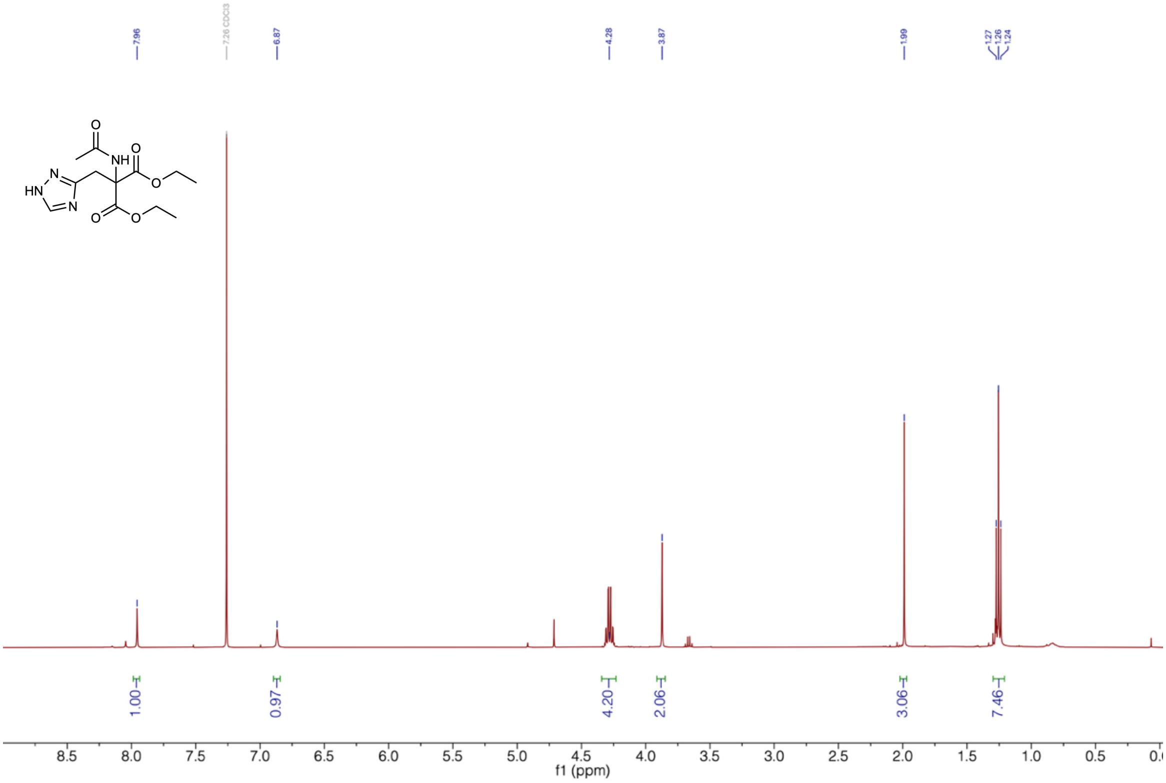

Supplementary Figure 22 | ^{1}H-NMR of diethyl-2-acetamido-2-(1,2,4-triazol-3-ylmethyl)malonate.

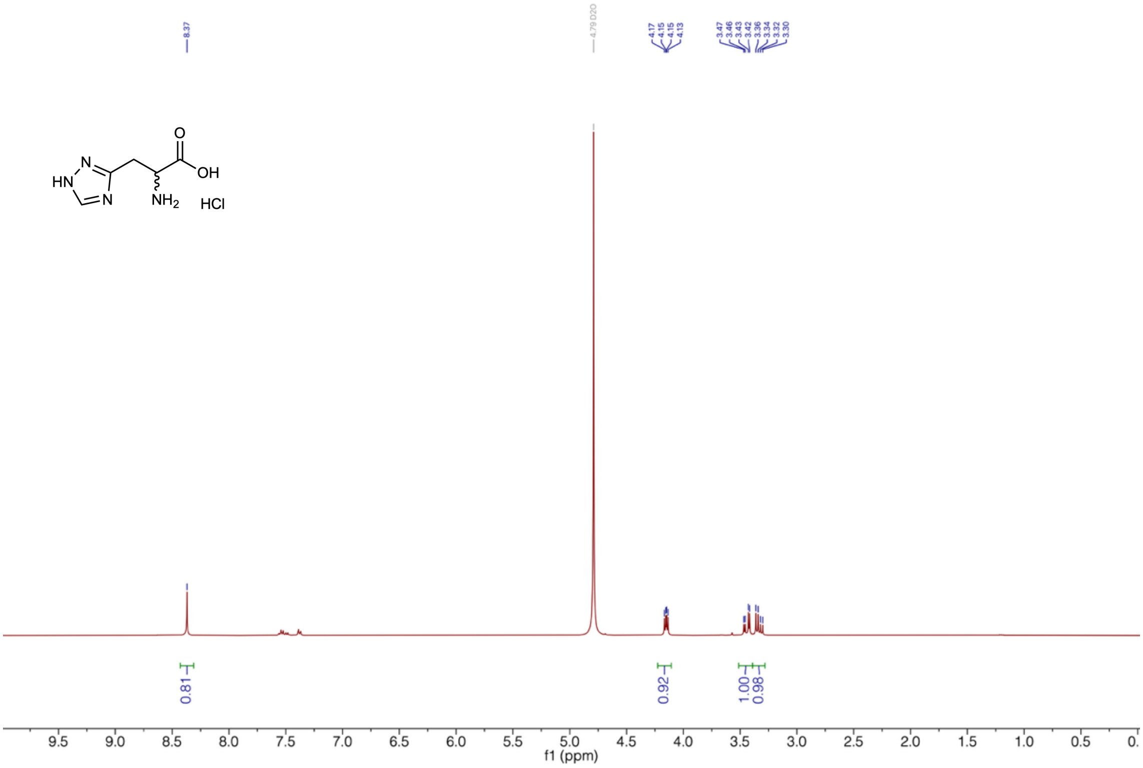

Supplementary Figure 23 | ^{1}H-NMR of 1,2,4-triazol-3-yl-alanine hydrochloride (124Trz-3A).

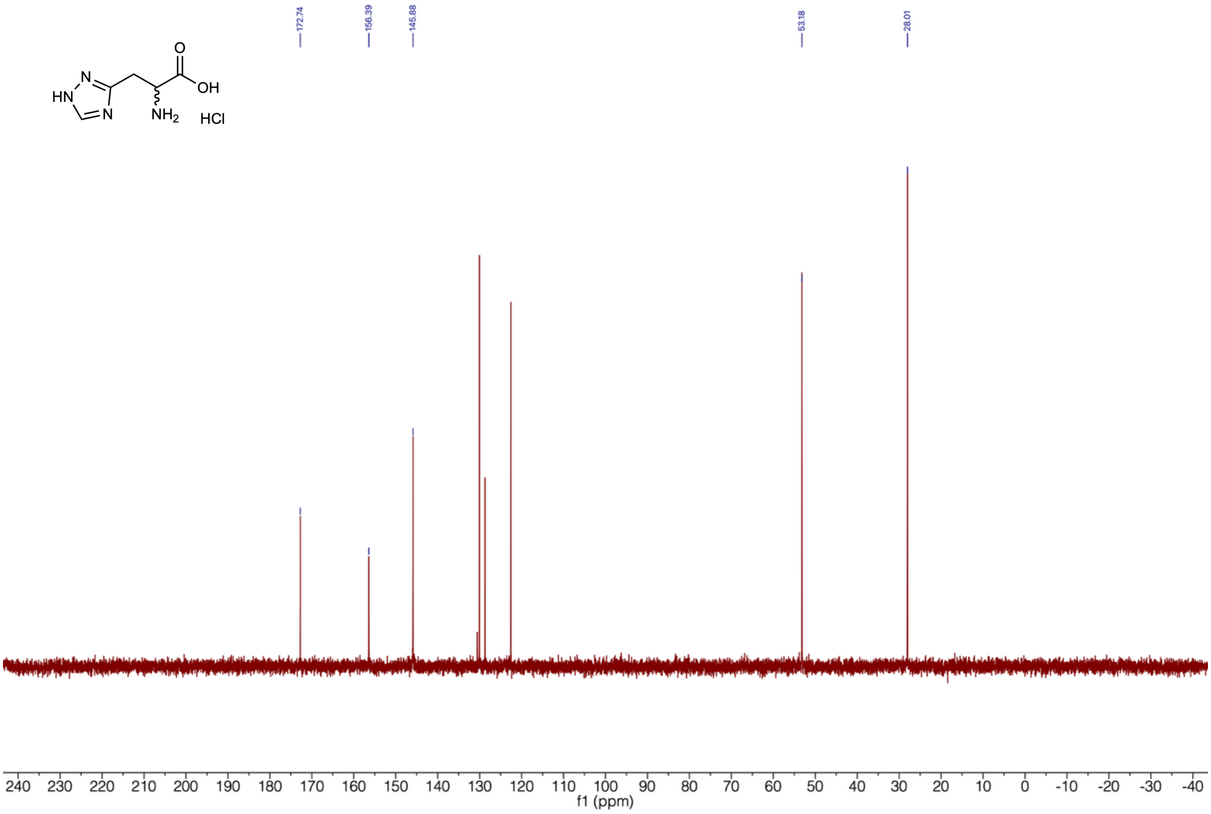

Supplementary Figure 24 | ^{13}C-NMR of 1,2,4-triazol-3-yl-alanine hydrochloride (124Trz-3A).

5-Nitro-L-histidine hydrochloride synthesis ($5NO_2H$):

5-Nitro-L-histidine hydrochloride was synthesized as previously described[8].

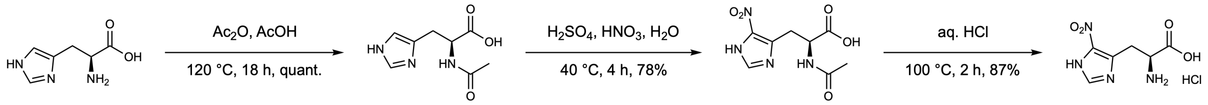

N-acetyl-L-histidine:

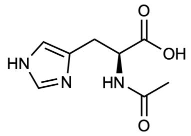

L-histidine (2.00 g, 12.8 mmol, 1.0 eq) was suspended in conc. acetic acid (10 mL). Acetic anhydride (1.2 mL, 13 mmol, 1.0 eq) was added and the solution was stirred at 120 °C for 18 h. The reaction mixture was allowed to reach room temperature, and the solvent was removed *in vacuo*. The residue was resuspended in water (5 mL) and the solvent was removed *in* vacuo. This step was repeated two more times to yield *N*-acetyl-L-histidine (2.52 g, 12.8 mmol, quant.) as a white powder.

N-acetyl- 5-nitro-L-histidine:

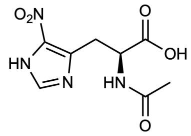

N-acetyl-L-histidine (2.52 g, 12.8 mmol, 1.0 eq.) was suspended in conc. sulfuric acid (20 mL) and cooled to 0 °C. Aq. nitric acid (65%, 8 mL) was added dropwise, and the reaction mixture was stirred at 40 °C for 4 h. The solution was allowed to reach room temperature and was poured into ice-cold water (100 mL). The pH was adjusted to pH 4 with aq. sat. K_2CO_3. The solvent was removed *in vacuo*. The crude product was resuspended in methanol, filtered, and the filtrate was concentrated *in vacuo* to yield *N*-acetyl-5-nitro-L-histidine (1.50 g, 7.49 mmol, 78%) as a colorless oil.

5-Nitro-L-histidine hydrochloride (5NO2H):

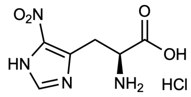

N-acetyl-5-nitro-L-histidine (1.00 g, 4.12 mmol, 1.0 eq.) was suspended in aq. HCl (2 M, 10 mL) and stirred at 100 °C for 2h. The solvent was removed *in vacuo* and the crude product was purified by automated flash column chromatography (C18, 0 – 95% acetonitrile in water) to yield 5-nitro-L-histidine hydrochloride as an off white solid (850 mg, 3.59 mmol, 87%).

^{1}H-NMR (400 MHz, D_2O) δ 7.75 (s, 1H), 4.44 (dd, $^{3}J_{HH}$ = 7.2, 6.6 Hz, 1H), 3.73 (dd, $^{2}J_{HH}$ = 14.9 Hz, $^{3}J_{HH}$ = 7.2 Hz, 1H), 3.66 (dd, $^{2}J_{HH}$ = 14.9 Hz, $^{3}J_{HH}$ = 6.6 Hz, 1H).

^{13}C-NMR (101 MHz, D_2O) δ 170.3, 143.8, 135.1, 128.1, 51.5, 26.0.

MS (ESI): calc. for $[M+H]^+$: 201.05, obs.: 200.9.

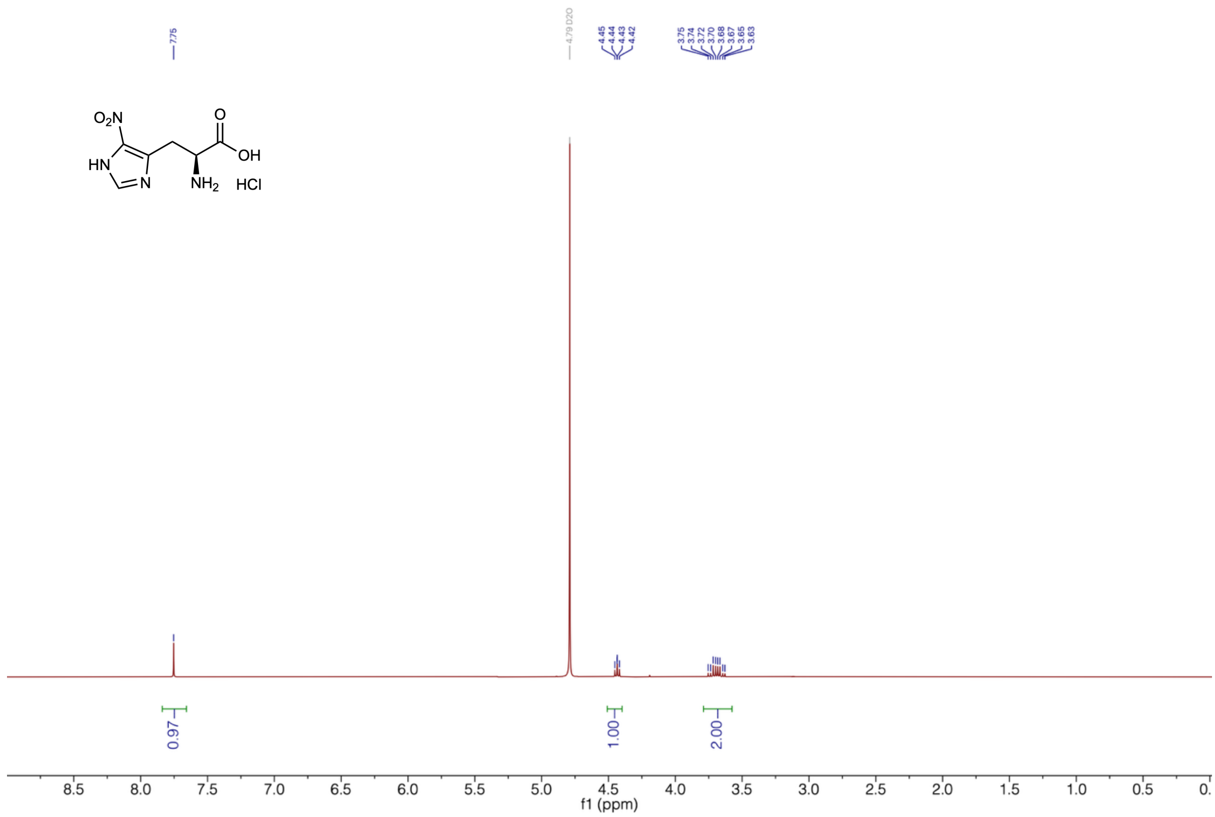

Supplementary Figure 25 | ^{1}H-NMR of 5-nitro-L-histidine hydrochloride (5NO_2H).

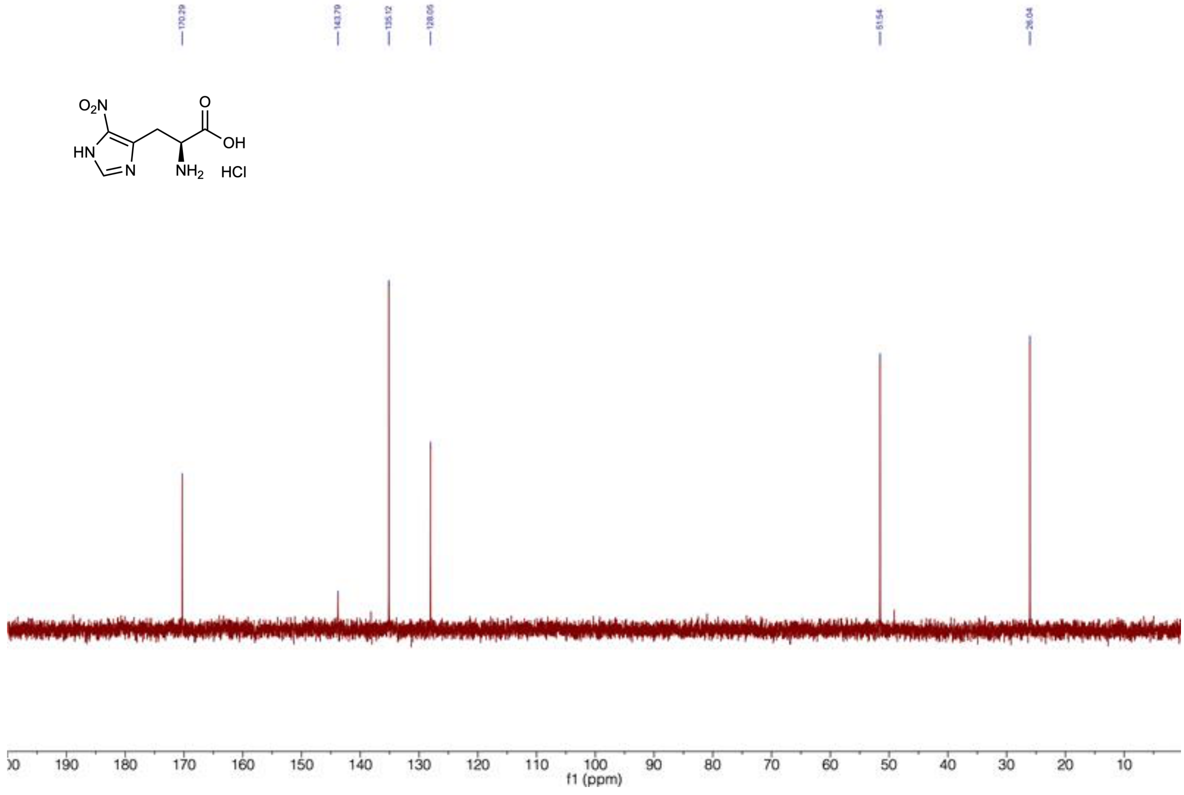

Supplementary Figure 26 | ^{13}C-NMR of 5-nitro-L-histidine hydrochloride (5NO_2H).

4-Pyridyl-alanine hydrochloride synthesis (4PyA)

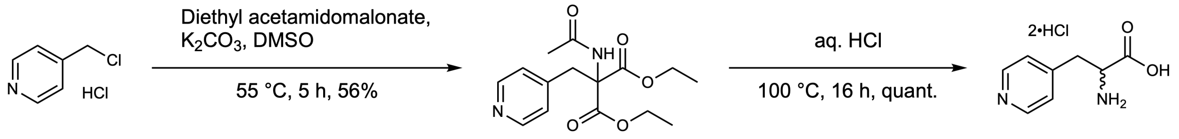

Diethyl-2-acetamido-2-(pyridin-4-ylmethyl) malonate:

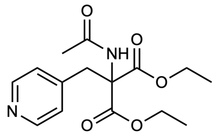

The following reaction was adapted from literature[9]. 4-Picolylchloride hydrochloride (2.18 g, 13.3 mmol, 1.0 eq.) was dissolved in DMSO (20 mL). Diethyl acetamidomalonate (2.91 g, 13.4 mmol, 1.0 eq) and K_2CO_3 (3.73 g, 27.0 mmol, 2.0 eq.) were added and the reaction was stirred at 55 °C for 5h. The reaction mixture was allowed to reach room temperature, was poured into ice-cold water (40 mL), and EtOAc (100 mL) was added. The mixture was centrifuged for 10 min at 4'000 g. The phases were separated and EtOAc (100 mL) was added to the aqueous phase. The mixture was centrifuged for 10 min at 4'000 g. The combined organic phase was washed with brine, dried over $MgSO_4$, and the solvent was removed *in vacuo* to yield diethyl-2-acetamido-2-(pyridin-4-ylmethyl)malonate (2.31 g, 7.49 mmol, 56%) as a brown solid.

^{1}H-NMR (400 MHz, DMSO-d$_6$) δ 8.49 – 8.44 (m, 2H), 7.03 – 6.96 (m, 2H), 4.21 – 4.10 (m, 4H), 3.44 (s, 2H), 2.54 (s, 3H), 1.17 (t, $^3J_{HH}$ = 7.1 Hz, 6H).

4-Pyridyl-alanine hydrochloride:

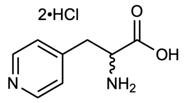

Diethyl-2-acetamido-2-(pyridin-4-ylmethyl)malonate (1.45 g, 4.70 mmol, 1.0 eq.) was suspended in aq. HCl (4 M, 20 mL) and stirred at 100 °C for 16 h. The solvent was removed *in vacuo* and the crude product was purified by automated flash column chromatography (C18, 0-95% acetonitrile in water) to yield 4-pyridyl alanine hydrochloride (953 mg, 4.70 mmol, quant.) as an off white solid.

^{1}H-NMR (400 MHz, D_2O) δ 8.76 (d, $^3J_{HH}$ = 6.8 Hz, 2H), 8.05 (d, $^3J_{HH}$ = 6.8 Hz, 2H), 4.47 – 4.41 (m, 1H), 3.66 – 3.52 (m, 2H).

^{13}C-NMR (101 MHz, H_2O+D_2O) δ 170.7, 156.8, 141.2, 128.2, 52.9, 35.8.

MS (ESI): calc. for $[M+H]^+$: 167.07, obs.: 167.2.

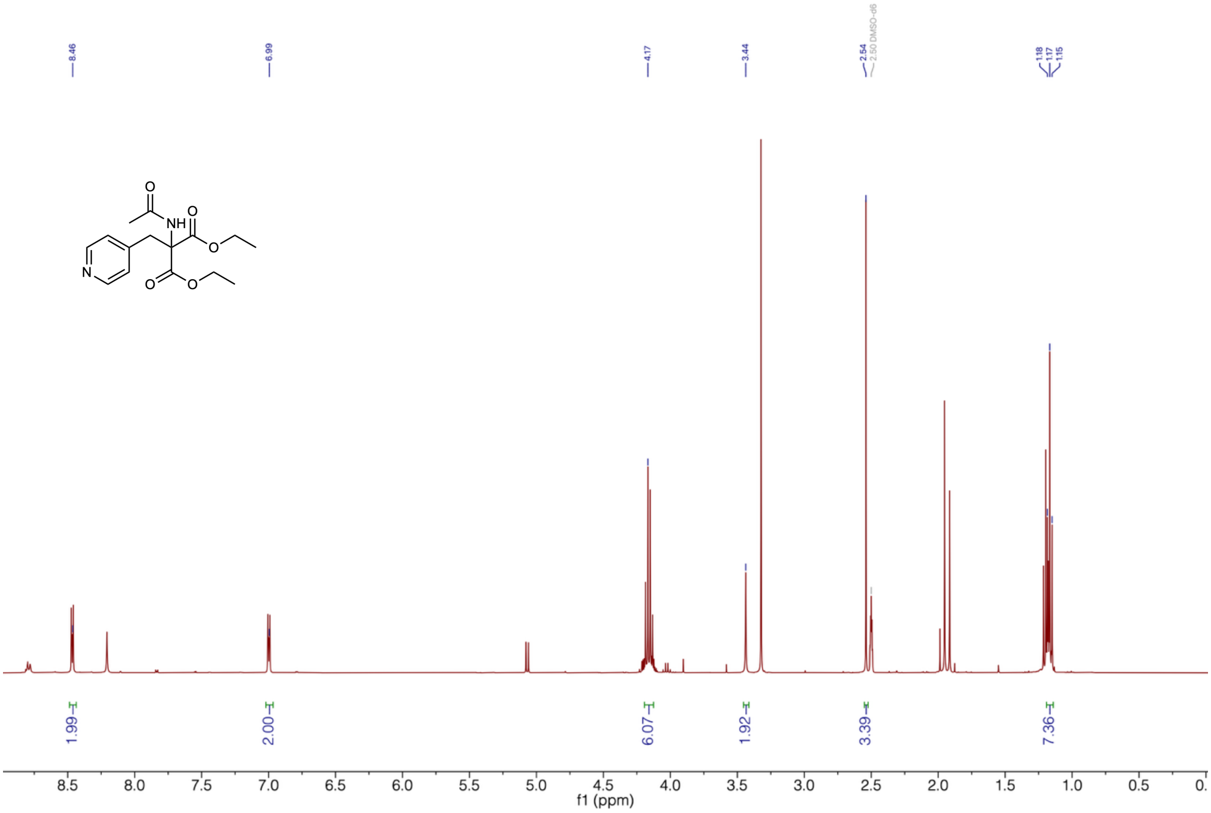

Supplementary Figure 27 | ¹H-NMR of diethyl-2-acetamido-2-(pyridin-4-ylmethyl) malonate.

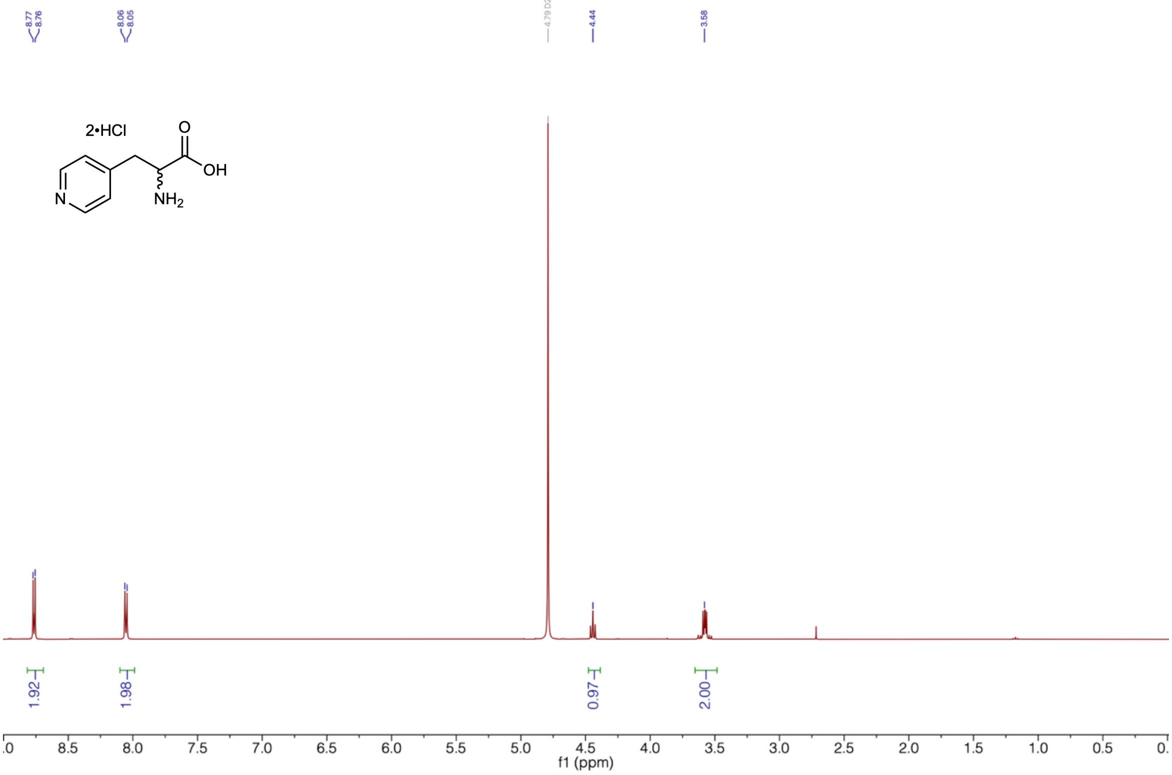

Supplementary Figure 28 | ^{1}H-NMR of 4-pyridyl-alanine (4PyA).

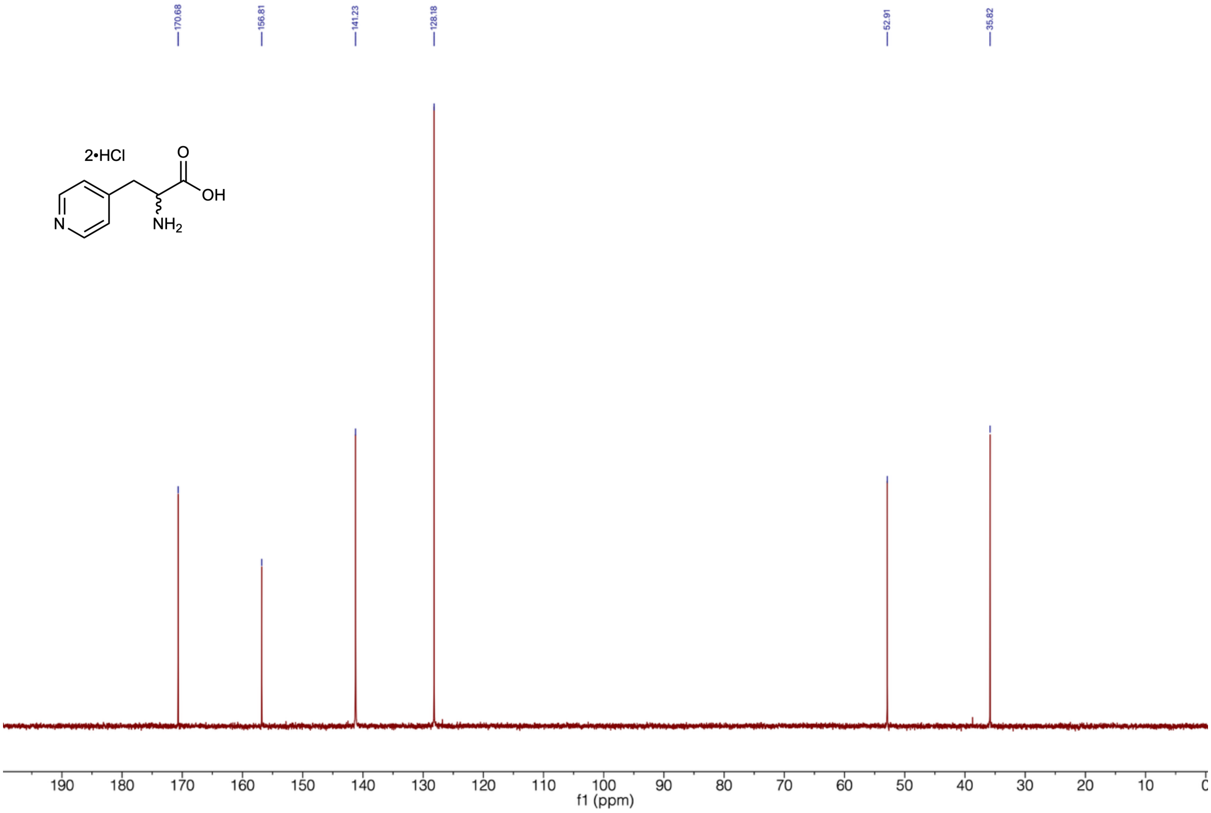

Supplementary Figure 29 | ^{13}C-NMR of 4-pyridyl-alanine (4PyA).

1,2,3-Triazol-4-yl-L-alanine synthesis (123Trz-4A):

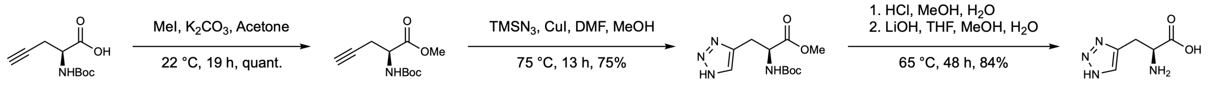

<u>*N*-Boc-propargyl-L-glycine-OMe</u>

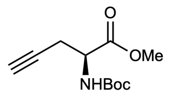

The following reaction was adapted from literature[10]. *N*-Boc-propargylglycine (5.00 g, 22.7 mmol, 1.0 eq.) was dissolved in acetone (100 mL). K_2CO_3 (6.37 g, 46.1 mmol, 2.0 eq.) and methyl iodide (2.9 mL, 46 mmol, 2.0 eq.) were added and the suspension was stirred at room temperature for 19 h. The solvent was removed *in vacuo* and the crude product was dissolved in ethyl acetate. The organic phase was washed with water and brine and dried over Na_2SO_4. The solvent was removed *in vacuo* to yield *N*-boc-propargyl-L-glycine-OMe (5.36 g, 22.7 mmol, quant.) as a yellow-orange oil.

^{1}H-NMR (400 MHz, $CDCl_3$) δ 5.34 (d, $^3J_{HH}$ = 8.6 Hz, 1H), 4.54 – 4.42 (m, 1H), 3.78 (s, 3H), 2.80 – 2.66 (m, 2H), 2.04 (t, $^4J_{HH}$ = 2.0 Hz, 1H), 1.45 (s, 9H).

<u>*N*-Boc-1,2,3-triazol-4-yl-L-alanine-OMe</u>

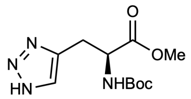

N-Boc-propargyl-L-glycine-OMe (1.10 g, 4.84 mmol, 1.0 eq.) was dissolved in dry dimethylformamide (5 mL) and dry methanol (1 mL) under an inert atmosphere. Trimethylsilylazide (0.97 mL, 7.3 mmol, 1.5 eq.) was added. Copper iodide (86 mg, 10 mol%) was added and the reaction mixture was stirred at 60 °C for 5 h and then at 75 °C for 13 h. The suspension was allowed to reach room temperature and was then further cooled to 4 °C. The reaction mixture was quenched by the addition of water, and the solution was extracted with EtOAc (2 x 50 mL). The combined organic phase was dried over Na_2SO_4, and the solvent was removed *in vacuo*. The crude product was purified by flash column chromatography (SiO_2, 50-100% ethyl acetate in cyclohexane) to yield *N*-Boc-1,2,3-triazol-4-yl-L-alanine-OMe (983 mg, 4.84 mmol, 75%) as a yellow sticky oil.

R_f (cyclohexane:ethyl acetate, 1:1): 0.2, brown spot with ninhydrin stain.

^{1}H-NMR (400 MHz, $CDCl_3$) δ 7.52 (s, 1H), 5.43 (d, $^3J_{HH}$ = 8.2 Hz, 1H), 4.72 – 4.62 (m, 1H), 3.74 (s, 3H), 3.30 – 3.23 (m, 2H), 1.43 (s, 9H).

<u>1,2,3-Triazol-4-yl-L-alanine (123Trz-4A)</u>

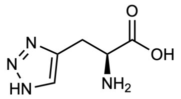

N-Boc-1,2,3-triazol-4-yl-alanine-OMe (2.95 g, 10.9 mmol, 1.0 eq.) was dissolved in methanol (70 mL) and aq. HCl (conc., 4.5 mL, 54 mmol, 5.0 eq.) was added. The solution was stirred at 40 °C for 17 h, followed by 50 °C for 4 h. The solvent was removed *in vacuo* to yield an orange-brown oil. The oil was dissolved in a mixture of THF, methanol, and water (15 mL each) and $LiOH•H_2O$ (1.15 g, 42.0 mmol, 2.5 eq.) was added. The suspension was stirred at 65 °C for 2 days. The solvents were removed *in vacuo* and the crude product was purified by preparative HPLC chromatography (C18, 50-90% acetonitrile in water (0.1% formic acid)) to yield 1,2,3-triazol-4-yl-L-alanine (1.43 g, 10.9 mmol, 84%) as a white solid.

^{1}H-NMR (400 MHz, D_2O) δ 7.82 (s, 1H), 4.10 (dd, $^3J_{HH}$ = 6.8, 5.3 Hz, 1H), 3.43 – 3.31 (m, 1H).

^{13}C-NMR (101 MHz, D_2O) δ 173.0, 140.2, 127.1, 54.2, 25.9.

MS (ESI): calc. for $[M+H]^+$: 157.06, obs.: 157.1.

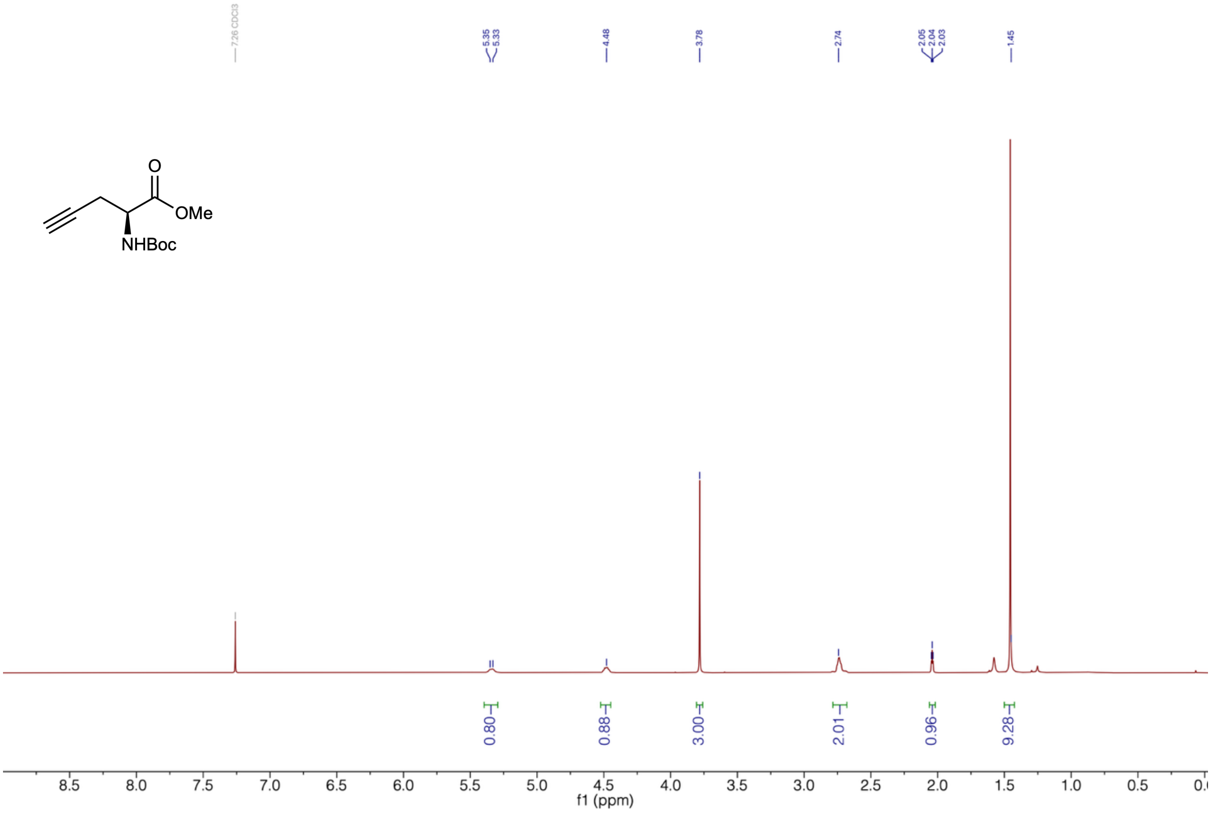

Supplementary Figure 30 | ^{1}H-NMR of *N*-Boc-propargyl-L-glycine-OMe.

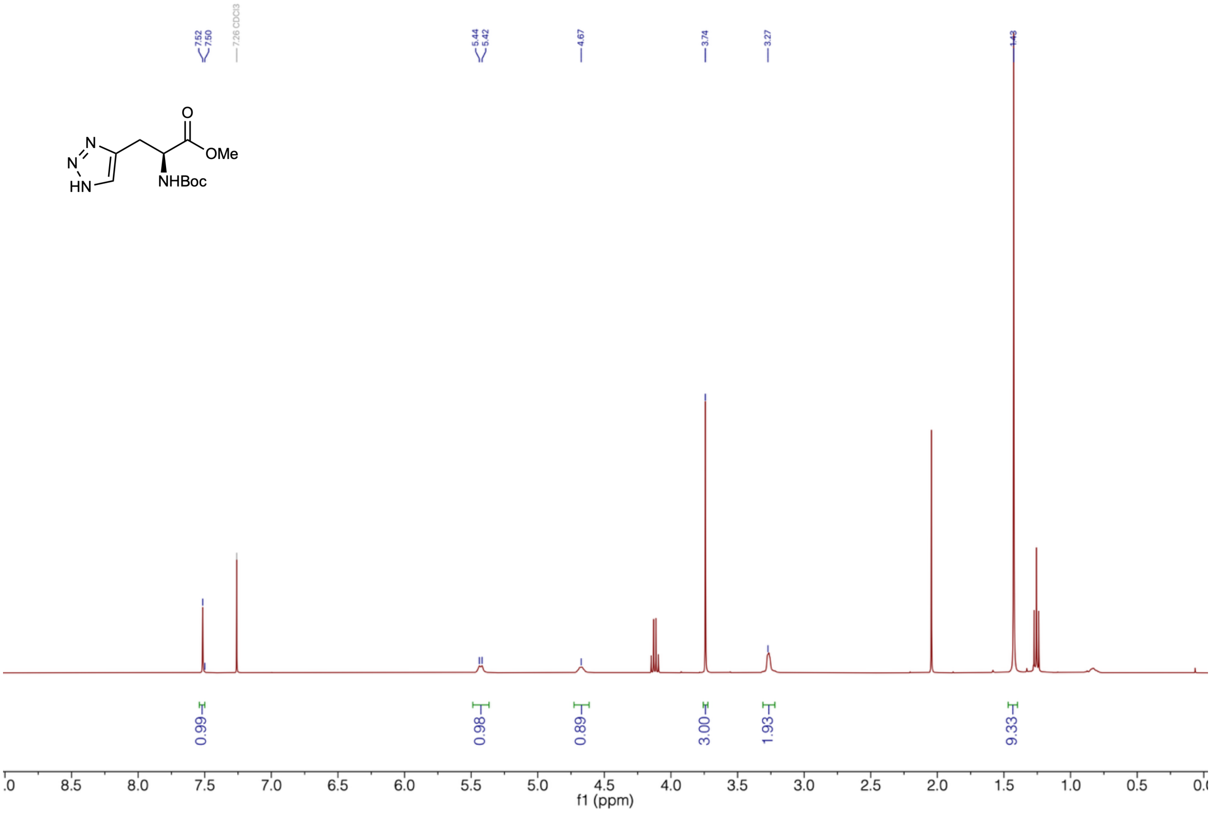

Supplementary Figure 31 | ^{1}H-NMR of *N*-Boc-1,2,3-triazol-4-yl-L-alanine-OMe.

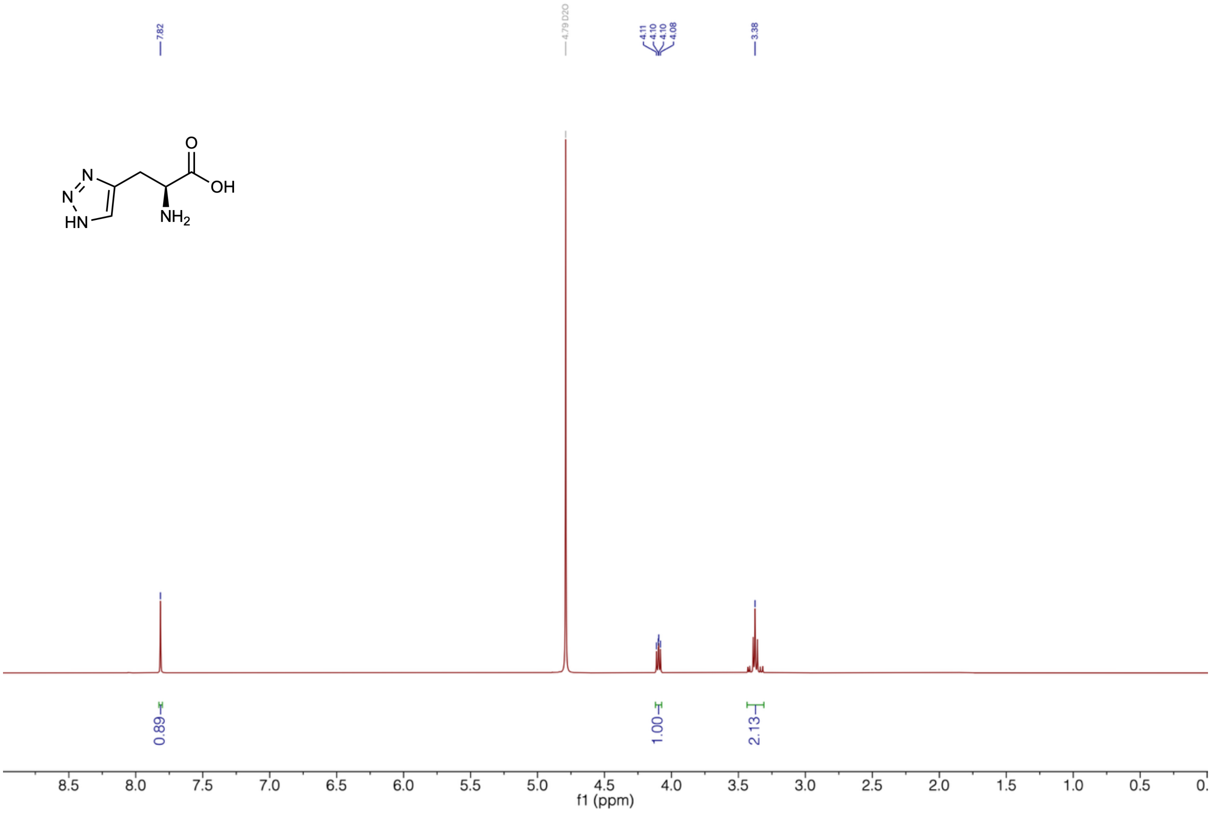

Supplementary Figure 32 | ^{1}H-NMR of 1,2,3-triazol-4-yl-L-alanine (123Trz-4A).

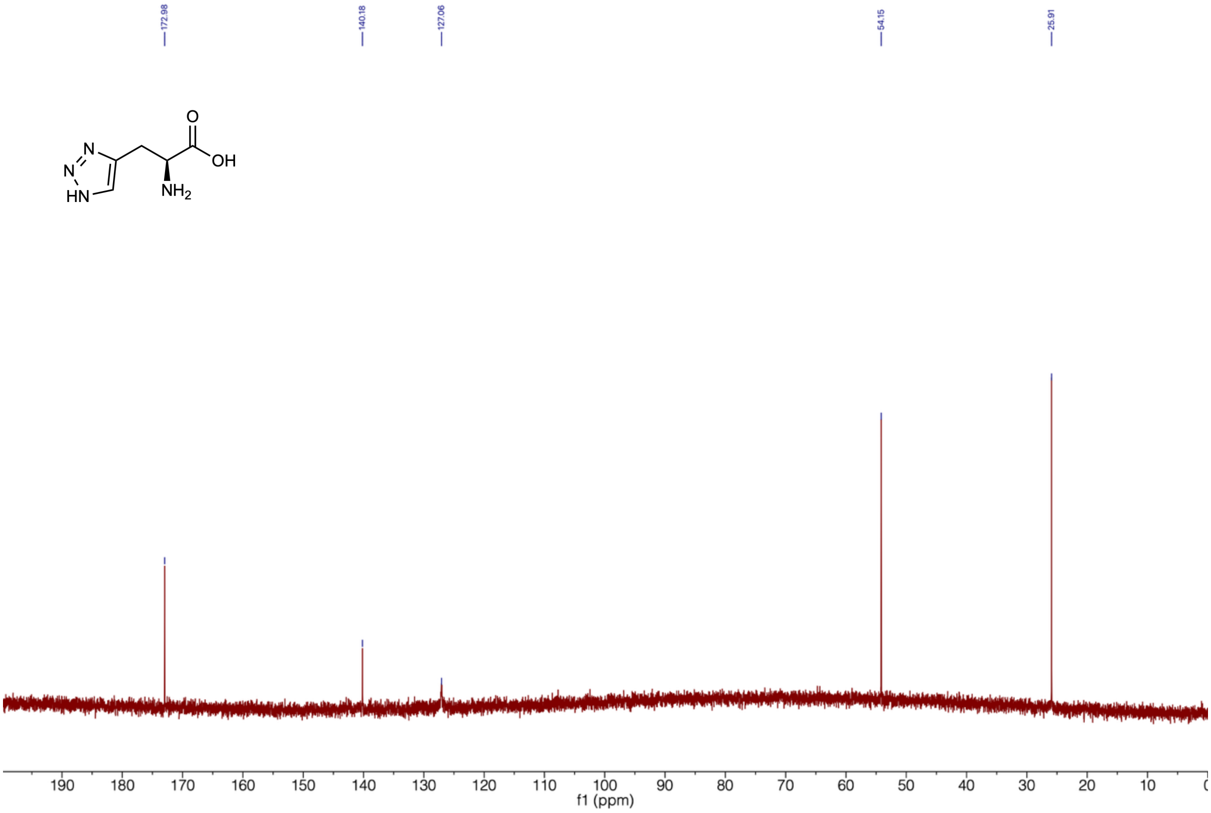

Supplementary Figure 33 | ^{13}C-NMR of 1,2,3-triazol-4-yl-L-alanine (123Trz-4A).

1-Benzyl-1,2,3-triazol-4-yl-L-alanine synthesis (1Bn123Trz-4A):

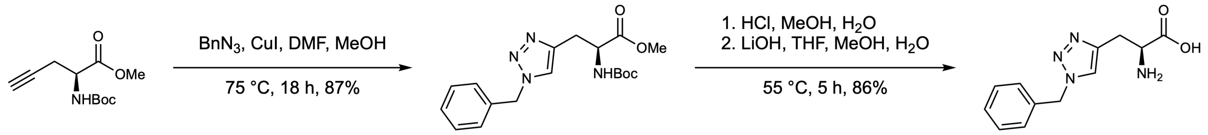

<u>*N*-Boc-1-benzyl-1,2,3-triazol-4-yl-L-alanine-OMe</u>

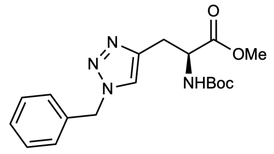

N-boc-propargyl-L-glycine-OMe (1.02 g, 4.48 mmol, 1.0 eq.) and benzylazide (913 mg, 6.86 mmol, 1.5 eq.) were added to a mixture of dry DMF (5 mL) and dry MeOH (1 mL) under a nitrogen atmosphere. CuI (82 mg, 430 µmol, 10 mol%) was added and a white precipitate occurred, that vanished after a short amount of time. The solution was stirred at 75 °C for 18 h. The dark green solution was cooled to 4 °C and quenched with water (25 mL). The mixture was extracted with EtOAc (2 x 125 mL), and the combined organic phases were washed with aq. LiCl (5%, 2 x 50 mL) and brine (1 x 100 mL). The organic phase was dried over Na_2SO_4 and the solvent was evaporated *in vacuo* to yield a green solid. The crude product was purified by automated flash column chromatography (SiO_2, 50-100% EtOAc in cyclohexane) to yield *N*-Boc-1-benzyl-1,2,3-triazol-4-yl-L-alanine-OMe as a green solid (1.40 g, 3.88 mmol, 87%).

Rf (EtOAc): 0.75, brown spot with ninhydrin stain.

^{1}H-NMR (400 MHz, $CDCl_3$) δ 7.41 – 7.29 (m, 3H), 7.21 (d, $^3J_{HH}$ = 7.1 Hz, 2H), 5.69 – 5.45 (m, 3H), 4.66 (s, 1H), 3.70 (s, 3H), 3.14 (br. s, 2H), 1.40 (s, 9H).

<u>1-Benzyl-1,2,3-triazol-4-yl-L-alanine (1Bn123Trz-4A)</u>

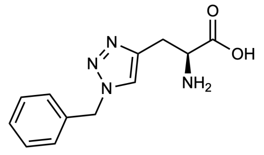

N-Boc-1-benzyl-1,2,3-triazol-4-yl-L-alanine-OMe (1.40 g, 3.88 mmol, 1.0 eq.) was dissolved in methanol (24 mL) and aq. HCl (conc., 1.6 mL, 19 mmol, 5.0 eq.) was added. The orange-green solution was stirred at room temperature for 5 h. The solvent was removed *in vacuo* to yield a green glue. The crude product was purified by automated flash column chromatography (C18, 5-90% acetonitrile in water (0.1% formic acid) to yield 1-benzyl-1,2,3-triazol-4-yl-L-alanine-OMe that was already partially deprotected and directly used for the next step. Crude 1-benzyl-1,2,3-triazol-4-yl-L-alanine-OMe was dissolved in THF, MeOH, and water (6 mL each). LiOH monohydrate (488 mg, 11.6 mmol, 3.0 eq.) was added and the reaction mixture was stirred at 55 °C for 30 min. The solvents were removed *in vacuo* to yield 1-benzyl-1,2,3-triazol-4-yl-L-alanine (821 mg, 3.33 mmol, 86%) as a pale blue solid.

^{1}H-NMR (400 MHz, MeOD) δ 8.05 (s, 1H), 7.43 – 7.31 (m, 5H), 5.65 (s, 2H), 4.37 (dd, $^3J_{HH}$ = 5.7 Hz, 1H), 3.43 (dd, $^2J_{HH}$ = 15.7, $^3J_{HH}$ = 5.7 Hz, 1H), 3.40 – 3.30 (m, 1H).

^{13}C-NMR (101 MHz, MeOD) δ 170.5, 136.2, 130.1, 129.8, 129.4, 126.0, 55.5, 53.3, 26.9.

MS (ESI): calc. for $[M+H]^+$: 247.11, obs.: 247.1.

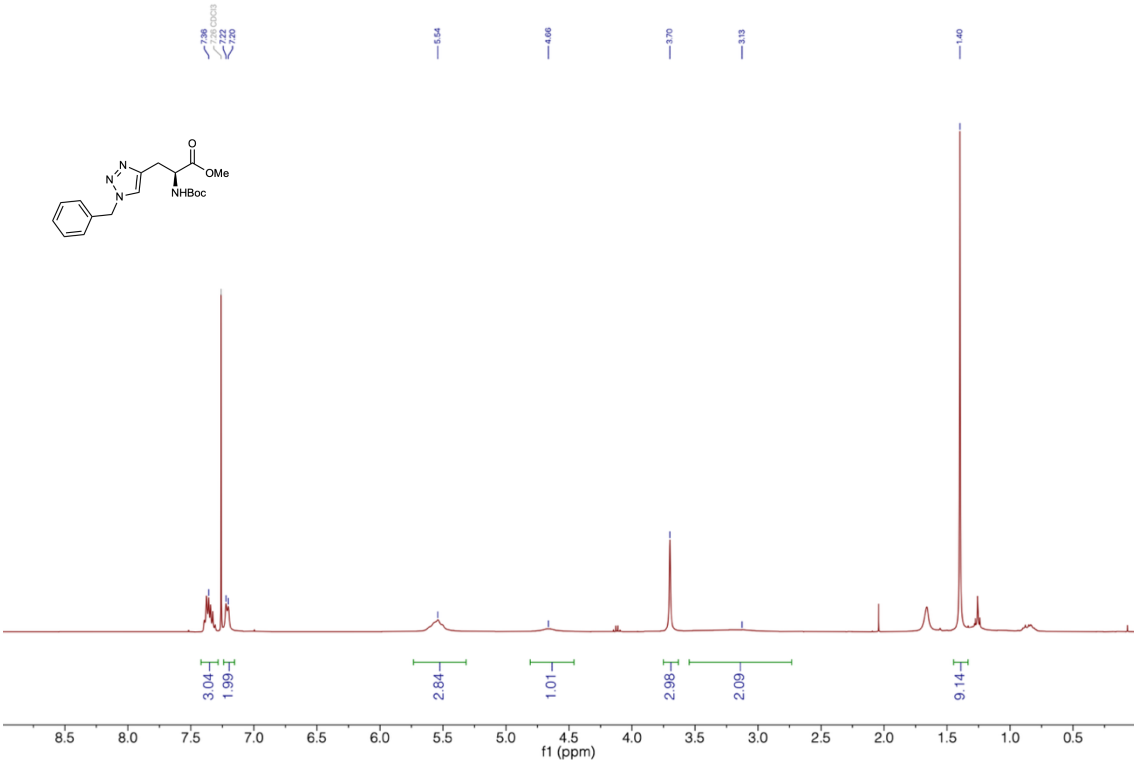

Supplementary Figure 34 | ^{1}H-NMR of *N*-Boc-1-benzyl-1,2,3-triazol-4-yl-L-alanine-OMe.

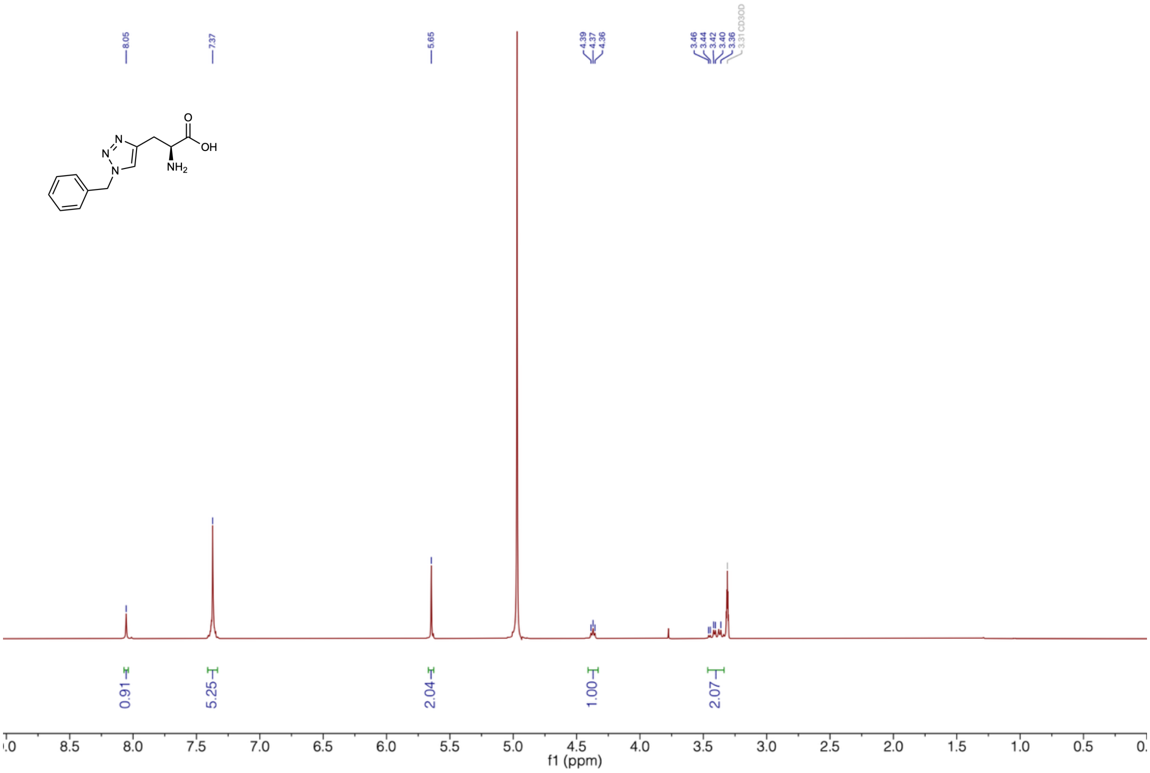

Supplementary Figure 35 | ^{1}H-NMR of 1-benzyl-1,2,3-triazol-4-yl-L-alanine (1Bn123Trz-4A).

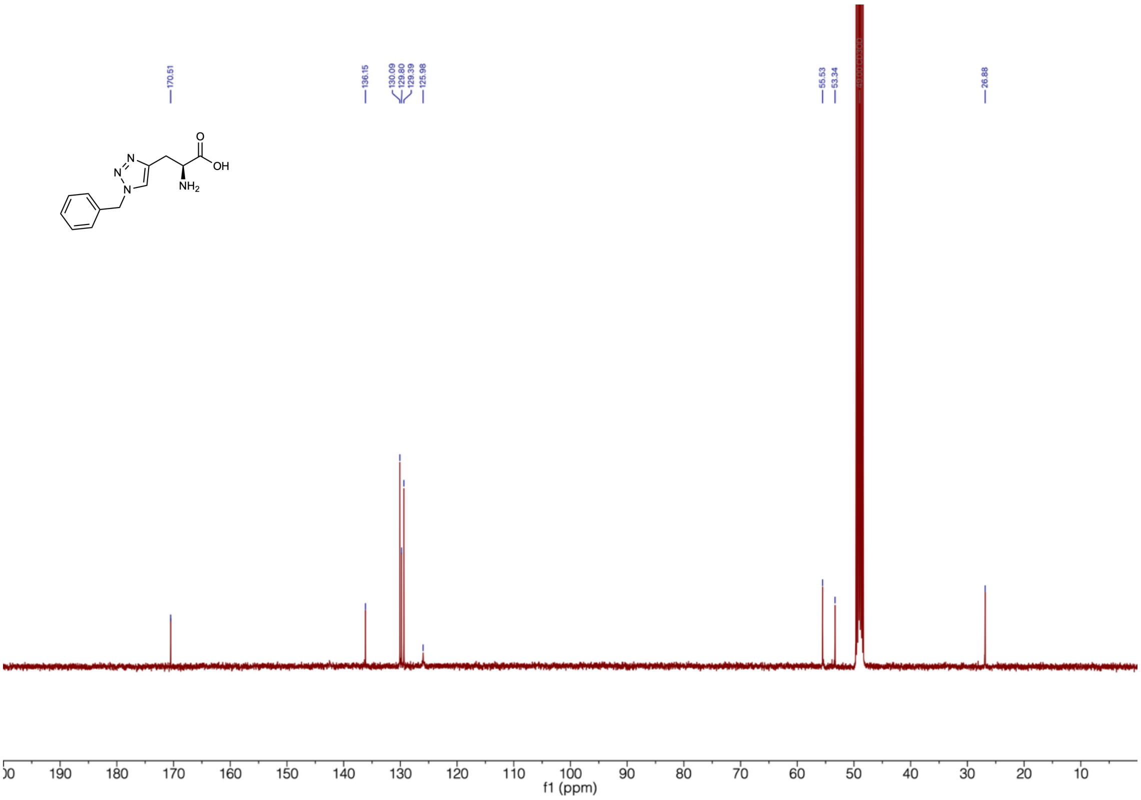

Supplementary Figure 36 | ^{13}C-NMR of 1-benzyl-1,2,3-triazol-4-yl-L-alanine (1Bn123Trz-4A).

Pyrazol-3-yl-alanine hydrochloride synthesis (3PzA):

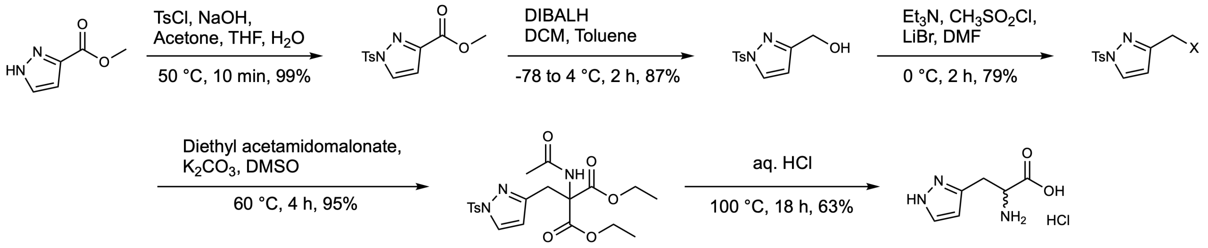

Methyl 1-tosyl-pyrazole-3-carboxylate

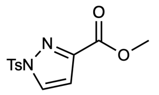

The synthesis of methyl 1-tosyl-pyrazole-3-carboxylate was adapted from literature[11]. *p*-Toluenesulfonylchloride (831 mg, 4.36 mmol, 1.1 eq.) was dissolved in acetone (6 mL). Aq. NaOH (2.5 M, 1.75 mL, 4.36 mmol, 1.1 eq.), tetrahydrofuran (1.5 mL) and methyl pyrazole-3-carboxylate (500 mg, 3.96 mmol, 1.0 eq.) were added. The reaction was stirred at 50 °C for 10 min in a microwave reactor. The suspension was diluted with dichloromethane (40 mL). The mixture was washed with water, aq. sat. $NaHCO_3$, aq. HCl (0.5 M), and brine. The organic phase was dried over $MgSO_4$ and the solvent was removed *in vacuo* to yield methyl 1-tosyl-pyrazole-3-carboxylate (1.10 g, 3.92 mmol, 99%) as a white solid.

^{1}H-NMR (400 MHz, $CDCl_3$) δ 8.13 (d, $^3J_{HH}$ = 2.8 Hz, 1H), 7.95 (d, $^3J_{HH}$ = 8.2 Hz, 2H), 7.35 (d, $^3J_{HH}$ = 8.2 Hz, 2H), 6.87 (d, $^3J_{HH}$ = 2.8 Hz, 1H), 3.90 (s, 3H), 2.43 (s, 3H).

(1-tosyl-pyrazol-3-yl)methanol

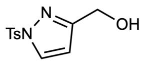

Methyl 1-tosyl-pyrazole-3-carboxylate (7.94 g, 28.3 mmol, 1.0 eq.) was dissolved in dry dichloromethane under a nitrogen atmosphere and the solution was cooled to -78 °C. DIBALH (25% in toluene, 30.1 mL, 65.0 mmol, 2.3 eq.) was added dropwise. The reaction stirred for 2 h while allowing to reach 4 °C. The reaction was quenched with water (2.7 mL), aq. NaOH (10%, 4 mL), and water (6.6 mL). The suspension was stirred for 15 min and filtered over celite. The filter cake was washed extensively with dichloromethane and the filtrate was washed with brine. The organic phase was dried over $MgSO_4$ and the solvent was removed *in vacuo* to yield (1-tosyl-pyrazol-3-yl)methanol (6.24 g, 24.7 mmol, 87%) as a white solid.

^{1}H-NMR (400 MHz, $CDCl_3$) δ 8.06 (d, $^3J_{HH}$ = 2.8 Hz, 1H), 7.87 (d, $^3J_{HH}$ = 8.2 Hz, 2H), 7.32 (d, $^3J_{HH}$ = 8.2 Hz, 2H), 6.41 (d, $^3J_{HH}$ = 2.8 Hz, 1H), 4.67 (s, 2H), 2.42 (s, 3H).

(1-tosyl-pyrazol-3-yl)methylchloride/bromide

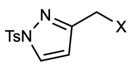

(1-tosyl-pyrazol-3-yl)methanol (12.5 g, 49.4 mmol, 1.0 eq.) was dissolved in dry DMF (100 mL) under a nitrogen atmosphere and triethylamine (9.0 mL, 65 mmol, 1.3 eq.) was added. The solution was cooled to 4 °C and methane sulfonyl chloride (5.0 mL, 64 mmol, 1.3 eq.) was added dropwise. The reaction was stirred for 45 min and lithium bromide (10.0 g, 115 mmol, 2.3 eq.) was added. The suspension was stirred for 2 h and the reaction was quenched with water (200 mL). The suspension was extracted with EtOAc (2 x 125 mL) and the combined organic phase was washed with water, aq. sat. $NaHCO_3$, aq. LiCl (5%), and brine. The organic phase was dried over $MgSO_4$ to yield a mixture of (1-tosyl-pyrazol-3-yl)methylchloride and (1-tosyl-pyrazol-3-yl)methylbromide (10 mol% (1-tosyl-pyrazol-3-yl)methylbromide, 10.8 g, 39.1 mmol, 79%) as an off-white solid.

^{1}H-NMR (400 MHz, $CDCl_3$) δ 8.06 (d, $^3J_{HH}$ = 2.8 Hz, 1H, Cl), 8.05 (d, $^3J_{HH}$ = 2.8 Hz, 1H, Br), 7.89 (d, $^3J_{HH}$ = 8.2 Hz, 2H), 7.34 (d, $^3J_{HH}$ = 8.2 Hz, 1H), 6.48 (d, $^3J_{HH}$ = 2.8 Hz, 1H, Cl), 6.47 (d, $^3J_{HH}$ = 2.8 Hz, 1H, Br), 4.54 (s, 2H, Cl), 4.39 (s, 2H, Br), 2.43 (s, 3H).

Diethyl-2-acetamido-2-((1-tosyl-pyrazol-3-yl)methyl)malonate

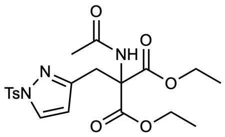

The following reaction was adapted from literature[9]. (1-Tosyl-pyrazol-3-yl)methylchloride/bromide (39.1 mmol, 1.0 eq.) was dissolved in DMSO (75 mL). Diethyl acetamidomalonate (8.61 g, 39.6 mmol, 1.0 eq.) and K_2CO_3 (9.42 g, 68.2 mmol, 1.7 eq.) were added and the suspension was stirred at 60 °C for 4 h. The reaction mixture was allowed to reach room temperature and ice-cold water (500 mL) was added. The mixture was extracted with EtOAc (2 x 250 mL) and the combined organic phases were washed with brine (250 mL). The organic phase was dried over $MgSO_4$ and the solvent was removed *in vacuo* to yield diethyl-2-acetamido-2-((1-tosyl-pyrazol-3-yl)methyl)malonate (16.8 g, 37.2 mmol, 95%) as a yellow solid.

^{1}H-NMR (400 MHz, $CDCl_3$) δ 7.97 (d, $^3J_{HH}$ = 2.6 Hz, 1H), 7.82 (d, $^3J_{HH}$ = 8.2 Hz, 2H), 7.33 (d, $^3J_{HH}$ = 8.2 Hz, 2H), 6.55 (s, 1H), 6.13 (d, $^3J_{HH}$ = 2.6 Hz, 1H), 4.19 (q, $^3J_{HH}$ = 7.1 Hz, 4H), 3.66 (s, 2H), 2.42 (s, 3H), 1.89 (s, 3H), 1.23 (t, $^3J_{HH}$ = 7.1 Hz, 6H).

Pyrazol-3-yl-alanine hydrochloride (3PzA):

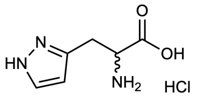

Diethyl-2-acetamido-2-((1-tosyl-pyrazol-3-yl)methyl)malonate (16.8 g, 37.2 mmol, 1.0 eq.) was dissolved in aq. HCl (4 M, 250 mL) and stirred at 100 °C for 16 h. The solvent was removed *in vacuo* and the crude product was purified by automated flash column chromatography (C18, 1% acetonitrile

in water (0.1% formic acid)) to yield pyrazol-3-yl-alanine hydrochloride (4.50 g, 23.5 mmol, 63%) as a white solid.

^{1}H-NMR (400 MHz, D_2O) δ 7.98 (d, $^{3}J_{HH}$ = 2.6 Hz, 1H), 6.61 (d, $^{3}J_{HH}$ = 2.6 Hz, 1H), 4.42 (t, $^{3}J_{HH}$ = 6.4 Hz, 1H), 3.54 – 3.39 (m, 2H).

^{13}C-NMR (101 MHz, D_2O) δ 170.4, 142.9, 134.0, 107.1, 52.0, 26.4.

MS (ESI): calc. for $[M+H]^+$: 156.07, obs.: 156.1.

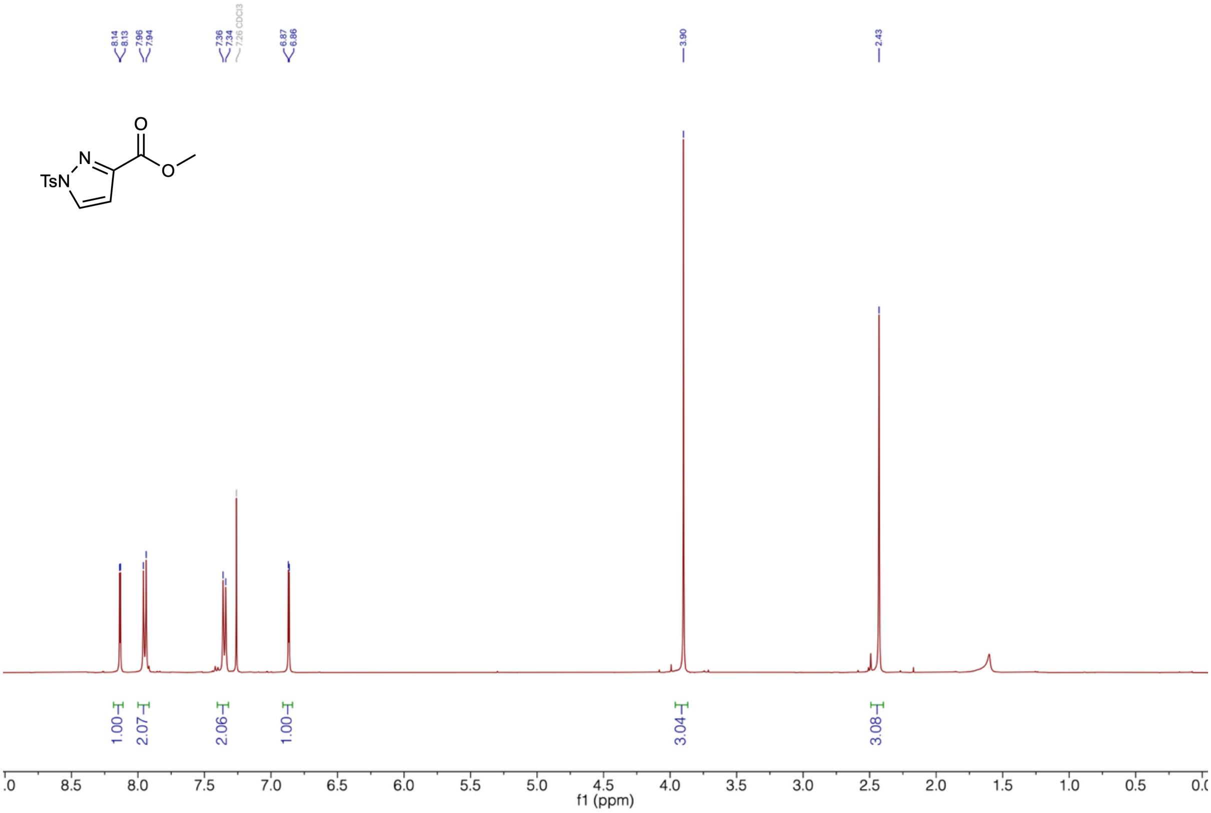

Supplementary Figure 37 | ^{1}H-NMR of Methyl 1-tosyl-pyrazole-3-carboxylate.

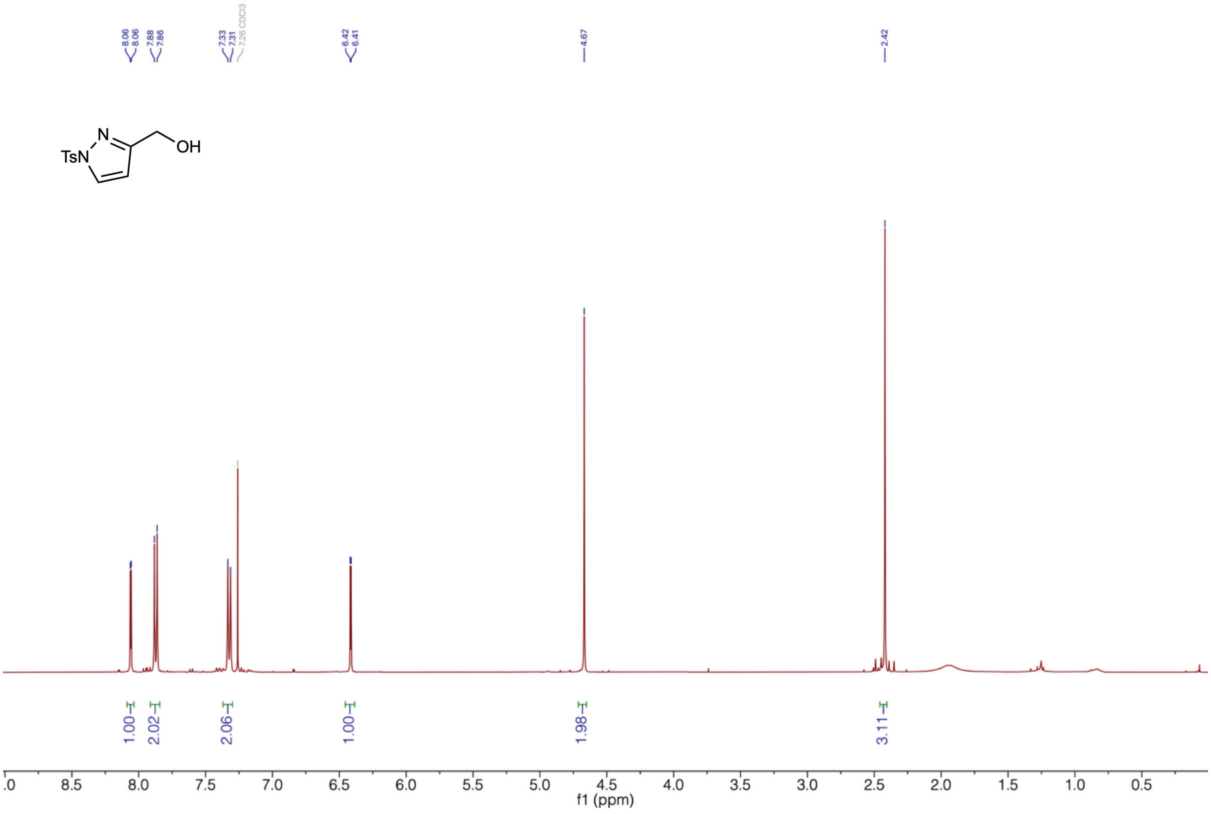

Supplementary Figure 38 | ^{1}H-NMR of (1-tosyl-pyrazol-3-yl)methanol.

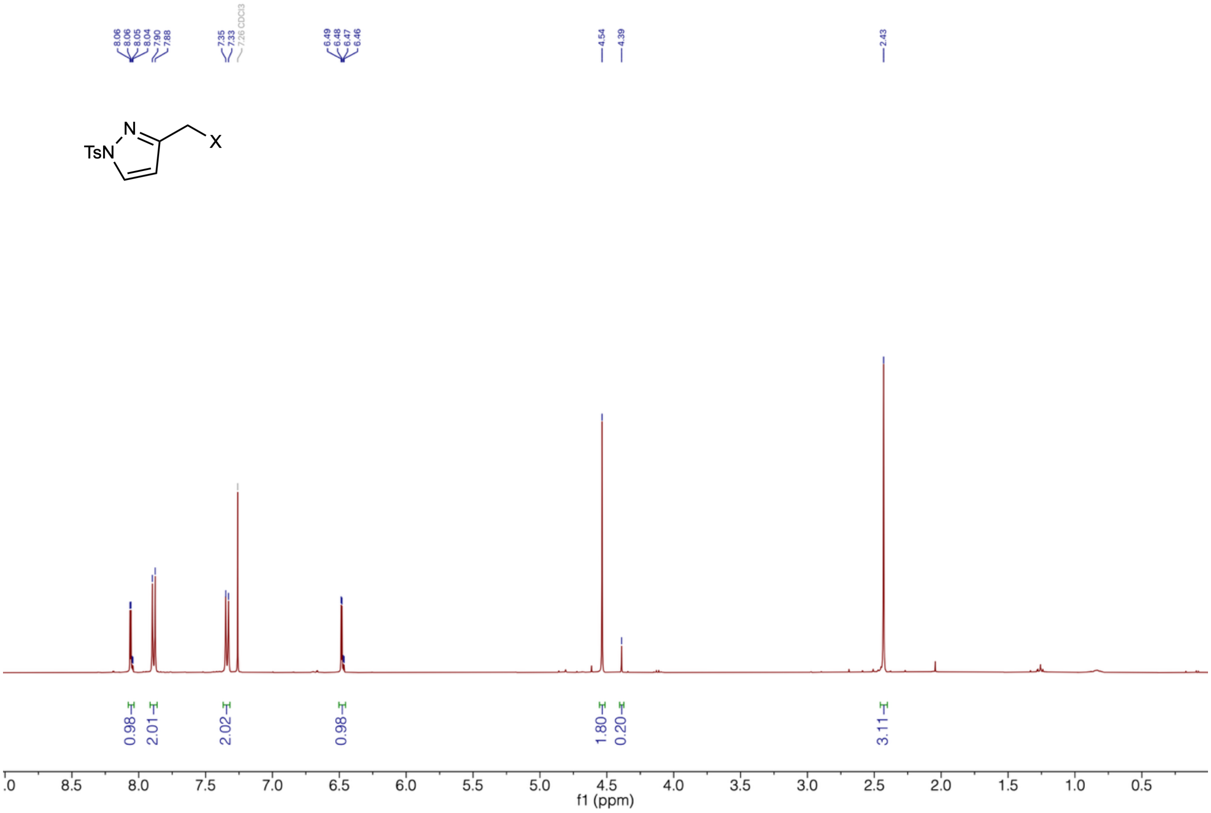

Supplementary Figure 39 | ^{1}H-NMR of (1-tosyl-pyrazol-3-yl)methylchloride/bromide.

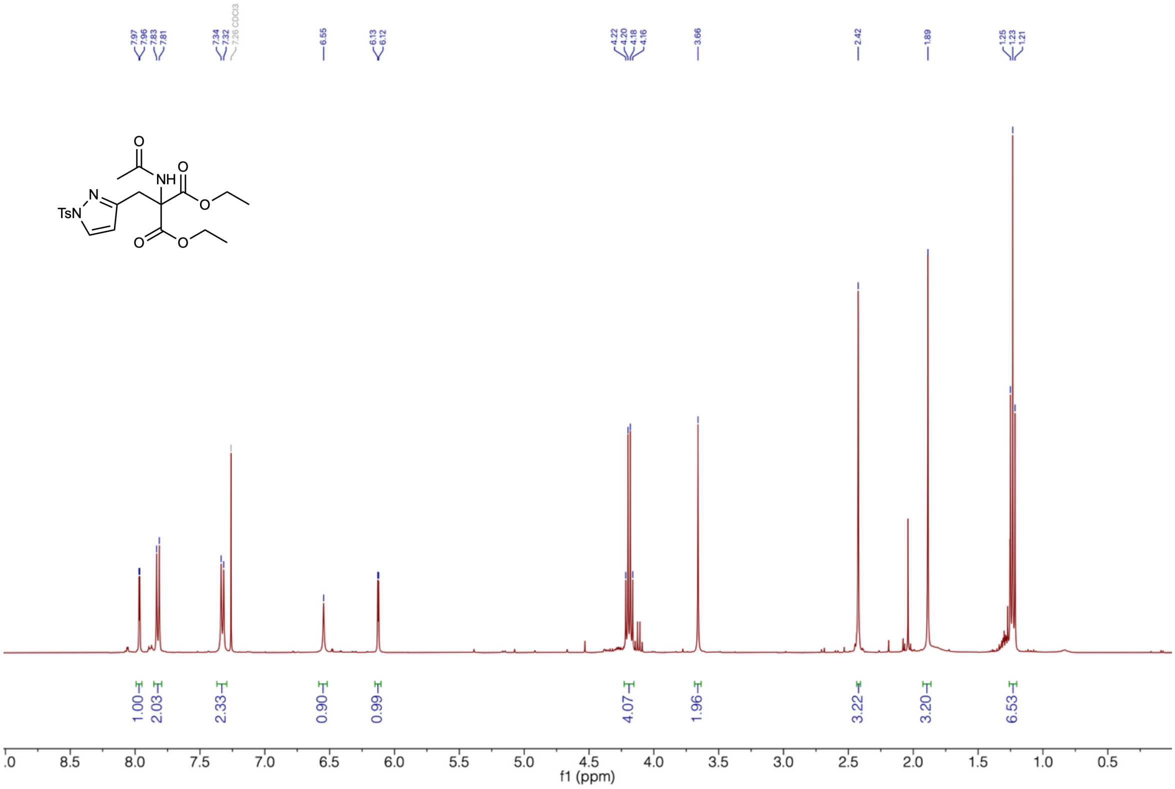

Supplementary Figure 40 | ^{1}H-NMR of Diethyl-2-acetamido-2-(*N*-tosyl-pyrazol-3-yl-methyl)malonate.

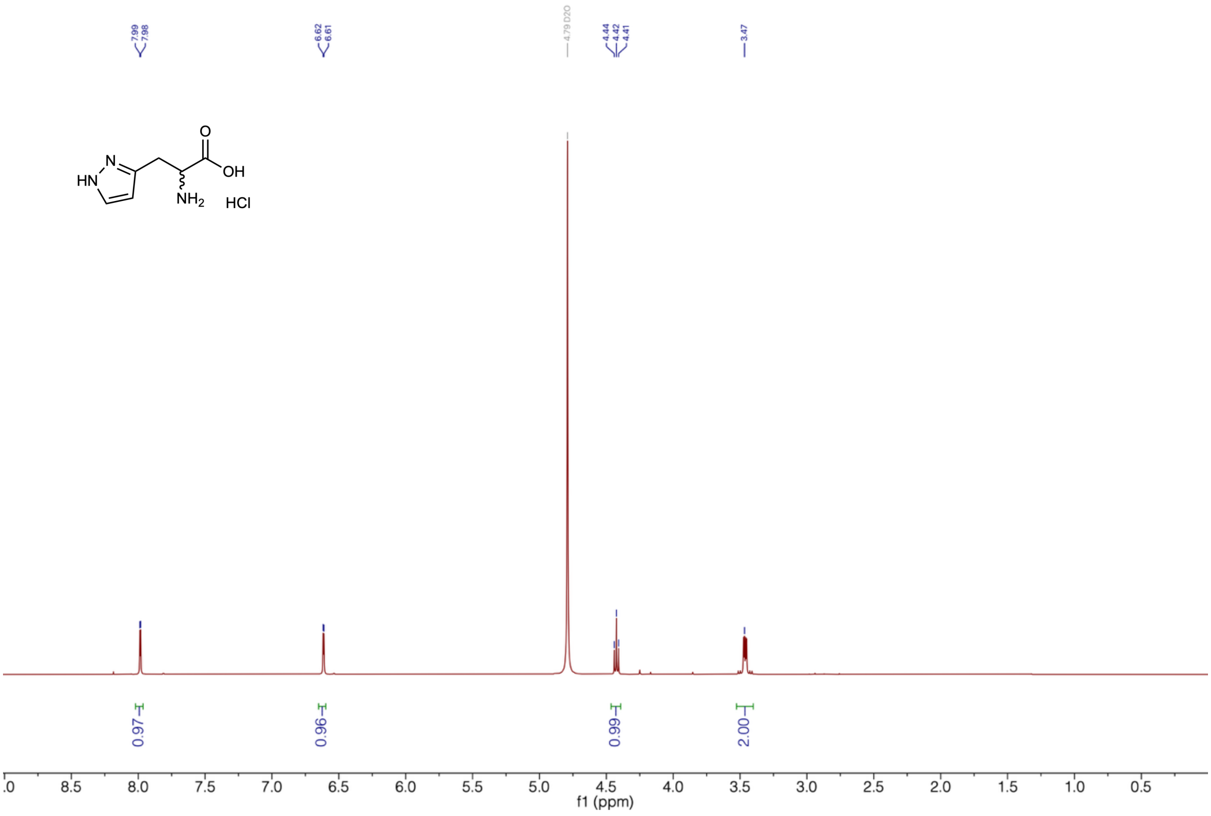

Supplementary Figure 41 | ^{1}H-NMR of pyrazol-3-yl-alanine hydrochloride (3PzA).

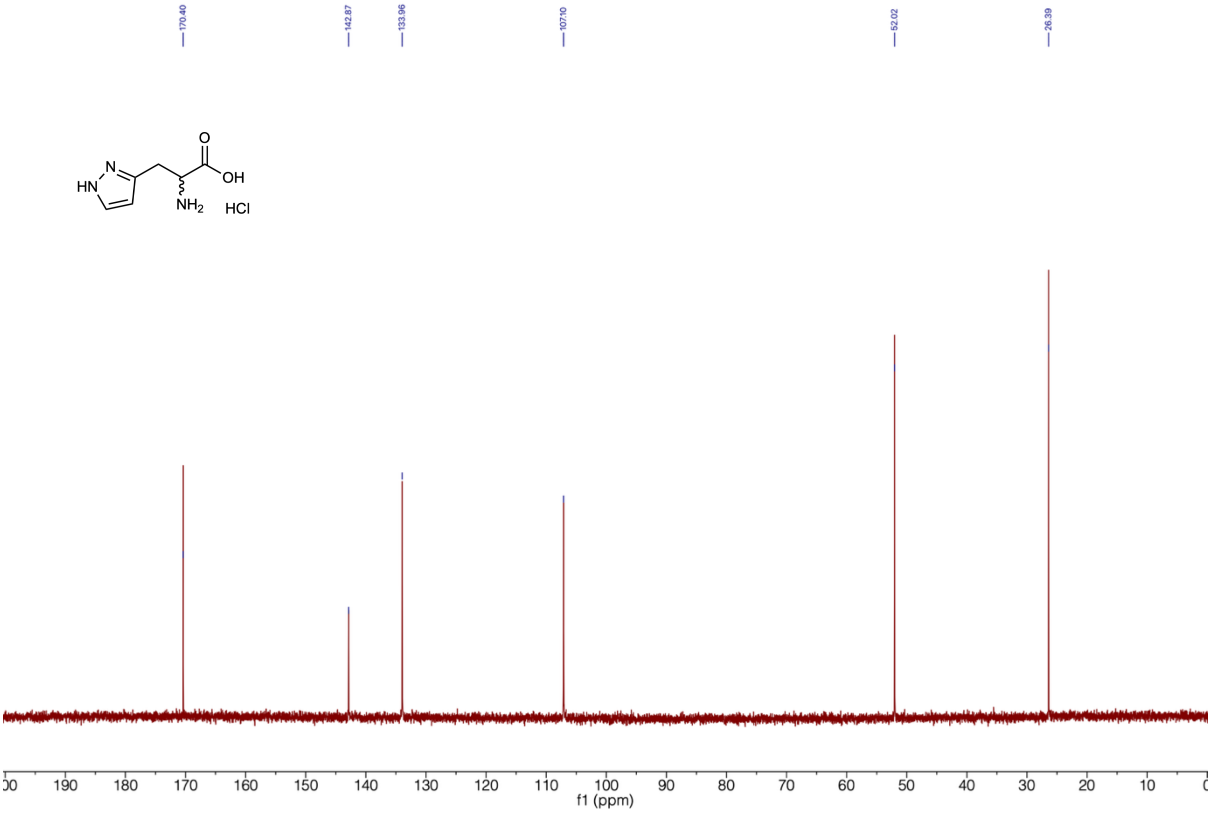

Supplementary Figure 42 | ^{13}C-NMR of pyrazol-3-yl-alanine hydrochloride (3PzA).

Oxazol-4-yl-L-alanine synthesis (4OxzA):

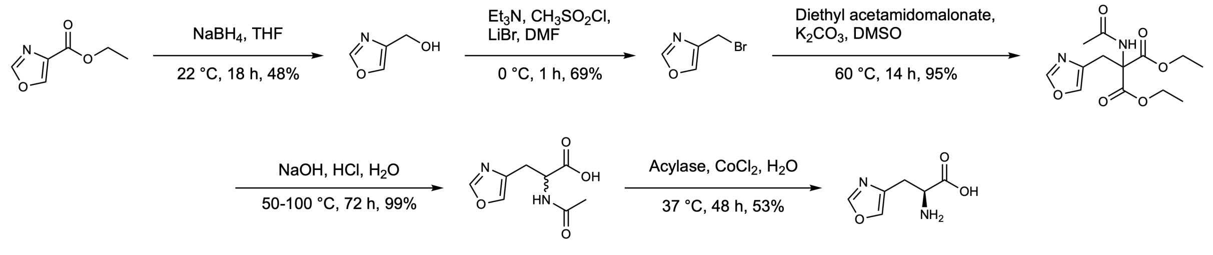

<u>(Oxazol-4-yl)methanol</u>

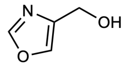

Oxazole-4-carboxylic acid ethyl ester (12.5 g, 88.6 mmol, 1.0 eq.) was dissolved in THF and water (9:1, 125 mL) and cooled to 0 °C. $NaBH_4$ (6.70 g, 177 mmol, 2.0 eq.) was added portion-wise, the suspension was allowed to reach room temperature and stirred for 18 h. The suspension was cooled to 0 °C and water (10 mL) was added. The mixture was stirred for 10 min, $MgSO_4$ was added, and stirred for another 10 min. The suspension was filtered through Celite and the filter cake was washed with a lot of EtOAc. The solvent was removed *in vacuo*. The crude product was purified by automated flash column chromatography (SiO_2, 0 – 10% MeOH in EtOAc) to yield (oxazol-4-yl)methanol (4.18 g, 42.1 mmol, 48%) as a colorless oil.

^{1}H-NMR (400 MHz, $CDCl_3$) δ 7.89 (s, 1H), 7.64 (s, 1H), 4.64 (s, 2H).

<u>(Oxazol-4-yl)methylbromide</u>

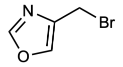

The following reaction was adapted from literature[12]. (Oxazol-4-yl)methanol (4.15 g, 41.9 mmol, 1.0 eq.) and Et_3N (7.6 mL, 55 mmol, 1.3 eq.) were dissolved in dry DMF (100 mL) under a nitrogen atmosphere. The solution was cooled to 0 °C and methanesulfonyl chloride (4.2 mL, 54 mmol, 1.3 eq.) was added. The yellow suspension was stirred for 10 min and LiBr (8.37 g, 96.3 mmol, 2.3 eq.) was added. The suspension was allowed to reach room temperature and stirred for 1 h. The reaction mixture was quenched with water and extracted with EtOAc (3 x). The combined organic phase was washed with water, sat. aq. $NaHCO_3$, and brine. The organic phase was dried over Na_2SO_4 and the solvent was removed *in vacuo* to yield (oxazol-4-yl)methylbromide (4.69 g, 29.0 mmol, 69%) as a light-brown oil that still contained DMF and EtOAc.

^{1}H-NMR (400 MHz, $CDCl_3$) δ 7.88 (s, 1H), 7.69 (s, 1H), 4.53 (s, 2H).

Diethyl-2-acetamido-2-(oxazol-4-yl-methyl)malonate

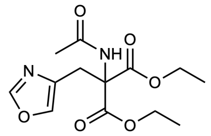

The following reaction was adapted from literature [9]. (Oxazol-4-yl)methylbromide (7.75 g, 28.7 mmol, 1.1 eq.) and diethyl-2-acetamidomalonate (5.94 g, 27.3 mmol, 1.0 eq.) were dissolved in DMSO (40 mL) and K_2CO_3 (5.67 g, 41.0 mmol, 1.5 eq.) was added. The suspension was stirred at 60 °C for 14 h and allowed to reach room temperature. Ice-cold water (200 mL) was added and the mixture was extracted with EtOAc (3 x 100 mL). The combined organic phase was washed with brine (2 x 100 mL) and dried over Na_2SO_4 to yield diethyl-2-acetamido-2-(oxazol-4-yl-methyl)malonate (7.78 g, 26.1 mmol, 95%) as a beige solid.

^{1}H-NMR (400 MHz, $CDCl_3$) δ 7.80 (s, 1H), 7.44 (s, 1H), 6.73 (s, 1H), 4.28 (q, $^3J_{HH}$ = 7.1 Hz, 4H), 3.62 (s, 2H), 2.00 (s, 3H), 1.28 (t, $^3J_{HH}$ = 7.1 Hz, 6H).

N-Acetyl-oxazol-4-yl-alanine

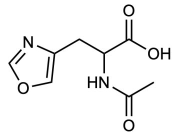

The following reaction was adapted from literature[13]. Diethyl-2-acetamido-2-(oxazol-4-yl-methyl)malonate (500 mg, 1.68 mmol, 1.0 eq.) was dissolved in water (7 mL) and NaOH (154 mg, 3.86 mmol, 2.3 eq.) was added. The solution was stirred at 50 °C for 26 h. The reaction mixture was adjusted to pH 4 with aq. HCl (6 M) and stirred at 100 °C for 39 h while adjusting the pH every few hours to pH 4. The solvent was removed *in vacuo* to yield *N*-acetyl-oxazol-4-yl-alanine (330 mg, 1.67 mmol, 99%) as a brown solid. The crude product was used for the next step without further purification.

^{1}H-NMR (400 MHz, D_2O) δ 8.12 (s, 1H), 7.72 (s, 1H), 4.48 (dd, $^3J_{HH}$ = 8.7, 4.7 Hz, 1H), 3.09 (dd, $^3J_{HH}$ = 15.1 Hz, $^3J_{HH}$ = 4.7 Hz, 1H), 2.94 (dd, $^2J_{HH}$ = 15.1 Hz, $^3J_{HH}$ = 8.7 Hz, 1H), 2.00 (s, 3H).

Oxazol-4-yl-L-alanine (4OxzA)

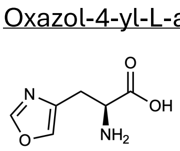

The following reaction was adapted from literature[13]. *N*-Acetyl-oxazol-4-yl-alanine (7.29 g, 36.8 mmol, 1.0 eq.) was dissolved in water (100 mL) and $CoCl_2$ (13 mg, 0.27 mol%) was added. The mixture was adjusted to pH 7-8 and acylase from *Aspergillus genus* (75 mg) was added. The reaction mixture was stirred at 37 °C for 48 h and the solvent was removed *in vacuo* to yield an orange glue. The crude product was purified by preparative HPLC chromatography (HILIC, 50-90% water in acetonitrile) to yield oxazol-4-yl-L-alanine (1.51 g, 9.67 mmol, 53%) as an off-white solid.

^{1}H-NMR (400 MHz, D_2O) δ 8.15 (s, 1H), 7.80 (s, 1H), 4.03 (dd, $^3J_{HH}$ = 7.6, 4.9 Hz, 1H), 3.25 – 3.06 (m, 2H).

^{13}C-NMR (101 MHz, D_2O) δ 173.3, 152.9, 137.3, 133.4, 54.0, 26.6.

MS (ESI): calc. for $[M+H]^+$: 157.05, obs.: 157.1.

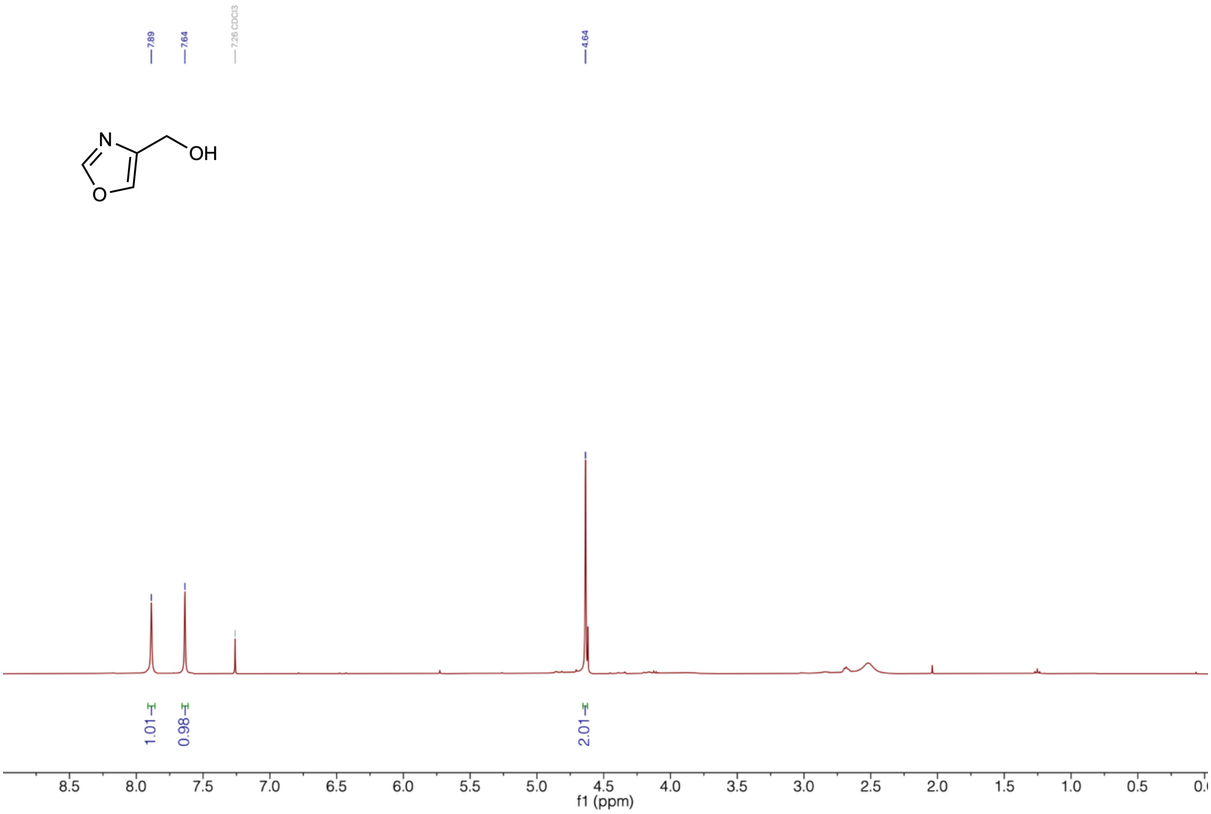

Supplementary Figure 43 | ^{1}H-NMR of (oxazol-4-yl)methanol.

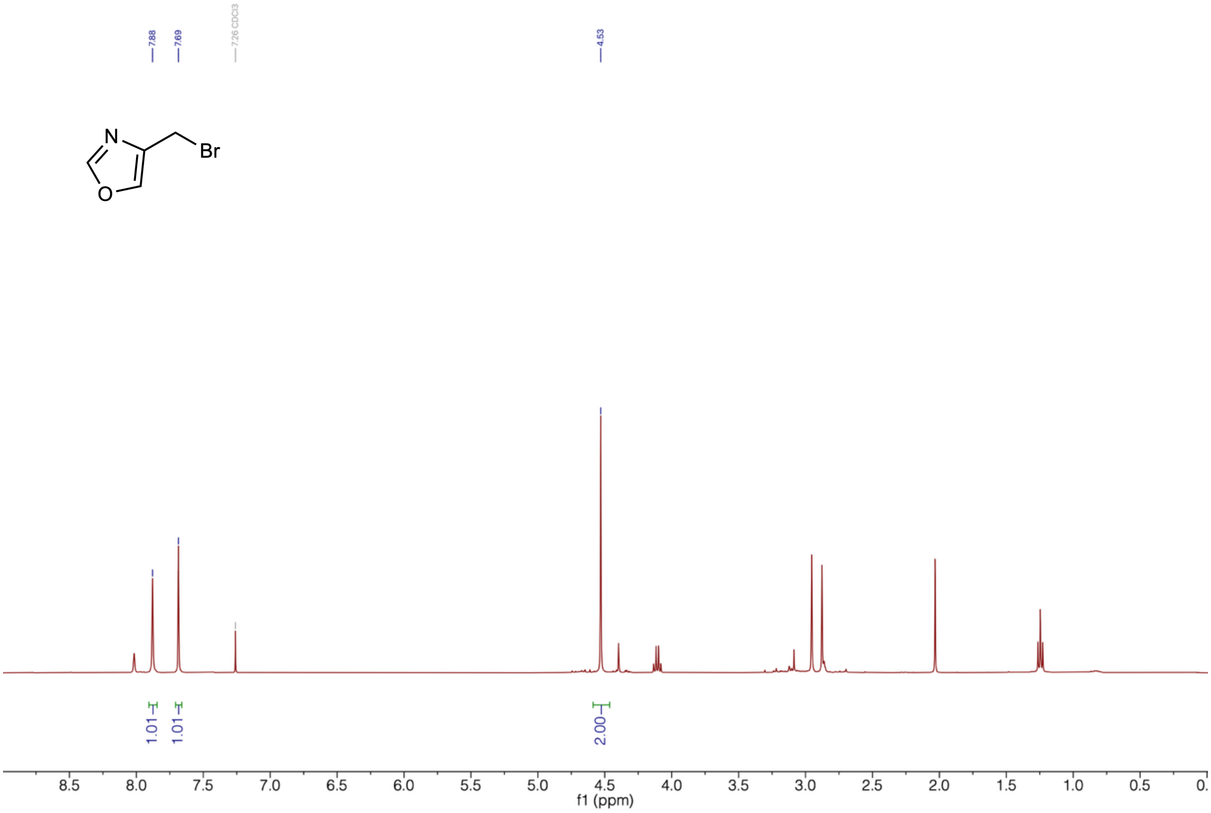

Supplementary Figure 44 | ^{1}H-NMR of (oxazol-4-yl)methylbromide.

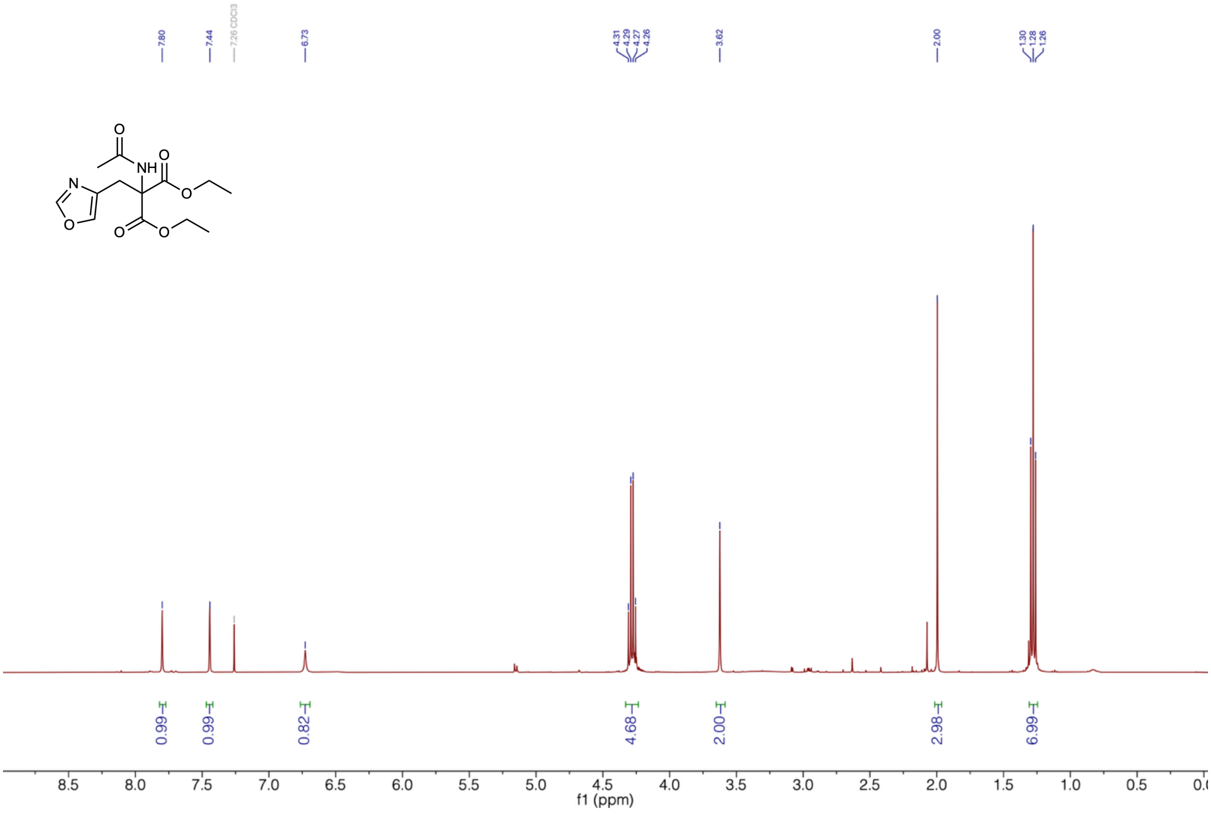

Supplementary Figure 45 | ^{1}H-NMR of diethyl-2-acetamido-2-(oxazol-4-yl-methyl)malonate

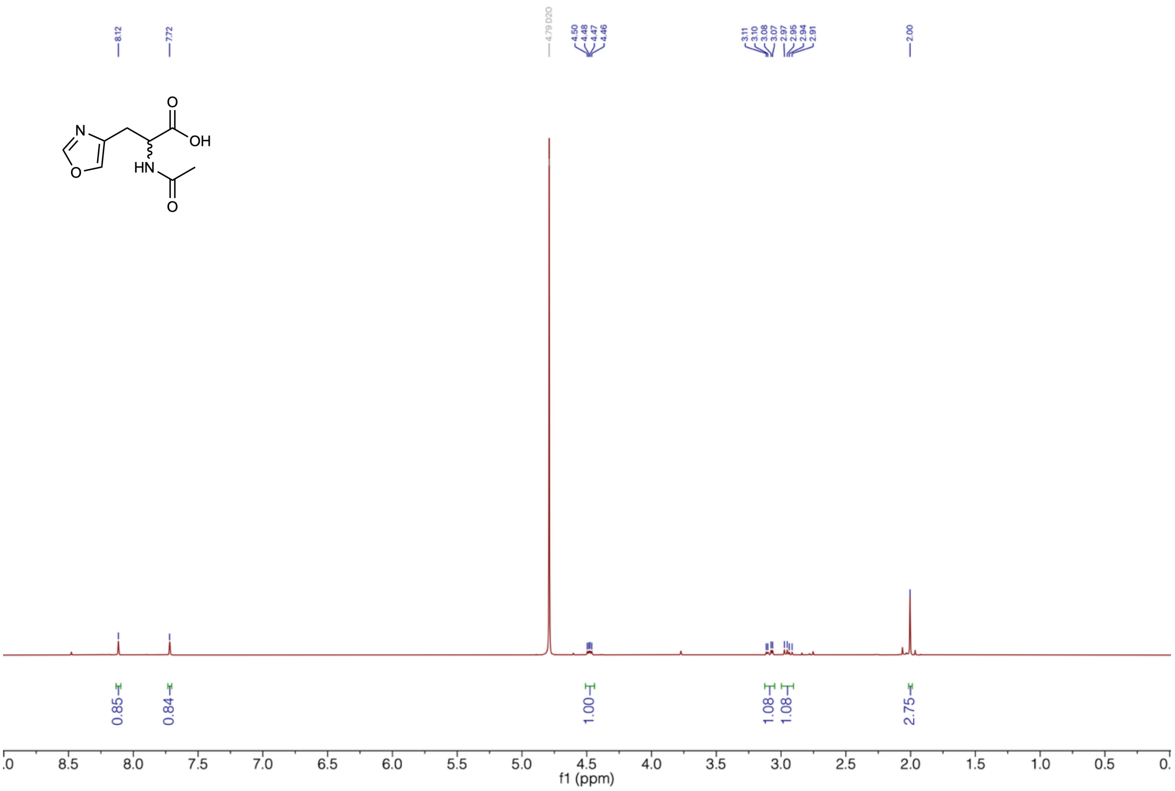

Supplementary Figure 46 | ^{1}H-NMR of *N*-acetyl-oxazol-4-yl-alanine.

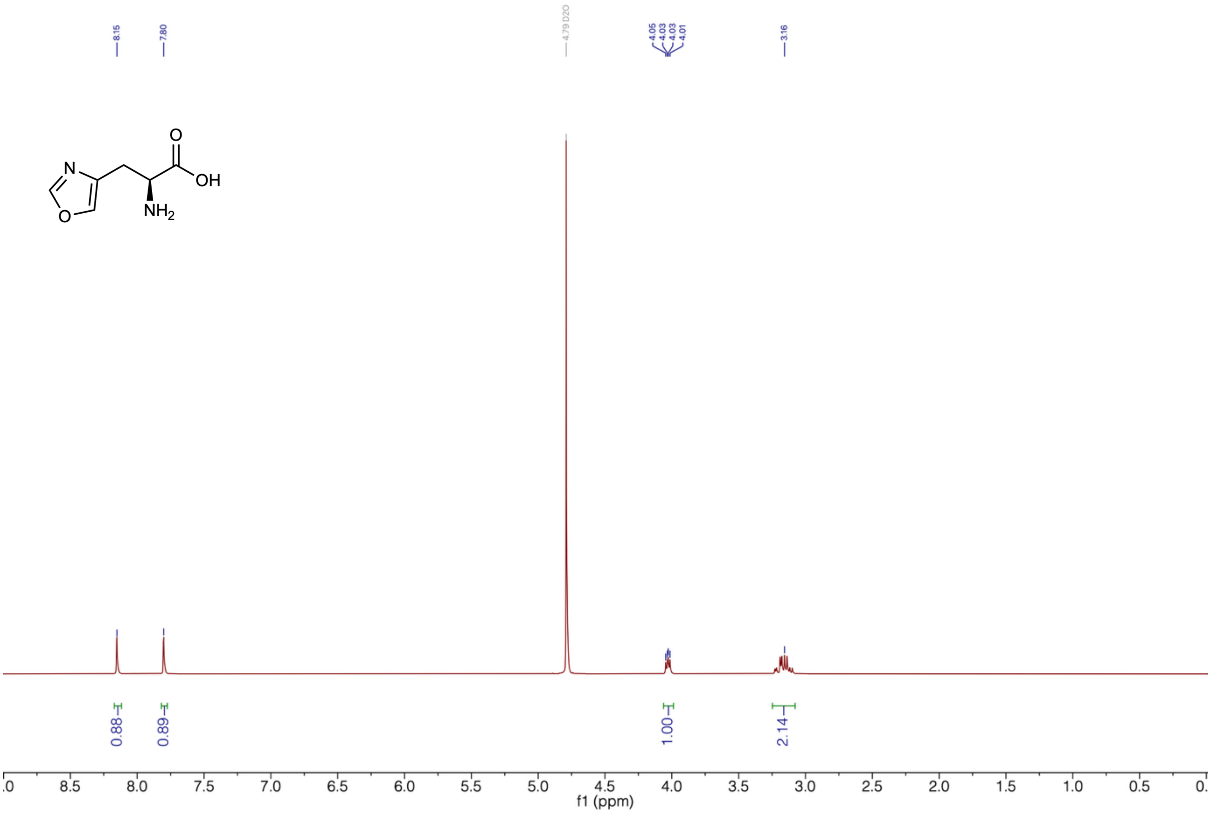

Supplementary Figure 47 | ^{1}H-NMR of oxazol-4-yl-L-alanine (4OxzA).

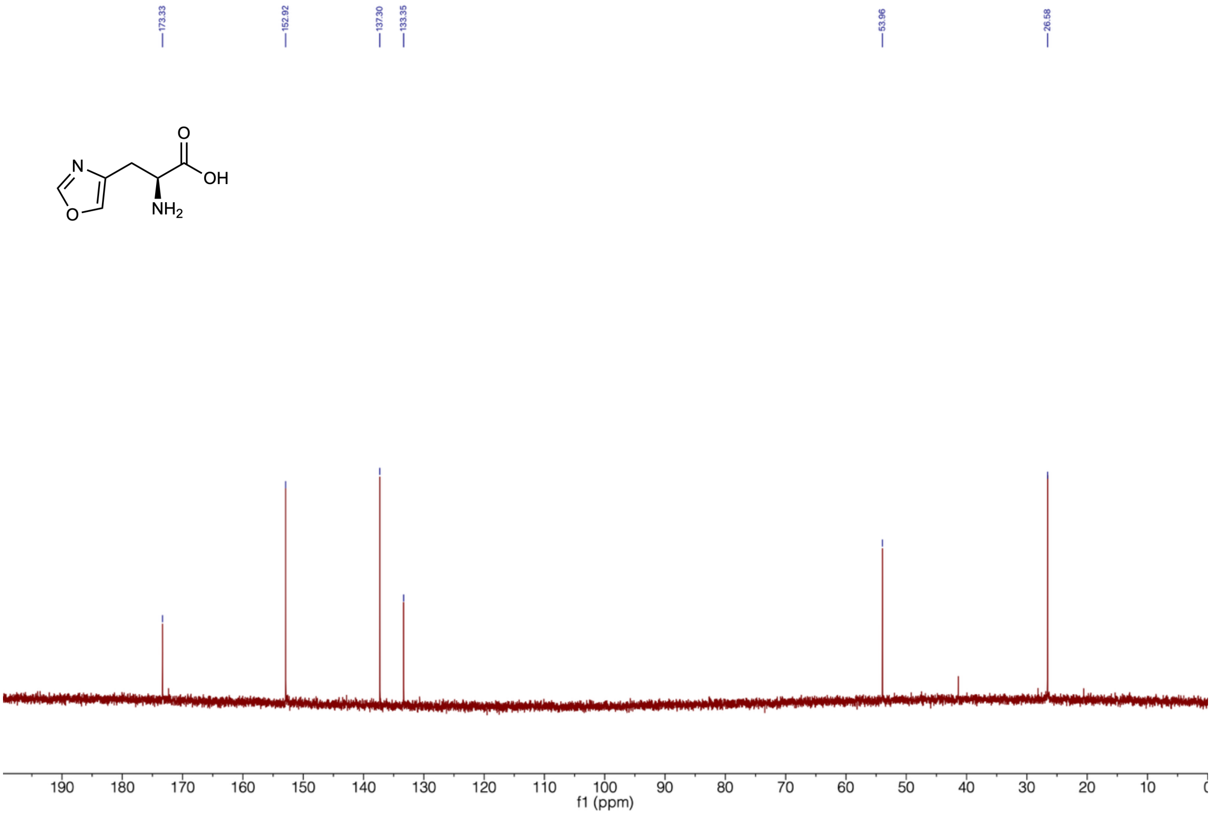

Supplementary Figure 48 | ^{13}C-NMR of oxazol-4-yl-L-alanine (4OxzA).

<u>2-Ethyl-5-methyl-histidine hydrochloride synthesis (2E5MH):</u>

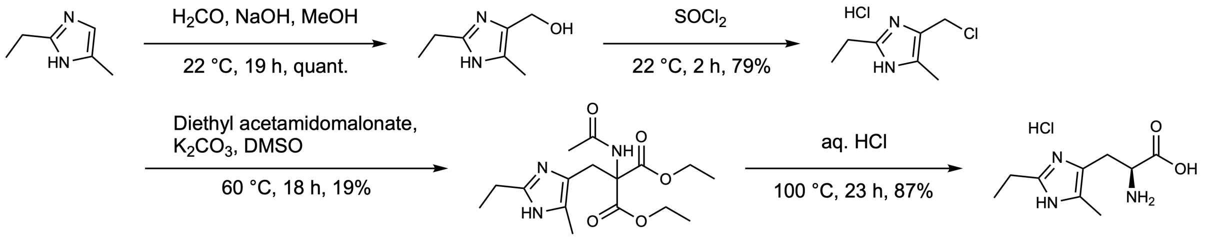

<u>2-Ethyl-4-hydroxymethyl-5-methylimidazole</u>

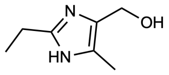

2-Ethyl-4-methylimidazole (5.00 g, 45.4 mmol, 1.0 eq.) was dissolved in 100 mL EtOH. Aq. NaOH (2.5 M, 16 mL) and paraformaldehyde (37% aq., 116 mmol, 2.5 eq.) was added. The mixture was stirred at room temperature for 19 h. The reaction was cooled to 0 °C and the pH was adjusted to pH 7 using aq. HCl (4 M). The solvent was removed *in vacuo* and the crude product was purified by automated flash column chromatography (SiO_2, 20 – 80% MeOH in EtOAc) to yield 2-ethyl-4-hydroxymethyl-5-methylimidazole (6.36 g, 45.4 mmol, quant.) as a yellow glue.

^{1}H-NMR (400 MHz, D_2O) δ 4.52 (s, 2H), 2.76 (q, $^{3}J_{HH}$ = 7.7 Hz, 2H), 2.19 (s, 3H), 1.25 (t, $^{3}J_{HH}$ = 7.7 Hz, 3H).

4-Chloromethyl-2-ethyl-5-methylimidazole hydrochloride

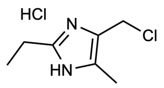

2-ethyl-4-hydroxymethyl-5-methylimidazole (5.20 g, 37.1 mmol, 1.0 eq.) was dissolved in thionyl chloride (13.6 mL, 185 mmol, 5.0 eq.) and the reaction mixture was stirred at room temperature for 2 h. Chloroform was added and the solvent was removed *in vacuo*. Chloroform was added and the solvent was removed two more times to yield 4-chloromethyl-2-ethyl-5-methylimidazole hydrochloride (5.69 g, 29.2 mmol, 79%) as a beige solid.

^{1}H-NMR (400 MHz, DMSO-d$_6$) δ 4.86 (s, 2H), 2.88 (q, $^3J_{HH}$ = 7.6 Hz, 2H), 2.27 (s, 3H), 1.29 (t, $^3J_{HH}$ = 7.6 Hz, 3H).

Diethyl-2-acetamido-2-(2-ethyl-4-methyl-imidaz-5-yl-methyl)malonate

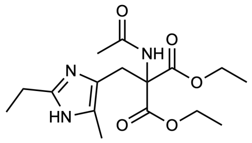

The following reaction was adapted from literature[9]. 4-Chloromethyl-2-ethyl-5-methyl-imidazole hydrochloride (5.69 g, 29.2 mmol, 1.1 eq.) was dissolved in DMSO (40 mL) and diethyl-2-acetamidomalonate (5.76 g, 26.5 mmol, 1.0 eq.) and K_2CO_3 (9.16 g, 66.3 mmol, 2.5 eq.) were added. The suspension was stirred at 60 °C for 18 h and the reaction mixture was allowed to reach room temperature. Ice-cold water (200 mL) was added and the solution was adjusted to pH 9-10 with aq. NaOH. The solution was extracted with EtOAc (4 x 100 mL) and the combined organic phase was washed with brine (200 mL). The organic phase was dried over Na_2SO_4 and the solvent was removed *in vacuo*. The crude product was purified by automated flash column chromatography (SiO_2, 5 – 15% MeOH in EtOAc) to yield diethyl-2-acetamido-2-(2-ethyl-4-methyl-imidaz-5-yl-methyl)malonate (1.75 g, 5.16 mmol, 19%) as a yellow glue. The product was directly used for the next step.

2-Ethyl-5-methyl-histidine hydrochloride (2E5MH)

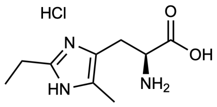

Diethyl-2-acetamido-2-(2-ethyl-4-methyl-imidaz-5-yl-methyl)malonate (1.75 g, 5.16 mmol, 1.0 eq.) was suspended in aq. HCl (4 M, 35 mL) and stirred at 100 °C for 23 h. The solvent was removed *in vacuo* and the crude product was purified by automated flash column chromatography (C18, 1% acetonitrile in water) to yield 2-ethyl-5-methyl-histidine hydrochloride (1.05 g, 4.49 mmol, 87%) as a white solid.

^{1}H-NMR (400 MHz, D_2O) δ 4.22 (t, $^3J_{HH}$ = 7.1 Hz, 1H), 3.37 – 3.21 (m, 1H), 2.91 (q, $^3J_{HH}$ = 7.6 Hz, 2H), 2.23 (s, 3H), 1.32 (t, $^3J_{HH}$ = 7.6 Hz, 3H).

^{13}C-NMR (101 MHz, D_2O) δ 170.8, 148.2, 127.2, 120.4, 52.3, 24.4, 18.9, 10.3, 7.9.

MS (ESI): calc. for $[M+H]^+$: 198.12, obs.: 198.2.

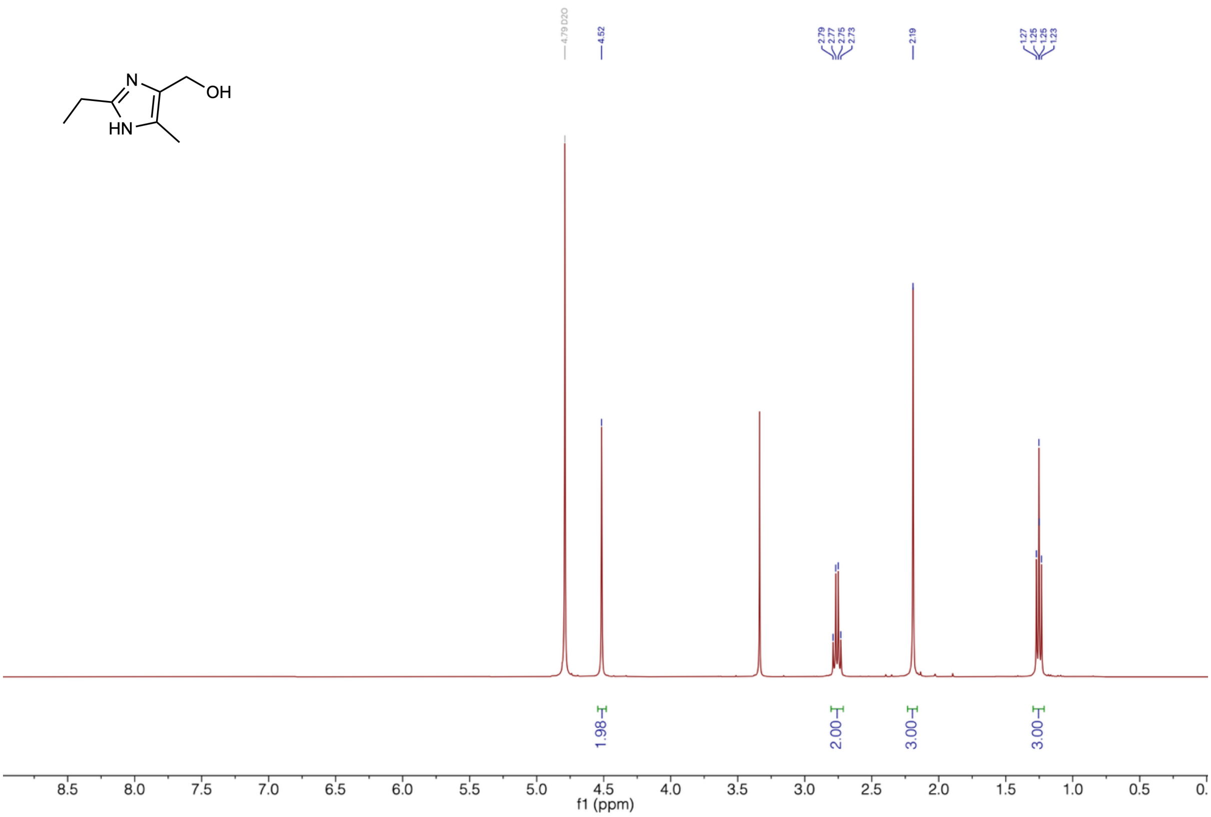

Supplementary Figure 49 | ^{1}H-NMR of 2-ethyl-4-hydroxymethyl-5-methylimidazole.

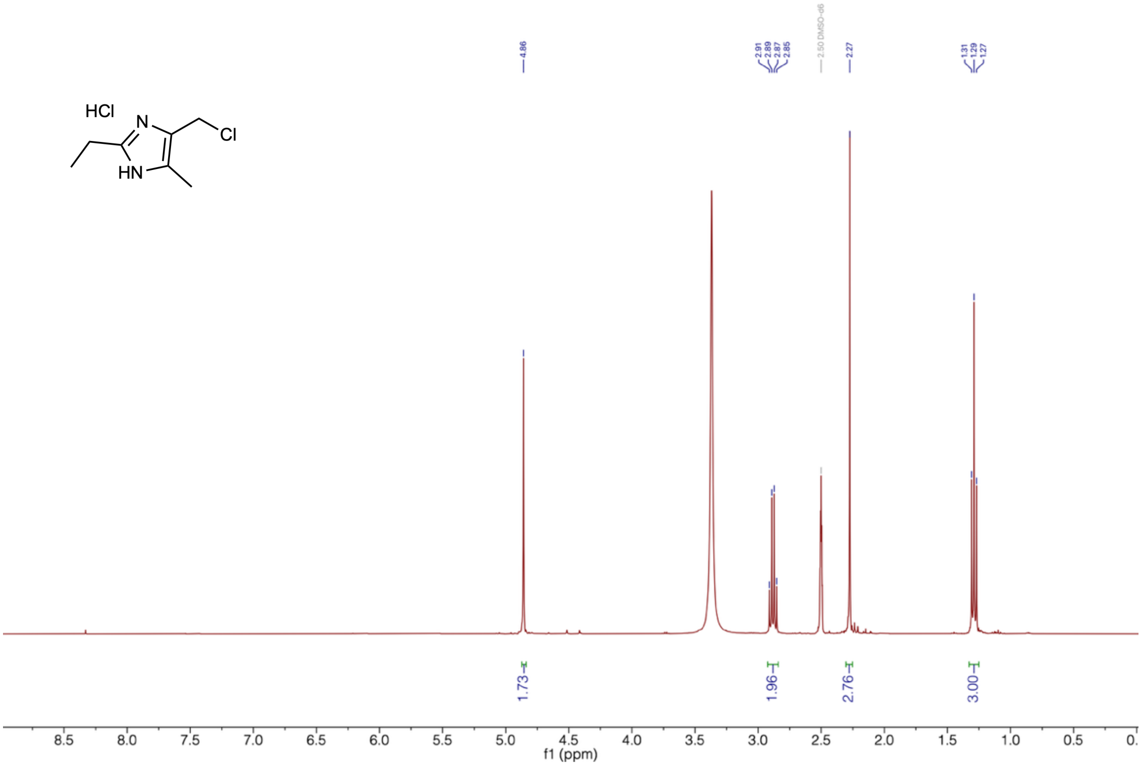

Supplementary Figure 50 | ^{1}H-NMR of 4-chloromethyl-2-ethyl-5-methylimidazole hydrochloride.

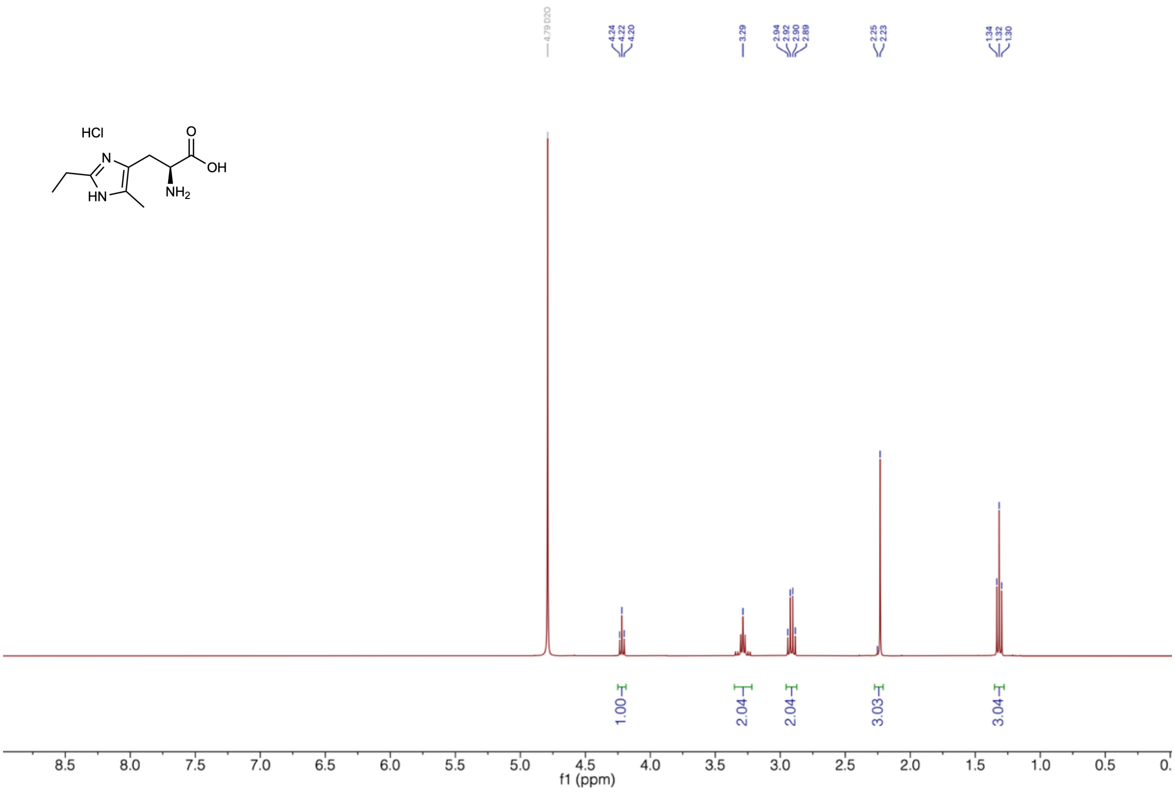

Supplementary Figure 51 | ^{1}H-NMR of 2-ethyl-5-methyl-histidine hydrochloride (2E5MH).

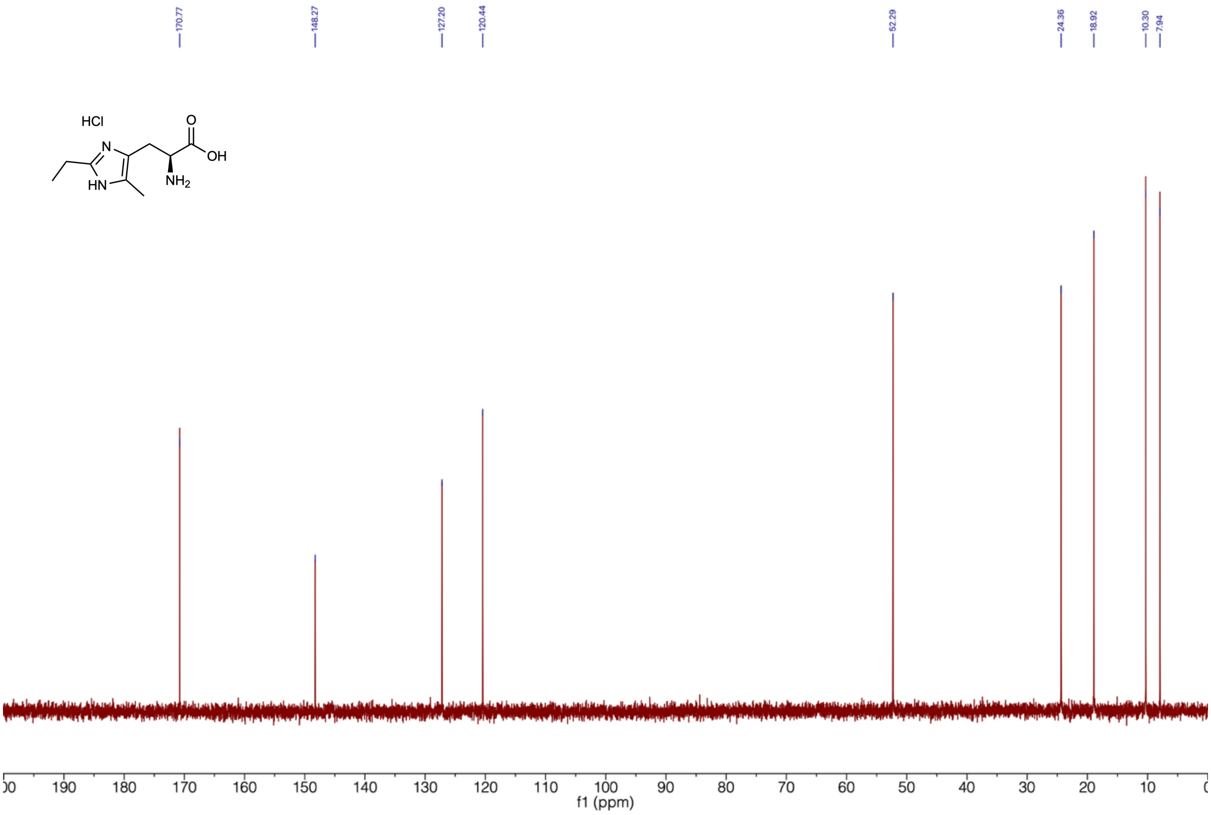

Supplementary Figure 52 | ^{13}C-NMR of 2-ethyl-5-methyl-histidine hydrochloride (2E5MH).

1,2,4-Triazol-1-yl-L-alanine synthesis (124Trz-1A):

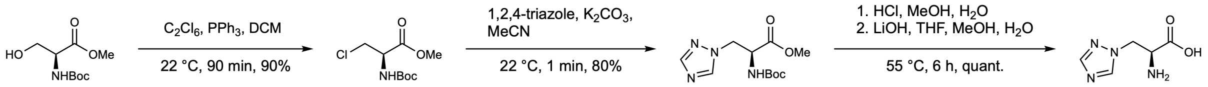

<u>*N*-Boc-chloro-L-alanine-OMe</u>

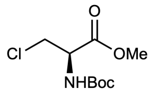

The following reaction was adapted from literature[14]. *N*-Boc-L-serine-OMe (2.00 g, 8.67 mmol, 1.0 eq.) was dissolved in dry dichloromethane (32 mL) under a nitrogen atmosphere. Triphenylphosphine (2.55 g. 9.71 mmol, 1.1 eq.) in dry dichloromethane (4 mL) and hexachloroethane (2.30 g, 9.62 mmol, 1.1 eq.) in dry dichloromethane (4 mL) were added in one portion and the reaction was stirred at room temperature for 90 min. The reaction was quenched with aq. sat. $NaHCO_3$ (20 mL) and the phases were separated. The aqueous phase was extracted with dichloromethane, the combined organic phase was washed with brine (2 x), and dried over Na_2SO_4. The solvent was removed *in vacuo* and the crude product was purified by flash column chromatography (SiO_2, dichloromethane) to yield *N*-boc-chloro-L-alanine-OMe (1.85 g, 7.78 mmol, 90%) as a white solid.

^{1}H-NMR (400 MHz, $CDCl_3$) δ 5.42 (d, $^3J_{HH}$ = 8.1 Hz, 1H), 4.76 – 4.67 (m, 1H), 3.97 (dd, $^2J_{HH}$ = 11.2 Hz, $^3J_{HH}$ = 3.1 Hz, 1H), 3.85 (dd, $^2J_{HH}$ = 11.2 Hz, $^3J_{HH}$ = 3.5 Hz, 1H), 3.81 (s, 3H), 1.46 (s, 9H).

<u>*N*-Boc-1,2,4-triazol-1-yl-L-alanine-OMe</u>

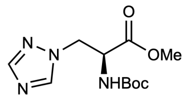

1,2,4-Triazole (1.56 g, 22.6 mmol, 3.1 eq.) was dissolved in acetonitrile and K_2CO_3 (1.91 g, 13.8 mmol, 1.9 eq.) was added. The suspension was stirred at room temperature for 15 min and *N*-boc-chloro-L-alanine-OMe (1.75 g, 7.36 mmol, 1.0 eq.) in acetonitrile (17 mL) was added dropwise. The suspension was stirred at room temperature for 1 min and filtered through Celite. The solvent was removed *in vacuo* and the crude product was purified by automated flash column chromatography (5 – 20% MeOH in DCM) to yield *N*-boc-1,2,4-triazol-1-yl-L-alanine-OMe (1.60 g, 5.92 mmol, 80%) as a colorless oil.

^{1}H-NMR (400 MHz, $CDCl_3$) δ 8.05 (s, 1H), 7.93 (s, 1H), 5.39 (s, 1H), 4.72 – 4.59 (m, 3H), 3.79 (s, 3H), 1.44 (s, 9H).

<u>1,2,4-Triazol-1-yl-L-alanine (124Trz-1A)</u>

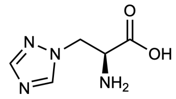

N-Boc-1,2,4-triazol-1-yl-L-alanine-OMe (1.60 g, 5.92 mmol, 1.0 eq.) was dissolved in MeOH and conc. aq. HCl (2.5 mL, 30 mmol, 5.0 eq.) was added. The solution was stirred at room temperature for 1 h, the solvent was removed *in vacuo* and the solid was dissolved in a mixture of THF, MeOH, and water

(9 mL each). LiOH monohydrate (621 mg, 14.8 mmol, 2.5 eq.) was added and the solution was stirred at 55 °C for 6 h. The solvent was removed *in vacuo to* yield 1,2,4-triazol-1-yl-L-alanine (924 mg, 5.92 mmol, quant.) as a white solid.

^{1}H-NMR (400 MHz, D_2O) δ 8.39 (s, 1H), 8.04 (s, 1H), 4.47 (dd, $^{2}J_{HH}$ = 14.2 Hz, $^{3}J_{HH}$ = 5.0 Hz, 1H), 4.41 (dd, $^{2}J_{HH}$ = 14.2 Hz, $^{3}J_{HH}$ = 6.8 Hz, 1H), 3.72 (dd, $^{3}J_{HH}$ = 6.8, 5.0 Hz, 1H).

^{13}C-NMR (101 MHz, D_2O) δ 168.7, 147.9, 144.0, 52.3, 49.1.

MS (ESI): calc. for $[M+H]^{+}$: 157.07, obs.: 157.1.

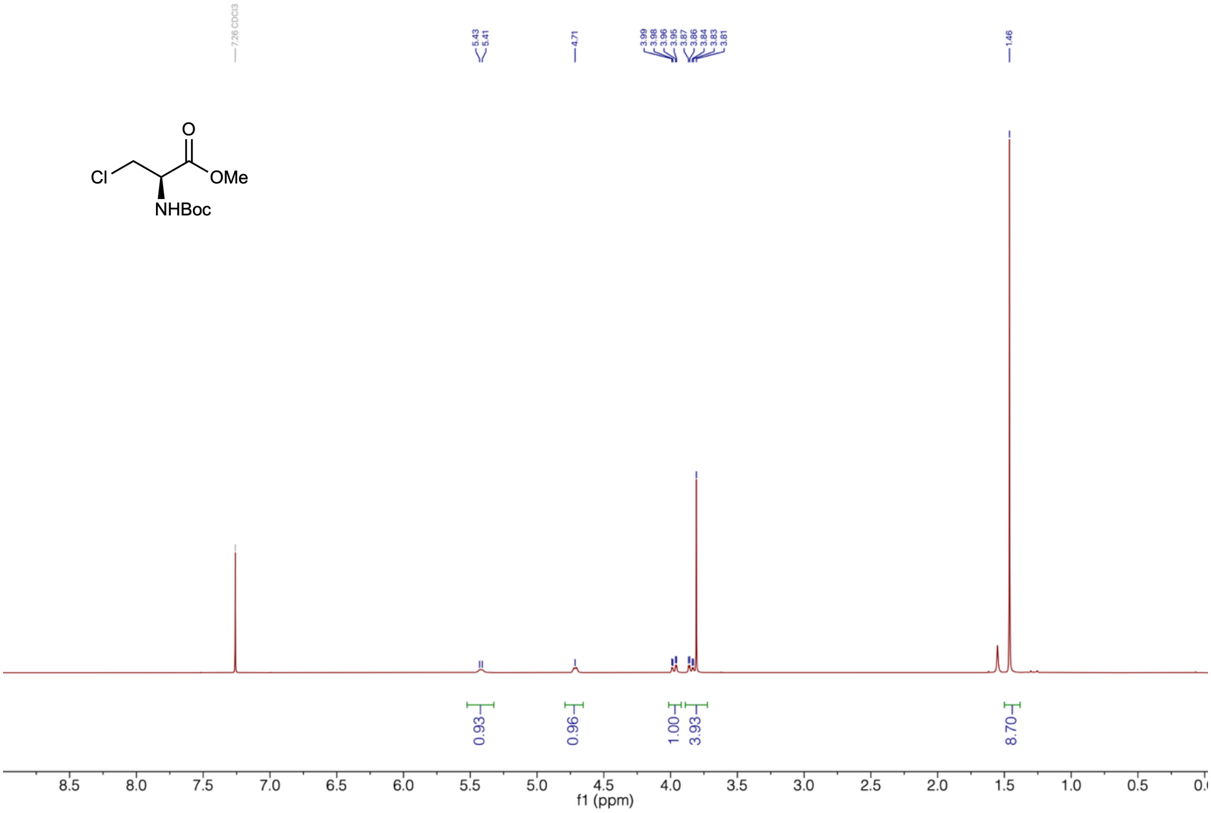

Supplementary Figure 53 | ^{1}H-NMR of *N*-Boc-chloro-L-alanine-OMe.

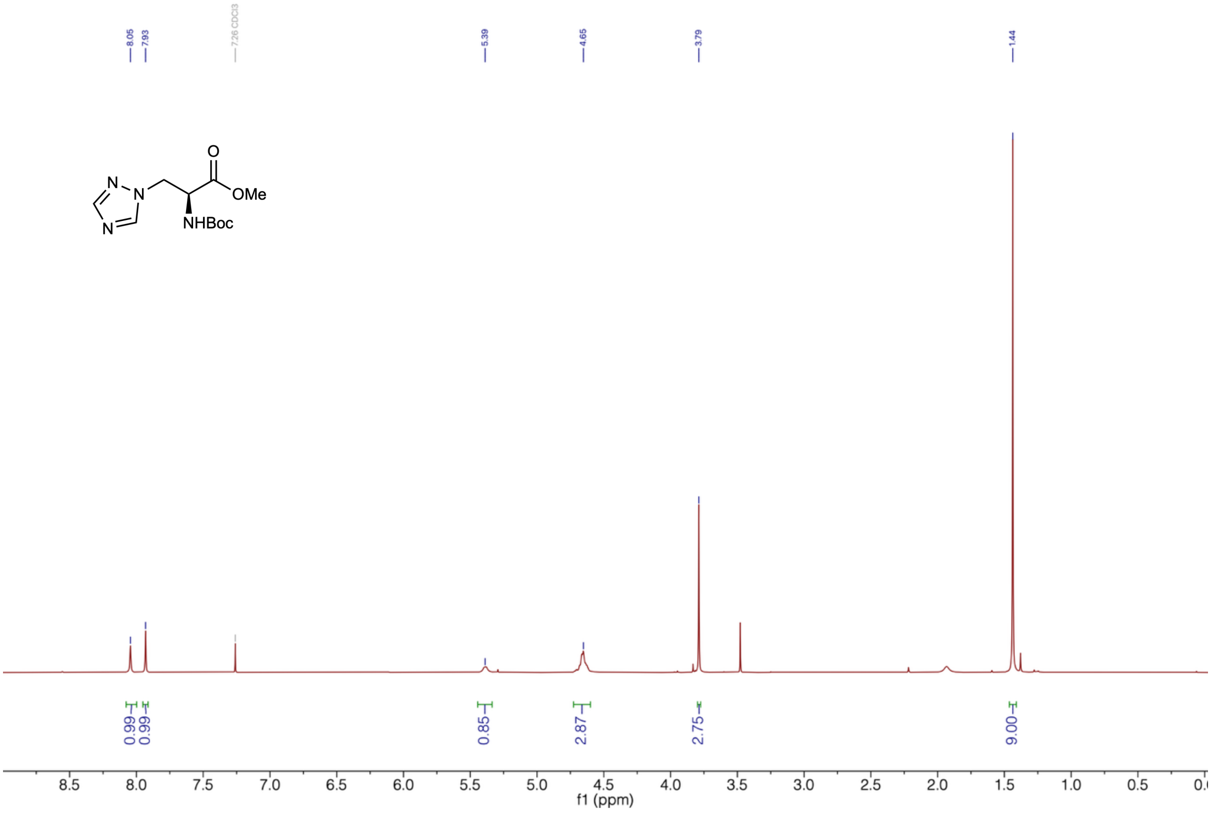

Supplementary Figure 54 | ^{1}H-NMR of *N*-Boc-1,2,4-triazol-1-yl-L-alanine-OMe.

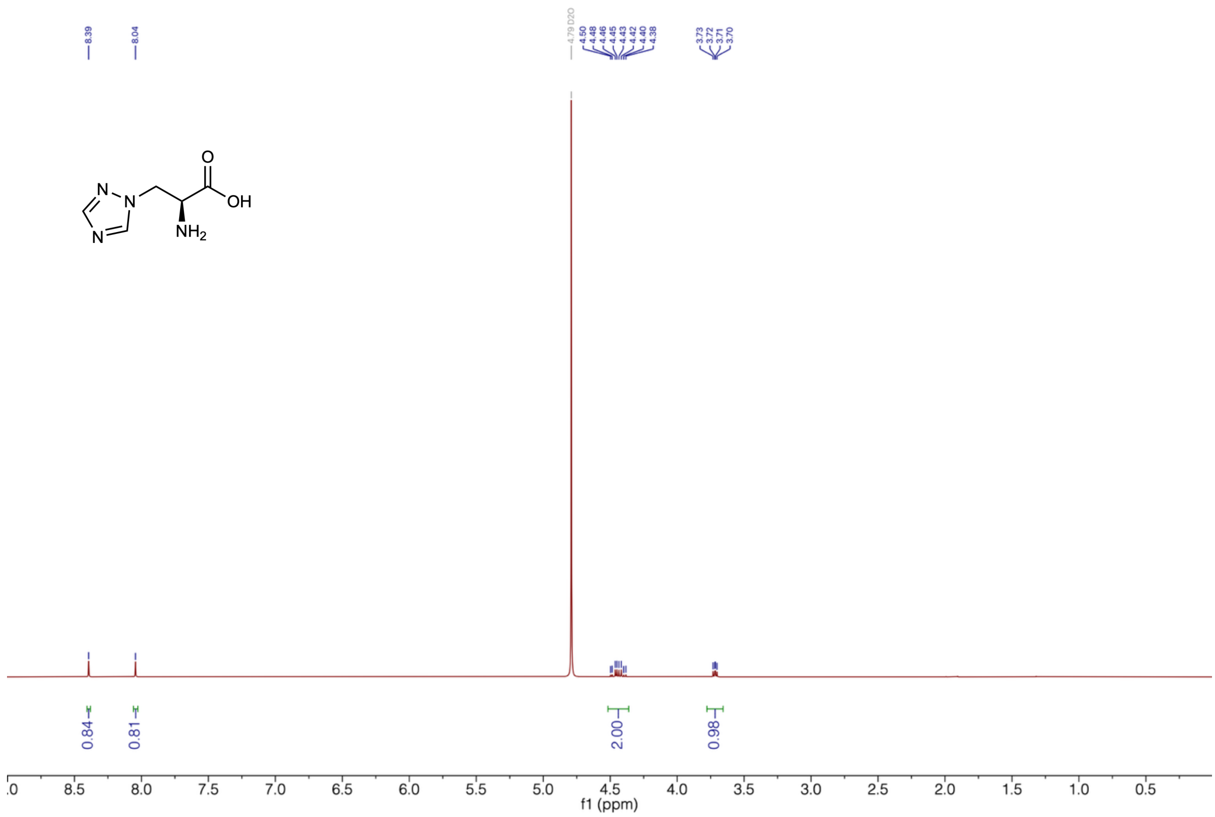

Supplementary Figure 55 | ^{1}H-NMR of 1,2,4-triazol-1-yl-L-alanine (124Trz-1A).

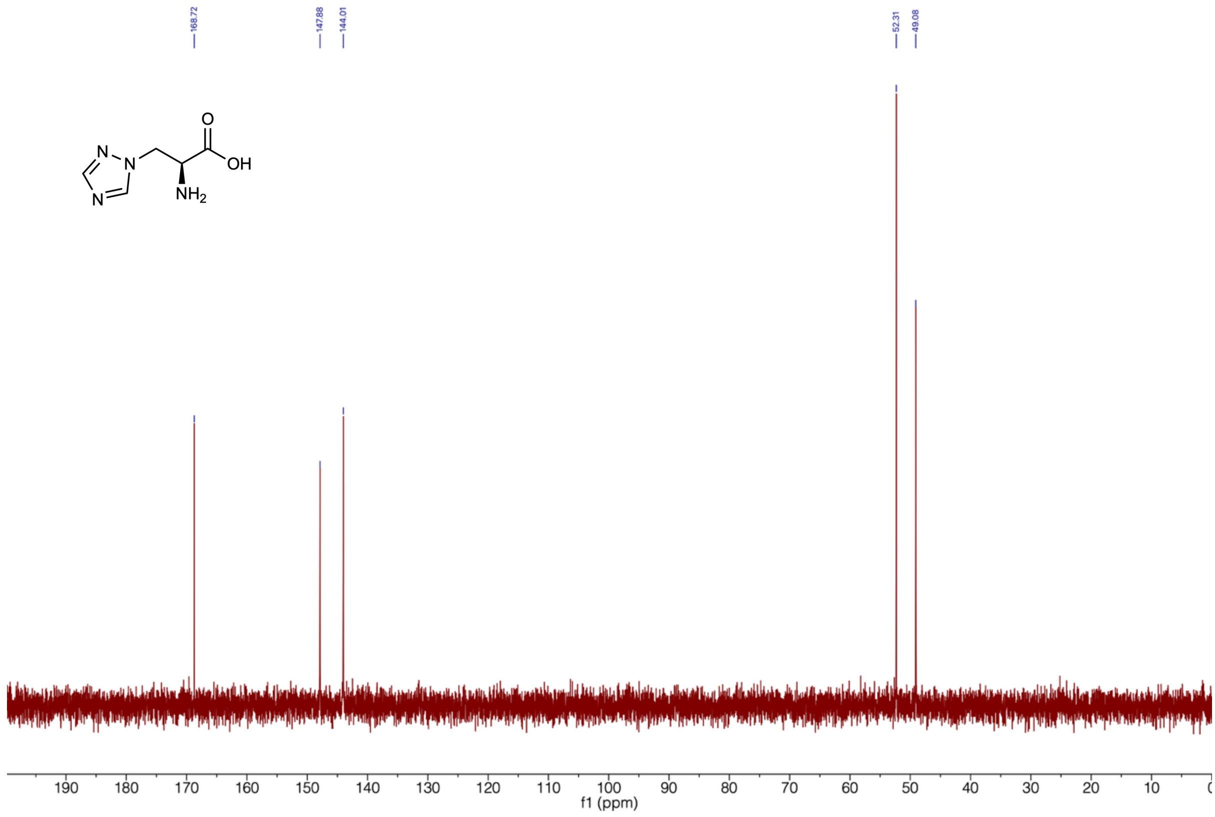

Supplementary Figure 56 | ^{13}C-NMR of 1,2,4-triazol-1-yl-L-alanine (124Trz-1A).

N^{τ}-methyl-L-histidine synthesis (τMH)

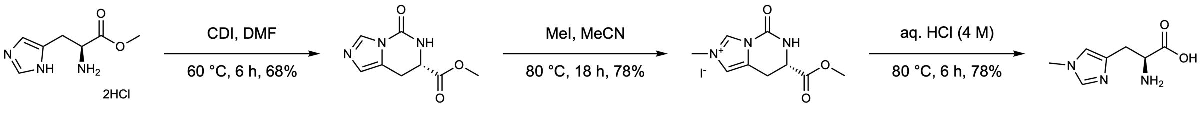

Methyl-(*S*)-5-oxo-5,6,7,8-tetrahydroimidazo[1,5-*c*]pyrimidine-7-carboxylate

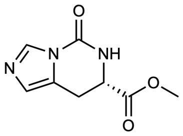

The procedure was adapted from literature[15]. L-histidine-OMe dihydrochloride (10.0 g, 41.3 mmol, 1.0 eq.) and *N*,*N'*-carbonyldiimidazole (7.37 g, 45.4 mmol, 1.1 eq.) were dissolved in DMF (200 mL). The reaction mixture was stirred at 60 ºC for 6 h. The solvent was removed *in vacuo* and the crude product was purified by automated flash column chromatography (SiO_2, 5% MeOH in DCM) to yield methyl-(*S*)-5-oxo-5,6,7,8-tetrahydroimidazo[1,5-c]pyrimidine-7-carboxylate (5.50 g, 28.2 mmol, 68%) as an off-white solid.

^{1}H-NMR (400 MHz, DMSO-d_6) δ 8.56-8.55 (m, 1H), 8.10 (s, 1H), 6.82 (s, 1H), 4.46-4.42 (m, 1H), 3.62 (s, 3H), 3.23-3.21 (m, 2H).

(*S*)-7-(methoxycarbonyl)-2-methyl-5-oxo-5,6,7,8-tetrahydroimidazo[1,5-*c*]pyrimidin-2-ium iodide

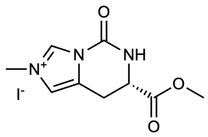

Methyl iodide (4.3 mL, 69 mmol, 6.7 eq.) was added to methyl (*S*)-5-oxo-5,6,7,8-tetrahydroimidazo[1,5-*c*]pyrimidine-7-carboxylate (2.00 g, 10.3 mmol, 1.0 eq.) in acetonitrile (60 mL). The reaction was stirred at 80 °C for 18 h. The solvent was removed *in vacuo* and the crude residue was recrystallized from 20% MeOH in DCM to yield (*S*)-7-(methoxycarbonyl)-2-methyl-5-oxo-5,6,7,8-tetrahydroimidazo[1,5-c]pyrimidin-2-ium iodide (2.70 g, 8.01 mmol, 78%) as an off-white solid.

^{1}H-NMR (400 MHz, D_2O) δ 9.38 (s, 1H), 7.42 (s, 1H), 4.74 (t, $^{3}J_{HH}$ = 5.6 Hz, 1H), 3.97 (s, 3H), 3.79 (s, 3H), 3.51-3.49 (m, 2H).

N^{τ}-methyl-L-histidine

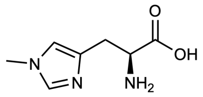

(*S*)-7-(methoxycarbonyl)-2-methyl-5-oxo-5,6,7,8-tetrahydroimidazo[1,5-c]pyrimidin-2-ium iodide (17.0 g, 50.4 mmol, 1.0 eq.) was suspended in aq. HCl (4 M, 500 mL) and stirred at 80 °C for 6 h. The solvent was removed *in vacuo* and the crude product was purified by automated flash column

chromatography (C18, 0-95% acetonitrile in water (0.1% formic acid)) to yield N^{τ}-methyl-L-histidine (6.40 g, 50.4 mmol, 75%) as an off-white solid.

^{1}H-NMR (400 MHz, D_2O) δ 8.64 (s, 1H), 7.38 (s, 1H), 4.19 (t, $^{3}J_{HH}$ = 6.6 Hz, 1H), 3.86 (s, 3H), 3.36-3.33 (m, 2H).

^{13}C-NMR (101 MHz, D_2O) δ 171.2, 135.5, 127.3, 121.9, 52.6, 35.6, 25.4.

MS (ESI): calc. for $[M+H]^{+}$: 170.09, obs.: 170.1.

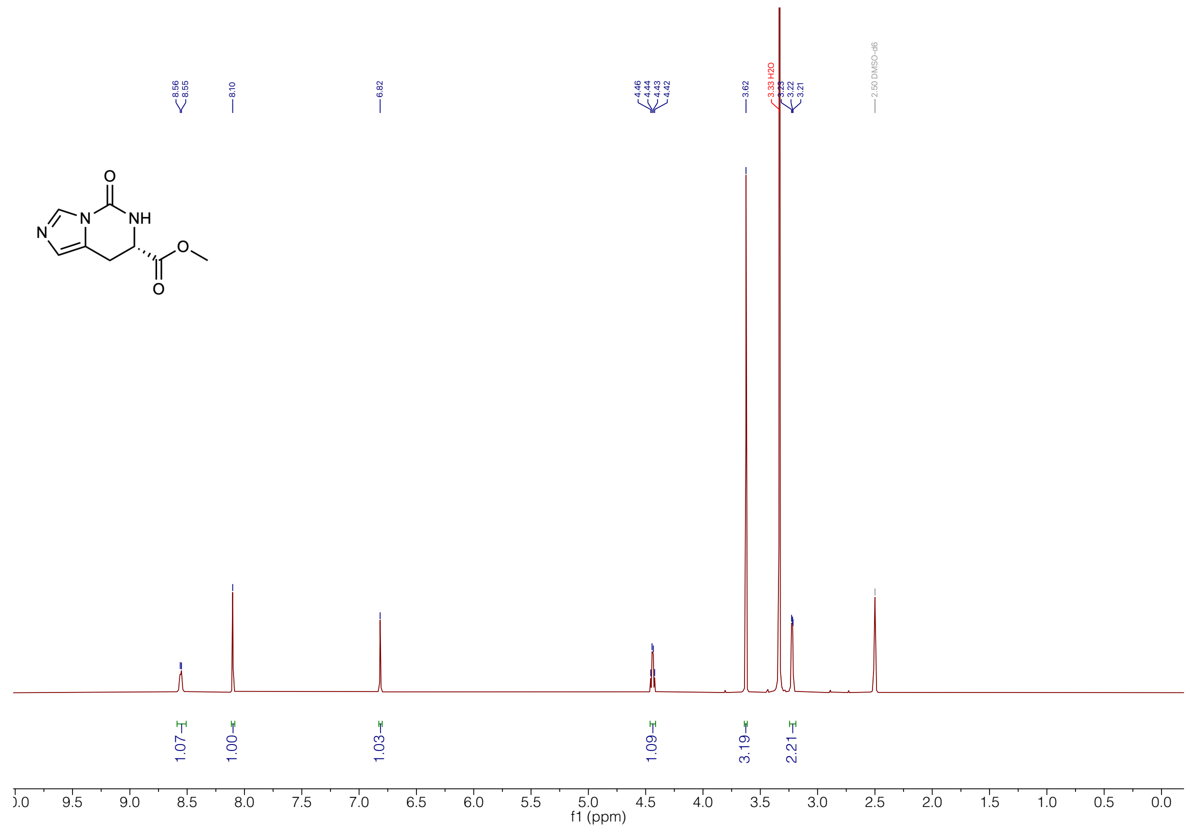

Supplementary Figure 57 | ¹H-NMR of methyl (*S*)-5-oxo-5,6,7,8-tetrahydroimidazo[1,5-*c*]pyrimidine-7-carboxylate.

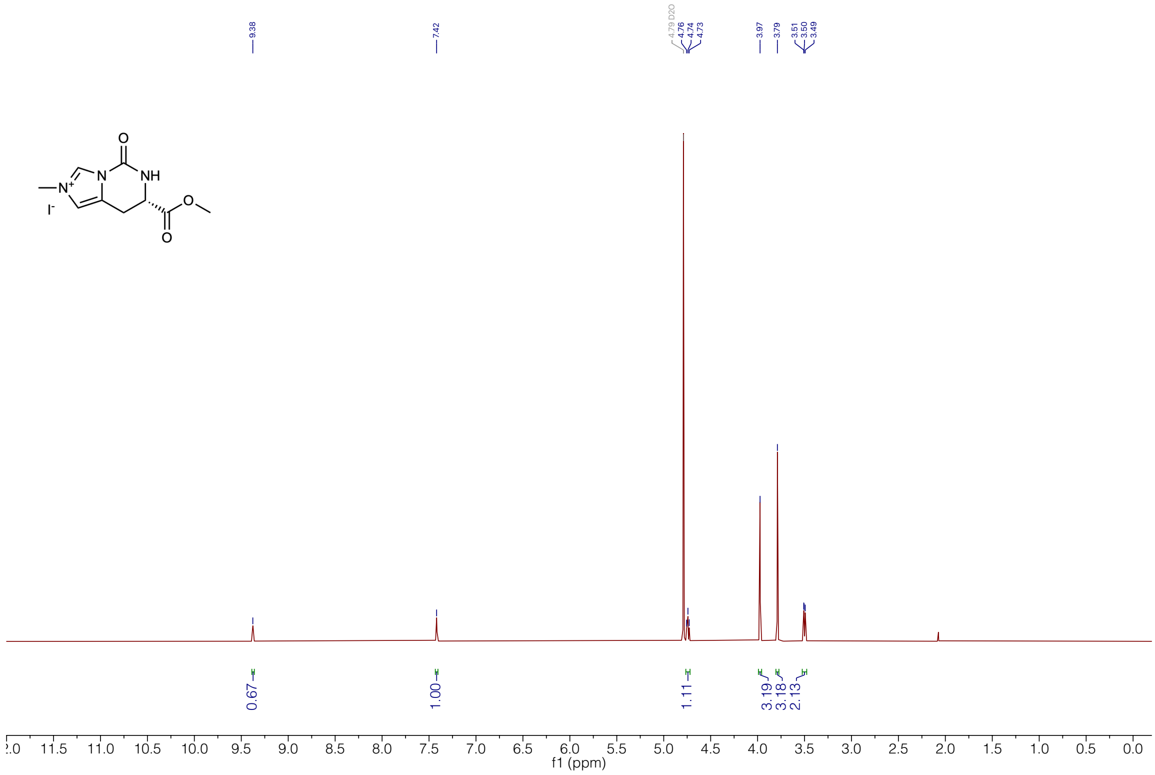

Supplementary Figure 58 | 1H-NMR of (S)-7-(methoxycarbonyl)-2-methyl-5-oxo-5,6,7,8-tetrahydroimidazo[1,5-c]pyrimidin-2-ium iodide.

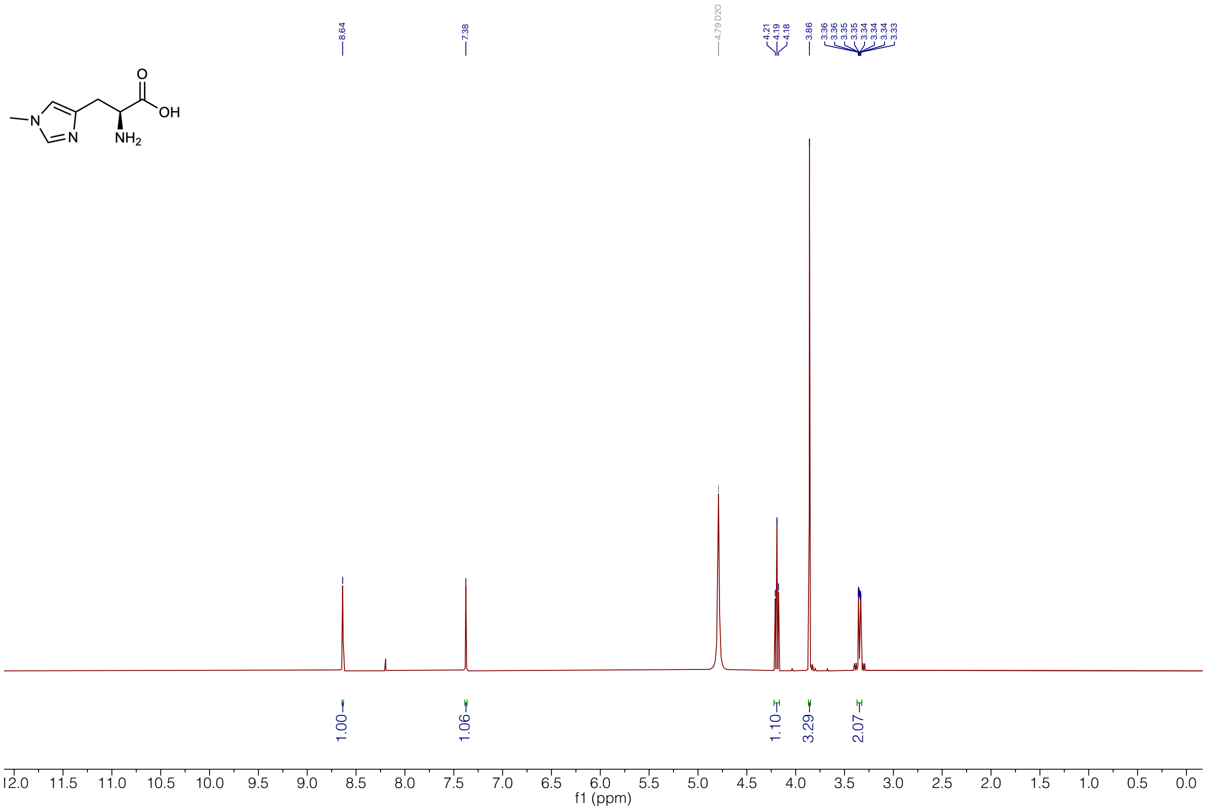

Supplementary Figure 59 | ^{1}H-NMR of N^{τ}-methyl-L-histidine (τMH).

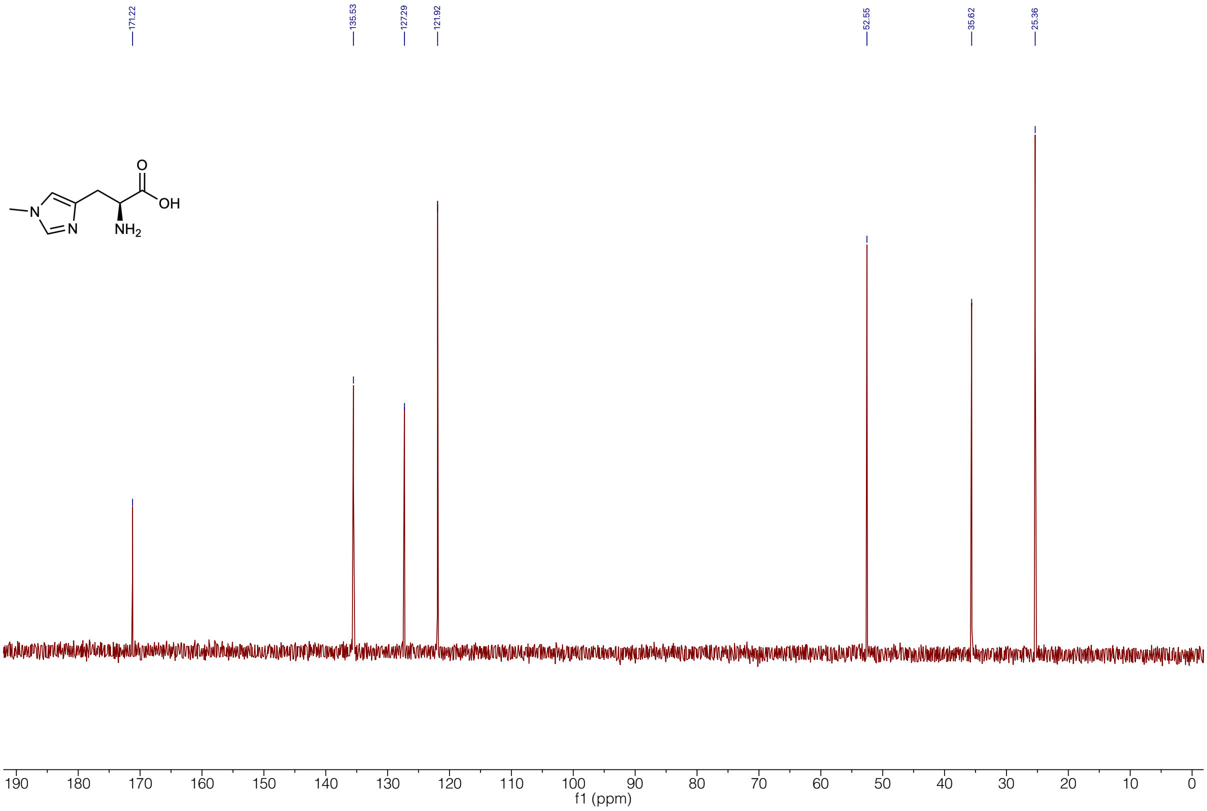

Supplementary Figure 60 | ^{13}C-NMR of N^{τ}-methyl-L-histidine (τMH).

Oxazol-5-yl-L-alanine synthesis (5OxzA)

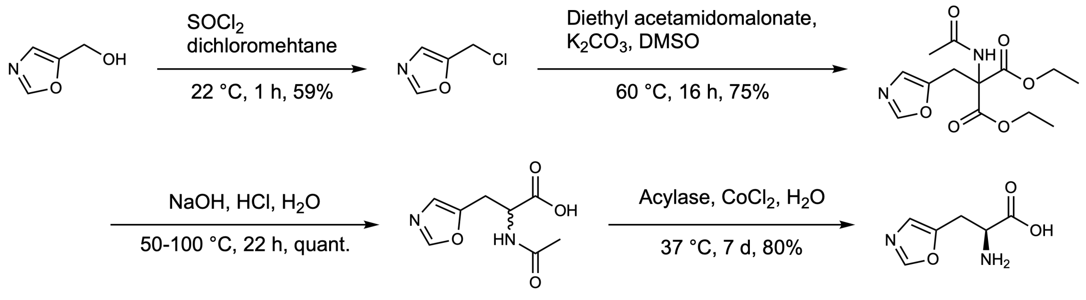

(Oxazol-5-yl)methylchloride:

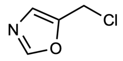

The reaction was performed under a nitrogen atmosphere. (Oxazol-5-yl)methanol (100 mg, 1.01 mmol, 1.0 eq.) was dissolved in dry dichloromethane (5 mL) and thienyl chloride (150 µL, 2.05 mmol, 2.0 eq.) was added. The solution was stirred for 1 h and the reaction was cooled to 4 °C. The reaction was quenched with aq. sat. $NaHCO_3$ (10 mL), the phases were separated, and the aqueous layer was extracted with EtOAc (3x). The combined organic phases were dried over Na_2SO_4 and the solvent was removed *in vacuo* to yield (oxaxol-5-yl)methanol (70 mg, 1.0 mmol, 59%) as a colorless oil.

^{1}H-NMR (400 MHz, $CDCl_3$) δ 7.90 (s, 1H), 7.11 (s, 1H), 4.63 (s, 2H).

Diethyl-2-acetamido-2-(oxazol-5-ylmethyl)malonate:

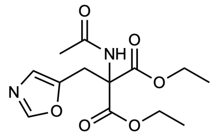

The following reaction was adapted from literature[9]. Diethyl acetamidomalonate (114 mg, 526 µmol, 1.0 eq.) was dissolved in DMSO (1.2 mL). (Oxazol-5-yl)methylchloride (68 mg, 579 µmol, 1.1 eq.) and K_2CO_3 (109 mg, 789 µmol, 1.5 eq.) were added and the suspension was stirred at 60 °C for 16 h. The reaction mixture was allowed to reach room temperature and ice-cold water (8 mL) was added. The mixture was extracted with EtOAc (2 x 10 mL), and the combined organic phase was washed with brine (10 mL). The organic phase was dried over Na_2SO_4 and the solvent was removed *in vacuo* to yield diethyl-2-acetamido-2-(oxazol-5-ylmethyl)malonate (118 mg, 526 mmol, 75%) as a white solid.

^{1}H-NMR (400 MHz, $CDCl_3$) δ 7.84 (s, 1H), 6.85 (s, 1H), 6.68 (s, 1H), 4.29 (q, $^3J_{HH}$ = 7.1 Hz, 4H), 3.82 (s, 2H), 2.03 (s, 3H), 1.29 (t, $^3J_{HH}$ = 7.1 Hz, 6H).

N-Acetyl-oxazol-5-yl-alanine:

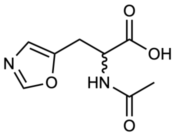

The following reaction was adapted from literature[13]. Diethyl-2-acetamido-2-(oxazol-5-yl-methyl)malonate (130 mg, 436 µmol, 1.0 eq.) was dissolved in water (7 mL) and NaOH (52 mg, 1.3 mmol, 3 eq.) was added. The solution was stirred at 50 °C for 16 h. The reaction mixture was adjusted to pH 4 with aq. HCl (4 M) and stirred at 100 °C for 6 h while adjusting the pH every few hours to pH 4. The solvent was removed *in vacuo* to yield *N*-acetyl-oxazol-5-yl-alanine (86 mg, 436 µmol, quant.) as a brown solid. The crude product was used for the next step without further purification.

Oxazol-5-yl-L-alanine (5OxzA):

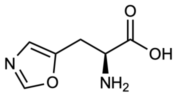

The following reaction was adapted from literature[13]. *N*-Acetyl-oxazol-5-yl-alanine (68 mg, 436 µmol, 1.0 eq.) was dissolved in water (1.2 mL) and $CoCl_2$ (0.27 mol%) was added. The mixture was adjusted to pH 7-8 and acylase from *Aspergillus genus* (1 mg) was added. The reaction mixture was stirred at 37 °C for 7 d and the solvent was removed *in vacuo* to yield an orange glue. The crude product was purified by automated flash column chromatography (C18, 1% acetonitrile in water (0.1% formic acid)) to yield oxazol-5-yl-L-alanine (27 mg, 174 µmol, 80%) as an off-white solid with formic acid as an impurity.

^{1}H-NMR (400 MHz, D_2O) δ 8.17 (s, 1H), 7.10 (s, 1H), 4.10 (dd, $^3J_{HH}$ = 6.4 Hz, $^3J_{HH}$ = 5.3 Hz, 1H), 3.47 – 3.35 (m, 2H).

^{13}C-NMR (101 MHz, D_2O) δ 173.0, 152.8, 147.2, 124.1, 53.2, 26.4.

MS (ESI): calc. for $[M+H]^+$: 157.05, obs.: 157.1.

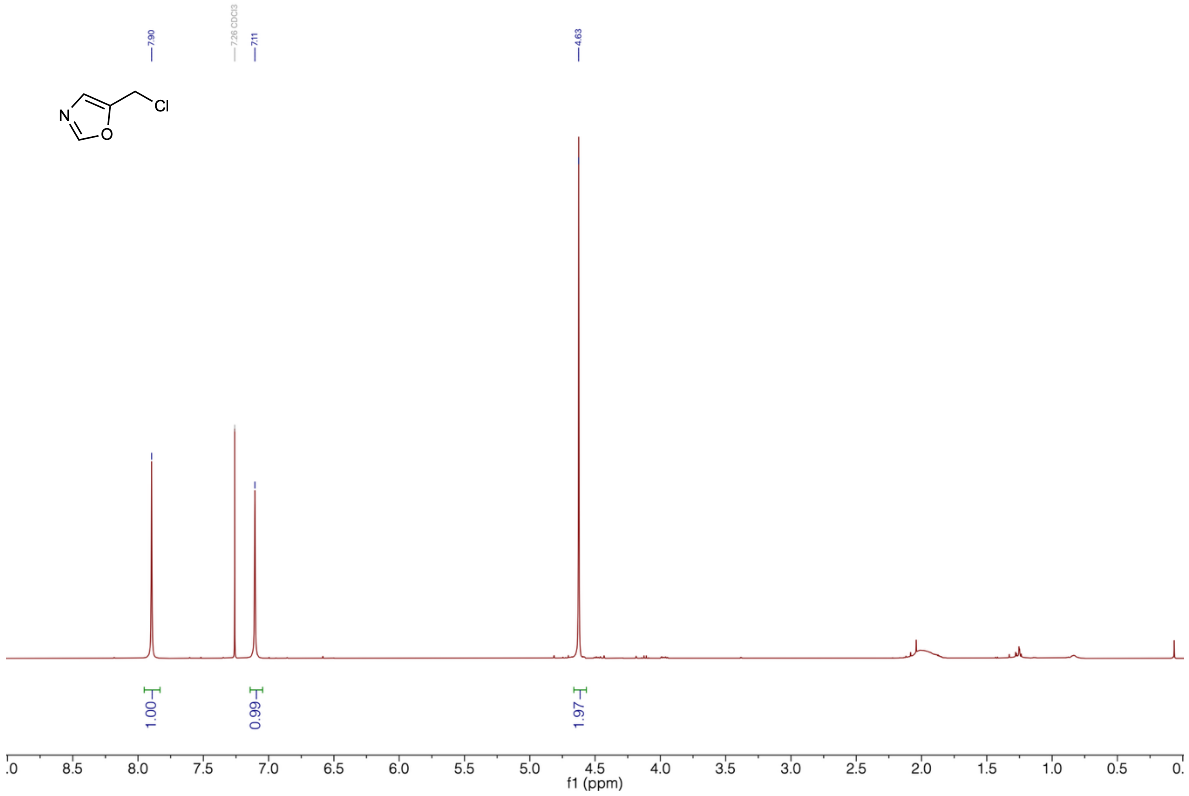

Supplementary Figure 61 | ¹H-NMR of (oxazol-5-yl)methylchloride.

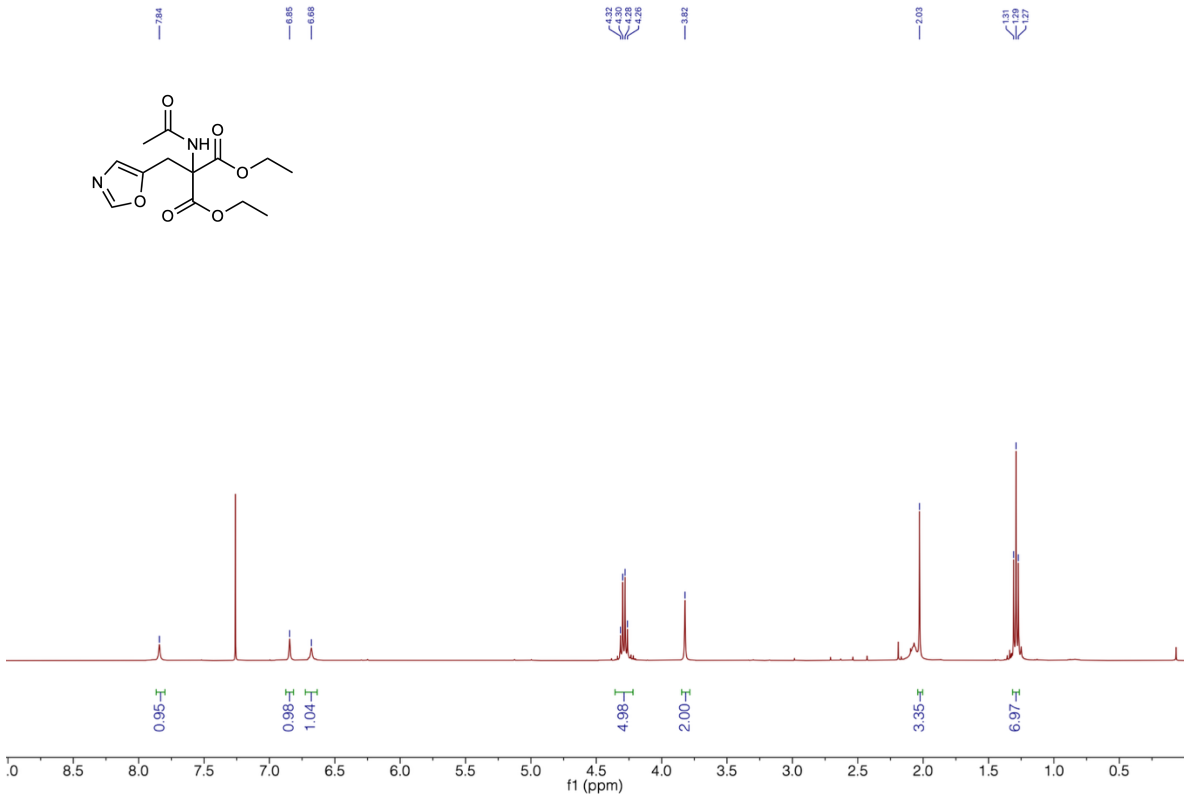

Supplementary Figure 62 | ¹H-NMR of diethyl-2-acetamido-2-(oxazol-5-ylmethyl)malonate.

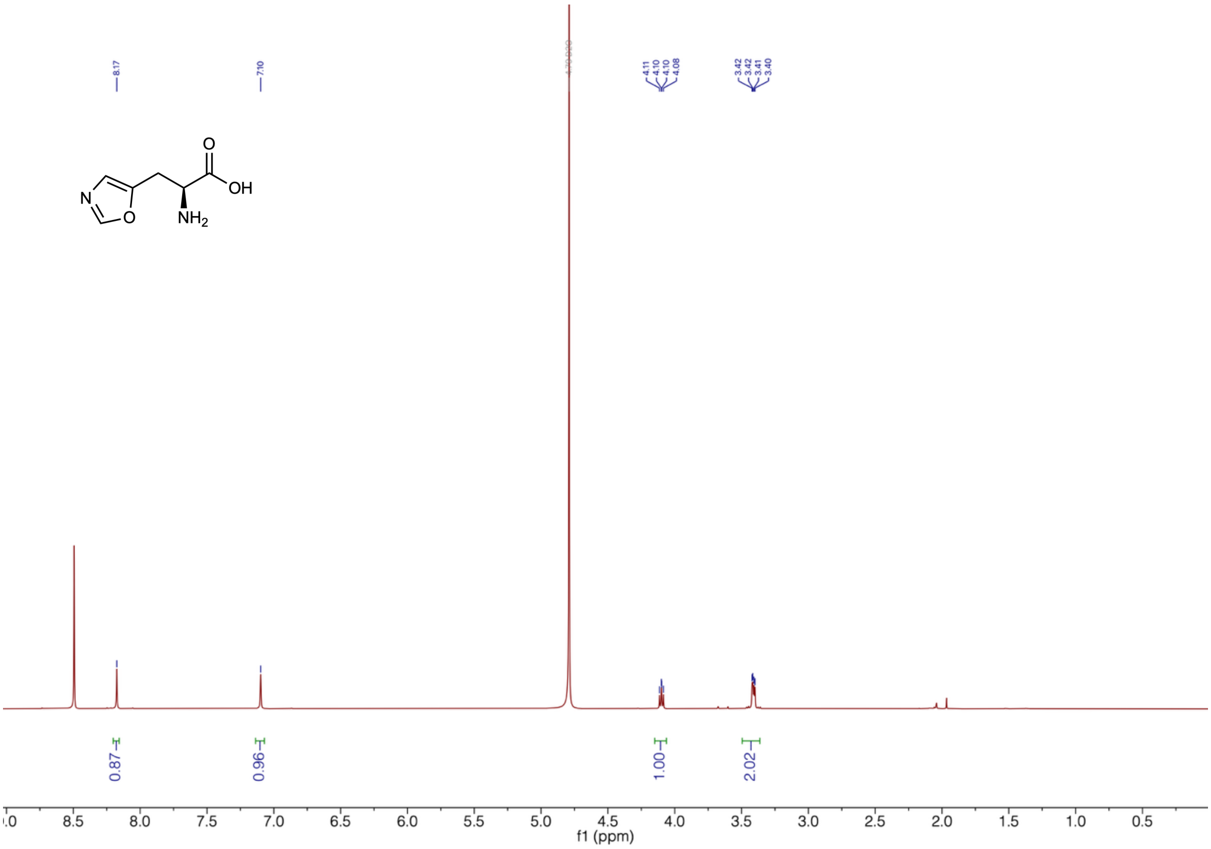

Supplementary Figure 63 | ^{1}H-NMR of oxazol-5-yl-alanaine (5OxzA)

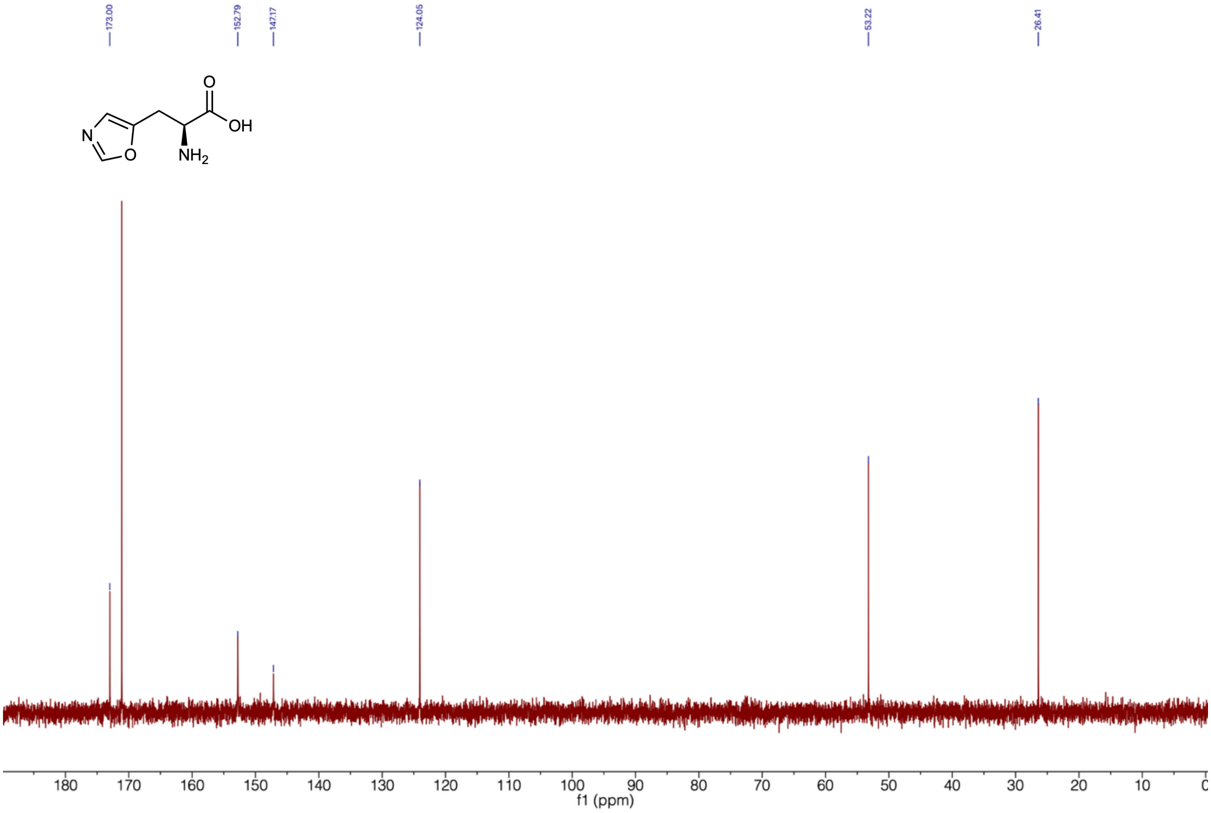

Supplementary Figure 64 | ^{13}C-NMR of oxazol-5-yl-alanaine (5OxzA).

III. Supplementary tables

Supplementary Table 1 | Concentrations of ncAAs used in this study. The following ncAA concentrations were used unless stated otherwise.

ncAA	ncAA abbreviation	Concentration (mM)
1,2,3-triazol-4-yl-L-alanine	123Trz-4A	4
2-pyridyl-L-alanine	2PyA	4
3-pyridyl-L-alanine	3PyA	5
3-pyrazolyl-alanine	3PzA	4
1,2,4-triazol-3-yl-alanine	124Trz-3A	5
1,2,4-triazol-1-yl-alanine	124Trz-1A	5
5-nitro-L-histidine	$5NO_2H$	5
2-ethyl-5-methyl-histidine	2E5MH	4
N^{τ}-benzyl-L-histidine	τBnH	2
1-benzyl-1,2,3-triazol-4-yl-L-alanine	1Bn123Trz-4A	2
4-pyridyl-alanine	4PyA	4
4-thiazolyl-L-alanine	4ThzA	6
5-thiazolyl-L-alanine	5ThzA	6
N^{τ}-methyl-L-histidine	τMH	5
N^{π}-methyl-L-histidine	πMH	5
2-thienyl-L-alanine	2ThA	8
3-thienyl-L-alanine	3ThA	8
oxazol-4-yl-L-alanine	4OxzA	5
oxazol-5-yl-L-alanine	5OxzA	5

Supplementary Table 2 | PylRS libraries screened for ncAA incorporation.

Library	Template	Fixed residues	Randomized residues (*Mm*PylRS residue numbering)
1	*Mm*PylRS	-	M300, A302, N346, C348, W417
2	*Mm*PylRS	-	L305, L309, N346, C348, V401
3	*Mm*PylRS	-	L305, L309, N346, C348, W417
4	*Mm*PylRS	-	L309, N346, C348, V401, W417
5	*Mm*PylRS	Y384F	A302, N346, C348
6	*Mm*PylRS	I405R	M300, A302, N346, C348
7	*Mm*PylRS	L305I, L309G, Y384F	A302, A306, N346, C348, V401
8	$chPylRS^{E7}$	-	A302, L306, V401, I405
9	$chPylRS^{E7}$	-	A302, N346, C348, I405
10	$chPylRS^{E7}$	-	L305, L309, N346, C348, V401
11	$chPylRS^{E7}$	-	L305, L309, N346, C348, W417

Supplementary Table 3 | Protein Sequences of N-terminal PylRS domains.

Organism of origin	Protein Sequence
Desulfacinum infernum	MLFRLIDKIKLWPSRSGTLHGIRAIEKSGDTALVTTHCGKTFFAN NSRNSRAARWLRNKWFHKVCGTCAVPQWKLEKY
Thermincola potens	MLLEKIKLWPSRSGTLHGIKTITIRGNTAEIVTHCNERFIVRNSRN SRAARWLRNKWHLGICRACRVPEWKLEKY
Caloranaerobacter ferrireducens	MLLKKIKLWPSRKGILHGIKSITVIGNKAEITTHCNEKFMIRNSKN SRASRWLRNKWLVKVCPRCKIPQWKLEKY

Methanohalobium evestigatum	MSKKSLASLISDLQVWVSRSGLLHEIKNYEVSQRYIHMEMDCGE KITVRNSRNSRTARILRLKKYKKPCKNCKVSDEVINRFLQKHTDRTDTKV

Supplementary Table 4 | chPylRS^{E7} libraries screened for 2PyA incorporation.

Library	Fixed residues	Randomized residues (*Mm*PylRS residue numbering)
12	N346A, C348A	M300, A302, V401, I405, W417
13	N346A, C348D	M300, A302, V401, I405, W417
14	N346S, C348D	M300, A302, V401, I405, W417
15	N346D, C348S	M300, A302, V401, I405, W417

Supplementary Table 5 | chPheRS libraries screened for 4PyA incorporation.

Library	Fixed residues	Randomized residues
16	T467G, A507G	Q365, E391, V393 T467, A507
17	T467G, A507G	Q365, T467, A507
18	T467G, A507G	M300, A302, V401, I405, W417
19	T467G, A507G	M300, A302, V401, I405, W417

Supplementary Table 6 | chPylRS^{E7} libraries screened for 4OxzA incorporation.

Library	Fixed residues	Randomized residues (*Mm*PylRS residue numbering)
20	A302Q	L305, L309, N346, C348, V401
21	A302Q	L305, Y306, L309, N346, C348

Supplementary Table 7 | Mutations of the synthetases evolved in this study.

Name	Template	Mutations (for chPylRS^{E7} constructs, *Mm*PylRS residue numbering was used)
*Mm*PylRS$^{123Trz\text{-}4A}$	*Mm*PylRS	A302W, L305I, Y306V, L309G, N346C, C348H, Y384F, V401C
*Mm*PylRS$^{\tau BnH}$	*Mm*PylRS	L309F, N346G, C348G
chPylRS^{2PyA}	chPylRS^{E7}	A302Q, N346A, C348D, V401C
chPylRS^{2E5MH}	chPylRS^{E7}	N346E, C348A, V401N
chPylRS^{5NO2H}	chPylRS^{E7}	A302H, N346S, C348Q, V401K
chPylRS^{4OxzA}	chPylRS^{E7}	A302Q, N346T, C348D
chPylRS$^{4OxzA\text{-}2}$	chPylRS^{E7}	A302Q, L305N, L309M, N346S, C348W
chPylRS^{3PzA}	chPylRS^{IPYE}	N346G, C348R, V401K
chPheRS$^{4Py\text{-}v0}$	chPheRS	Q365N, A507S
chPheRS^{4Py}	chPheRS$^{4Py\text{-}v0}$	L23M, V48G, T84A, P115L, S127P, R132H, S149P, L155R, R241G, H259L, W283C, F508I
Mb(IPYE)PylRS$^{\tau MH}$	*Mb*(IPYE)PylRS^{4ThzA}	S99F, T201I, D344N, S364T

Supplementary Table 8 | Expected masses for sfGFP150$_{TAG}$ production with ncAAs.

Amino acid at 150	ncAA mass (Da)	sfGFP150ncAA mass (Da)
Asn (wt GFP)	132.05	27827.32
3ThA	171.04	27866.31
2ThA	171.04	27866.31

2PyA	166.07	27861.34
3PyA	166.07	27861.34
4PyA	166.07	27861.34
τMH	169.09	27864.36
πMH	169.09	27864.36
4ThzA	172.03	27867.30
5ThzA	172.03	27867.30
τBnH	245.11	27940.38
1Bn123Trz-4A	246.11	27941.38
124Trz-3A	156.06	27851.33
123Trz-4A	156.06	27851.33
5OxzA	156.05	27851.32
4OxzA	156.05	27851.32
NO2H	200	27895.27
2E5MH	197.12	27892.39
3PzA	155.07	27850.34

Supplementary Table 9 | Analysis of misincorporation potential. See **Supplementary Table 10** for analysis of the incorporation fidelity for cases where misincorporation was observed by LC-MS/MS.

ncAA	***Lower bound**** **of fidelity based on sfGFP production ± ncAA**	**Observation of misincorporation by LC-MS/MS**
2ThA	50%	no
3ThA	89%	yes
3PyA	75%, 96%	yes, yes
4ThzA	94%	no
πMH	97%, 98%	no, no
123Trz-4A	83%	no
2PyA	95%	no
4PyA	95%	no
3PzA	97%	no
5NO_2H	94%	yes
2E5MH	97%	no
τBnH	94%	yes
1Bn123Trz-4A	89%	yes
124Trz-3A	89%	yes
5OxzA	75%	yes
4OxzA	88%	no
τMH	80%	yes

* The lower bound for fidelity was determined based on the ratio of sfGFP production in the presence and absence of the ncAA. Example: with four-fold higher sfGFP production in the presence vs the absence of the ncAA, a lower bound for fidelity was 75%.

Supplementary Table 10 | Details of LC-MS/MS observations.

	peptide	z	Amino acid	mass	Retention time	Peak area	Fraction	Comment
3ThA + *MmPylRS^7_1*	K.GIDFKEDGNILGHKLEYNFNSHJVYITADK.Q	5	3ThA	698.9404	39.8687	18469717	100%	Unique peptide+charges: 10<br>States with impurities: 2<br><br>Lower bound for purity: 96%
	K.EDGNILGHKLEYNFNSHJVYITADKQK.N	5	3ThA	638.112	35.1851	75589333	100%	
		4	3ThA	797.3881	35.1877	45565352	100%	
	K.LEYNFNSHJVYITADKQKNGIK.A	5	3ThA	527.8652	31.8243	28578349	100%	
		4	3ThA	659.5797	31.8217	39246651	100%	
	K.LEYNFNSHJVYITADKQK.N	4	3ThA	556.5188	33.2905	844390521	99%	
		4	Gln	550.2774	29.4312	11714403	1%	
		3	3ThA	741.6893	33.1004	493589366	96%	
		3	Phe	739.7038	33.6706	15943395	3%	
		3	Gln	733.3674	29.464	5970847	1%	
		2	3ThA	1112.0303	33.0426	11203927	100%	
	K.LEYNFNSHJVYITADK.Q	3	3ThA	656.3048	35.5322	678024974	100%	
		2	3ThA	983.9536	35.5745	127022507	100%	
3PyA + *MmPylRS^FLF*	K.LEYNFNSHJVYITADKQK.N	4	3PyA	555.2785	28.3235	437265173	84%	Unique peptide+charges: 5<br>States with impurities: 3<br><br>Lower bound for purity: 78%
		4	Phe	555.0297	33.7735	80811956	16%	
		3	3PyA	740.0356	28.3627	94269277	81%	
		3	Phe	739.7038	33.7964	21907303	19%	
	K.LEYNFNSHJVYITADK.Q	4	3PyA	491.2401	30.7739	318730217	100%	
		3	3PyA	654.6511	30.738	270936395	78%	
		3	Phe	654.3193	36.3715	76608508	22%	
		2	3PyA	981.473	30.4183	6912698	100%	
3PyA + *MmPylRS^8_2*	K.LEYNFNSHJVYITADKQKNGIK.A	5	3PyA	526.8729	28.359	38742342	100%	Unique peptide+charges: 6<br>States with impurities: 1<br><br>Lower bound for purity: 99%
		4	3PyA	658.3394	28.0548	38870771	100%	
	K.LEYNFNSHJVYITADKQK.N	4	3PyA	555.2785	29.1759	513108614	99%	
		4	Gln	550.2774	29.6386	4395555	1%	
		3	3PyA	740.0356	28.2843	104714024	100%	
	K.LEYNFNSHJVYITADK.Q	4	3PyA	491.2401	30.4923	188416756	100%	
		3	3PyA	654.6511	30.6509	153458568	100%	
5NO2H + chPylRS^5NO2H	K.GIDFKEDGNILGHKLEYNFNSHJVYITADK.Q	5	5NO$_2$H	704.7443	50.8307	38755278	99%	Unique peptide+charges: 12<br>States with impurities: 5<br><br>Lower bound for purity: 97%
		5	Gln	693.9473	50.0843	395146	1%	
		4	5NO$_2$H	880.6785	50.8473	34827974	99%	
		4	Gln	867.1823	50.0674	400837	1%	
		3	5NO$_2$H	1173.9023	50.9202	1353770	100%	
	K.EDGNILGHKLEYNFNSHJVYITADKQK.N	5	5NO$_2$H	643.9158	44.2882	11211685	100%	
		4	5NO$_2$H	804.6429	44.35	4922877	100%	
		4	Phe	795.899		Byonicscore too low	<1%	
	K.EDGNILGHKLEYNFNSHJVYITADK.Q	4	5NO$_2$H	740.6046	47.2104	5861952	100%	
		3	5NO$_2$H	987.137		177642	100%	
	K.LEYNFNSHJVYITADKQK.N	4	5NO$_2$H	563.7736	42.4193	326449725	99%	
		4	Trp	564.7824	46.395	1484036	0%	
		4	Gln	550.2774	38.2811	2023424	1%	
		3	5NO$_2$H	751.3624	40.6651	187341344	99%	
		3	Gln	733.3674	38.2555	1609189	1%	
		3	Trp	752.7075	46.3804	747945	0%	
		3	Phe	739.7038	44.9801	376747	0%	
		2	5NO$_2$H	1126.5399	39.8374	5564443	100%	
	K.LEYNFNSHJVYITADK.Q	3	5NO$_2$H	665.9778	43.678	174704920	97%	
		3	Phe	654.3193	49.0319	4468411	2%	
		3	Gln	647.9829	42.1101	1770761	1%	
		2	5NO$_2$H	998.4631	43.6522	16761798	100%	
τBnH + *MmPylRS^τBnH*	K.LEYNFNSHJVYITADKQK.N	3	τBnH	766.383	36.8617	930673	100%	Unique peptide+charges: 4<br>States with impurities: 2<br><br>Lower bound for purity: 89%
	K.LEYNFNSHJVYITADK.Q	4	τBnH	511.0007	40.433	231836279	100%	
		3	τBnH	680.9985	40.4286	170947677	91%	
		3	Gln	647.9829	38.6854	11600943	6%	
		3	Phe	654.3193	45.5489	5343780	3%	
		2	τBnH	1020.9941	40.4636	13337836	89%	
		2	Gln	971.4707	38.7457	1664242	11%	
1Bn123Trz-4zA + MmPylRS^τBnH	K.LEYNFNSHJVYITADK.Q	3	1Bn123Trz-4A	681.3302	48.4962	37062112	79%	Unique peptide+charges: 1<br>States with impurities: 1<br><br>Lower bound for purity: 79%
		3	Phe	654.3193	46.2824	1862330	4%	
		3	Gln	647.9829	38.1889	7982290	17%	
124Trz-3A + MmPylRS^123Trz-4A	K.EDGNILGHKLEYNFNSHJVYITADK.Q	4	124Trz-3A	729.6071	42.3603	961500	100%	Unique peptide+charges: 6<br>States with impurities: 2<br><br>Lower bound for purity: 93%
	K.LEYNFNSHJVYITADKQK.N	4	124Trz-3A	552.7761	35.298	8007527	100%	
		3	His	736.3675	35.251	2500846	0%	
	K.LEYNFNSHJVYITADK.Q	4	124Trz-3A	488.7377	38.737	29162507	100%	
		3	124Trz-3A	651.3146	38.836	153890130	93%	

	peptide	z	Amino acid	mass	Retention time	Peak area	Fraction	Comment
		3	Gln	647.9829	38.0891	11047377	7%	
		3	Tyr	659.6511	41.513	1210924	1%	
		2	124Trz-3A	976.4682	38.7938	11135598	100%	
**5OxzA + *MmPylRS^IFGFF***	K.LEYNFNSHJVYITADKQK.N	4	5OxzA	552.7733	30.9708	694877397	87%	Unique peptide+charges: 4<br>States with impurities: 3<br><br>Lower bound for purity: 86%
		4	Phe	555.0297	34.6105	105731006	13%	
		3	5OxzA	736.6953	30.9899	433099340	87%	
		3	Phe	739.7038	34.6451	64154192	13%	
	K.LEYNFNSHJVYITADK.Q	3	5OxzA	651.3108	33.4749	261109925	86%	
		3	Phe	654.3193	37.2903	34498740	11%	
		3	Gln	733.3674	30.2014	8131376	3%	
		2	5OxzA	976.4626	33.5182	39182356	100%	
**τMH + *Mb*(IPYE)PylRS^τMH**	K.GIDFKEDGNILGHKLEYNFNSHJVYITADK.Q	6	τMH	582.292	45.0182	1002187	100%	Unique peptide+charges: 12<br>States with impurities: 3<br><br>Lower bound for purity: 89%
		5	τMH	698.5489	45	5006834	100%	
		4	τMH	872.9343	44.9804	9077482	100%	
	K.EDGNILGHKLEYNFNSHJVYITADKQK.N	5	τMH	637.7205	37.4236	2609014	100%	
		4	τMH	796.8988	37.461	2188461	100%	
	K.EDGNILGHKLEYNFNSHJVYITADK.Q	4	τMH	732.8604	40.1782	2563201	100%	
	K.LEYNFNSHJVYITADKQKNGIK.A	4	τMH	659.0903	30.5532	10754267	100%	
		3	τMH	878.4513	30.5821	953424	100%	
	K.LEYNFNSHJVYITADKQK.N	4	τMH	556.0294	31.2283	58973801	97%	
		4	Phe	555.0297	41.9791	1848070	3%	
		3	τMH	741.0368	31.2561	13820191	89%	
		3	Phe	739.7038	41.9751	1737851	11%	
	K.LEYNFNSHJVYITADK.Q	4	τMH	491.991	34.7516	28763358	100%	
		3	τMH	655.6523	34.7472	39349222	95%	
		3	Phe	654.3193	46.0901	2234755	5%	

*The lower bound for purity was determined based on the lowest signal integration ratio for the desired ncAA incorporation vs. the sum of the desired ncAA incorporation and misincorporation of canonical amino acids within the same peptide and charge state (z). Notably, in most cases the lower bound is quite pessimistic as most systems have several peptides and charge states with 100% purity. However, because the different peptides and charge states may have different ionization efficiencies, we did not think there was a responsible way to mathematically merge the data for different peptides and charge states. See the methods section for information on the extracted ion chromatograms (XIC) analysis.

Supplementary Table 11 | aaRS-ncAA combinations for dual histidine-like-ncAA incorporation.

Combination	ncAA at 40_{TAG}	ncAA at 150_{TGA}
1	123Trz-4A	πMH
2	123Trz-4A	3PyA
3	2PyA	3PyA
4	2PyA	πMH
5	4ThzA	πMH
6	4PyA	πMH
**Combination**	**ncAA at 40_{TGA}**	**ncAA at 150_{TAG}**
1	πMH	123Trz-4A
2	3PyA	123Trz-4A
3	3PyA	2PyA
4	πMH	2PyA
5	πMH	4ThzA
6	πMH	4PyA

Supplementary Table 12 | List of primers used for PylRS library generation.

Name	Sequence 5'→3'	Targeted Positions
M300A302_f	GTAATTGGTCTCCGCCCGNNKCTGNNKCCGAACCTGTATAACTATCTG	M300, A302
M300A302_r	GTAATTGGTCTCCGGGCGCAGGCAAAAG	M300, A302
L305L309_f1	TATCTAGGTCTCATAACTATNDTCGCAAACTGGATCGCGCG	L305, L309
L305L309_f2	TATCTAGGTCTCTAACTATVMACGCAAACTGGATCGCGCG	L305, L309
L305L309_f3	TATCTAGGTCTCATAACTATTGGCGCAAACTGGATCGCGCG	L305, L309
L305L309_f4	TATCTAGGTCTCATAACTATATGCGCAAACTGGATCGCGCG	L305, L309
L305L309_r1	TATCTAGGTCTCAGTTATAAHNGTTCGGCGCCAGCATC	L305, L309
L305L309_r2	TATCTAGGTCTCAGTTATATKBGTTCGGCGCCAGCATC	L305, L309
L305L309_r3	TATCTAGGTCTCAGTTATACATGTTCGGCGCCAGCATC	L305, L309
L305L309_r4	TATCTAGGTCTCAGTTATACCAGTTCGGCGCCAGCATC	L305, L309
N346C348_f	GATTACGGTCTCCAGATGGGCAGCGGC	N346, C348
N346C348_r	GATACTGGTCTCCATCTGMNNAAAMNNCAGCATGGTAAATTCTTCCAG	N346, C348
S399A400V401_f	AATAAGGTCTCGTGGGCCCGCGCCC	S399, A400, V401
S399A400V401_r	AAATAAGGTCTCGCCCACMNNMNNMNNGCTCAGTTCCAGATCGCCA	S399, A400, V401
V401_f1	GATCATGGTCTCACCCACCATCGCGCTGCTCAGTTC	V401
V401_f2	GATCATGGTCTCACCCACAHNCGCGCTGCTCAGTTC	V401
V401_f3	GATCATGGTCTCACCCACTKBCGCGCTGCTCAGTTC	V401
V401_f4	GATCATGGTCTCACCCACCCACGCGCTGCTCAGTTC	V401
V401_r1	GATCATGGTCTCGTGGGCCCGATTCCGC	V401
W417_f1	TCTAGGTCTCAAACCANDTATTGGCGCGGGCTTTG	W417
W417_f2	TCTAGGTCTCAAACCAVMAATTGGCGCGGGCTTTG	W417
W417_f3	TCTAGGTCTCAAACCATGGATTGGCGCGGGCTTTG	W417
W417_f4	TCTAGGTCTCAAACCAATGATTGGCGCGGGCTTTG	W417
W417_r	TCTAGGTCTCGGTTTATCAATGCCCCATTCG	W417
W417G419_f	ATCTATGGTCTCGCGGGCTTTGG	W416G419
W417G419_r	ATCTATGGTCTCGCCCGCMNNAATMNNTGGTTTATCAATGCCCC	W416G419
G419_f1	GATCATGGTCTCTGGATTNDTGCGGGCTTTGGTCTG	G419
G419_f2	GATCATGGTCTCTGGATTVMAGCGGGCTTTGGTCTG	G419
G419_f3	GATCATGGTCTCTGGATTTGGGCGGGCTTTGGTCTG	G419
G419_f4	GATCATGGTCTCTGGATTATGGCGGGCTTTGGTCTG	G419
G419_r	GATTTCGGTCTCAATCCATGGTTTATCAATGCCC	G419

Supplementary Table 13 | List of primers used for chPheRS library generation.

Name	Sequence 5'→3'	Targeted Positions
T467NNK-F	AATAAGGTCTCCCTTTTNNKCATCCTTCCTTTGAGATGGAG	T467
T467NNK-R	GGTCTCAAAAGGMNNGTAGCAGTCTACCCACCTTA	T467
A507NNK-F	AATAAGGTCTCGGCTGGNNKTTTGGCCTAGGGTTAGAAAG	A507
A507NNK-R	AATAAGGTCTCCCAGCCGATTCGGTC	A507
T464NNK-R	GGTCTCAAAAGGMNNGTAGCAGTCTACCCACCTTA	T464
Q356NNK-F	AATAAGGTCTCGCACACNNKTGGGACTTGCTGCAC	Q356
Q356NNK-R	AATAAGGTCTCGTGTGCGGACGTGT	Q356
E391NNK_V393NNK-F	AATAAGGTCTCCAGCTGNNKGCCNNKCGGCTCTTCACCAAGC	E391, V393
E391NNK_V393NNK-R	AATAAGGTCTCCAGCTGGTGGAAAATAGGGTA	E391, V393

Supplementary Table 14 | List of primers used for chPylRS^{E7}-A314Q library generation.

Name	Sequence 5'→3'	**Targeted Positions**
A302Q_305_306_309-F	AATAAGGTCTCCCGAACNNKNNKAACTATTTTCGCAAACTGGAT	L305, Y306, L309
A302Q_305_306_309-R	AATAAGGTCTCGTTCGGMNNCAGCATCGGGCGC	L305, Y306, L309
A302Q_L305NNK_L309NNK-F	AATAAGGTCTCAGTTATAMNNGTTCGGCTGCAGCATCG	L305, L309
A302Q_L305NNK_L309NNK-R	AATAAGGTCTCATAACTATNNKCGCAAACTGGATCGCGC	L305, L309

Supplementary Table 15 | List of primers used for error-prone PCR of chPheRS.

Name	Sequence 5'→3'
Lib-in-F	GATACTGGTCTCGCTTTGAGGAATCCCATATG
Lib-in-R	CTAATTGGTCTCCGAAAAGTGCCACCTTTA
Lib-bb-F	GTAATTGGTCTCTTTTCGGGGAAATGTGC
Lib-bb-R	GATACTGGTCTCCAAAGCGTAAACAACGTATAACG

IV. DNA and Protein Sequences

DNA sequence of wild-type sfGFP:

```
ATGGTTAGCAAAGGTGAAGAACTGTTTACCGGCGTTGTGCCGATTCTGGTGGAACTGGATGGTGATGTGAATGGCCATAAATT
TAGCGTTCGTGGCGAAGGCGAAGGTGATGCGACCAACGGTAAACTGACCCTGAAATTTATTTGCACCACCGGTAAACTGCCGG
TTCCGTGGCCGACCCTGGTGACCACCCTGACCTATGGCGTTCAGTGCTTTAGCCGCTATCCGGATCATATGAAACGCCATGAT
TTCTTTAAAAGCGCGATGCCGGAAGGCTATGTGCAGGAACGTACCATTAGCTTCAAAGATGATGGCACCTATAAAACCCGTGC
GGAAGTTAAATTTGAAGGCGATACCCTGGTGAACCGCATTGAACTGAAAGGTATTGATTTTAAAGAAGATGGCAACATTCTGG
GTCATAAACTGGAATATAATTTCAACAGCCATAACGTGTATATTACCGCCGATAAACAGAAAAATGGCATCAAAGCGAACTTT
AAAATCCGTCACAACGTGGAAGATGGTAGCGTGCAGCTGGCGGATCATTATCAGCAGAATACCCCGATTGGTGATGGCCCGGT
GCTGCTGCCGGATAATCATTATCTGAGCACCCAGAGCGTTCTGAGCAAAGATCCGAATGAAAAACGTGATCATATGGTGCTGC
TGGAATTTGTTACCGCCGCGGGCATTACCCACGGTATGGATGAACTGTATAAAGGCAGCCACCATCATCATCACCATTAA
```

Protein sequence of wild-type sfGFP:

```
MVSKGEELFTGVVPILVELDGDVNGHKFSVRGEGEGDATNGKLTLKFICTTGKLPVPWPTLVTTLTYGVQCFSRYPDHMKRHD
FFKSAMPEGYVQERTISFKDDGTYKTRAEVKFEGDTLVNRIELKGIDFKEDGNILGHKLEYNFNSHNVYITADKQKNGIKANF
KIRHNVEDGSVQLADHYQQNTPIGDGPVLLPDNHYLSTQSVLSKDPNEKRDHMVLLEFVTAAGITHGMDELYKGSHHHHHH*
```

DNA sequence of sfGFP150$_{TAG}$:

```
ATGGTTAGCAAAGGTGAAGAACTGTTTACCGGCGTTGTGCCGATTCTGGTGGAACTGGATGGTGATGTGAATGGCCATAAATT
TAGCGTTCGTGGCGAAGGCGAAGGTGATGCGACCAACGGTAAACTGACCCTGAAATTTATTTGCACCACCGGTAAACTGCCGG
TTCCGTGGCCGACCCTGGTGACCACCCTGACCTATGGCGTTCAGTGCTTTAGCCGCTATCCGGATCATATGAAACGCCATGAT
TTCTTTAAAAGCGCGATGCCGGAAGGCTATGTGCAGGAACGTACCATTAGCTTCAAAGATGATGGCACCTATAAAACCCGTGC
GGAAGTTAAATTTGAAGGCGATACCCTGGTGAACCGCATTGAACTGAAAGGTATTGATTTTAAAGAAGATGGCAACATTCTGG
GTCATAAACTGGAATATAATTTCAACAGCCATTAGGTGTATATTACCGCCGATAAACAGAAAAATGGCATCAAAGCGAACTTT
AAAATCCGTCACAACGTGGAAGATGGTAGCGTGCAGCTGGCGGATCATTATCAGCAGAATACCCCGATTGGTGATGGCCCGGT
GCTGCTGCCGGATAATCATTATCTGAGCACCCAGAGCGTTCTGAGCAAAGATCCGAATGAAAAACGTGATCATATGGTGCTGC
TGGAATTTGTTACCGCCGCGGGCATTACCCACGGTATGGATGAACTGTATAAAGGCAGCCACCATCATCATCACCATTAA
```

Protein sequence of sfGFP150$_{TAG}$ with (ncAA) at position 150:

```
MVSKGEELFTGVVPILVELDGDVNGHKFSVRGEGEGDATNGKLTLKFICTTGKLPVPWPTLVTTLTYGVQCFSRYPDHMKRHD
FFKSAMPEGYVQERTISFKDDGTYKTRAEVKFEGDTLVNRIELKGIDFKEDGNILGHKLEYNFNSH(ncAA)VYITADKQKNG
IKANFKIRHNVEDGSVQLADHYQQNTPIGDGPVLLPDNHYLSTQSVLSKDPNEKRDHMVLLEFVTAAGITHGMDELYKGSHHH
HHH*
```

DNA sequence of sfGFP150$_{TGA}$:

```
ATGGTTAGCAAAGGTGAAGAACTGTTTACCGGCGTTGTGCCGATTCTGGTGGAACTGGATGGTGATGTGAATGGCCATAAATT
TAGCGTTCGTGGCGAAGGCGAAGGTGATGCGACCAACGGTAAACTGACCCTGAAATTTATTTGCACCACCGGTAAACTGCCGG
TTCCGTGGCCGACCCTGGTGACCACCCTGACCTATGGCGTTCAGTGCTTTAGCCGCTATCCGGATCATATGAAACGCCATGAT
TTCTTTAAAAGCGCGATGCCGGAAGGCTATGTGCAGGAACGTACCATTAGCTTCAAAGATGATGGCACCTATAAAACCCGTGC
GGAAGTTAAATTTGAAGGCGATACCCTGGTGAACCGCATTGAACTGAAAGGTATTGATTTTAAAGAAGATGGCAACATTCTGG
GTCATAAACTGGAATATAATTTCAACAGCCATTGAGTGTATATTACCGCCGATAAACAGAAAAATGGCATCAAAGCGAACTTT
AAAATCCGTCACAACGTGGAAGATGGTAGCGTGCAGCTGGCGGATCATTATCAGCAGAATACCCCGATTGGTGATGGCCCGGT
GCTGCTGCCGGATAATCATTATCTGAGCACCCAGAGCGTTCTGAGCAAAGATCCGAATGAAAAACGTGATCATATGGTGCTGC
TGGAATTTGTTACCGCCGCGGGCATTACCCACGGTATGGATGAACTGTATAAAGGCAGCCACCATCATCATCACCATTAA
```

Protein sequence of sfGFP150$_{TAG}$ with (ncAA) at position 150:

```
MVSKGEELFTGVVPILVELDGDVNGHKFSVRGEGEGDATNGKLTLKFICTTGKLPVPWPTLVTTLTYGVQCFSRYPDHMKRHD
FFKSAMPEGYVQERTISFKDDGTYKTRAEVKFEGDTLVNRIELKGIDFKEDGNILGHKLEYNFNSH(ncAA)VYITADKQKNG
IKANFKIRHNVEDGSVQLADHYQQNTPIGDGPVLLPDNHYLSTQSVLSKDPNEKRDHMVLLEFVTAAGITHGMDELYKGSHHH
HHH*
```

DNA sequence of sfGFP40$_{TAG}$150$_{TGA}$:

```
ATGGTTAGCAAAGGTGAAGAACTGTTTACCGGCGTTGTGCCGATTCTGGTGGAACTGGATGGTGATGTGAATGGCCATAAATT
TAGCGTTCGTGGCGAAGGCGAAGGTGATGCGACCTAGGGTAAACTGACCCTGAAATTTATTTGCACCACCGGTAAACTGCCGG
```

TTCCGTGGCCGACCCTGGTGACCACCCTGACCTATGGCGTTCAGTGCTTTAGCCGCTATCCGGATCATATGAAACGCCATGAT
TTCTTTAAAAGCGCGATGCCGGAAGGCTATGTGCAGGAACGTACCATTAGCTTCAAAGATGATGGCACCTATAAAACCCGTGC
GGAAGTTAAATTTGAAGGCGATACCCTGGTGAACCGCATTGAACTGAAAGGTATTGATTTTAAAGAAGATGGCAACATTCTGG
GTCATAAACTGGAATATAATTTCAACAGCCATTGAGTGTATATTACCGCCGATAAACAGAAAAATGGCATCAAAGCGAACTTT
AAAATCCGTCACAACGTGGAAGATGGTAGCGTGCAGCTGGCGGATCATTATCAGCAGAATACCCCGATTGGTGATGGCCCGGT
GCTGCTGCCGGATAATCATTATCTGAGCACCCAGAGCGTTCTGAGCAAAGATCCGAATGAAAAACGTGATCATATGGTGCTGC
TGGAATTTGTTACCGCCGCGGGCATTACCCACGGTATGGATGAACTGTATAAAGGCAGCCACCATCATCATCACCATTAA

Protein sequence of sfGFP40$_{TAG}$150$_{TGA}$ with (ncAA1) at position 40 and (ncAA2) at position 150:

MVSKGEELFTGVVPILVELDGDVNGHKFSVRGEGEGDAT(ncAA1)GKLTLKFICTTGKLPVPWPTLVTTLTYGVQCFSRYPD
HMKRHDFFKSAMPEGYVQERTISFKDDGTYKTRAEVKFEGDTLVNRIELKGIDFKEDGNILGHKLEYNFNSH(ncAA2)VYIT
ADKQKNGIKANFKIRHNVEDGSVQLADHYQQNTPIGDGPVLLPDNHYLSTQSVLSKDPNEKRDHMVLLEFVTAAGITHGMDEL
YKGSHHHHHH*

DNA sequence of sfGFP40$_{TGA}$:

ATGGTTAGCAAAGGTGAAGAACTGTTTACCGGCGTTGTGCCGATTCTGGTGGAACTGGATGGTGATGTGAATGGCCATAAATT
TAGCGTTCGTGGCGAAGGCGAAGGTGATGCGACCTGAGGTAAACTGACCCTGAAATTTATTTGCACCACCGGTAAACTGCCGG
TTCCGTGGCCGACCCTGGTGACCACCCTGACCTATGGCGTTCAGTGCTTTAGCCGCTATCCGGATCATATGAAACGCCATGAT
TTCTTTAAAAGCGCGATGCCGGAAGGCTATGTGCAGGAACGTACCATTAGCTTCAAAGATGATGGCACCTATAAAACCCGTGC
GGAAGTTAAATTTGAAGGCGATACCCTGGTGAACCGCATTGAACTGAAAGGTATTGATTTTAAAGAAGATGGCAACATTCTGG
GTCATAAACTGGAATATAATTTCAACAGCCATAACGTGTATATTACCGCCGATAAACAGAAAAATGGCATCAAAGCGAACTTT
AAAATCCGTCACAACGTGGAAGATGGTAGCGTGCAGCTGGCGGATCATTATCAGCAGAATACCCCGATTGGTGATGGCCCGGT
GCTGCTGCCGGATAATCATTATCTGAGCACCCAGAGCGTTCTGAGCAAAGATCCGAATGAAAAACGTGATCATATGGTGCTGC
TGGAATTTGTTACCGCCGCGGGCATTACCCACGGTATGGATGAACTGTATAAAGGCAGCCACCATCATCATCACCATTAA

Protein sequence of sfGFP40$_{TGA}$ with (ncAA) at position 40:

MVSKGEELFTGVVPILVELDGDVNGHKFSVRGEGEGDAT(ncAA)GKLTLKFICTTGKLPVPWPTLVTTLTYGVQCFSRYPDH
MKRHDFFKSAMPEGYVQERTISFKDDGTYKTRAEVKFEGDTLVNRIELKGIDFKEDGNILGHKLEYNFNSHNVYITADKQKNG
IKANFKIRHNVEDGSVQLADHYQQNTPIGDGPVLLPDNHYLSTQSVLSKDPNEKRDHMVLLEFVTAAGITHGMDELYKGSHHH
HHH*

DNA sequence of sfGFP40$_{TGA}$150$_{TAG}$:

ATGGTTAGCAAAGGTGAAGAACTGTTTACCGGCGTTGTGCCGATTCTGGTGGAACTGGATGGTGATGTGAATGGCCATAAATT
TAGCGTTCGTGGCGAAGGCGAAGGTGATGCGACCTGAGGTAAACTGACCCTGAAATTTATTTGCACCACCGGTAAACTGCCGG
TTCCGTGGCCGACCCTGGTGACCACCCTGACCTATGGCGTTCAGTGCTTTAGCCGCTATCCGGATCATATGAAACGCCATGAT
TTCTTTAAAAGCGCGATGCCGGAAGGCTATGTGCAGGAACGTACCATTAGCTTCAAAGATGATGGCACCTATAAAACCCGTGC
GGAAGTTAAATTTGAAGGCGATACCCTGGTGAACCGCATTGAACTGAAAGGTATTGATTTTAAAGAAGATGGCAACATTCTGG
GTCATAAACTGGAATATAATTTCAACAGCCATTAGGTGTATATTACCGCCGATAAACAGAAAAATGGCATCAAAGCGAACTTT
AAAATCCGTCACAACGTGGAAGATGGTAGCGTGCAGCTGGCGGATCATTATCAGCAGAATACCCCGATTGGTGATGGCCCGGT
GCTGCTGCCGGATAATCATTATCTGAGCACCCAGAGCGTTCTGAGCAAAGATCCGAATGAAAAACGTGATCATATGGTGCTGC
TGGAATTTGTTACCGCCGCGGGCATTACCCACGGTATGGATGAACTGTATAAAGGCAGCCACCATCATCATCACCATTAA

Protein sequence of sfGFP40$_{TGA}$150$_{TAG}$ with (ncAA1) at position 40 and (ncAA2) at position 150:

MVSKGEELFTGVVPILVELDGDVNGHKFSVRGEGEGDAT(ncAA1)GKLTLKFICTTGKLPVPWPTLVTTLTYGVQCFSRYPD
HMKRHDFFKSAMPEGYVQERTISFKDDGTYKTRAEVKFEGDTLVNRIELKGIDFKEDGNILGHKLEYNFNSH(ncAA2)VYIT
ADKQKNGIKANFKIRHNVEDGSVQLADHYQQNTPIGDGPVLLPDNHYLSTQSVLSKDPNEKRDHMVLLEFVTAAGITHGMDEL
YKGSHHHHHH*

DNA sequence of *Mm*PylRS:

ATGGATAAAAAACCGCTGAACACCCTGATTAGCGCGACCGGCCTGTGGATGAGCCGCACCGGCACCATTCATAAAATTAAACA
TCATGAAGTGAGCCGCAGCAAAATTTATATTGAAATGGCGTGCGGCGATCATCTGGTGGTGAACAACAGCCGCAGCAGCCGCA
CCGCGCGCGCGCTGCGCCATCATAAATATCGCAAAACCTGCAAACGCTGCCGCGTGAGCGATGAAGATCTGAACAAATTTCTG
ACCAAAGCGAACGAAGATCAGACCAGCGTGAAAGTGAAAGTGGTGAGCGCGCCGACCCGCACCAAAAAAGCGATGCCGAAAAG
CGTGGCGCGCGCGCCGAAACCGCTGGAAAACACCGAAGCGGCGCAGGCGCAGCCGAGCGGCAGCAAATTTAGCCCGGCGATTC
CGGTGAGCACCCAGGAAAGCGTGAGCGTGCCGGCGAGCGTGAGCACCAGCATTAGCAGCATTAGCACCGGCGCGACCGCGAGC
GCGCTGGTGAAAGGCAACACCAACCCGATTACCAGCATGAGCGCGCCGGTGCAGGCGAGCGCGCCGGCGCTGACCAAAAGCCA
GACCGATCGCCTGGAAGTGCTGCTGAACCCGAAAGATGAAATTAGCCTGAACAGCGGCAAACCGTTTCGCGAACTGGAAAGCG
AACTGCTGAGCCGCCGCAAAAAAGATCTGCAGCAGATTTATGCGGAAGAACGCGAAAACTATCTGGGCAAACTGGAACGCGAA

```
ATTACCCGCTTTTTTGTGGATCGCGGCTTTCTGGAAATTAAAAGCCCGATTCTGATTCCGCTGGAATATATTGAACGCATGGG
CATTGATAACGATACCGAACTGAGCAAACAGATTTTTCGCGTGGATAAAAACTTTTGCCTGCGCCCGATGCTGGCGCCGAACC
TGTATAACTATCTGCGCAAACTGGATCGCGCGCTGCCGGATCCGATTAAAATTTTTGAAATTGGCCCGTGCTATCGCAAAGAA
AGCGATGGCAAAGAACATCTGGAAGAATTTACCATGCTGAACTTTTGCCAGATGGGCAGCGGCTGCACCCGCGAAAACCTGGA
AAGCATTATTACCGATTTTCTGAACCATCTGGGCATTGATTTTAAAATTGTGGGCGATAGCTGCATGGTGTATGGCGATACCC
TGGATGTGATGCATGGCGATCTGGAACTGAGCAGCGCGGTGGTGGGCCCGATTCCGCTGGATCGCGAATGGGGCATTGATAAA
CCGTGGATTGGCGCGGGCTTTGGCCTGGAACGCCTGCTGAAAGTGAAACATGATTTTAAAAACATTAAACGCGCGGCGCGCAG
CGAAAGCTATTATAACGGCATTAGCACCAACCTGTAA
```

Protein sequence of *Mm*PylRS:

```
MDKKPLNTLISATGLWMSRTGTIHKIKHHEVSRSKIYIEMACGDHLVVNNSRSSRTARALRHHKYRKTCKRCRVSDEDLNKFL
TKANEDQTSVKVKVVSAPTRTKKAMPKSVARAPKPLENTEAAQAQPSGSKFSPAIPVSTQESVSVPASVSTSISSISTGATAS
ALVKGNTNPITSMSAPVQASAPALTKSQTDRLEVLLNPKDEISLNSGKPFRELESELLSRRKKDLQQIYAEERENYLGKLERE
ITRFFVDRGFLEIKSPILIPLEYIERMGIDNDTELSKQIFRVDKNFCLRPMLAPNLYNYLRKLDRALPDPIKIFEIGPCYRKE
SDGKEHLEEFTMLNFCQMGSGCTRENLESIITDFLNHLGIDFKIVGDSCMVYGDTLDVMHGDLELSSAVVGPIPLDREWGIDK
PWIGAGFGLERLLKVKHDFKNIKRAARSESYYNGISTNL*
```

DNA sequence of chPylRS^{e7}:

```
ATGAGCAAAAAAAGCCTGGCGtgcCTGATTAGCGATCTGCAGGTGTGGGTGAGCCGCAGCGGCCTGCTGCATGAAATTAAAAA
CTATGAAGTGAGCCAGCGCTATATTCATATGGAAATGgatTGCGGCGAAgaaATTACCGTGCGCAACAGCCGCAACAGCCGCA
CCGCGCGCATTCTGCGCCTGAAAAAATATaaaAAAcagTGCataAACTGCAAAGTGAGCGATGAAGTGATTagcCGCTTTCTG
CAGAAACATACCGATCGCACCGATACCataGTGctgACCaaaGCGAACGAAGATCAGACCAGCGTGAAAGTGAAAGTGGTGAG
CGCGCCGACCCGCACCAAAAAAGCGATGCCGAAAAGCGTGGCGCGCGCGCCGAAACCGCTGGAAAACACCGAAGCGGCGCAGG
CGCAGCCGAGCGGCAGCAAATTTAGCCCGGCGATTCCGGTGAGCACCCAGGAAAGCGTGAGCGTGCCGGCGAGCGTGAGCACC
AGCATTAGCAGCATTAGCACCGGCGCGACCGCGAGCGCGCTGGTGAAAGGCAACACCAACCCGATTACCAGCATGAGCGCGCC
GGTGCAGGCGAGCGCGCCGGCGCTGACCAAAAGCCAGACCGATCGCCTGGAAGTGCTGCTGAACCCGAAAGATGAAATTAGCC
TGAACAGCGGCAAACCGTTTCGCGAACTGGAAAGCGAACTGCTGAGCCGCCGCAAAAAAGATCTGCAGCAGATTTATGCGGAA
GAACGCGAAAACTATCTGGGCAAACTGGAACGCGAAATTACCCGCTTTTTTGTGGATCGCGGCTTTCTGGAAATTAAAAGCCC
GATTCTGATTCCGCTGGAATATATTGAACGCATGGGCATTGATAACGATACCGAACTGAGCAAACAGATTTTTCGCGTGGATA
AAAACTTTTGCCTGCGCCCGATGCTGGCGCCGAACCTGTATAACTATCTGCGCAAACTGGATCGCGCGCTGCCGGATCCGATT
AAAATTTTTGAAATTGGCCCGTGCTATCGCAAAGAAAGCGATGGCAAAGAACATCTGGAAGAATTTACCATGCTGAACTTTTG
CCAGATGGGCAGCGGCTGCACCCGCGAAAACCTGGAAAGCATTATTACCGATTTTCTGAACCATCTGGGCATTGATTTTAAAA
TTGTGGGCGATAGCTGCATGGTGTATGGCGATACCCTGGATGTGATGCATGGCGATCTGGAACTGAGCAGCGCGGTGGTGGGC
CCGATTCCGCTGGATCGCGAATGGGGCATTGATAAACCGTGGATTGGCGCGGGCTTTGGCCTGGAACGCCTGCTGAAAGTGAA
ACATGATTTTAAAAACATTAAACGCGCGGCGCGCAGCGAAAGCTATTATAACGGCATTAGCACCAACCTGTAA
```

Protein sequence of chPylRS^{e7}:

```
MSKKSLACLISDLQVWVSRSGLLHEIKNYEVSQRYIHMEMDCGEEITVRNSRNSRTARILRLKKYKKQCINCKVSDEVISRFL
QKHTDRTDTIVLTKANEDQTSVKVKVVSAPTRTKKAMPKSVARAPKPLENTEAAQAQPSGSKFSPAIPVSTQESVSVPASVST
SISSISTGATASALVKGNTNPITSMSAPVQASAPALTKSQTDRLEVLLNPKDEISLNSGKPFRELESELLSRRKKDLQQIYAE
ERENYLGKLEREITRFFVDRGFLEIKSPILIPLEYIERMGIDNDTELSKQIFRVDKNFCLRPMLAPNLYNYLRKLDRALPDPI
KIFEIGPCYRKESDGKEHLEEFTMLNFCQMGSGCTRENLESIITDFLNHLGIDFKIVGDSCMVYGDTLDVMHGDLELSSAVVG
PIPLDREWGIDKPWIGAGFGLERLLKVKHDFKNIKRAARSESYYNGISTNL*
```

DNA sequence of chPylRS^{IPYE}:

```
ATGGATAAGAAGCCGCTGGATGTTCTGATCTCTGCGACCGGTCTGTGGATGTCCCGTACCGGCACGCTGCACAAGATCAAGCA
CTATGAGATTTCTCGTTCTAAAATCTACATCGAAATGGCGTGTGGTGACCATCTGGTTGTGAACAACTCTCGTTCTTGTCGTC
CCGCACGTGCATTCCGTTATCATAAATACCGTAAAACCTGCAAACGTTGTCGTGTTTCTGACGAAGATATCAACAACTTCCTG
ACCCGTTCTACCGAAGGCAAAACCTCTGTTAAAGTTAAAGTTGTTTCTGAGCCGAAAGTGAAAAAAGCGATGCCGAAATCTGT
TTCTCGTGCGCCGAAACCGCTGGAAAATCCGGTTTCTGCGAAAGCGTCTACCGACACCTCTCGTTCTGTTCCGTCTCCGGCGA
AATCTACCCCGAACTCTCCGGTTCCGACCTCTGCGAGCGCGCCGGCGCTGACCAAAAGCCAGACCGATCGCCTGGAAGTGCTG
CTGAACCCGAAAGATGAAATTAGCCTGAACAGCGGCAAACCGTTTCGCGAACTGGAAAGCGAACTGCTGAGCCGCCGCAAAAA
AGATCTGCAGCAGATTTATGCGGAAGAACGCGAAAACTATCTGGGCAAACTGGAACGCGAAATTACCCGCTTTTTTGTGGATC
GCGGCTTTCTGGAAATTAAAAGCCCGATTCTGATTCCGCTGGAATATATTGAACGCATGGGCATTGATAACGATACCGAACTG
AGCAAACAGATTTTTCGCGTGGATAAAAACTTTTGCCTGCGCCCGATGCTGGCGCCGAACCTGTATAACTATCTGCGCAAACT
GGATCGCGCGCTGCCGGATCCGATTAAAATTTTTGAAATTGGCCCGTGCTATCGCAAAGAAAGCGATGGCAAAGAACATCTGG
AAGAATTTACCATGCTGAACTTTTGCCAGATGGGCAGCGGCTGCACCCGCGAAAACCTGGAAAGCATTATTACCGATTTTCTG
AACCATCTGGGCATTGATTTTAAAATTGTGGGCGATAGCTGCATGGTGTATGGCGATACCCTGGATGTGATGCATGGCGATCT
GGAACTGAGCAGCGCGGTGGTGGGCCCGATTCCGCTGGATCGCGAATGGGGCATTGATAAACCGTGGATTGGCGCGGGCTTTG
GCCTGGAACGCCTGCTGAAAGTGAAACATGATTTTAAAAACATTAAACGCGCGGCGCGCAGCGAAAGCTATTATAACGGCATT
AGCACCAACCTGTAA
```

Protein sequence of chPylRS^{IPYE}:

MDKKPLDVLISATGLWMSRTGTLHKIKHYEISRSKIYIEMACGDHLVVNNSRSCRPARAFRYHKYRKTCKRCRVSDEDINNFL
TRSTEGKTSVKVKVVSEPKVKKAMPKSVSRAPKPLENPVSAKASTDTSRSVPSPAKSTPNSPVPTSASAPALTKSQTDRLEVL
LNPKDEISLNSGKPFRELESELLSRRKKDLQQIYAEERENYLGKLEREITRFFVDRGFLEIKSPILIPLEYIERMGIDNDTEL
SKQIFRVDKNFCLRPMLAPNLYNYLRKLDRALPDPIKIFEIGPCYRKESDGKEHLEEFTMLNFCQMGSGCTRENLESIITDFL
NHLGIDFKIVGDSCMVYGDTLDVMHGDLELSSAVVGPIPLDREWGIDKPWIGAGFGLERLLKVKHDFKNIKRAARSESYYNGI
STNL*

DNA sequence of chPheRS(T467G, A507G):

ATGGATAAGAAGCCGCTGGATGTTCTGATCTCTGCGACCGGcCTGTGGATGTCCCGTACCGGCACGCTGCACAAGATCAAGCA
CTATGAGATTTCTCGTTCTAAAATCTACATCGAAATGGCGTGTGGTGACCATCTGGTTGTGAACAACTCTCGTTCTTGTCGTC
CCGCACGTGCATTCCGTTATCATAAATACCGTAAAACCTGCAAACGTTGTCGTGTTTCTGACGAAGATATCAACAACTTCCTG
ACCCGTTCTACCGAAGGCAAAACCTCTGTTAAAGTTAAAGTTGTTTCTGAGCCGAAAGTGAAAAAAGCGATGCCGAAATCTGT
TTCTCGTGCGCCGAAACCGCTGGAAAATCCGGTTTCTGCGAAAGCGTCTACCGACACCTCTCGTTCTGTTCCGTCTCCGGCGA
AATCTACCCCGAACTCTCCGGTTCCGACCTCTGCAAGCGCCCCAGCTCTGACTAAATCCCAGACGGACCGTCTGGAGGTGCTG
CTGAACCCAAAGGATGAAATCTCTCTGAACAGCGGCAAGCCTTTCCGTGAGCTGGAAAGCGAGCTGCTGTCTCGTCGTAAAAA
GGATCTGCAACAGATCTACGCTGAGGAACGCGAGGGTGGCGGAAGCGGTGGCGGAAGCGGTGGCGGAAGTGGTGGCGGAAGCG
GCGGCGGAAGCCAGGCCTGGGGATCCAGGCCTCCTGCAGCAGAGTGTGCCACCCAAAGAGCTCCAGGCAGTGTGGTGGAGCTG
CTGGGCAAATCCTACCCTCAGGACGACCACAGCAACCTCACCCGGAAGGTCCTCACCAGAGTTGGCAGGAACCTGCACAACCA
GCAGCATCACCCTCTGTGGCTGATCAAGGAGAGGGTGAAGGAGCACTTCTACAAGCAGTATGTGGGCCGCTTTGGGACCCCGT
TGTTCTCTGTCTACGACAACCTTTCTCCAGTGGTCACGACCTGGCAGAACTTTGACAGCCTGCTCATCCCAGCTGATCACCCC
AGCAGGAAGAAGGGGGACAACTATTACCTGAATCGGACTCACATGCTGAGAGCGCACACGTCcGCAcacCAGTGGGACTTGCT
GCACGCGGGACTGGATGCCTTCCTGGTGGTGGGTGATGTCTACAGGCGTGACCAGATCGACTCCCAGCACTACCCTATTTTCC
ACCAGCTGGAGGCCGTGCGGCTCTTCTCCAAGCATGAGTTATTTGCTGGTATAAAGGATGGAGAAAGCCTGCAGCTCTTTGAA
CAAAGTTCTCGCTCTGCGCATAAACAAGAGACACACACCATGGAGGCCGTGAAGCTTGTAGAGTTTGATCTTAAGCAAACGCT
TACCAGGCTCATGGCACATCTTTTTGGAGATGAGCTGGAGATAAGGTGGGTAGACTGCTACTTCCCTTTTGGCCATCCTTCCT
TTGAGATGGAGATCAACTTTCATGGAGAATGGCTGGAAGTTCTTGGCTGCGGGGTGATGGAACAACAACTGGTCAATTCtGCT
GGTGCTCAAGACCGAATCGGCTGGGGTTTTGGCCTAGGGTTAGAAAGGCTAGCCATGATCCTCTACGACATCCCTGATATCCG
TCTCTTCTGGTGTGAGGACGAGCGCTTCCTGAAGCAGTTCTGTGTATCCAACATTAATCAGAAGGTGAAGTTTCAGCCTCTTA
GCAAATAA

Protein sequence of chPheRS(T467G, A507G):

MDKKPLDVLISATGLWMSRTGTLHKIKHYEISRSKIYIEMACGDHLVVNNSRSCRPARAFRYHKYRKTCKRCRVSDEDINNFL
TRSTEGKTSVKVKVVSEPKVKKAMPKSVSRAPKPLENPVSAKASTDTSRSVPSPAKSTPNSPVPTSASAPALTKSQTDRLEVL
LNPKDEISLNSGKPFRELESELLSRRKKDLQQIYAEEREGGGSGGGSGGGSGGGSGGGSQAWGSRPPAAECATQRAPGSVVEL
LGKSYPQDDHSNLTRKVLTRVGRNLHNQQHHPLWLIKERVKEHFYKQYVGRFGTPLFSVYDNLSPVVTTWQNFDSLLIPADHP
SRKKGDNYYLNRTHMLRAHTSAHQWDLLHAGLDAFLVVGDVYRRDQIDSQHYPIFHQLEAVRLFSKHELFAGIKDGESLQLFE
QSSRSAHKQETHTMEAVKLVEFDLKQTLTRLMAHLFGDELEIRWVDCYFPFGHPSFEMEINFHGEWLEVLGCGVMEQQLVNSA
GAQDRIGWGFGLGLERLAMILYDIPDIRLFWCEDERFLKQFCVSNINQKVKFQPLSK*

DNA sequence of S^{Δ}-*Clos*PylRS^{QSW}:

ATGGAAAATTTTACCATCACCCAGACGGAACGCCTTAAACAACTTAATTGTGAGAATGATGTATTGGAGCTGGAATTTGAAGA
TTCCGAGGCTCGCAATTCCAAGTTTCGTGAAATTGAGATTGGGCGCGTGAAAAAGGGCAAGGAAAATATCAAAAACCTGCTTA
AGGAAAAGCATATCACGATCTCGGACGAAGTAGGGAACAAGTTGTCAGATTGGCTTATGAGCAAAGATTATACTAAAGTCCTT
ACCCCGACTATCATCAGCAAGGACCAGTTAAAGGCAATGACTATCGACGAAGAGAATCACTTGTTTTCACAAGTTTTTTGGAT
CGATAATAACAAGTGTCTGCGCCCTATGCTGCAGCCGAATCTGTATATCGTCATGCGTGAGTTGAAGCGTATCACGAATGAAC
CCGTAAAGATTTTCGAGATTGGATCATGCTTTCGTAAGGAGTCGCAAGGCGCACGTCACATGAATGAGTTTACCATGCTGTCC
ATGTGGGAGCTGGCGTCCGTGGAGGATGGAAAGCAATTGGACACATTGAAAGCACTGGCGCATGAGGCAATGGAGAGCCTTGG
GGTGGAATCATACGAGTTAGTTATCGAGGAAAGTGCTGTTTACGGATCAACACTGGATATCGAAATCGACGGTATCGAGGTCG
CCAGCGGCAGTTACGGCCCTCATGAGCTGGATGCGAACTGGGATATTTTTGATACCTGGGTCGGTATTGGTTTCGGGATTGAG
CGCTTGGCGATGGCAATCAATGGAGGTTCAACTATCAAAAAATACGGCCGTTCCATCAACTTCATCGATGGCGAGACTATGAA
GCTTTAA

Protein sequence of S^{Δ}-*Clos*PylRS^{QSW}:

MENFTITQTERLKQLNCENDVLELEFEDSEARNSKFREIEIGRVKKGKENIKNLLKEKHITISDEVGNKLSDWLMSKDYTKVL
TPTIISKDQLKAMTIDEENHLFSQVFWIDNNKCLRPMLQPNLYIVMRELKRITNEPVKIFEIGSCFRKESQGARHMNEFTMLS
MWELASVEDGKQLDTLKALAHEAMESLGVESYELVIEESAVYGSTLDIEIDGIEVASGSYGPHELDANWDIFDTWVGIGFGIE
RLAMAINGGSTIKKYGRSINFIDGETMKL*

DNA sequence of S^{Δ}-*Deb*PylRS^{QSW}:

ATGAACAGTTCTTGGACGGAGGTCCAGCGTCATCGCTTAAAAGAGTTGAACGGTGCAGAAAAAGACCTGGAAACAGCGTTCGG
TGACGATCTTCAACGCAACCGTGCCTTTCAAAAGTTAGAAAAACAGTTAGTGTACCAGGAGCGTAAACGCCTGGATCGCCTTC
TGGACACTCGTTTTCGTCCTCTTCGTTGTGAGTTAGAAAGCCTGTTAATCGACGCGCTTAAATGCGAGGGGTTTACACGTGTC
GAAACGCCCACTATCATTTCTCAGAATGACTTAGAGCGCATGTCAATCGACCGCTCCCACCCTTTTAACGATCAGGTTTACCG
TGTGGACTCAAAACACTGTTTACGTCCGATGTTACAGCCTGGATTATATCGTCTGATGAAAGATTTAGCCCGCATCCGCTCGG

GTAAGCCAGTTCGTATCTTCGAAATTGGTCCCTGCTTTCGTAAGGAAACGTCTGGCGCCCGTCACGCCGGAGAGTTTACGATG
CTTTCCCTGTGGGAGATGCGCATCGAAAAGGGGTCGCGTCGCTTTCGTATCGAGACTTTGGCCAAGCGTATCATGCACGCCGC
TGGTATTGACACCTATGATTTGGTCGATGAGCCATCCGAGGTGTACAATACAACACTTGACATTGTCTGTGGGTCCGACCCTC
TTGAAGTCGCCTCTTGCGCCATGGGTCCGCACCCCTTAGACGCAGCATGGGGTATCATTGATACCTGGGTAGGCCTTGGGTTT
GGGCTGGAGCGCTTGTTGATGGCCCGCGAAAATTCTCCGGGAATTGGAAAGTGGTGCAAGAGTGTATCCTACTTAGACGGCAT
CCGCCTTACCCTTTAA

Protein sequence of S^{Δ}-*Deb*PylRS^{QSW}:

MNSSWTEVQRHRLKELNGAEKDLETAFGDDLQRNRAFQKLEKQLVYQERKRLDRLLDTRFRPLRCELESLLIDALKCEGFTRV
ETPTIISQNDLERMSIDRSHPFNDQVYRVDSKHCLRPMLQPGLYRLMKDLARIRSGKPVRIFEIGPCFRKETSGARHAGEFTM
LSLWEMRIEKGSRRFRIETLAKRIMHAAGIDTYDLVDEPSEVYNTTLDIVCGSDPLEVASCAMGPHPLDAAWGIIDTWVGLGF
GLERLLMARENSPGIGKWCKSVSYLDGIRLTL*

DNA sequence of S^{Δ}-*Spi*PylRS^{QSW}:

ATGACAGCTCGCCACACAAACGATCCTCATACATTGCCAACACCCGGCCCCGCCCCAGGCTGGACCCCAGTCCAGCGTCAGCG
CCTTCGTCAATTAAATGTTCCTGAGCAAGAAGAAGAGCTGGTGTTTGCGAACCCCGAGGAGCGCGACCAGGCCTTTCGCGAAA
TTGAAAAACGTTTATCACAAGAACAACGCCGCCAATTAAAAGCGTTGCAAGGGTCTGTGGGCGAAGCCGCCCCAAGTCCACGT
ATTTTACGCCTTGAAGCGACCTTGGCCACCGGCTTACGCGGCGCCGGATTCACACAAGTTCGCACGCCAACTTTGTTAAGCCG
CCGTCTTTTAGCACGTATGGGCATTCGCGAAGGACATCCGATGTATGATCGTATGTTCTGGGTCGACGACCGTCACTGCCTTC
GTCCAATGCTGCAGCCCCACCTGTATTCTTTATTGGTTGATTTAGCACGCGTTTGGGATCGTCCGATTCGTTTATTCGAAGTC
GGGTCATGTTTCCGTCGCGAAACGCGCGGTTCACAGCATGCCGCTGAGTTTACTATGCTGTCCTTATGGGAAATGGGCCTGCC
AGGACCCTCTTGTCGTGAACGCCTGGAAACTCTGGCCCGTCTTGTGCTGCAAGAAGCTGGTATTCCAGAATACCGCATCGAAG
AGAAACGCTCAGCAGTGTACGGGACTACGATCGATCTGGTGGCAGGAGACTTAGAGTTAGGTTCGGCGGCCATTGGCCCACAT
AGCTTAGACCGCCCCTGGCGTATCACTGAAGCATGGGTGGGGATCGGATTTGGGCTTGAACGTCTTTTGATGGCCCGTGAGAA
CAGCGGGAGCTTAGGTCGCTGGAGCCGTTCTTTAGAATACTTGGACGGAATCACTTTACGTTTGTAA

Protein sequence of S^{Δ}-*Spi*PylRS^{QSW}:

MTARHTNDPHTLPTPGPAPGWTPVQRQRLRQLNVPEQEEELVFANPEERDQAFREIEKRLSQEQRRQLKALQGSVGEAAPSPR
ILRLEATLATGLRGAGFTQVRTPTLLSRRLLARMGIREGHPMYDRMFWVDDRHCLRPMLQPHLYSLLVDLARVWDRPIRLFEV
GSCFRRETRGSQHAAEFTMLSLWEMGLPGPSCRERLETLARLVLQEAGIPEYRIEEKRSAVYGTTIDLVAGDLELGSAAIGPH
SLDRPWRITEAWVGIGFGLERLLMARENSGSLGRWSRSLEYLDGITLRL*

DNA sequence of S^{Δ}-*Mic*PylRS^{QSW}:

ATGATTGGATGGTCCAAAGTACAGGAACAACGCCTTAAAGAATTAAACGCTGAGCTTTCTGATTTCGACCGTAAATTTTCAAA
CCAGAAAGATCGCGACCGTGCATTCCAGGAATTAGAACATCAATTAGTACAAAAGCAACGTTCCAACTTAGAAGCATTACGCG
AGTCACTTCCACGTCCCGACCTTATTAAGCTTACCGACACTCTTACCACTACGTTTATTCAGAACGGATTTTTACAGGTAGCC
ACACCACTGCTTATGTCAAAAGGCCATTTGCAGAAAATGGGAATTGATGAGACACATGATCTGTTTGAGCAAGTGTTTTGGAT
TGACAAGAAACGTTGCCTGCGTCCAATGCTGCAGCCTCATCTTTACTACGTAGTGCAAAATTTATTACGTTTATGGGAGCCTC
CAGTACGCATCTTTGAGATCGGCCCATGCTTCCGCAAGGAGACGAAGGGAGCGATTCACGCCGCCGAGTTCACCATGCTGTCC
GCATGGGAGTTCGGTTTGACCGAGGAACAACGCCAACCACGTTTAGAGGAAGTAATCGGGCGTGCAATGGCCGGGGCGGGCAT
TGATGATTATCAATTGGCGTACGAAGATTCCACAGTGTACGGACAGACCTTGGATATTGTCGTGGGTCCCGAACAAATGGAGG
TGGGCAGTGCGTCAATGGGGCCACATCCGTTGGACGCCGAGTGGGAAATTTTTACTCCTTGGATTGGGGTCGGTTTCGGTTTA
GAGCGCTTACTGATGTACACTCGCAATACTAACAATATCCGTAAGGTAGGCCGTTCTTTGAGCTATGTTCAGGGCATCCGCCT
TAACATCTAA

Protein sequence of S^{Δ}-*Mic*PylRS^{QSW}:

MIGWSKVQEQRLKELNAELSDFDRKFSNQKDRDRAFQELEHQLVQKQRSNLEALRESLPRPDLIKLTDTLTTTFIQNGFLQVA
TPLLMSKGHLQKMGIDETHDLFEQVFWIDKKRCLRPMLQPHLYYVVQNLLRLWEPPVRIFEIGPCFRKETKGAIHAAEFTMLS
AWEFGLTEEQRQPRLEEVIGRAMAGAGIDDYQLAYEDSTVYGQTLDIVVGPEQMEVGSASMGPHPLDAEWEIFTPWIGVGFGL
ERLLMYTRNTNNIRKVGRSLSYVQGIRLNI*

DNA sequence of S^{Δ}-*I2*PylRS^{QSW}:

ATGGGTATTATCTGGACTCCTATTCAAAAGCAGCGCTTGCAAGAGTTGAATGCTTCAGAAGCCCAGCGCGAGATGTGTTTCGA
GAGTCAGCAGGCTCGTGATCGCGCTTTTCAGGAGCAAGAACATAGTTTGGTAGTTGAGGGGAAACGCCGCTTGATGGAGTTGC
GTGACATCAAGCGCCGTCCCTCACTTTCGGTCTTGGAACAGCAATTGGTCGAGGCACTGACACAACAAGGGTTCGTACAAGTG
GTTACTCCAACGATTATCAGTAAAACGTCATTGGCTAAGATGTCTGTTTCAGACGACCACCCATTGTTCTCTCAAGTTTTCTG
GCTTGACAGCAAACGTTGTCTGCGCCCAATGTTACAGCCCAATCTGTATACCTTGTGGAAAGATTTGTTGCGTTTATGGGAAA
AGCCCATCCGCATCTTTGAGATTGGGACGTGCTATCGTAAGGAGAGTAAAGGTTCCTTGCATTTAAACGAGTTTACAATGTTG
TCCCTGTGGGAGTTGGGCCTGCCGGAGGACCAACGCCATCAACGCCTGGAGGAGTTGGCGTCACTGGTTATGGAAACTGTTGG
TATTGCGGATTACGAAATGGAGTTGACCACTAGTGTAGTCTATGGAGATACATTGGACGTCGTGAAAGGGATCGAATTAGGTT
CGTCTGCTATGGGGCCCCACCCATTGGACGACCAATGGGGGATCATTGATCCGTGGGTTGGGATTGGCTTCGGGCTGGAGCGC
CTGTTAATGATTAAAGAGGGTAGCCAGAACGTCCAGTCTATGGGCCGTTCGTTGACTTACTTAAACGGGGTCCGTCTTAACAT
CTAA

Protein sequence of S^{Δ}-*I2*PylRS^{QSW}:

MGIIWTPIQKQRLQELNASEAQREMCFESQQARDRAFQEQEHSLVVEGKRRLMELRDIKRRPSLSVLEQQLVEALTQQGFVQV
VTPTIISKTSLAKMSVSDDHPLFSQVFWLDSKRCLRPMLQPNLYTLWKDLLRLWEKPIRIFEIGTCYRKESKGSLHLNEFTML
SLWELGLPEDQRHQRLEELASLVMETVGIADYEMELTTSVVYGDTLDVVKGIELGSSAMGPHPLDDQWGIIDPWVGIGFGLER
LLMIKEGSQNVQSMGRSLTYLNGVRLNI*

DNA sequence of S^{Δ}-*Tron*PylRS^{QSW}:

ATGGAGTTCACTGTGACACAGAAACAGCGTCTTCAGGAATTGGGATTCGAAGGTGTGTTCCCTTCGGATTTCGAGGATGTAGA
TGAACGCAACCGCTTTTTCGAAGAGTTGGTTGGGCGTCTGCGTGACCGTAACCGTAAGCGTTTCGAGCGTCTTGTAGGGAACA
AAATTCCCTTTTGGCGCAAAGTATCCTCTGACCTTCGTAACCGCTTTTACGAATTAGGATTCGTTGAGGTGCGCACACCCGAA
ATTATTAGCTATAGTTTGCTGGAAAAGATGGAAATCAGCGATGACCTGCGTGAGCAAGTCTACTGGCTGGAAGAAGATAATCG
TTGTCTTCGTCCGATGCTTCAGCCAAATTTATATAACGAATTGCGCCATTTTAATCGTATTTCAAATCAGTCGAAGGTGCGCA
TTTTTGAGATCGGTACCTGCTTCCGTCGCGAGAAATCGTCATCGGAGCATTTGAATGAGTTTACTATGCTGTCCGCATGGGAA
ATGGGAGACATTGGTGATACGGAGGAGCGTCTTGACCGCCTTATTGAGGAAGTCTTTGGCGAATTCACAGACTATAAGAAAGT
CGGCGAGGAATCTTCCTTGTACGGTAAGACAGTAGACGTGTTGGTCGACGGGGTAGAAGTTGCCTCCTGTATTGCCGGCCCGC
ATCCACTTGATTCTAACTGGAGTATTGACCAGCCGTGGGTGGGCATTGGATTGGGTGTGGAGCGCCTGGCTATGCTGCTGGAT
GATGGCTCTACGGCTAAAGCATACGGGAATTCGTACATTTACCAGGATGGAGTACGTCTGGATATCAAGTAA

Protein sequence of S^{Δ}-*Tron*PylRS^{QSW}:

MEFTVTQKQRLQELGFEGVFPSDFEDVDERNRFFEELVGRLRDRNRKRFERLVGNKIPFWRKVSSDLRNRFYELGFVEVRTPE
IISYSLLEKMEISDDLREQVYWLEEDNRCLRPMLQPNLYNELRHFNRISNQSKVRIFEIGTCFRREKSSSEHLNEFTMLSAWE
MGDIGDTEERLDRLIEEVFGEFTDYKKVGEESSLYGKTVDVLVDGVEVASCIAGPHPLDSNWSIDQPWVGIGLGVERLAMLLD
DGSTAKAYGNSYIYQDGVRLDIK*

DNA sequence of S^{Δ}-*Nitra*PylRS^{QSW}:

ATGTCGAAGATCCGTTTCACCCGCGGACAAATCCACCGTTTGATTGAGCTGGGTGCCGAACCCACTGAACTGGAACGCGACTT
TGAAACTGAAGCGGAGCGCGATAAGGAATTTAACAAAATTGCCGAAAACCTTGCCCGCAAAAACTTAAAGAATATCAAAGACT
TTCTGGAGCAACGCCGTAAGCCTCTGGTACGTGTTATCGAAGAAAAACTTCGTACCACCGCCTTGCGTCTGGGATTTTCTGAA
GTCGTAACGCCAATTATTATCCCCCGTTTGTTCATTAAACGCATGGGTATTGATGAAGGGGATCCGCTTTGGAAGCAAGTTAT
GCTGATTGATGATAAACGTGCACTGCGCCCCATGTTGCAGCCTAACCTGTACGTCCTTATGGCAAAATTGTCAAACATCGTGC
GTCCGGTCAAAATTTTCGAAATCGGTCCTTGTTTTCGTCGTGAGACCGGAGGCCGTTACCACTTGGAGGAGTTCACCATGTTT
TCCATGTGGGAATTAGCACCGGAGGGGGACCCAAAGGAACGCCTGCTTGATTATATTGACACAATTATGCGCGATATTGGTCT
TAATTACACGATTTCCGTGGAACCATCAAATGTATATGGTGAGACCTTGGATGTAGTCGTTAATGGAATCGAAGTGGCGTCGG
CGGCAATCGGACCTAAACCGATTGATGCAAACTGGGGCGTGCGCGAGCCTTGGATCGGTGTGGGTTTTGGGGTCGAGCGCTTA
GCTATGCTTGTGGGGGGGTATAACTCTATTGCACGTATTGCCAAGAGCCTGTCGTATCTGGATGGCTCGACATTATCTGTGAT
CAAATTGCGCTGGTAA

Protein sequence of S^{Δ}-*Nitra*PylRS^{QSW}:

MSKIRFTRGQIHRLIELGAEPTELERDFETEAERDKEFNKIAENLARKNLKNIKDFLEQRRKPLVRVIEEKLRTTALRLGFSE
VVTPIIIPRLFIKRMGIDEGDPLWKQVMLIDDKRALRPMLQPNLYVLMAKLSNIVRPVKIFEIGPCFRRETGGRYHLEEFTMF
SMWELAPEGDPKERLLDYIDTIMRDIGLNYTISVEPSNVYGETLDVVVNGIEVASAAIGPKPIDANWGVREPWIGVGFGVERL
AMLVGGYNSIARIAKSLSYLDGSTLSVIKLRW*

DNA sequence of S^{Δ}-*1R26*PylRS^{QSW}:

ATGGCAGAACATTTTACAGATGCACAGATTCAGCGCTTACGCGAGTATGGTAATGGGACATATAAGGATATGGAGTTCGCAGA
CGTGAGTGCGCGTGAAAAGGCGTTCACGAAGCTTATGTCTGATGCCAGTCGCGATAATGAATCAGCCCTTAAGGGAATGATTG
CGCACCCTGCACGCCAGGGTTTATCACGCTTAATGAATGACATTGCAGACGCTTTGGTGGCTGATGGATTTATTGAAGTTCGC
ACACCCATTATTATCAGTAAAGATGCTTTAGCCAAAATGACAATCACTCCCGATAAACCATTATTCAAACAAGTATTTTGGAT
TGATGATAAACGCGCTCTGCGTCCAATGTTACAGCCGTCTCTGTACACGGTCATGCGTAGCCTGCGTGATCATACTGACGGCC
CTGTCAAAATTTTCGAAATGGGCTCTTGTTTTCGCAAAGAATCGCACAGTGGGATGCACCTGGAAGAGTTTACCATGTTATCC
CTGTGGGATATGGGCCCCGCCGGCGATGCCACTGAGTCCCTGAAGAAATATATTGGGATCGTAATGAAGGCCGCCGGGCTGCC
TGACTACCAATTAGTCCACGAGGAATCTGATGTGTATAAAGAAACGATTGATGTTGAAATCAACGGACAAGAGGTTTGTTCGG
CTGCTGTAGGTCCTCACTATTTGGATGCCGCCCATGACGTGCACGAGCCGTGGGCAGGGGCAGGGTTCGGCCTGGAGCGCCTG
CTGACAATTCGCCAGGGATATAGCACAGTGATGAAAGGGGGAGCTTCCACAACCTATTTGAACGGGGCTAAGATGGACTAA

Protein sequence of S^{Δ}-*1R26*PylRS^{QSW}:

MAEHFTDAQIQRLREYGNGTYKDMEFADVSAREKAFTKLMSDASRDNESALKGMIAHPARQGLSRLMNDIADALVADGFIEVR
TPIIISKDALAKMTITPDKPLFKQVFWIDDKRALRPMLQPSLYTVMRSLRDHTDGPVKIFEMGSCFRKESHSGMHLEEFTMLS
LWDMGPAGDATESLKKYIGIVMKAAGLPDYQLVHEESDVYKETIDVEINGQEVCSAAVGPHYLDAAHDVHEPWAGAGFGLERL
LTIRQGYSTVMKGGASTTYLNGAKMD*

DNA sequence of *Mm*tRNA$^{Pyl}_{CUA}$:

GGAAACCTGATCATGTAGATCGAATGGACTCTAAATCCGTTCAGCCGGGTTAGATTCCCGGGGTTTCCGCCA

DNA sequence of *Ms*tRNA$^{Pyl}_{CUA}$:

GGAAATCTGATCATGTAGATCGAATGGACTCTAAATCCGTTCAGCCGGGTTAGATTCCCGGGGTTTCCGCCA

DNA sequence of *Ma*tRNA$^{\Delta NPyl}$(8)$_{CUA}$:

GGGGGACGGTCCGGCGACCAGCGGGTCTCTAAAACCTAGCCTTGCGGGGTTCGACACCCCGGTCTCTCGCCA

DNA sequence of *G1*tRNA$^{\Delta NPyl}_{CUA}$:

GGAGGGCGCTCCGGCGAGCAAACGGGTCTCTAAAACCTGTAAGCGGGGTTCGACCCCCCGGCCTTTCGCCA

DNA sequence of 3C11-chPheT$_{CUA}$:

GTGAGAGTGATCATGTAGATCGAACGGACTCTAAATCCGTTCAGCCGGGTTAGATTCCCGGCTCTCACACCA

DNA sequence of *Tron*tRNA$^{Pyl}_{CUA}$:

GGGGGGCTGGTCGGGGTGACCACGGAGGCCCTATACCTCCCTTAGCCGGGTTCAACTCCCGGGTCCCTCGCCA

DNA sequence of *Int*tRNA$^{Pyl}_{CUA}$:

GGAGTGTTGGTCCGGGACCACCAGGCCTCTACAGCCACGGCAGCCGGGTTCAACTCCCGGGCACTTCGCCA

DNA sequence of *I2*tRNA$^{Pyl}_{CUA}$:

GGGGGGTAGATCGGATTGATCGCGTGGACTCTAAATCCGCGTAGACGGGTGAAACTCCCGTACTCCTCGCCA

DNA sequence of *Ma*RNA$^{Pyl}_{CUA}$:

GGGGGACGGTCCGGCGACCAGCGGGTCTCTAAAACCTAGCCAGCGGGGTTCGACGCCCCGGTCTCTCGCCA

DNA sequence of *Nitra*tRNA$^{Pyl}_{CUA}$:

GGGGGGCTGGTCGGGTGGCCAAGGGGGCTCTAAACCCTCGGTTGCCGGGTTCAACTCCCGGGCTCCCCACCA

V. Plasmid Construction

DNA sequence of pBAD-sfGFP150$_{TAG}$:

The pBAD-sfGFP150$_{TAG}$ plasmid contains a pBR322 type origin of replication, a kanamycin resistance gene (KanR), an araC element and an sfGFP150$_{TAG}$ under control of an araBAD promoter. Modified from Addgene #85483 (replacement of antibiotic resistance cassette)[16].

araBAD; sfGFP150$_{TAG}$-6xHis; KanR; pBR322 ori; araC.

AAGAAACCAATTGTCCATATTGCATCAGACATTGCCGTCACTGCGTCTTTTACTGGCTCTTCTCGCTAACCAAACCGGTAACC
CCGCTTATTAAAAGCATTCTGTAACAAAGCGGGACCAAAGCCATGACAAAAACGCGTAACAAAAGTGTCTATAATCACGGCAG
AAAAGTCCACATTGATTATTTGCACGGCGTCACACTTTGCTATGCCATAGCATTTTTATCCATAAGATTAGCGGATCCTACCT
GACGCTTTTTATCGCAACTCTCTACTGTTTCTCCATACCCGTTTTTTGGGCTAACAGGAGGAATTAACCATGGTTAGCAAAGG
TGAAGAACTGTTTACCGGCGTTGTGCCGATTCTGGTGGAACTGGATGGTGATGTGAATGGCCATAAATTTAGCGTTCGTGGCG
AAGGCGAAGGTGATGCGACCAACGGTAAACTGACCCTGAAATTTATTTGCACCACCGGTAAACTGCCGGTTCCGTGGCCGACC
CTGGTGACCACCCTGACCTATGGCGTTCAGTGCTTTAGCCGCTATCCGGATCATATGAAACGCCATGATTTCTTTAAAAGCGC
GATGCCGGAAGGCTATGTGCAGGAACGTACCATTAGCTTCAAAGATGATGGCACCTATAAAACCCGTGCGGAAGTTAAATTTG
AAGGCGATACCCTGGTGAACCGCATTGAACTGAAAGGTATTGATTTTAAAGAAGATGGCAACATTCTGGGTCATAAACTGGAA
TATAATTTCAACAGCCATTAGGTGTATATTACCGCCGATAAACAGAAAAATGGCATCAAAGCGAACTTTAAAATCCGTCACAA
CGTGGAAGATGGTAGCGTGCAGCTGGCGGATCATTATCAGCAGAATACCCCGATTGGTGATGGCCCGGTGCTGCTGCCGGATA
ATCATTATCTGAGCACCCAGAGCGTTCTGAGCAAAGATCCGAATGAAAAACGTGATCATATGGTGCTGCTGGAATTTGTTACC
GCCGCGGGCATTACCCACGGTATGGATGAACTGTATAAAGGCAGCCACCATCATCATCACCATTAAAGCTCGAGATCTGCAGC
TGGTACCATATGGGAATTCGAAGCTTGGCTGTTTTGGCGGATGAGAGAAGATTTTCAGCCTGATACAGATTAAATCAGAACGC
AGAAGCGGTCTGATAAAACAGAATTTGCCTGGCGGCAGTAGCGCGGTGGTCCCACCTGACCCCATGCCGAACTCAGAAGTGAA
ACGCCGTAGCGCCGATGGTAGTGTGGGGTCTCCCCATGCGAGAGTAGGGAACTGCCAGGCATCAAATAAAACGAAAGGCTCAG
TCGAAAGACTGGGCCTTTCGTTTTATCTGTTGTTTGTCGGTGAACGCTCTCCTGAGTAGGACAAATCCGCCGGGAGCGGATTT
GAACGTTGCGAAGCAACGGCCCGGAGGGTGGCGGGCAGGACGCCCGCCATAAACTGCCAGGCATCAAATTAAGCAGAAGGCCA
TCCTGACGGATGGCCTTTTTGCGTTTCTACAAACTCTTGTGCGCGGAACCCCTATTTGTTTATTTTTCTAAATACATTCAAAT
ATGTATCCGCTCATGAATTAATTCTTAGAAAAACTCATCGAGCATCAAATGAAACTGCAATTTATTCATATCAGGATTATCAA
TACCATATTTTTGAAAAAGCCGTTTCTGTAATGAAGGAGAAAACTCACCGAGGCAGTTCCATAGGATGGCAAGATCCTGGTAT
CGGTCTGCGATTCCGACTCGTCCAACATCAATACAACCTATTAATTTCCCCTCGTCAAAAATAAGGTTATCAAGTGAGAAATC
ACCATGAGTGACGACTGAATCCGGTGAGAATGGCAAAAGTTTATGCATTTCTTTCCAGACTTGTTCAACAGGCCAGCCATTAC
GCTCGTCATCAAAATCACTCGCATCAACCAAACCGTTATTCATTCGTGATTGCGCCTGAGCGAGACGAAATACGCGATCGCTG
TTAAAAGGACAATTACAAACAGGAATCGAATGCAACCGGCGCAGGAACACTGCCAGCGCATCAACAATATTTTCACCTGAATC
AGGATATTCTTCTAATACCTGGAATGCTGTTTTCCCGGGGATCGCAGTGGTGAGTAACCATGCATCATCAGGAGTACGGATAA
AATGCTTGATGGTCGGAAGAGGCATAAATTCCGTCAGCCAGTTTAGTCTGACCATCTCATCTGTAACATCATTGGCAACGCTA
CCTTTGCCATGTTTCAGAAACAACTCTGGCGCATCGGGCTTCCCATACAATCGATAGATTGTCGCACCTGATTGCCCGACATT
ATCGCGAGCCCATTTATACCCATATAAATCAGCATCCATGTTGGAATTTAATCGCGGCCTAGAGCAAGACGTTTCCCGTTGAA
TATGGCTCATAACACCCCTTGTATTACTGTTTATGTAAGCAGACAGTTTTATTGTTCATGACCAAAATCCCTTAACGTGAGTT
TTCGTTCCACTGAGCGTCAGACCCCGTAGAAAAGATCAAAGGATCTTCTTGAGATCCTTTTTTTCTGCGCGTAATCTGCTGCT
TGCAAACAAAAAAACCACCGCTACCAGCGGTGGTTTGTTTGCCGGATCAAGAGCTACCAACTCTTTTTCCGAAGGTAACTGGC
TTCAGCAGAGCGCAGATACCAAATACTGTCCTTCTAGTGTAGCCGTAGTTAGGCCACCACTTCAAGAACTCTGTAGCACCGCC
TACATACCTCGCTCTGCTAATCCTGTTACCAGTGGCTGCTGCCAGTGGCGATAAGTCGTGTCTTACCGGGTTGGACTCAAGAC
GATAGTTACCGGATAAGGCGCAGCGGTCGGGCTGAACGGGGGGTTCGTGCACACAGCCCAGCTTGGAGCGAACGACCTACACC
GAACTGAGATACCTACAGCGTGAGCTATGAGAAAGCGCCACGCTTCCCGAAGGGAGAAAGGCGGACAGGTATCCGGTAAGCGG
CAGGGTCGGAACAGGAGAGCGCACGAGGGAGCTTCCAGGGGGAAACGCCTGGTATCTTTATAGTCCTGTCGGGTTTCGCCACC
TCTGACTTGAGCGTCGATTTTTGTGATGCTCGTCAGGGGGGCGGAGCCTATGGAAAAACGCCAGCAACGCGGCCTTTTTACGG
TTCCTGGCCTTTTGCTGGCCTTTTGCTCACATGTTCTTTCCTGCGTTATCCCCTGATTCTGTGGATAACCGTATTACCGCCTT
TGAGTGAGCTGATACCGCTCGCCGCAGCCGAACGACCGAGCGCAGCGAGTCAGTGAGCGAGGAAGCGGAAGAGCGCCTGATGC
GGTATTTTCTCCTTACGCATCTGTGCGGTATTTCACACCGCATATGGTGCACTCTCAGTACAATCTGCTCTGATGCCGCATAG
TTAAGCCAGTATACACTCCGCTATCGCTACGTGACTGGGTCATGGCTGCGCCCCGACACCCGCCAACACCCGCTGACGCGCCC
TGACGGGCTTGTCTGCTCCCGGCATCCGCTTACAGACAAGCTGTGACCGTCTCCGGGAGCTGCATGTGTCAGAGGTTTTCACC
GTCATCACCGAAACGCGCGAGGCAGCAGATCAATTCGCGCGCGAAGGCGAAGCGGCATGCATAATGTGCCTGTCAAATGGACG
AAGCAGGGATTCTGCAAACCCTATGCTACTCCGTCAAGCCGTCAATTGTCTGATTCGTTACCAATTATGACAACTTGACGGCT
ACATCATTCACTTTTTCTTCACAACCGGCACGGAACTCGCTCGGGCTGGCCCCGGTGCATTTTTTAAATACCCGCGAGAAATA
GAGTTGATCGTCAAAACCAACATTGCGACCGACGGTGGCGATAGGCATCCGGGTGGTGCTCAAAAGCAGCTTCGCCTGGCTGA
TACGTTGGTCCTCGCGCCAGCTTAAGACGCTAATCCCTAACTGCTGGCGGAAAAGATGTGACAGACGCGACGGCGACAAGCAA
ACATGCTGTGCGACGCTGGCGATATCAAAATTGCTGTCTGCCAGGTGATCGCTGATGTACTGACAAGCCTCGCGTACCCGATT
ATCCATCGGTGGATGGAGCGACTCGTTAATCGCTTCCATGCGCCGCAGTAACAATTGCTCAAGCAGATTTATCGCCAGCAGCT
CCGAATAGCGCCCTTCCCCTTGCCCGGCGTTAATGATTTGCCCAAACAGGTCGCTGAAATGCGGCTGGTGCGCTTCATCCGGG
CGAAAGAACCCCGTATTGGCAAATATTGACGGCCAGTTAAGCCATTCATGCCAGTAGGCGCGCGGACGAAAGTAAACCCACTG
GTGATACCATTCGCGAGCCTCCGGATGACGACCGTAGTGATGAATCTCTCCTGGCGGGAACAGCAAAATATCACCCGGTCGGC
AAACAATTCTCGTCCCTGATTTTTCACCACCCCCTGACCGCGAATGGTGAGATTGAGAATATAACCTTTCATTCCCAGCGGT

CGGTCGATAAAAAAATCGAGATAACCGTTGGCCTCAATCGGCGTTAAACCCGCCACCAGATGGGCATTAAACGAGTATCCCGG
CAGCAGGGGATCATTTTGCGCTTCAGCCATACTTTTCATACTCCCGCCATTCAGAG

pDPS2-*Mm*tRNA$^{Pyl}_{CUA}$ plasmid:

The pDPS2-*Mm*tRNA$^{Pyl}_{CUA}$ plasmid contains a p15a origin of replication, a tetracycline resistance gene (TetR), an sfGFP gene containing an amber mutation at position 150 under control of an L-arabinose inducible araBAD promoter, a chloramphenicol resistance gene with an amber mutation at position 111 and an MmtRNA$^{Pyl}_{CUA}$ tRNA flanked by aproK promoter and terminator.

TetR; *Mm*tRNA$^{Pyl}_{CUA}$; cat-promoter/cat111TAG; p15A ori; araC; araBAD; sfGFP150$_{TAG}$-6xHis.

GAGAGAAGATTTTCAGCCTGATACAGATTAAATCAGAACGCAGAAGCGGTCTGATAAAACAGAATTTGCCTGGCGGCAGTAGC
GCGGTGGTCCCACCTGACCCCATGCCGAACTCAGAAGTGAAACGCCGTAGCGCCGATGGTAGTGTAGCACCTGAAGTCAGCCC
CATACGATATAAGTTGTAATTCTCATGTTTGACAGCTTATCATCGATAAGCTTTAATGCGGTAGTTTATCACAGTTAAATTGC
TAACGCAGTCAGGCACCGTGTATGAAATCTAACAATGCGCTCATCGTCATCCTCGGCACCGTCACCCTGGATGCTGTAGGCAT
AGGCTTGGTTATGCCGGTACTGCCGGGCCTCTTGCGGGATATCGTCCATTCCGACAGCATCGCCAGTCACTATGGCGTGCTGC
TAGCGCTATATGCGTTGATGCAATTTCTATGCGCACCCGTTCTCGGAGCACTGTCCGACCGCTTTGGCCGCCGCCCAGTCCTG
CTCGCTTCGCTACTTGGAGCCACTATCGACTACGCGATCATGGCGACCACACCCGTCCTGTGGATCCTCTACGCCGGACGCAT
CGTGGCCGGCATCACCGGCGCCACAGGTGCGGTTGCTGGCGCCTATATCGCCGACATCACCGATGGGGAAGATCGGGCTCGCC
ACTTCGGGCTCATGAGCGCTTGTTTCGGCGTGGGTATGGTGGCAGGCCCCGTGGCCGGGGGACTGTTGGGCGCCATCTCCTTG
CATGCACCATTCCTTGCGGCGGCGGTGCTCAACGGCCTCAACCTACTACTGGGCTGCTTCCTAATGCAGGAGTCGCATAAGGG
AGAGCGTCGACCGATGCCCTTGAGAGCCTTCAACCCAGTCAGCTCCTTCCGGTGGGCGCGGGGCATGACTATCGTCGCCGCAC
TTATGACTGTCTTCTTTATCATGCAACTCGTAGGACAGGTGCCGGCAGCGCTCTGGGTCATTTTCGGCGAGGACCGCTTTCGC
TGGAGCGCGACGATGATCGGCCTGTCGCTTGCGGTATTCGGAATCTTGCACGCCCTCGCTCAAGCCTTCGTCACTGGTCCCGC
CACCAAACGTTTCGGCGAGAAGCAGGCCATTATCGCCGGCATGGCGGCCGACGCGCTGGGCTACGTCTTGCTGGCGTTCGCGA
CGCGAGGCTGGATGGCCTTCCCCATTATGATTCTTCTCGCTTCCGGCGGCATCGGGATGCCCGCGTTGCAGGCCATGCTGTCC
AGGCAGGTAGATGACGACCATCAGGGACAGCTTCAAGGATCGCTCGCGGCTCTTACCAGCCTAACTTCGATCATTGGACCGCT
GATCGTCACGGCGATTTATGCCGCCTCGGCGAGCACATGGAACGGGTTGGCATGGATTGTAGGCGCCGCCCTATACCTTGTCT
GCCTCCCCGCGTTGCGTCGCGGTGCATGGAGCCGGGCCACCTCGACCTAATAAAACGAAAGGCTCAGTCGAAAGACTGGGCCT
TGTTTGTGAGCTCCCGGTCATCAATCATCCCCATAATCCTTGTTAGATTATCAATTTTAAAAAACTAACAGTTGTCAGCCTGT
CCCGCTTTAATATCATACGCCGTTATACGTTGTTTACGCTTTGAGGAATCCCATATGATACGCGGCCGCTTTCAAACGCTAAA
TTGCCTGATGCGCTACGCTTATCAGGCCTACATGATCTCTGCAATATATTGAGTTTGCGTGCTTTTGTAGGCCGGATAAGGCG
TTCACGCCGCATCCGGCAAGAAACAGCAAACAATCCAAAACGCCGCGTTCAGCGGCGTTTTTTCTGCTTTTCTTCGCGAATTA
ATTCCGCTTCGCAACATGTGAGCACCGGTTTATTGACTACCGGAAGCAGTGTGACCGTGTGCTTCTCAAATGCCTGAGGCCAG
TTTGCTCAGGCTCTCCCCGTGGAGGTAATAATTGACGATATGATCAGTGCACGGCTAACTAAGCGGCCTGCTGACTTTCTCGC
CGATCAAAAGGCATTTTGCTATTAAGGGATTGACGAGGGCGTATCTGCGCAGTAAGATGCGCCCCGCATTGGAAACCTGATCA
TGTAGATCGAATGGACTCTAAATCCGTTCAGCCGGGTTAGATTCCCGGGGTTTCCGCCAAATTCGAAAAGCCTGCTCAACGAG
CAGGCTTTTTTGCATGCTCGAGCAGCTCAGGGTCGAATTTGCTTTCGAATTTCTGCCATTCATCCGCTTATTATCACTTATTC
AGGCGTAGCAACCAGGCGTTTAAGGGCACCAATAACTGCCTTAAAAAAATTACGCCCCGCCCTGCCACTCATCGCAGTACTGT
TGTAATTCATTAAGCATTCTGCCGACATGGAAGCCATCACAAACGGCATGATGAACCTGAATCGCCAGCGGCATCAGCACCTT
GTCGCCTTGCGTATAATATTTGCCCATGGTGAAAACGGGGGCGAAGAAGTTGTCCATATTGGCCACGTTTAAATCAAAACTGG
TGAAACTCACCCAGGGATTGGCTGAGACGAAAAACATATTCTCAATAAACCCTTTAGGGAAATAGGCCAGGTTTTCACCGTAA
CACGCCACATCTTGCGAATATATGTGTAGAAACTGCCGGAAATCCTAGTGGTATTCACTCCAGAGCGATGAAAACGTTTCAGT
TTGCTCATGGAAAACGGTGTAACAAGGGTGAACACTATCCCATATCACCAGCTCACCGTCTTTCATTGCCATACGGAATTCCG
GATGAGCATTCATCAGGCGGGCAAGAATGTGAATAAAGGCCGGATAAAACTTGTGCTTATTTTTCTTTACGGTCTTTAAAAAG
GCCGTAATATCCAGCTGAACGGTCTGGTTATAGGTACATTGAGCAACTGACTGAAATGCCTCAAAATGTTCTTTACGATGCCA
TTGGGATATATCAACGGTGGTATATCCAGTGATTTTTTTCTCCATTTTAGCTTCCTTAGCTCCTGAAAATCTCGATAACTCAA
AAAATACGCCCGGTAGTGATCTTATTTCATTATGGTGAAAGTTGGAACCTCTTACGTGCCGATCAACGTCTCATTTTCGCCAA
AAGTTGGCCCAGGGCTTCCCGGTATCAACAGGGACACCAGGATTTATTTATTCTGCGAAGTGATCTTCCGTCACAGGTATTTA
TTCGGCGCAAAGTGCGTCGGGTGATGCTGCCAACTTACTGATTTAGTGTATGATGGTGTTTTTGAGGTGCTCCAGTGGCTTCT
GTTTCTATCAGCTGTCCCTCCTGTTCAGCTACTGACGGGGTGGTGCGTAACGGCAAAAGCACCGCCGGACATCAGCGCTAGCG
GAGTGTATACTGGCTTACTATGTTGGCACTGATGAGGGTGTCAGTGAAGTGCTTCATGTGGCAGGAGAAAAAAGGCTGCACCG
GTGCGTCAGCAGAATATGTGATACAGGATATATTCCGCTTCCTCGCTCACTGACTCGCTACGCTCGGTCGTTCGACTGCGGCG
AGCGGAAATGGCTTACGAACGGGGCGGAGATTTCCTGGAAGATGCCAGGAAGATACTTAACAGGGAAGTGAGAGGGCCGCGGC
AAAGCCGTTTTTCCATAGGCTCCGCCCCCCTGACAAGCATCACGAAATCTGACGCTCAAATCAGTGGTGGCGAAACCCGACAG
GACTATAAAGATACCAGGCGTTTCCCCCTGGCGGCTCCCTCGTGCGCTCTCCTGTTCCTGCCTTTCGGTTTACCGGTGTCATT
CCGCTGTTATGGCCGCGTTTGTCTCATTCCACGCCTGACACTCAGTTCCGGGTAGGCAGTTCGCTCCAAGCTGGACTGTATGC
ACGAACCCCCCGTTCAGTCCGACCGCTGCGCCTTATCCGGTAACTATCGTCTTGAGTCCAACCCGGAAAGACATGCAAAAGCA
CCACTGGCAGCAGCCACTGGTAATTGATTTAGAGGAGTTAGTCTTGAAGTCATGCGCCGGTTAAGGCTAAACTGAAAGGACAA
GTTTTGGTGACTGCGCTCCTCCAAGCCAGTTACCTCGGTTCAAAGAGTTGGTAGCTCAGAGAACCTTCGAAAAACCGCCCTGC
AAGGCGGTTTTTTCGTTTTCAGAGCAAGAGATTACGCGCAGACCAAAACGATCTCAAGAAGATCATCTTATTAATCAGATAAA
ATATTTCTAGATTTCAGTGCAATTTATCTCTTCAAATGTAGCACCTGAAGTCAGCCCCATACGATATAAGTTGTAATTCTCAT
GTTTGACAGCTTATCATCGATAAGCTTGGTACCCAATTATGACAACTTGACGGCTACATCATTCACTTTTTCTTCACAACCGG

CACGGAACTCGCTCGGGCTGGCCCCGGTGCATTTTTTAAATACCCGCGAGAAATAGAGTTGATCGTCAAAACCAACATTGCGA
CCGACGGTGGCGATAGGCATCCGGGTGGTGCTCAAAAGCAGCTTCGCCTGGCTGATACGTTGGTCCTCGCGCCAGCTTAAGAC
GCTAATCCCTAACTGCTGGCGGAAAAGATGTGACAGACGCGACGGCGACAAGCAAACATGCTGTGCGACGCTGGCGATATCAA
AATTGCTGTCTGCCAGGTGATCGCTGATGTACTGACAAGCCTCGCGTACCCGATTATCCATCGGTGGATGGAGCGACTCGTTA
ATCGCTTCCATGCGCCGCAGTAACAATTGCTCAAGCAGATTTATCGCCAGCAGCTCCGAATAGCGCCCTTCCCCTTGCCCGGC
GTTAATGATTTGCCCAAACAGGTCGCTGAAATGCGGCTGGTGCGCTTCATCCGGGCGAAAGAACCCCGTATTGGCAAATATTG
ACGGCCAGTTAAGCCATTCATGCCAGTAGGCGCGCGGACGAAAGTAAACCCACTGGTGATACCATTCGCGAGCCTCCGGATGA
CGACCGTAGTGATGAATCTCTCCTGGCGGGAACAGCAAAATATCACCCGGTCGGCAAACAAATTCTCGTCCCTGATTTTTCAC
CACCCCCTGACCGCGAATGGTGAGATTGAGAATATAACCTTTCATTCCCAGCGGTCGGTCGATAAAAAAATCGAGATAACCGT
TGGCCTCAATCGGCGTTAAACCCGCCACCAGATGGGCATTAAACGAGTATCCCGGCAGCAGGGGATCATTTTGCGCTTCAGCC
ATACTTTTCATACTCCCGCCATTCAGAG**AAGAAACCAATTGTCCATATTGCATCAGACATTGCCGTCACTGCGTCTTTTACTG**
GCTCTTCTCGCTAACCAAACCGGTAACCCCGCTTATTAAAAGCATTCTGTAACAAAGCGGGACCAAAGCCATGACAAAAACGC
GTAACAAAAGTGTCTATAATCACGGCAGAAAAGTCCACATTGATTATTTGCACGGCGTCACACTTTGCTATGCCATAGCATTT
TTATCCATAAGATTAGCGGATCCTACCTGACGCTTTTTATCGCAACTCTCTACTGTTTCTCCATACCCGTTTTTTGGGCTAAC
AGGAGGAATTAACC**ATGGTTAGCAAAGGTGAAGAACTGTTTACCGGCGTTGTGCCGATTCTGGTGGAACTGGATGGTGATGTG**
AATGGCCATAAATTTAGCGTTCGTGGCGAAGGCGAAGGTGATGCGACCAACGGTAAACTGACCCTGAAATTTATTTGCACCAC
CGGTAAACTGCCGGTTCCGTGGCCGACCCTGGTGACCACCCTGACCTATGGCGTTCAGTGCTTTAGCCGCTATCCGGATCATA
TGAAACGCCATGATTTCTTTAAAAGCGCGATGCCGGAAGGCTATGTGCAGGAACGTACCATTAGCTTCAAAGATGATGGCACC
TATAAAACCCGTGCGGAAGTTAAATTTGAAGGCGATACCCTGGTGAACCGCATTGAACTGAAAGGTATTGATTTTAAAGAAGA
TGGCAACATTCTGGGTCATAAACTGGAATATAATTTCAACAGCCATTAGGTGTATATTACCGCCGATAAACAGAAAAATGGCA
TCAAAGCGAACTTTAAAATCCGTCACAACGTGGAAGATGGTAGCGTGCAGCTGGCGGATCATTATCAGCAGAATACCCCGATT
GGTGATGGCCCGGTGCTGCTGCCGGATAATCATTATCTGAGCACCCAGAGCGTTCTGAGCAAAGATCCGAATGAAAAACGTGA
TCATATGGTGCTGCTGGAATTTGTTACCGCCGCGGGCATTACCCACGGTATGGATGAACTGTATAAAGGCAGCCACCATCATC
ATCACCATTAAAGCTCGAGCGAAGCTTGGGCCCGAACAAAAACTCATCTCAGAAGAGGATCTGAATAGCGCCGTCGACCATCA
TCATCATCATCATTGAGTTTAAACGGTCTCCAGCTTGGCTGTTTTGGCGGATGAGAGAAGATTTTCAGCCTGATACAGATTAA
ATCAGAACGCAGAAGCGGTCTGATAAAACAGAATTTGCCTGGCGGCAGTAGCGCGGTGGTCCCACCTGACCCCATGCCGAACT
CAGAAGTGAAACGCCGTAGCGCCGATGGTAGTGTGGGGTCTCCCCATGCGAGAGTAGGGAACTGCCAGGCATCAAATAAAACG
AAAGGCTCAGTCGAAAGACTGGGCCTTTCGTTTTATCTGTTGTTTGTCGGTGAACGCTCTCCTGAGTAGGACAAATCCTGGCT
GTTTTGGCGGAT

pDPS2-3C11-chPheT$_{CUA}$ plasmid:

The pDPS2-3C11-chPheT$_{CUA}$ plasmid contains a p15a origin of replication, a tetracycline resistance gene (TetR), an sfGFP gene containing an amber mutation at position 150 under control of an L-arabinose inducible araBAD promoter, a chloramphenicol resistance gene with an amber mutation at position 111 and a 3C11-chPheT$_{CUA}$ tRNA flanked by a proK promoter and terminator.

TetR; 3C11-chPheT$_{CUA}$; cat-promoter/cat111TAG; p15A ori; araC; araBAD; sfGFP150$_{TAG}$-6xHis.

GAGAGAAGATTTTCAGCCTGATACAGATTAAATCAGAACGCAGAAGCGGTCTGATAAAACAGAATTTGCCTGGCGGCAGTAGC
GCGGTGGTCCCACCTGACCCCATGCCGAACTCAGAAGTGAAACGCCGTAGCGCCGATGGTAGTGTAGCACCTGAAGTCAGCCC
CATACGATATAAGTTGTAATTCTCATGT**TTGACAGCTTATCATCGATAAGCTTTAATGCGGTAGTTTATCACAGTTAAATTGC**
TAACGCAGTCAGGCACCGTGTATGAAATCTAACAATGCGCTCATCGTCATCCTCGGCACCGTCACCCTGGATGCTGTAGGCAT
AGGCTTGGTTATGCCGGTACTGCCGGGCCTCTTGCGGGATATCGTCCATTCCGACAGCATCGCCAGTCACTATGGCGTGCTGC
TAGCGCTATATGCGTTGATGCAATTTCTATGCGCACCCGTTCTCGGAGCACTGTCCGACCGCTTTGGCCGCCGCCCAGTCCTG
CTCGCTTCGCTACTTGGAGCCACTATCGACTACGCGATCATGGCGACCACACCCGTCCTGTGGATCCTCTACGCCGGACGCAT
CGTGGCCGGCATCACCGGCGCCACAGGTGCGGTTGCTGGCGCCTATATCGCCGACATCACCGATGGGGAAGATCGGGCTCGCC
ACTTCGGGCTCATGAGCGCTTGTTTCGGCGTGGGTATGGTGGCAGGCCCCGTGGCCGGGGGACTGTTGGGCGCCATCTCCTTG
CATGCACCATTCCTTGCGGCGGCGGTGCTCAACGGCCTCAACCTACTACTGGGCTGCTTCCTAATGCAGGAGTCGCATAAGGG
AGAGCGTCGACCGATGCCCTTGAGAGCCTTCAACCCAGTCAGCTCCTTCCGGTGGGCGCGGGGCATGACTATCGTCGCCGCAC
TTATGACTGTCTTCTTTATCATGCAACTCGTAGGACAGGTGCCGGCAGCGCTCTGGGTCATTTTCGGCGAGGACCGCTTTCGC
TGGAGCGCGACGATGATCGGCCTGTCGCTTGCGGTATTCGGAATCTTGCACGCCCTCGCTCAAGCCTTCGTCACTGGTCCCGC
CACCAAACGTTTCGGCGAGAAGCAGGCCATTATCGCCGGCATGGCGGCCGACGCGCTGGGCTACGTCTTGCTGGCGTTCGCGA
CGCGAGGCTGGATGGCCTTCCCCATTATGATTCTTCTCGCTTCCGGCGGCATCGGGATGCCCGCGTTGCAGGCCATGCTGTCC
AGGCAGGTAGATGACGACCATCAGGGACAGCTTCAAGGATCGCTCGCGGCTCTTACCAGCCTAACTTCGATCATTGGACCGCT
GATCGTCACGGCGATTTATGCCGCCTCGGCGAGCACATGGAACGGGTTGGCATGGATTGTAGGCGCCGCCCTATACCTTGTCT
GCCTCCCCGCGTTGCGTCGCGGTGCATGGAGCCGGGCCACCTCGACCTAATAAAACGAAAGGCTCAGTCGAAAGACTGGGCCT
TGTTTGTGAGCTCCCGGTCATCAATCATCCCCATAATCCTTGTTAGATTATCAATTTTAAAAACTAACAGTTGTCAGCCTGT
CCCGCTTTAATATCATACGCCGTTATACGTTGTTTACGCTTTGAGGAATCCCATATGATACGCGGCCGCTTTCAAACGCTAAA
TTGCCTGATGCGCTACGCTTATCAGGCCTACATGATCTCTGCAATATATTGAGTTTGCGTGCTTTTGTAGGCCGGATAAGGCG
TTCACGCCGCATCCGGCAAGAAACAGCAAACAATCCAAAACGCCGCGTTCAGCGGCGTTTTTTCTGCTTTTCTTCGCGAATTA
ATTCCGCTTCGCAACATGTGAGCACCGGTTTATTGACTACCGGAAGCAGTGTGACCGTGTGCTTCTCAAATGCCTGAGGCCAG
TTTGCTCAGGCTCTCCCCGTGGAGGTAATAATTGACGATATGATCAGTGCACGGCTAACTAAGCGGCCTGCTGACTTTCTCGC
CGATCAAAAGGCATTTTGCTATTAAGGGATTGACGAGGGCGTATCTGCGCAGTAAGATGCGCCCCGCATT**GTGAGAGTGATCA**
TGTAGATCGAACGGACTCTAAATCCGTTCAGCCGGGTTAGATTCCCGGCTCTCACACCAAATTCGAAAAGCCTGCTCAACGAG
CAGGCTTTTTTGCATGCTCGAGCAGCTCAGGGTCGAATTTGCTTTCGAATTTCTGCCATTCATCCGCTTATTATCACTTATTC

AGGCGTAGCAACCAGGCGTTTAAGGGCACCAATAACTGCCTTAAAAAAATTACGCCCCGCCCTGCCACTCATCGCAGTACTGT
TGTAATTCATTAAGCATTCTGCCGACATGGAAGCCATCACAAACGGCATGATGAACCTGAATCGCCAGCGGCATCAGCACCTT
GTCGCCTTGCGTATAATATTTGCCCATGGTGAAAACGGGGGCGAAGAAGTTGTCCATATTGGCCACGTTTAAATCAAAACTGG
TGAAACTCACCCAGGGATTGGCTGAGACGAAAAACATATTCTCAATAAACCCTTTAGGGAAATAGGCCAGGTTTTCACCGTAA
CACGCCACATCTTGCGAATATATGTGTAGAAACTGCCGGAAATCCTAGTGGTATTCACTCCAGAGCGATGAAAACGTTTCAGT
TTGCTCATGGAAAACGGTGTAACAAGGGTGAACACTATCCCATATCACCAGCTCACCGTCTTTCATTGCCATACGGAATTCCG
GATGAGCATTCATCAGGCGGGCAAGAATGTGAATAAAGGCCGGATAAAACTTGTGCTTATTTTTCTTTACGGTCTTTAAAAAG
GCCGTAATATCCAGCTGAACGGTCTGGTTATAGGTACATTGAGCAACTGACTGAAATGCCTCAAAATGTTCTTTACGATGCCA
TTGGGATATATCAACGGTGGTATATCCAGTGATTTTTTTCTCCATTTTAGCTTCCTTAGCTCCTGAAAATCTCGATAACTCAA
AAAATACGCCCGGTAGTGATCTTATTTCATTATGGTGAAAGTTGGAACCTCTTACGTGCCGATCAACGTCTCATTTTCGCCAA
AAGTTGGCCCAGGGCTTCCCGGTATCAACAGGGACACCAGGATTTATTTATTCTGCGAAGTGATCTTCCGTCACAGGTATTTA
TTCGGCGCAAAGTGCGTCGGGTGATGCTGCCAACTTACTGATTTAGTGTATGATGGTGTTTTTGAGGTGCTCCAGTGGCTTCT
GTTTCTATCAGCTGTCCCTCCTGTTCAGCTACTGACGGGGTGGTGCGTAACGGCAAAAGCACCGCCGGACATCAGCGCTAGCG
GAGTGTATACTGGCTTACTATGTTGGCACTGATGAGGGTGTCAGTGAAGTGCTTCATGTGGCAGGAGAAAAAAGGCTGCACCG
GTGCGTCAGCAGAATATGTGATACAGGATATATTCCGCTTCCTCGCTCACTGACTCGCTACGCTCGGTCGTTCGACTGCGGCG
AGCGGAAATGGCTTACGAACGGGGCGGAGATTTCCTGGAAGATGCCAGGAAGATACTTAACAGGGAAGTGAGAGGGCCGCGGC
AAAGCCGTTTTTCCATAGGCTCCGCCCCCCTGACAAGCATCACGAAATCTGACGCTCAAATCAGTGGTGGCGAAACCCGACAG
GACTATAAAGATACCAGGCGTTTCCCCCTGGCGGCTCCCTCGTGCGCTCTCCTGTTCCTGCCTTTCGGTTTACCGGTGTCATT
CCGCTGTTATGGCCGCGTTTGTCTCATTCCACGCCTGACACTCAGTTCCGGGTAGGCAGTTCGCTCCAAGCTGGACTGTATGC
ACGAACCCCCCGTTCAGTCCGACCGCTGCGCCTTATCCGGTAACTATCGTCTTGAGTCCAACCCGGAAAGACATGCAAAAGCA
CCACTGGCAGCAGCCACTGGTAATTGATTTAGAGGAGTTAGTCTTGAAGTCATGCGCCGGTTAAGGCTAAACTGAAAGGACAA
GTTTTGGTGACTGCGCTCCTCCAAGCCAGTTACCTCGGTTCAAAGAGTTGGTAGCTCAGAGAACCTTCGAAAAACCGCCCTGC
AAGGCGGTTTTTTCGTTTTCAGAGCAAGAGATTACGCGCAGACCAAAACGATCTCAAGAAGATCATCTTATTAATCAGATAAA
ATATTTCTAGATTTCAGTGCAATTTATCTCTTCAAATGTAGCACCTGAAGTCAGCCCCATACGATATAAGTTGTAATTCTCAT
GTTTGACAGCTTATCATCGATAAGCTTGGTACCCAATTATGACAACTTGACGGCTACATCATTCACTTTTTCTTCACAACCGG
CACGGAACTCGCTCGGGCTGGCCCCGGTGCATTTTTTAAATACCCGCGAGAAATAGAGTTGATCGTCAAAACCAACATTGCGA
CCGACGGTGGCGATAGGCATCCGGGTGGTGCTCAAAAGCAGCTTCGCCTGGCTGATACGTTGGTCCTCGCGCCAGCTTAAGAC
GCTAATCCCTAACTGCTGGCGGAAAAGATGTGACAGACGCGACGGCGACAAGCAAACATGCTGTGCGACGCTGGCGATATCAA
AATTGCTGTCTGCCAGGTGATCGCTGATGTACTGACAAGCCTCGCGTACCCGATTATCCATCGGTGGATGGAGCGACTCGTTA
ATCGCTTCCATGCGCCGCAGTAACAATTGCTCAAGCAGATTTATCGCCAGCAGCTCCGAATAGCGCCCTTCCCCTTGCCCGGC
GTTAATGATTTGCCCAAACAGGTCGCTGAAATGCGGCTGGTGCGCTTCATCCGGGCGAAAGAACCCCGTATTGGCAAATATTG
ACGGCCAGTTAAGCCATTCATGCCAGTAGGCGCGCGGACGAAAGTAAACCCACTGGTGATACCATTCGCGAGCCTCCGGATGA
CGACCGTAGTGATGAATCTCTCCTGGCGGGAACAGCAAAATATCACCCGGTCGGCAAACAAATTCTCGTCCCTGATTTTTCAC
CACCCCCTGACCGCGAATGGTGAGATTGAGAATATAACCTTTCATTCCCAGCGGTCGGTCGATAAAAAAATCGAGATAACCGT
TGGCCTCAATCGGCGTTAAACCCGCCACCAGATGGGCATTAAACGAGTATCCCGGCAGCAGGGGATCATTTTGCGCTTCAGCC
ATACTTTTCATACTCCCGCCATTCAGAG**AAGAAACCAATTGTCCATATTGCATCAGACATTGCCGTCACTGCGTCTTTTACTG**
GCTCTTCTCGCTAACCAAACCGGTAACCCCGCTTATTAAAAGCATTCTGTAACAAAGCGGGACCAAAGCCATGACAAAAACGC
GTAACAAAAGTGTCTATAATCACGGCAGAAAAGTCCACATTGATTATTTGCACGGCGTCACACTTTGCTATGCCATAGCATTT
TTATCCATAAGATTAGCGGATCCTACCTGACGCTTTTTATCGCAACTCTCTACTGTTTCTCCATACCCGTTTTTTGGGCTAAC
AGGAGGAATTAACC**ATGGTTAGCAAAGGTGAAGAACTGTTTACCGGCGTTGTGCCGATTCTGGTGGAACTGGATGGTGATGTG**
AATGGCCATAAATTTAGCGTTCGTGGCGAAGGCGAAGGTGATGCGACCAACGGTAAACTGACCCTGAAATTTATTTGCACCAC
CGGTAAACTGCCGGTTCCGTGGCCGACCCTGGTGACCACCCTGACCTATGGCGTTCAGTGCTTTAGCCGCTATCCGGATCATA
TGAAACGCCATGATTTCTTTAAAAGCGCGATGCCGGAAGGCTATGTGCAGGAACGTACCATTAGCTTCAAAGATGATGGCACC
TATAAAACCCGTGCGGAAGTTAAATTTGAAGGCGATACCCTGGTGAACCGCATTGAACTGAAAGGTATTGATTTTAAAGAAGA
TGGCAACATTCTGGGTCATAAACTGGAATATAATTTCAACAGCCATTAGGTGTATATTACCGCCGATAAACAGAAAAATGGCA
TCAAAGCGAACTTTAAAATCCGTCACAACGTGGAAGATGGTAGCGTGCAGCTGGCGGATCATTATCAGCAGAATACCCCGATT
GGTGATGGCCCGGTGCTGCTGCCGGATAATCATTATCTGAGCACCCAGAGCGTTCTGAGCAAAGATCCGAATGAAAAACGTGA
TCATATGGTGCTGCTGGAATTTGTTACCGCCGCGGGCATTACCCACGGTATGGATGAACTGTATAAAGGCAGCCACCATCATC
ATCACCATTAAAGCTCGAGCGAAGCTTGGGCCCGAACAAAAACTCATCTCAGAAGAGGATCTGAATAGCGCCGTCGACCATCA
TCATCATCATCATTGAGTTTAAACGGTCTCCAGCTTGGCTGTTTTGGCGGATGAGAGAAGATTTTCAGCCTGATACAGATTAA
ATCAGAACGCAGAAGCGGTCTGATAAAACAGAATTTGCCTGGCGGCAGTAGCGCGGTGGTCCCACCTGACCCCATGCCGAACT
CAGAAGTGAAACGCCGTAGCGCCGATGGTAGTGTGGGGTCTCCCCATGCGAGAGTAGGGAACTGCCAGGCATCAAATAAAACG
AAAGGCTCAGTCGAAAGACTGGGCCTTTCGTTTTATCTGTTGTTTGTCGGTGAACGCTCTCCTGAGTAGGACAAATCCTGGCT
GTTTTGGCGGAT

DNA sequence of pSL-chPylRS$^{Mb(IPYE)\text{-}Mm}$:

The pSL- chPylRS$^{Mb(IPYE)\text{-}Mm}$ plasmid contains a pBR322 type origin of replication, a kanamycin resistance gene (KanR), and a chPylRS$^{Mb(IPYE)\text{-}Mm}$ [17] under control of a glnS promoter.

chPylRS$^{Mb(IPYE)\text{-}Mm}$; KanR; pBR322 ori.

CGAACTTTTGCTGAGTTGAAGGATCCTCGGGTTGTCAGCCTGTCCCGCTTATAAGATCATACGCCGTTATACGTTGTTTACGC
TTTGAGGAATCCCATATGGATAAGAAGCCGCTGGATGTTCTGATCTCTGCGACCGGTCTGTGGATGTCCCGTACCGGCACGCT
GCACAAGATCAAGCACTATGAGATTTCTCGTTCTAAAATCTACATCGAAATGGCGTGTGGTGACCATCTGGTTGTGAACAACT

CTCGTTCTTGTCGTCCCGCACGTGCATTCCGTTATCATAAATACCGTAAAACCTGCAAACGTTGTCGTGTTTCTGACGAAGAT
ATCAACAACTTCCTGACCCGTTCTACCGAAGGCAAAACCTCTGTTAAAGTTAAAGTTGTTTCTGAGCCGAAAGTGAAAAAAGC
GATGCCGAAATCTGTTTCTCGTGCGCCGAAACCGCTGGAAAATCCGGTTTCTGCGAAAGCGTCTACCGACACCTCTCGTTCTG
TTCCGTCTCCGGCGAAATCTACCCCGAACTCTCCGGTTCCGACCTCTGCGAGCGCGCCGGCGCTGACCAAAAGCCAGACCGAT
CGCCTGGAAGTGCTGCTGAACCCGAAAGATGAAATTAGCCTGAACAGCGGCAAACCGTTTCGCGAACTGGAAAGCGAACTGCT
GAGCCGCCGCAAAAAAGATCTGCAGCAGATTTATGCGGAAGAACGCGAAAACTATCTGGGCAAACTGGAACGCGAAATTACCC
GCTTTTTTGTGGATCGCGGCTTTCTGGAAATTAAAAGCCCGATTCTGATTCCGCTGGAATATATTGAACGCATGGGCATTGAT
AACGATACCGAACTGAGCAAACAGATTTTTCGCGTGGATAAAAACTTTTGCCTGCGCCCGATGCTGGCGCCGAACCTGTATAA
CTATCTGCGCAAACTGGATCGCGCGCTGCCGGATCCGATTAAAATTTTTGAAATTGGCCCGTGCTATCGCAAAGAAAGCGATG
GCAAAGAACATCTGGAAGAATTTACCATGCTGAACTTTTGCCAGATGGGCAGCGGCTGCACCCGCGAAAACCTGGAAAGCATT
ATTACCGATTTTCTGAACCATCTGGGCATTGATTTTAAAATTGTGGGCGATAGCTGCATGGTGTATGGCGATACCCTGGATGT
GATGCATGGCGATCTGGAACTGAGCAGCGCGGTGGTGGGCCCGATTCCGCTGGATCGCGAATGGGGCATTGATAAACCGTGGA
TTGGCGCGGGCTTTGGCCTGGAACGCCTGCTGAAAGTGAAACATGATTTTAAAAACATTAAACGCGCGGCGCGCAGCGAAAGC
TATTATAACGGCATTAGCACCAACCTGTAAAGGTGGCACTTTTCGGGGAAATGTGCGCGGAACCCCTATTTGTTTATTTTTCT
AAATACATTCAAATATGTATCCGCTCATGAATTAATTCTTAGAAAAACTCATCGAGCATCAAATGAAACTGCAATTTATTCAT
ATCAGGATTATCAATACCATATTTTTGAAAAAGCCGTTTCTGTAATGAAGGAGAAAACTCACCGAGGCAGTTCCATAGGATGG
CAAGATCCTGGTATCGGTCTGCGATTCCGACTCGTCCAACATCAATACAACCTATTAATTTCCCCTCGTCAAAAATAAGGTTA
TCAAGTGAGAAATCACCATGAGTGACGACTGAATCCGGTGAGAATGGCAAAAGTTTATGCATTTCTTTCCAGACTTGTTCAAC
AGGCCAGCCATTACGCTCGTCATCAAAATCACTCGCATCAACCAAACCGTTATTCATTCGTGATTGCGCCTGAGCGAGACGAA
ATACGCGATCGCTGTTAAAAGGACAATTACAAACAGGAATCGAATGCAACCGGCGCAGGAACACTGCCAGCGCATCAACAATA
TTTTCACCTGAATCAGGATATTCTTCTAATACCTGGAATGCTGTTTTCCCGGGGATCGCAGTGGTGAGTAACCATGCATCATC
AGGAGTACGGATAAAATGCTTGATGGTCGGAAGAGGCATAAATTCCGTCAGCCAGTTTAGTCTGACCATCTCATCTGTAACAT
CATTGGCAACGCTACCTTTGCCATGTTTCAGAAACAACTCTGGCGCATCGGGCTTCCCATACAATCGATAGATTGTCGCACCT
GATTGCCCGACATTATCGCGAGCCCATTTATACCCATATAAATCAGCATCCATGTTGGAATTTAATCGCGGCCTAGAGCAAGA
CGTTTCCCGTTGAATATGGCTCATAACACCCCTTGTATTACTGTTTATGTAAGCAGACAGTTTTATTGTTCATGACCAAAATC
CCTTAACGTGAGTTTTCGTTCCACTGAGCGTCAGACCCCGTAGAAAAGATCAAAGGATCTTCTTGAGATCCTTTTTTTCTGCG
CGTAATCTGCTGCTTGCAAACAAAAAAACCACCGCTACCAGCGGTGGTTTGTTTGCCGGATCAAGAGCTACCAACTCTTTTTC
CGAAGGTAACTGGCTTCAGCAGAGCGCAGATACCAAATACTGTCCTTCTAGTGTAGCCGTAGTTAGGCCACCACTTCAAGAAC
TCTGTAGCACCGCCTACATACCTCGCTCTGCTAATCCTGTTACCAGTGGCTGCTGCCAGTGGCGATAAGTCGTGTCTTACCGG
GTTGGACTCAAGACGATAGTTACCGGATAAGGCGCAGCGGTCGGGCTGAACGGGGGGTTCGTGCACACAGCCCAGCTTGGAGC
GAACGACCTACACCGAACTGAGATACCTACAGCGTGAGCTATGAGAAAGCGCCACGCTTCCCGAAGGGAGAAAGGCGGACAGG
TATCCGGTAAGCGGCAGGGTCGGAACAGGAGAGCGCACGAGGGAGCTTCCAGGGGGAAACGCCTGGTATCTTTATAGTCCTGT
CGGGTTTCGCCACCTCTGACTTGAGCGTCGATTTTTGTGATGCTCGTCAGGGGGGCGGAGCCTATGGAAAAACGCCAGCAACG
CGGCCTTTTTACGGTTCCTGGCCTTTTGCTGGCCTTTTGCTCACATGTTCTTTCCTGCGTTATCCCCTGATTCTGTGGATAAC
CGTATTACCGCCTTTGAGTGAGCTGATACCGCTCGCCGCAGCCGAACGACCGAGCGCAGCGAGTCAGTGAGCGAGGAAGCGGA
AGAG

DNA sequence of pSL-chPylRS$^{Me\text{-}Mm}$:

The pSL- chPylRS$^{Me\text{-}Mm}$ plasmid contains a pBR322 type origin of replication, a kanamycin resistance gene (KanR), and a chPylRS$^{Me\text{-}Mm}$ under control of a glnS promoter.

chPylRS$^{Me\text{-}Mm}$; KanR; pBR322 ori.

CGAACTTTTGCTGAGTTGAAGGATCCTCGGGTTGTCAGCCTGTCCCGCTTATAAGATCATACGCCGTTATACGTTGTTTACGC
TTTGAGGAATCCCATATGAGCAAAAAAAGCCTGGCGAGCCTGATTAGCGATCTGCAGGTGTGGGTGAGCCGCAGCGGCCTGCT
GCATGAAATTAAAAACTATGAAGTGAGCCAGCGCTATATTCATATGGAAATGGATTGCGGCGAAAAAATTACCGTGCGCAACA
GCCGCAACAGCCGCACCGCGCGCATTCTGCGCCTGAAAAAATATAAAAAACCGTGCAAAAACTGCAAAGTGAGCGATGAAGTG
ATTAACCGCTTTCTGCAGAAACATACCGATCGCACCGATACCAAAGTGCTGACCAAAGCGAACGAAGATCAGACCAGCGTGAA
AGTGAAAGTGGTGAGCGCGCCGACCCGCACCAAAAAAGCGATGCCGAAAAGCGTGGCGCGCGCGCCGAAACCGCTGGAAAACA
CCGAAGCGGCGCAGGCGCAGCCGAGCGGCAGCAAATTTAGCCCGGCGATTCCGGTGAGCACCCAGGAAAGCGTGAGCGTGCCG
GCGAGCGTGAGCACCAGCATTAGCAGCATTAGCACCGGCGCGACCGCGAGCGCGCTGGTGAAAGGCAACACCAACCCGATTAC
CAGCATGAGCGCGCCGGTGCAGGCGAGCGCGCCGGCGCTGACCAAAAGCCAGACCGATCGCCTGGAAGTGCTGCTGAACCCGA
AAGATGAAATTAGCCTGAACAGCGGCAAACCGTTTCGCGAACTGGAAAGCGAACTGCTGAGCCGCCGCAAAAAAGATCTGCAG
CAGATTTATGCGGAAGAACGCGAAAACTATCTGGGCAAACTGGAACGCGAAATTACCCGCTTTTTTGTGGATCGCGGCTTTCT
GGAAATTAAAAGCCCGATTCTGATTCCGCTGGAATATATTGAACGCATGGGCATTGATAACGATACCGAACTGAGCAAACAGA
TTTTTCGCGTGGATAAAAACTTTTGCCTGCGCCCGATGCTGGCGCCGAACCTGTATAACTATCTGCGCAAACTGGATCGCGCG
CTGCCGGATCCGATTAAAATTTTTGAAATTGGCCCGTGCTATCGCAAAGAAAGCGATGGCAAAGAACATCTGGAAGAATTTAC
CATGCTGAACTTTTGCCAGATGGGCAGCGGCTGCACCCGCGAAAACCTGGAAAGCATTATTACCGATTTTCTGAACCATCTGG
GCATTGATTTTAAAATTGTGGGCGATAGCTGCATGGTGTATGGCGATACCCTGGATGTGATGCATGGCGATCTGGAACTGAGC
AGCGCGGTGGTGGGCCCGATTCCGCTGGATCGCGAATGGGGCATTGATAAACCGTGGATTGGCGCGGGCTTTGGCCTGGAACG
CCTGCTGAAAGTGAAACATGATTTTAAAAACATTAAACGCGCGGCGCGCAGCGAAAGCTATTATAACGGCATTAGCACCAACC
TGTAAAGGTGGCACTTTTCGGGGAAATGTGCGCGGAACCCCTATTTGTTTATTTTTCTAAATACATTCAAATATGTATCCGCT
CATGAATTAATTCTTAGAAAAACTCATCGAGCATCAAATGAAACTGCAATTTATTCATATCAGGATTATCAATACCATATTTT
TGAAAAAGCCGTTTCTGTAATGAAGGAGAAAACTCACCGAGGCAGTTCCATAGGATGGCAAGATCCTGGTATCGGTCTGCGAT

TCCGACTCGTCCAACATCAATACAACCTATTAATTTCCCCTCGTCAAAAATAAGGTTATCAAGTGAGAAATCACCATGAGTGA
CGACTGAATCCGGTGAGAATGGCAAAAGTTTATGCATTTCTTTCCAGACTTGTTCAACAGGCCAGCCATTACGCTCGTCATCA
AAATCACTCGCATCAACCAAACCGTTATTCATTCGTGATTGCGCCTGAGCGAGACGAAATACGCGATCGCTGTTAAAAGGACA
ATTACAAACAGGAATCGAATGCAACCGGCGCAGGAACACTGCCAGCGCATCAACAATATTTTCACCTGAATCAGGATATTCTT
CTAATACCTGGAATGCTGTTTTCCCGGGGATCGCAGTGGTGAGTAACCATGCATCATCAGGAGTACGGATAAAATGCTTGATG
GTCGGAAGAGGCATAAATTCCGTCAGCCAGTTTAGTCTGACCATCTCATCTGTAACATCATTGGCAACGCTACCTTTGCCATG
TTTCAGAAACAACTCTGGCGCATCGGGCTTCCCATACAATCGATAGATTGTCGCACCTGATTGCCCGACATTATCGCGAGCCC
ATTTATACCCATATAAATCAGCATCCATGTTGGAATTTAATCGCGGCCTAGAGCAAGACGTTTCCCGTTGAATATGGCTCATA
ACACCCCTTGTATTACTGTTTATGTAAGCAGACAGTTTTATTGTTCATGACCAAAATCCCTTAACGTGAGTTTTCGTTCCACT
GAGCGTCAGACCCCGTAGAAAAGATCAAAGGATCTTCTTGAGATCCTTTTTTTCTGCGCGTAATCTGCTGCTTGCAAACAAAA
AAACCACCGCTACCAGCGGTGGTTTGTTTGCCGGATCAAGAGCTACCAACTCTTTTTCCGAAGGTAACTGGCTTCAGCAGAGC
GCAGATACCAAATACTGTCCTTCTAGTGTAGCCGTAGTTAGGCCACCACTTCAAGAACTCTGTAGCACCGCCTACATACCTCG
CTCTGCTAATCCTGTTACCAGTGGCTGCTGCCAGTGGCGATAAGTCGTGTCTTACCGGGTTGGACTCAAGACGATAGTTACCG
GATAAGGCGCAGCGGTCGGGCTGAACGGGGGGTTCGTGCACACAGCCCAGCTTGGAGCGAACGACCTACACCGAACTGAGATA
CCTACAGCGTGAGCTATGAGAAAGCGCCACGCTTCCCGAAGGGAGAAAGGCGGACAGGTATCCGGTAAGCGGCAGGGTCGGAA
CAGGAGAGCGCACGAGGGAGCTTCCAGGGGGAAACGCCTGGTATCTTTATAGTCCTGTCGGGTTTCGCCACCTCTGACTTGAG
CGTCGATTTTTGTGATGCTCGTCAGGGGGGCGGAGCCTATGGAAAAACGCCAGCAACGCGGCCTTTTTACGGTTCCTGGCCTT
TTGCTGGCCTTTTGCTCACATGTTCTTTCCTGCGTTATCCCCTGATTCTGTGGATAACCGTATTACCGCCTTTGAGTGAGCTG
ATACCGCTCGCCGCAGCCGAACGACCGAGCGCAGCGAGTCAGTGAGCGAGGAAGCGGAAGAG

DNA sequence of pSL-chPylRS^{E7}:

The pSL-chPylRS^{E7} plasmid contains a pBR322 type origin of replication, a kanamycin resistance gene (KanR), and a chPylRS^{E7}, that is chPylRS$^{\textit{Me-Mm}}$ with the mutations S8C/K45E/P68Q/K70I/V74A/N80S and K93I, under control of a glnS promoter.

chPylRS^{E7}; KanR; pBR322 ori.

CGAACTTTTGCTGAGTTGAAGGATCCTCGGGTTGTCAGCCTGTCCCGCTTATAAGATCATACGCCGTTATACGTTGTTTACGC
TTTGAGGAATCCCATATGAGCAAAAAAAGCCTGGCGTgcCTGATTAGCGATCTGCAGGTGTGGGTGAGCCGCAGCGGCCTGCT
GCATGAAATTAAAAACTATGAaGTGAGCCAGCGCTATATTCATATGGAAATGGATTGCGGCGAAAAAATTACCGTGCGCAACA
GCCGCAACAGCCGCACCGCGCGCATTCTGCGCCTGAAAAAATATAAAAAACCGTGCAAAAACTGCAAAGTGAGCGATGAAGTG
ATTAACCGCTTTCTGCAGAAACATACCGATCGCACCGATACCAAAGTGCTGACCAAAGCGAACGAAGATCAGACCAGCGTGAA
AGTGAAAGTGGTGAGCGCGCCGACCCGCACCAAAAAAGCGATGCCGAAAAGCGTGGCGCGCGCGCCGAAACCGCTGGAAAACA
CCGAAGCGGCGCAGGCGCAGCCGAGCGGCAGCAAATTTAGCCCGGCGATTCCGGTGAGCACCCAGGAAAGCGTGAGCGTGCCG
GCGAGCGTGAGCACCAGCATTAGCAGCATTAGCACCGGCGCGACCGCGAGCGCGCTGGTGAAAGGCAACACCAACCCGATTAC
CAGCATGAGCGCGCCGGTGCAGGCGAGCGCGCCGGCGCTGACCAAAAGCCAGACCGATCGCCTGGAAGTGCTGCTGAACCCGA
AAGATGAAATTAGCCTGAACAGCGGCAAACCGTTTCGCGAACTGGAAAGCGAACTGCTGAGCCGCCGCAAAAAAGATCTGCAG
CAGATTTATGCGGAAGAACGCGAAAACTATCTGGGCAAACTGGAACGCGAAATTACCCGCTTTTTTGTGGATCGCGGCTTTCT
GGAAATTAAAAGCCCGATTCTGATTCCGCTGGAATATATTGAACGCATGGGCATTGATAACGATACCGAACTGAGCAAACAGA
TTTTTCGCGTGGATAAAAACTTTTGCCTGCGCCCGATGCTGGCGCCGAACCTGTATAACTATCTGCGCAAACTGGATCGCGCG
CTGCCGGATCCGATTAAAATTTTTGAAATTGGCCCGTGCTATCGCAAAGAAAGCGATGGCAAAGAACATCTGGAAGAATTTAC
CATGCTGAACTTTTGCCAGATGGGCAGCGGCTGCACCCGCGAAAACCTGGAAAGCATTATTACCGATTTTCTGAACCATCTGG
GCATTGATTTTAAAATTGTGGGCGATAGCTGCATGGTGTATGGCGATACCCTGGATGTGATGCATGGCGATCTGGAACTGAGC
AGCGCGGTGGTGGGCCCGATTCCGCTGGATCGCGAATGGGGCATTGATAAACCGTGGATTGGCGCGGGCTTTGGCCTGGAACG
CCTGCTGAAAGTGAAACATGATTTTAAAAACATTAAACGCGCGGCGCGCAGCGAAAGCTATTATAACGGCATTAGCACCAACC
TGTAAAGGTGGCACTTTTCGGGGAAATGTGCGCGGAACCCCTATTTGTTTATTTTTCTAAATACATTCAAATATGTATCCGCT
CATGAATTAATTCTTAGAAAAACTCATCGAGCATCAAATGAAACTGCAATTTATTCATATCAGGATTATCAATACCATATTTT
TGAAAAAGCCGTTTCTGTAATGAAGGAGAAAACTCACCGAGGCAGTTCCATAGGATGGCAAGATCCTGGTATCGGTCTGCGAT
TCCGACTCGTCCAACATCAATACAACCTATTAATTTCCCCTCGTCAAAAATAAGGTTATCAAGTGAGAAATCACCATGAGTGA
CGACTGAATCCGGTGAGAATGGCAAAAGTTTATGCATTTCTTTCCAGACTTGTTCAACAGGCCAGCCATTACGCTCGTCATCA
AAATCACTCGCATCAACCAAACCGTTATTCATTCGTGATTGCGCCTGAGCGAGACGAAATACGCGATCGCTGTTAAAAGGACA
ATTACAAACAGGAATCGAATGCAACCGGCGCAGGAACACTGCCAGCGCATCAACAATATTTTCACCTGAATCAGGATATTCTT
CTAATACCTGGAATGCTGTTTTCCCGGGGATCGCAGTGGTGAGTAACCATGCATCATCAGGAGTACGGATAAAATGCTTGATG
GTCGGAAGAGGCATAAATTCCGTCAGCCAGTTTAGTCTGACCATCTCATCTGTAACATCATTGGCAACGCTACCTTTGCCATG
TTTCAGAAACAACTCTGGCGCATCGGGCTTCCCATACAATCGATAGATTGTCGCACCTGATTGCCCGACATTATCGCGAGCCC
ATTTATACCCATATAAATCAGCATCCATGTTGGAATTTAATCGCGGCCTAGAGCAAGACGTTTCCCGTTGAATATGGCTCATA
ACACCCCTTGTATTACTGTTTATGTAAGCAGACAGTTTTATTGTTCATGACCAAAATCCCTTAACGTGAGTTTTCGTTCCACT
GAGCGTCAGACCCCGTAGAAAAGATCAAAGGATCTTCTTGAGATCCTTTTTTTCTGCGCGTAATCTGCTGCTTGCAAACAAAA
AAACCACCGCTACCAGCGGTGGTTTGTTTGCCGGATCAAGAGCTACCAACTCTTTTTCCGAAGGTAACTGGCTTCAGCAGAGC
GCAGATACCAAATACTGTCCTTCTAGTGTAGCCGTAGTTAGGCCACCACTTCAAGAACTCTGTAGCACCGCCTACATACCTCG
CTCTGCTAATCCTGTTACCAGTGGCTGCTGCCAGTGGCGATAAGTCGTGTCTTACCGGGTTGGACTCAAGACGATAGTTACCG
GATAAGGCGCAGCGGTCGGGCTGAACGGGGGGTTCGTGCACACAGCCCAGCTTGGAGCGAACGACCTACACCGAACTGAGATA
CCTACAGCGTGAGCTATGAGAAAGCGCCACGCTTCCCGAAGGGAGAAAGGCGGACAGGTATCCGGTAAGCGGCAGGGTCGGAA

CAGGAGAGCGCACGAGGGAGCTTCCAGGGGGAAACGCCTGGTATCTTTATAGTCCTGTCGGGTTTCGCCACCTCTGACTTGAG
CGTCGATTTTTGTGATGCTCGTCAGGGGGGCGGAGCCTATGGAAAAACGCCAGCAACGCGGCCTTTTTACGGTTCCTGGCCTT
TTGCTGGCCTTTTGCTCACATGTTCTTTCCTGCGTTATCCCCTGATTCTGTGGATAACCGTATTACCGCCTTTGAGTGAGCTG
ATACCGCTCGCCGCAGCCGAACGACCGAGCGCAGCGAGTCAGTGAGCGAGGAAGCGGAAGAG

DNA sequence of pSL-chPheRS(T357G/A507G):

The pSL-chPheRS(T467G/A507G) plasmid contains a pBR322 type origin of replication, a kanamycin resistance gene (KanR), and a chPheRS(T357G/A507G) under control of a glnS promoter.

chPheRS(T467G/A507G); KanR; pBR322 ori.

CGAACTTTTGCTGAGTTGAAGGATCCTCGGGTTGTCAGCCTGTCCCGCTTATAAGATCATACGCCGTTATACGTTGTTTACGC
TTTGAGGAATCCCATATGGATAAGAAGCCGCTGGATGTTCTGATCTCTGCGACCGGcCTGTGGATGTCCCGTACCGGCACGCT
GCACAAGATCAAGCACTATGAGATTTCTCGTTCTAAAATCTACATCGAAATGGCGTGTGGTGACCATCTGGTTGTGAACAACT
CTCGTTCTTGTCGTCCCGCACGTGCATTCCGTTATCATAAATACCGTAAAACCTGCAAACGTTGTCGTGTTTCTGACGAAGAT
ATCAACAACTTCCTGACCCGTTCTACCGAAGGCAAAACCTCTGTTAAAGTTAAAGTTGTTTCTGAGCCGAAAGTGAAAAAAGC
GATGCCGAAATCTGTTTCTCGTGCGCCGAAACCGCTGGAAAATCCGGTTTCTGCGAAAGCGTCTACCGACACCTCTCGTTCTG
TTCCGTCTCCGGCGAAATCTACCCCGAACTCTCCGGTTCCGACCTCTGCAAGCGCCCCAGCTCTGACTAAATCCCAGACGGAC
CGTCTGGAGGTGCTGCTGAACCCAAAGGATGAAATCTCTCTGAACAGCGGCAAGCCTTTCCGTGAGCTGGAAAGCGAGCTGCT
GTCTCGTCGTAAAAAGGATCTGCAACAGATCTACGCTGAGGAACGCGAGGGTGGCGGAAGCGGCGGCGGAAGCGGTGGCGGAA
GTGGTGGCGGAAGCGGCGGCGGAAGCCAGGCCTGGGGATCGAGGCCTCCTGCAGCAGAGTGTGCCACCCAAAGAGCTCCAGGC
AGTGTGGTGGAGCTGCTGGGCAAATCCTACCCTCAGGACGACCACAGCAACCTCACCCGGAAGGTCCTCACCAGAGTTGGCAG
GAACCTGCACAACCAGCAGCATCACCCTCTGTGGCTGATCAAGGAGAGGGTGAAGGAGCACTTCTACAAGCAGTATGTGGGCC
GCTTTGGGACCCCGTTGTTCTCTGTCTACGACAACCTTTCTCCAGTGGTCACGACCTGGCAGAACTTTGACAGCCTGCTCATC
CCAGCTGATCACCCCAGCAGGAAGAAGGGGGACAACTATTACCTGAATCGGACTCACATGCTGAGAGCGCACACGTCCGCACA
CCAGTGGGACTTGCTGCACGCGGGACTGGATGCCTTCCTGGTGGTGGGTGATGTCTACAGGCGTGACCAGATCGACTCCCAGC
ACTACCCTATTTTCCACCAGCTGGAGGCCGTGCGGCTCTTCTCCAAGCATGAGTTATTTGCTGGTATAAAGGATGGAGAAAGC
CTGCAGCTCTTTGAACAAAGTTCTCGCTCTGCGCATAAACAAGAGACACACACCATGGAGGCCGTGAAGCTTGTAGAGTTTGA
TCTTAAGCAAACGCTTACCAGGCTCATGGCACATCTTTTTGGAGATGAGCTGGAGATAAGGTGGGTAGACTGCTACTTCCCTT
TTGGACATCCTTCCTTTGAGATGGAGATCAACTTTCATGGAGAATGGCTGGAAGTTCTTGGCTGCGGGGTGATGGAACAACAA
CTGGTCAATTCtGCTGGTGCTCAAGACCGAATCGGCTGGGGATTTGGCCTAGGgTTAGAAAGGCTAGCCATGATCCTCTACGA
CATCCCTGATATCCGTCTCTTCTGGTGTGAGGACGAGCGCTTCCTGAAGCAGTTCTGTGTATCCAACATTAATCAGAAGGTGA
AGTTTCAGCCTCTTAGCAAATAAAGGTGGCACTTTTCGGGGAAATGTGCGCGGAACCCCTATTTGTTTATTTTTCTAAATACA
TTCAAATATGTATCCGCTCATGAATTAATTCTTAGAAAAACTCATCGAGCATCAAATGAAACTGCAATTTATTCATATCAGGA
TTATCAATACCATATTTTTGAAAAAGCCGTTTCTGTAATGAAGGAGAAAACTCACCGAGGCAGTTCCATAGGATGGCAAGATC
CTGGTATCGGTCTGCGATTCCGACTCGTCCAACATCAATACAACCTATTAATTTCCCCTCGTCAAAAATAAGGTTATCAAGTG
AGAAATCACCATGAGTGACGACTGAATCCGGTGAGAATGGCAAAAGTTTATGCATTTCTTTCCAGACTTGTTCAACAGGCCAG
CCATTACGCTCGTCATCAAAATCACTCGCATCAACCAAACCGTTATTCATTCGTGATTGCGCCTGAGCGAGACGAAATACGCG
ATCGCTGTTAAAAGGACAATTACAAACAGGAATCGAATGCAACCGGCGCAGGAACACTGCCAGCGCATCAACAATATTTTCAC
CTGAATCAGGATATTCTTCTAATACCTGGAATGCTGTTTTCCCGGGGATCGCAGTGGTGAGTAACCATGCATCATCAGGAGTA
CGGATAAAATGCTTGATGGTCGGAAGAGGCATAAATTCCGTCAGCCAGTTTAGTCTGACCATCTCATCTGTAACATCATTGGC
AACGCTACCTTTGCCATGTTTCAGAAACAACTCTGGCGCATCGGGCTTCCCATACAATCGATAGATTGTCGCACCTGATTGCC
CGACATTATCGCGAGCCCATTTATACCCATATAAATCAGCATCCATGTTGGAATTTAATCGCGGCCTAGAGCAAGACGTTTCC
CGTTGAATATGGCTCATAACACCCCTTGTATTACTGTTTATGTAAGCAGACAGTTTTATTGTTCATGACCAAAATCCCTTAAC
GTGAGTTTTCGTTCCACTGAGCGTCAGACCCCGTAGAAAAGATCAAAGGATCTTCTTGAGATCCTTTTTTTCTGCGCGTAATC
TGCTGCTTGCAAACAAAAAAACCACCGCTACCAGCGGTGGTTTGTTTGCCGGATCAAGAGCTACCAACTCTTTTTCCGAAGGT
AACTGGCTTCAGCAGAGCGCAGATACCAAATACTGTCCTTCTAGTGTAGCCGTAGTTAGGCCACCACTTCAAGAACTCTGTAG
CACCGCCTACATACCTCGCTCTGCTAATCCTGTTACCAGTGGCTGCTGCCAGTGGCGATAAGTCGTGTCTTACCGGGTTGGAC
TCAAGACGATAGTTACCGGATAAGGCGCAGCGGTCGGGCTGAACGGGGGGTTCGTGCACACAGCCCAGCTTGGAGCGAACGAC
CTACACCGAACTGAGATACCTACAGCGTGAGCTATGAGAAAGCGCCACGCTTCCCGAAGGGAGAAAGGCGGACAGGTATCCGG
TAAGCGGCAGGGTCGGAACAGGAGAGCGCACGAGGGAGCTTCCAGGGGGAAACGCCTGGTATCTTTATAGTCCTGTCGGGTTT
CGCCACCTCTGACTTGAGCGTCGATTTTTGTGATGCTCGTCAGGGGGGCGGAGCCTATGGAAAAACGCCAGCAACGCGGCCTT
TTTACGGTTCCTGGCCTTTTGCTGGCCTTTTGCTCACATGTTCTTTCCTGCGTTATCCCCTGATTCTGTGGATAACCGTATTA
CCGCCTTTGAGTGAGCTGATACCGCTCGCCGCAGCCGAACGACCGAGCGCAGCGAGTCAGTGAGCGAGGAAGCGGAAGAG

DNA sequence of pSL-*Mm*PylRS:

The pSL-*Mm*PylRS plasmid contains a pBR322 type origin of replication, a kanamycin resistance gene (KanR), and an *Mm*PylRS under control of a glnS promoter.

*Mm*PylRS; KanR; pBR322 ori.

CGAACTTTTGCTGAGTTGAAGGATCCTCGGGTTGTCAGCCTGTCCCGCTTATAAGATCATACGCCGTTATACGTTGTTTACGC
TTTGAGGAATCCCATATGGATAAAAAACCGCTGAACACCCTGATTAGCGCGACCGGCCTGTGGATGAGCCGCACCGGCACCAT
TCATAAAATTAAACATCATGAAGTGAGCCGCAGCAAAATTTATATTGAAATGGCGTGCGGCGATCATCTGGTGGTGAACAACA
GCCGCAGCAGCCGCACCGCGCGCGCGCTGCGCCATCATAAATATCGCAAAACCTGCAAACGCTGCCGCGTGAGCGATGAAGAT
CTGAACAAATTTCTGACCAAAGCGAACGAAGATCAGACCAGCGTGAAAGTGAAAGTGGTGAGCGCGCCGACCCGCACCAAAAA
AGCGATGCCGAAAAGCGTGGCGCGCGCGCCGAAACCGCTGGAAAACACCGAAGCGGCGCAGGCGCAGCCGAGCGGCAGCAAAT
TTAGCCCGGCGATTCCGGTGAGCACCCAGGAAAGCGTGAGCGTGCCGGCGAGCGTGAGCACCAGCATTAGCAGCATTAGCACC
GGCGCGACCGCGAGCGCGCTGGTGAAAGGCAACACCAACCCGATTACCAGCATGAGCGCGCCGGTGCAGGCGAGCGCGCCGGC
GCTGACCAAAAGCCAGACCGATCGCCTGGAAGTGCTGCTGAACCCGAAAGATGAAATTAGCCTGAACAGCGGCAAACCGTTTC
GCGAACTGGAAAGCGAACTGCTGAGCCGCCGCAAAAAAGATCTGCAGCAGATTTATGCGGAAGAACGCGAAAACTATCTGGGC
AAACTGGAACGCGAAATTACCCGCTTTTTTGTGGATCGCGGCTTTCTGGAAATTAAAAGCCCGATTCTGATTCCGCTGGAATA
TATTGAACGCATGGGCATTGATAACGATACCGAACTGAGCAAACAGATTTTTCGCGTGGATAAAAACTTTTGCCTGCGCCCGA
TGCTGGCGCCGAACCTGTATAACTATCTGCGCAAACTGGATCGCGCGCTGCCGGATCCGATTAAAATTTTTGAAATTGGCCCG
TGCTATCGCAAAGAAAGCGATGGCAAAGAACATCTGGAAGAATTTACCATGCTGAACTTTTGCCAGATGGGCAGCGGCTGCAC
CCGCGAAAACCTGGAAAGCATTATTACCGATTTTCTGAACCATCTGGGCATTGATTTTAAAATTGTGGGCGATAGCTGCATGG
TGTATGGCGATACCCTGGATGTGATGCATGGCGATCTGGAACTGAGCAGCGCGGTGGTGGGCCCGATTCCGCTGGATCGCGAA
TGGGGCATTGATAAACCGTGGATTGGCGCGGGCTTTGGCCTGGAACGCCTGCTGAAAGTGAAACATGATTTTAAAAACATTAA
ACGCGCGGCGCGCAGCGAAAGCTATTATAACGGCATTAGCACCAACCTGTAAAGGTGGCACTTTTCGGGGAAATGTGCGCGGA
ACCCCTATTTGTTTATTTTTCTAAATACATTCAAATATGTATCCGCTCATGAATTAATTCTTAGAAAAACTCATCGAGCATCA
AATGAAACTGCAATTTATTCATATCAGGATTATCAATACCATATTTTTGAAAAAGCCGTTTCTGTAATGAAGGAGAAAACTCA
CCGAGGCAGTTCCATAGGATGGCAAGATCCTGGTATCGGTCTGCGATTCCGACTCGTCCAACATCAATACAACCTATTAATTT
CCCCTCGTCAAAAATAAGGTTATCAAGTGAGAAATCACCATGAGTGACGACTGAATCCGGTGAGAATGGCAAAAGTTTATGCA
TTTCTTTCCAGACTTGTTCAACAGGCCAGCCATTACGCTCGTCATCAAAATCACTCGCATCAACCAAACCGTTATTCATTCGT
GATTGCGCCTGAGCGAGACGAAATACGCGATCGCTGTTAAAAGGACAATTACAAACAGGAATCGAATGCAACCGGCGCAGGAA
CACTGCCAGCGCATCAACAATATTTTCACCTGAATCAGGATATTCTTCTAATACCTGGAATGCTGTTTTCCCGGGGATCGCAG
TGGTGAGTAACCATGCATCATCAGGAGTACGGATAAAATGCTTGATGGTCGGAAGAGGCATAAATTCCGTCAGCCAGTTTAGT
CTGACCATCTCATCTGTAACATCATTGGCAACGCTACCTTTGCCATGTTTCAGAAACAACTCTGGCGCATCGGGCTTCCCATA
CAATCGATAGATTGTCGCACCTGATTGCCCGACATTATCGCGAGCCCATTTATACCCATATAAATCAGCATCCATGTTGGAAT
TTAATCGCGGCCTAGAGCAAGACGTTTCCCGTTGAATATGGCTCATAACACCCCTTGTATTACTGTTTATGTAAGCAGACAGT
TTTATTGTTCATGACCAAAATCCCTTAACGTGAGTTTTCGTTCCACTGAGCGTCAGACCCCGTAGAAAAGATCAAAGGATCTT
CTTGAGATCCTTTTTTTCTGCGCGTAATCTGCTGCTTGCAAACAAAAAAACCACCGCTACCAGCGGTGGTTTGTTTGCCGGAT
CAAGAGCTACCAACTCTTTTTCCGAAGGTAACTGGCTTCAGCAGAGCGCAGATACCAAATACTGTCCTTCTAGTGTAGCCGTA
GTTAGGCCACCACTTCAAGAACTCTGTAGCACCGCCTACATACCTCGCTCTGCTAATCCTGTTACCAGTGGCTGCTGCCAGTG
GCGATAAGTCGTGTCTTACCGGGTTGGACTCAAGACGATAGTTACCGGATAAGGCGCAGCGGTCGGGCTGAACGGGGGGTTCG
TGCACACAGCCCAGCTTGGAGCGAACGACCTACACCGAACTGAGATACCTACAGCGTGAGCTATGAGAAAGCGCCACGCTTCC
CGAAGGGAGAAAGGCGGACAGGTATCCGGTAAGCGGCAGGGTCGGAACAGGAGAGCGCACGAGGGAGCTTCCAGGGGGAAACG
CCTGGTATCTTTATAGTCCTGTCGGGTTTCGCCACCTCTGACTTGAGCGTCGATTTTTGTGATGCTCGTCAGGGGGGCGGAGC
CTATGGAAAAACGCCAGCAACGCGGCCTTTTTACGGTTCCTGGCCTTTTGCTGGCCTTTTGCTCACATGTTCTTTCCTGCGTT
ATCCCCTGATTCTGTGGATAACCGTATTACCGCCTTTGAGTGAGCTGATACCGCTCGCCGCAGCCGAACGACCGAGCGCAGCG
AGTCAGTGAGCGAGGAAGCGGAAGAG

DNA sequence of pBARN -*Mm*tRNA$^{Pyl}_{CUA}$:

The pBARN-*Mm*tRNA$^{Pyl}_{CUA}$ plasmid contains a p15A type origin of replication, a chloramphenicol resistance gene (CamR), and an *Mm*tRNA$^{Pyl}_{CUA}$ under control of a proK promoter.

***Mm*tRNA$^{Pyl}_{CUA}$**; **CamR**; **p15A ori**; araBAD-barnase(3TAG, 45TAG, 66TAG).

GGCACCTCGCTAACGGACGCCTGAATAAGTGATAATAAGCGGATGAATGGCAGAAATTCGAAAGCAAATTCGACCCTGAGCTG
CTCGAGCATGCAAGGCATTTTGCTATTAAGGGATTGACGAGGGCGTATCTGCGCAGTAAGATGCGCCCCGCATT**GGAAACCTG**
ATCATGTAGATCGAATGGACTCTAAATCCGTTCAGCCGGGTTAGATTCCCGGGGTTTCCGCCAAATTCGAAAAGCCTGCTCAA
CGAGCAGGCTTTTTTGCATGCTCGAGCAGCTCAGGGTCGAATTTGCTTTCGAATTTCTGCCATTCATCCGCTTATTATCACTT
ATTCAGGCGTAGCAACCAGGCGTTTAAGGGCACCAATAACTGCCTTAAAAAAA**TTACGCCCCGCCCTGCCACTCATCGCAGTA**
CTGTTGTAATTCATTAAGCATTCTGCCGACATGGAAGCCATCACAAACGGCATGATGAACCTGAATCGCCAGCGGCATCAGCA
CCTTGTCGCCTTGCGTATAATATTTGCCCATGGTGAAAACGGGGGCGAAGAAGTTGTCCATATTGGCCACGTTTAAATCAAAA
CTGGTGAAACTCACCCAGGGATTGGCTGAGACGAAAAACATATTCTCAATAAACCCTTTAGGGAAATAGGCCAGGTTTTCACC
GTAACACGCCACATCTTGCGAATATATGTGTAGAAACTGCCGGAAATCGTCGTGGTATTCACTCCAGAGCGATGAAAACGTTT
CAGTTTGCTCATGGAAAACGGTGTAACAAGGGTGAACACTATCCCATATCACCAGCTCACCGTCTTTCATTGCCATACGGAAT
TCCGGATGAGCATTCATCAGGCGGGCAAGAATGTGAATAAAGGCCGGATAAAACTTGTGCTTATTTTTCTTTACGGTCTTTAA
AAAGGCCGTAATATCCAGCTGAACGGTCTGGTTATAGGTACATTGAGCAACTGACTGAAATGCCTCAAAATGTTCTTTACGAT
GCCATTGGGATATATCAACGGTGGTATATCCAGTGATTTTTTTCTCCATTTTAGCTTCCTTAGCTCCTGAAAATCTCGATAAC
TCAAAAAATACGCCCGGTAGTGATCTTATTTCATTATGGTGAAAGTTGGAACCTCTTACGTGCCGATCAACGTCTCATTTTCG
CCAAAAGTTGGCCCAGGGCTTCCCGGTATCAACAGGGACACCAGGATTTATTTATTCTGCGAAGTGATCTTCCGTCACAGGTA
TTTATTCGGCGCAAAGTGCGTCGGGTGATGCTGCCAACTTACTGATTTAGTGTATGATGGTGTTTTTGAGGTGCTCCAGTGGC
TTCTGTTTCTATCAGCTGTCCCTCCTGTTCAGCTACTGACGGGGTGGTGCGTAACGGCAAAAGCACCGCCGGACATCAGCGCT
AGCGGAGTGTATACTGGCTTACTATGTTGGCACTGATGAGGGTGTCAGTGAAGTGCTTCATGTGGCAGGAGAAAAAAGGCTGC

ACCGGTGCGTCAGCAGAATATGTGATACAGGATATATTCCGCTTCCTCGCTCACTGACTCGCTACGCTCGGTCGTTCGACTGC
GGCGAGCGGAAATGGCTTACGAACGGGGCGGAGATTTCCTGGAAGATGCCAGGAAGATACTTAACAGGGAAGTGAGAGGGCCG
CGGCAAAGCCGTT**TTTCCATAGGCTCCGCCCCCCTGACAAGCATCACGAAATCTGACGCTCAAATCAGTGGTGGCGAAACCCG**
ACAGGACTATAAAGATACCAGGCGTTTCCCCCTGGCGGCTCCCTCGTGCGCTCTCCTGTTCCTGCCTTTCGGTTTACCGGTGT
CATTCCGCTGTTATGGCCGCGTTTGTCTCATTCCACGCCTGACACTCAGTTCCGGGTAGGCAGTTCGCTCCAAGCTGGACTGT
ATGCACGAACCCCCCGTTCAGTCCGACCGCTGCGCCTTATCCGGTAACTATCGTCTTGAGTCCAACCCGGAAAGACATGCAAA
AGCACCACTGGCAGCAGCCACTGGTAATTGATTTAGAGGAGTTAGTCTTGAAGTCATGCGCCGGTTAAGGCTAAACTGAAAGG
ACAAGTTTTGGTGACTGCGCTCCTCCAAGCCAGTTACCTCGGTTCAAAGAGTTGGTAGCTCAGAGAACCTTCGAAAAACCGCC
CTGCAAGGCGGTTTTTTCGTTTTCAGAGCAAGAGATTACGCGCAGACCAAAACGATCTCAAGAAGATCATCTTATTAATCAGA
TAAAATATTTCTAGATTTCAGTGCAATTTATCTCTTCAAATGTAGCACCTGAAGTCAGCCCCATACGATATAAGTTGTAATTC
TCATGTTTGACAGCATTATCATCGATAAGCTTTAATGCGGTAGTTTATCACAGTTAAATTGCTAACGCAGTCAGGCACCGTGT
ATGAAATCTAACAATGCGCTCATCGTCATCCTCGGCACCGTCACCCTGGATGCTGTAGGCATAGGCTTGGTTATGCCGGTACT
GCCGGGCCTCTTGCGGGATGGCCACGATGCGTCCGGCGTAGAGGATCTGCTCATGTTTGACAGCTTATCATCGATGCATAATG
TGCCTGTCAAATGGACGAAGCAGGGATTCTGCAAACCCTATGCTACTCCGTCAAGCCGTCAATTGTCTGATTCGTTACCAATT
ATGACAACTTGACGGCTACATCATTCACTTTTTCTTCACAACCGGCACGGAACTCGCTCGGGCTGGCCCCGGTGCATTTTTTA
AATACCCGCGAGAAATAGAGTTGATCGTCAAAACCAACATTGCGACCGACGGTGGCGATAGGCATCCGGGTGGTGCTCAAAAG
CAGCTTCGCCTGGTGATACGTTGGTCCTCGCGCCAGCTTAAGACGCTAATCCCTAACTGCTGGCGGAAAAGATGTGACAGACG
CGACGGCGACAAGCAAACATGCTGTGCGACGCTGGCGATATCAAAATTGCTGTCTGCCAGGTGATCGCTGATGTACTGACAAG
CCTCGCGTACCCGATTATCCATCGGTGGATGGAGCGACTCGTTAATCGCTTCCATGCGCCGCAGTAACAATTGCTCAAGCAGA
TTTATCGCCAGCAGCTCCGAATAGCGCCCTTCCCCTTGCCCGGCGTTAATGATTTGCCCAAACAGGTCGCTGAAATGCGGCTG
GTGCGCTTCATCCGGGCGAAAGAACCCCGTATTGGCAAATATTGACGGCCAGTTAAGCCATTCATGCCAGTAGGCGCGCGGAC
GAAAGTAAACCCACTGGTGATACCATTCGCGAGCCTCCGGATGACGACCGTAGTGATGAATCTCTCCTGGCGGGAACAGCAAA
ATATCACCCGGTCGGCAAACAAATTCTCGTCCCTGATTTTTCACCACCCCCTGACCGCGAATGGTGAGATTGAGAATATAACC
TTTCATTCCCAGCGGTCGGTCGATAAAAAAATCGAGATAACCGTTGGCCTCAATCGGCGTTAAACCCGCCACCAGATGGGCAT
TAAACGAGTATCCCGGCAGCAGGGGATCATTTTGCGCTTCAGCCATACTTTTCATACTCCCGCCATTCAGAG**AAGAAACCAAT**
TGTCCATATTGCATCAGACATTGCCGTCACTGCGTCTTTTACTGGCTCTTCTCGCTAACCAAAACCGGTAACCCCGCTTATTA
AAAGCATTCTGTAACAAAGCGGGACCAAAGCCATGACAAAAACGCGTAACAAAAGTGTCTATAATCACGGCAGAAAAGTCCAC
ATTGATTATTTGCACGGCGTCACACTTTGCTATGCCATAGCATTTTTATCCATAAGATTAGCGGATCCTACCTGACGCTTTTT
ATCGCAACTCTCTACTGTTTCTCCATACCCGTTTTTTTGGGCTAGAAATAATTTTGTTTAACTTTAAGAAGGAGATATACATA
TGGCATAGGTTATCAACACGTTTGATGGGGTTGCGGATTATCTTCAGACATATCATAAGCTACCTGATAATTACATTACAAAA
TCAGAAGCACAAGCCCTCGGCTGGGTGGCATCAAAAGGGAACCTTGCATAGGTCGCTCCGGGGAAAAGCATCGGCGGAGACAT
CTTCTCAAACAGGGAAGGCAAACTCCCGTAGAAAAGCGGACGAACATGGCGTGAAGCGGATATTAACTATACATCAGGCTTCA
GAAATTCAGACCGGATTCTTTACTCAAGCGACTGGCTGATTTACAAAACAACGGACCATTATCAGACCTTTACAAAAATCAGA
TAAGCATGCACCATTCCTTGCGGCGGCGGTGCTCAACGGCCTCAACCTACTACTGGGCTGCTTCGGC

DNA sequence of pBARN-3C11-chPheT$_{CUA}$:

The pBARN-3C11-chPheT$_{CUA}$ plasmid contains a p15A type origin of replication, a chloramphenicol resistance gene (CamR), and an 3C11-chPheT$_{CUA}$ under control of a proK promoter.

3C11-chPheT$_{CUA}$; **CamR**; **p15A ori**; araBAD-barnase(3TAG, 45TAG, 66TAG).

GGCACCTCGCTAACGGACGCCTGAATAAGTGATAATAAGCGGATGAATGGCAGAAATTCGAAAGCAAATTCGACCCTGAGCTG
CTCGAGCATGCAAGGCATTTTGCTATTAAGGGATTGACGAGGGCGTATCTGCGCAGTAAGATGCGCCCCGCATT**GTGAGAGTG**
ATCATGTAGATCGAACGGACTCTAAATCCGTTCAGCCGGGTTAGATTCCCGGCTCTCACACCAAATTCGAAAAGCCTGCTCAA
CGAGCAGGCTTTTTTGCATGCTCGAGCAGCTCAGGGTCGAATTTGCTTTCGAATTTCTGCCATTCATCCGCTTATTATCACTT
ATTCAGGCGTAGCAACCAGGCGTTTAAGGGCACCAATAACTGCCTTAAAAAAA**TTACGCCCCGCCCTGCCACTCATCGCAGTA**
CTGTTGTAATTCATTAAGCATTCTGCCGACATGGAAGCCATCACAAACGGCATGATGAACCTGAATCGCCAGCGGCATCAGCA
CCTTGTCGCCTTGCGTATAATATTTGCCCATGGTGAAAACGGGGGCGAAGAAGTTGTCCATATTGGCCACGTTTAAATCAAAA
CTGGTGAAACTCACCCAGGGATTGGCTGAGACGAAAAACATATTCTCAATAAACCCTTTAGGGAAATAGGCCAGGTTTTCACC
GTAACACGCCACATCTTGCGAATATATGTGTAGAAACTGCCGGAAATCGTCGTGGTATTCACTCCAGAGCGATGAAAACGTTT
CAGTTTGCTCATGGAAAACGGTGTAACAAGGGTGAACACTATCCCATATCACCAGCTCACCGTCTTTCATTGCCATACGGAAT
TCCGGATGAGCATTCATCAGGCGGGCAAGAATGTGAATAAAGGCCGGATAAAACTTGTGCTTATTTTTCTTTACGGTCTTTAA
AAAGGCCGTAATATCCAGCTGAACGGTCTGGTTATAGGTACATTGAGCAACTGACTGAAATGCCTCAAAATGTTCTTTACGAT
GCCATTGGGATATATCAACGGTGGTATATCCAGTGATTTTTTTCTCCATTTTAGCTTCCTTAGCTCCTGAAAATCTCGATAAC
TCAAAAAATACGCCCGGTAGTGATCTTATTTCATTATGGTGAAAGTTGGAACCTCTTACGTGCCGATCAACGTCTCATTTTCG
CCAAAAGTTGGCCCAGGGCTTCCCGGTATCAACAGGGACACCAGGATTTATTTATTCTGCGAAGTGATCTTCCGTCACAGGTA
TTTATTCGGCGCAAAGTGCGTCGGGTGATGCTGCCAACTTACTGATTTAGTGTATGATGGTGTTTTTGAGGTGCTCCAGTGGC
TTCTGTTTCTATCAGCTGTCCCTCCTGTTCAGCTACTGACGGGGTGGTGCGTAACGGCAAAAGCACCGCCGGACATCAGCGCT
AGCGGAGTGTATACTGGCTTACTATGTTGGCACTGATGAGGGTGTCAGTGAAGTGCTTCATGTGGCAGGAGAAAAAAGGCTGC
ACCGGTGCGTCAGCAGAATATGTGATACAGGATATATTCCGCTTCCTCGCTCACTGACTCGCTACGCTCGGTCGTTCGACTGC
GGCGAGCGGAAATGGCTTACGAACGGGGCGGAGATTTCCTGGAAGATGCCAGGAAGATACTTAACAGGGAAGTGAGAGGGCCG
CGGCAAAGCCGTT**TTTCCATAGGCTCCGCCCCCCTGACAAGCATCACGAAATCTGACGCTCAAATCAGTGGTGGCGAAACCCG**
ACAGGACTATAAAGATACCAGGCGTTTCCCCCTGGCGGCTCCCTCGTGCGCTCTCCTGTTCCTGCCTTTCGGTTTACCGGTGT
CATTCCGCTGTTATGGCCGCGTTTGTCTCATTCCACGCCTGACACTCAGTTCCGGGTAGGCAGTTCGCTCCAAGCTGGACTGT

ATGCACGAACCCCCCGTTCAGTCCGACCGCTGCGCCTTATCCGGTAACTATCGTCTTGAGTCCAACCCGGAAAGACATGCAAA
AGCACCACTGGCAGCAGCCACTGGTAATTGATTTAGAGGAGTTAGTCTTGAAGTCATGCGCCGGTTAAGGCTAAACTGAAAGG
ACAAGTTTTGGTGACTGCGCTCCTCCAAGCCAGTTACCTCGGTTCAAAGAGTTGGTAGCTCAGAGAACCTTCGAAAAACCGCC
CTGCAAGGCGGTTTTTTCGTTTTCAGAGCAAGAGATTACGCGCAGACCAAAACGATCTCAAGAAGATCATCTTATTAATCAGA
TAAAATATTTCTAGATTTCAGTGCAATTTATCTCTTCAAATGTAGCACCTGAAGTCAGCCCCATACGATATAAGTTGTAATTC
TCATGTTTGACAGCATTATCATCGATAAGCTTTAATGCGGTAGTTTATCACAGTTAAATTGCTAACGCAGTCAGGCACCGTGT
ATGAAATCTAACAATGCGCTCATCGTCATCCTCGGCACCGTCACCCTGGATGCTGTAGGCATAGGCTTGGTTATGCCGGTACT
GCCGGGCCTCTTGCGGGATGGCCACGATGCGTCCGGCGTAGAGGATCTGCTCATGTTTGACAGCTTATCATCGATGCATAATG
TGCCTGTCAAATGGACGAAGCAGGGATTCTGCAAACCCTATGCTACTCCGTCAAGCCGTCAATTGTCTGATTCGTTACCAATT
ATGACAACTTGACGGCTACATCATTCACTTTTTCTTCACAACCGGCACGGAACTCGCTCGGGCTGGCCCCGGTGCATTTTTTA
AATACCCGCGAGAAATAGAGTTGATCGTCAAAACCAACATTGCGACCGACGGTGGCGATAGGCATCCGGGTGGTGCTCAAAAG
CAGCTTCGCCTGGTGATACGTTGGTCCTCGCGCCAGCTTAAGACGCTAATCCCTAACTGCTGGCGGAAAAGATGTGACAGACG
CGACGGCGACAAGCAAACATGCTGTGCGACGCTGGCGATATCAAAATTGCTGTCTGCCAGGTGATCGCTGATGTACTGACAAG
CCTCGCGTACCCGATTATCCATCGGTGGATGGAGCGACTCGTTAATCGCTTCCATGCGCCGCAGTAACAATTGCTCAAGCAGA
TTTATCGCCAGCAGCTCCGAATAGCGCCCTTCCCCTTGCCCGGCGTTAATGATTTGCCCAAACAGGTCGCTGAAATGCGGCTG
GTGCGCTTCATCCGGGCGAAAGAACCCCGTATTGGCAAATATTGACGGCCAGTTAAGCCATTCATGCCAGTAGGCGCGCGGAC
GAAAGTAAACCCACTGGTGATACCATTCGCGAGCCTCCGGATGACGACCGTAGTGATGAATCTCTCCTGGCGGGAACAGCAAA
ATATCACCCGGTCGGCAAACAAATTCTCGTCCCTGATTTTTCACCACCCCCTGACCGCGAATGGTGAGATTGAGAATATAACC
TTTCATTCCCAGCGGTCGGTCGATAAAAAAATCGAGATAACCGTTGGCCTCAATCGGCGTTAAACCCGCCACCAGATGGGCAT
TAAACGAGTATCCCGGCAGCAGGGGATCATTTTGCGCTTCAGCCATACTTTTCATACTCCCGCCATTCAGAG**AAGAAACCAAT**
TGTCCATATTGCATCAGACATTGCCGTCACTGCGTCTTTTACTGGCTCTTCTCGCTAACCAAAACCGGTAACCCCGCTTATTA
AAAGCATTCTGTAACAAAGCGGGACCAAAGCCATGACAAAAACGCGTAACAAAAGTGTCTATAATCACGGCAGAAAAGTCCAC
ATTGATTATTTGCACGGCGTCACACTTTGCTATGCCATAGCATTTTTATCCATAAGATTAGCGGATCCTACCTGACGCTTTTT
ATCGCAACTCTCTACTGTTTCTCCATACCCGTTTTTTTGGGCTAGAAATAATTTTGTTTAACTTTAAGAAGGAGATATACATA
TGGCATAGGTTATCAACACGTTTGATGGGGTTGCGGATTATCTTCAGACATATCATAAGCTACCTGATAATTACATTACAAAA
TCAGAAGCACAAGCCCTCGGCTGGGTGGCATCAAAAGGGAACCTTGCATAGGTCGCTCCGGGGAAAAGCATCGGCGGAGACAT
CTTCTCAAACAGGGAAGGCAAACTCCCGTAGAAAAGCGGACGAACATGGCGTGAAGCGGATATTAACTATACATCAGGCTTCA
GAAATTCAGACCGGATTCTTTACTCAAGCGACTGGCTGATTTACAAAACAACGGACCATTATCAGACCTTTACAAAAATCAGA
TAAGCATGCACCATTCCTTGCGGCGGCGGTGCTCAACGGCCTCAACCTACTACTGGGCTGCTTCGGC

DNA sequence of pOS1T-*ch*PheRS^{4PyA}-3C11-chPheT$_{CUA}$:

The pOS1T plasmids contains a p15A type origin of replication, a tetracycline resistance gene (TetR), a tRNA under control of a proK promoter and an aaRS under control of a OXB20 promoter.

*ch*PheRS^{4PyA}; p15a ori; TetR; 3C11-chPheT$_{CUA}$.

ATGGATAAGAAGCCGCTGGATGTACTGATCTCTGCGACCGGcCTGTGGATGTCCCGTACCGGCACGATGCACAAGATCAAGCA
CTATGAGATTTCACGTTCTAAAATCTACATCGAAATGGCGTGTGGTGACCATCTGGTTGGGAACAACTCTCGTTCTTGTCGTC
CCGCACGTGCATTCCGTTATCATAAATACCGTAAAACCTGCAAACGTTGTCGTGTTTCTGACGAAGATATCAACAACTTCCTG
GCCCGTTCTACCGAAGGCAAAACCTCTGTTAAAGTTAAAGTTGTTTCTGAGCCGAAAGTGAAAAAAGCGATGCCGAAATCTGT
TTCTCGTGCGCTGAAACCGCTGGAAAATCCGGTTTCTGCGAAAGCGCCTACCGACACCTCTCATTCTGTTCCGTCTCCGGCGA
AATCTACCCCGAACTCTCCGGTTCCGACCCCTGCAAGCGCCCCAGCTCGGACTAAATCCCAGACGGACCGTCTGGAGGTGCTG
CTGAACCCAAAGGATGAAATCTCTCTGAACAGCGGCAAGCCTTTCCGTGAGCTGGAAAGCGAGCTGCTGTCTCGTCGTAAAAA
GGATCTGCAACAGATCTACGCTGAGGAACGCGAGGGTGGCGGAAGCGGTGGCGGAAGCGGTGGCGGAAGTGGTGGCGGAAGCG
GCGGCGGAAGCCAGGCCTGGGGATCCAGGCCTCCTGCAGCAGAGTGTGCCACCCAAGGAGCTCCAGGCAGTGTGGTGGAGTTG
CTGGGCAAATCCTACCCTCAGGACGACCTCAGCAACCTCACCCGGAAGGTCCTCACCAGAgtcGGCAGGAACCTTCACAACCA
GCAGCATCACCCTCTGTGTCTGATCAAGGAGAGGGTGAAGGAGCACTTCTACAAGCAGTATGTGGGCCGCTTTGGACCCCGT
TGTTCTCTGTCTACGACAACCTTTCTCCAGTGGTCACGACCTGGCAGAACTTTGACAGCCTGCTCATCCCAGCTGATCACCCC
AGCAGGAAGAAGGGGGACAACTATTACCTGAATCGGACTCACATGCTGAGAGCGCACACGTCCGCGCACaatTGGGACTTGCT
GCACGCGGGACTGGATGCCTTCCTGGTGGTGGGTGATGTCTACAGGCGTGACCAGATCGACTCCCAGCACTACCCTATATTCC
ACCAGCTGGAGGCCGTGCGGCTCTTCTCCAAGCATGAGTTATTTGCTGGTATAAAGGATGGAGAAAGCTTGCAGCTCTTTGAA
CAAAGTTCTCGCTCTGCGCATAAACAAGAGACACACACCATGGAGGCCGTGAAGCTTGTAGAGTTTGATCTTAAGCAAACGCT
TACCAGGCTCATGGCACATCTTTTTGGAGATGAGCTGGAGATAAGGTGGGTAGACTGCTACTTtCCTTTTacgCATCCTTCCT
TTGAGATGGAGATCAACTTTCATGGAGAATGGCTGGAAGTACTTGGCTGCGGGGTGATGGAACAACAACTGGTCAATTCtGCT
GGTGCTcagGACCGAATCGGCTGGTCGATTGGCCTAGGgTTAGAAAGGCTAGCCATGATCCTCTACGACATCCCTGATATCCG
TCTCTTCTGGTGTGAGGACGAGCGCTTCCTGAAGCAGTTCTGTGTATCCAACATTAATCAGAAGGTGAAGTTTCAGCCTCTTA
GCAAATAAAGGTGGCACTTTTCGGGGCAGTTTCAAACGGGTACCATATGGGAATTCGAAGCTTGGGCCCGAACAAAAACTCAT
CTCAGAAGAGGATCTGAATAGCGCCGTCGACCATCATCATCATCATCATTGAGTTTAAACGGTCTCCAGCTTGGCTGTTTTGG
CGGATGAGAGAAGATTTTCAGCCTGATACAGATTAAATCAGAACGCAGAAGCGGTCTGATAAAACAGAATTTGCCTGGCGGCA
GTAGCGCGGTGGTCCCACCTGACCCCATGCCGAACTCAGAAGTGAAACGCCGTAGCGCCGATGGTAGTGTGGGGTCTCCCCAT
GCGAGAGTAGGGAACTGCCAGGCATCAAATAAAACGAAAGGCTCAGTCGAAAGACTGGGCCTTTCGTTTTATCTGTTGTTTGT
CGGTGAACGCTCTCCTGAGTAGGACAAATCCGCCGGGAGCTGTCCCTCCTGTTCAGCTACTGACGGGGTGGTGCGTAACGGCA
AAAGCACCGCCGGACATCAGCGCTAGCGGAGTGTATACTGGCTTACTATGTTGGCACTGATGAGGGTGTCAGTGAAGTGCTTC
ATGTGGCAGGAGAAAAAAGGCTGCACCGGTGCGTCAGCAGAATATGTGATACAGGATATATTCCGCTTCCTCGCTCACTGACT
CGCTACGCTCGGTCGTTCGACTGCGGCGAGCGGAAATGGCTTACGAACGGGGCGGAGATTTCCTGGAAGATGCCAGGAAGATA
CTTAACAGGGAAGTGAGAGGGCCGCGGCAAAGCCGTTTTTCCATAGGCTCCGCCCCCCTGACAAGCATCACGAAATCTGACGC

TCAAATCAGTGGTGGCGAAACCCGACAGGACTATAAAGATACCAGGCGTTTCCCCCTGGCGGCTCCCTCGTGCGCTCTCCTGT
TCCTGCCTTTCGGTTTACCGGTGTCATTCCGCTGTTATGGCCGCGTTTGTCTCATTCCACGCCTGACACTCAGTTCCGGGTAG
GCAGTTCGCTCCAAGCTGGACTGTATGCACGAACCCCCCGTTCAGTCCGACCGCTGCGCCTTATCCGGTAACTATCGTCTTGA
GTCCAACCCGGAAAGACATGCAAAAGCACCACTGGCAGCAGCCACTGGTAATTGATTTAGAGGAGTTAGTCTTGAAGTCATGC
GCCGGTTAAGGCTAAACTGAAAGGACAAGTTTTGGTGACTGCGCTCCTCCAAGCCAGTTACCTCGGTTCAAAGAGTTGGTAGC
TCAGAGAACCTTCGAAAAACCGCCCTGCAAGGCGGTTTTTTCGTTTTCAGAGCAAGAGATTACGCGCAGACCAAAACGATCTC
AAGAAGATCATCTTATTAATCAGATAAAATATTTCTAGATTTCAGTGCAATTTATCTCTTCAAATGTAGCACCTGAAGTCAGC
CCCATACGATATAAGTTGTAATTCTCATGTTGATCGGCACGTAATAGCGGGGCGTAAATGGAAGCCGGCGGCGCTGGTAGCAA
AATTCCTCGACGAACGTGGGATCGTAGTAGAGAAAACCGGCCCTTATAACCTGCTGTTTCTCTTTAGTATTGGCATCGATAAA
ACCAAAGCAATGGGATTATTGCGTGGGTTGACGGAATTCAAACGCTCTTACGATCTCAACCTGCGGATCAAAAATATGCTACC
CGATCTCTATGCAGAAGATCCCGATTTCTACCGCAATATGCGTATTCAGGATCTGGCACAAGGGATCCATAAGCTGATTCGTA
AACACGATCTTCCCGGTTTGATGTTGCGGGCATTCGATACTTTGCCGGAGATGATCATGACGCCACATCAGGCATGGCAACGA
CAAATTAAAGGCGAAGTAGAAACCATTGCGCTGGAACAACTGGTCGGTAGAGTATCGGCAAATATGATCCTGCCTTATCCACC
GGGCGTACCGCTGTTGATGCCTGGAGAAATGCTGACCAAAGAGAGCCGCACAGTACTCGATTTTCTACTGATGCTTTGTTCCG
TCGGGCAACATTACCCCGGTTTTGAAACGGATATTCACGGCGCGAAACAGGACGAAGACGGCGTTTACCGCGTACGAGTCCTA
AAAATGGCGGGATAACTTGCCAGAGCGGCTTCCATTTACGCCCCGCCCTGTTGACAGCTTATCATCGATAAGCTTTAATGCGG
TAGTTTATCACAGTTAAATTGCTAACGCAGTCAGGCACCGTGTATGAAATCTAACAATGCGCTCATCGTCATCCTCGGCACCG
TCACCCTGGATGCTGTAGGCATAGGCTTGGTTATGCCGGTACTGCCGGGCCTCTTGCGGGATATCGTCCATTCCGACAGCATC
GCCAGTCACTATGGCGTGCTGCTAGCGCTATATGCGTTGATGCAATTTCTATGCGCACCCGTTCTCGGAGCACTGTCCGACCG
CTTTGGCCGCCGCCCAGTCCTGCTCGCTTCCCTACTTGGAGCCACTATCGACTACGCGATCATGGCGACCACACCCGTCCTGT
GGATCCTCTACGCCGGACGCATCGTGGCCGGCATCACCGGCGCCACAGGTGCGGTTGCTGGCGCCTATATCGCCGACATCACC
GATGGGGAAGATCGGGCTCGCCACTTCGGGCTCATGAGCGCTTGTTTCGGCGTGGGTATGGTGGCAGGCCCCGTGGCCGGGGG
ACTGTTGGGCGCCATCTCCTTGCATGCACCATTCCTTGCGGCGGCGGTGCTCAACGGCCTCAACCTACTACTGGGCTGCTTCC
TAATGCAGGAGTCCCATAAGGGAGAGCGTCGACCGATGCCCTTGAGAGCCTTCAACCCAGTCAGCTCCTTCCGGTGGGCGCGG
GGCATGACTATCGTCGCCGCACTTATGACTGTCTTCTTTATCATGCAACTCGTAGGACAGGTGCCGGCAGCGCTCTGGGTCAT
TTTCGGCGAGGACCGCTTTCGCTGGAGCGCGACGATGATCGGCCTGTCCCTTGCGGTATTCGGAATCTTGCACGCCCTCGCTC
AAGCCTTCGTCACTGGTCCCGCCACCAAACGTTTCGGCGAGAAGCAGGCCATTATCGCCGGCATGGCGGCCGACGCGCTGGGC
TACGTCTTGCTGGCGTTCGCGACGCGAGGCTGGATGGCCTTCCCCATTATGATTCTTCTCGCTTCCGGCGGCATCGGGATGCC
CGCGTTGCAGGCCATGCTGTCCAGGCAGGTAGATGACGACCATCAGGGACAGCTTCAAGGATCCCTCGCGGCTCTTACCAGCC
TAACTTCCATCATTGGACCGCTGATCGTCACGGCGATTTATGCCGCCTCCGCGAGCACATGGAACGGGTTGGCATGGATTGTA
GGCGCCGCCCTATACCTTGTCTGCCTCCCCGCGTTGCGTCGCGGTGCATGGAGCCGGGCCACCTCCACCTGAAGGGCGGGGCG
TAAATGGAAGCCGGCGGCACCTCGCTAACGGATTCACCACTCCAAGAATTGGAGCCAATCAATTCTTGCGGAGAACTGTGAAT
GCGCAAACCAACCCTTGGCAGAACATATCCATCGCGTCCGCCATCTCCAGCAGCCGCACGCGGCGCATCTCGGGCTCCTTGCA
TGCACCATTCCTTGCGGCGGCGGTGCTCAACGGCCTCAACCTACTACTGGGCTGCTTCCTAATGCAGGAGTCGCATAAGGGAG
AGCGTCTGGCGAAAAAAAAGCCTGCTCGTTGAGCAGGCTTTTCGAATTGTGAGAGTGATCATGTAGATCGAACGGACTCTAAA
TCCGTTCAGCCGGGTTAGATTCCCGGCTCTCACACCAAATGCGGGGCGCATCTTACTGCGCAGATACGCCCTCGTCAATCCCT
TAATAGCAAAATGCCTCCTGCACCATTATGTTCCGGATCTGCATCGCAGGATGCTGCTGGCTACCCTGTGGAACACCTACATC
TGTATTAACGAAGCGCTAACCGTTTTTATCATGCTCTGGGAGGCAGAATAAATGATCATATCGTCAATTATTACCTCCACGGG
GAGAGCCTGAGCAAACTGGCCTCAGGCATTTGAGAAGCACACGGTCACACTGCTTCCGGTAGTCAATAAACCGGTAAACCAGC
AATAGACATAAGCGGCTATTTAACGAGAACGACCGAGCGCAGCGAGTCAGTGAGCGAGGAAGCGGAAGAGCGAACTTTTGCTG
AGTTGAAGGATCCTCGGGTTGTCAGCCTGTCCCGCTTATAAGATCATACGCCGTTATACGTTGTTTACGCTTTGAGGAATCCC
AT

DNA sequence of pOS1Tcc-*G1*PylRS^{MIFAF}-*Ma*tRNA^{Pyl}(8)$_{UCA}$:

The pOS1Tcc-*G1*PylRS^{MIFAF}-*Ma*tRNA^{Pyl}(8)$_{UCA}$ plasmid contains a CloDF13 origin of replication, a chloramphenicol resistance gene (CamR), *G1*PylRS^{MIFAF} under control of a OXB20 promoter and a *Ma*tRNA^{Pyl}(8)$_{UCA}$ under control of a proK promoter.

*G1*PylRS^{MIFAF}; CloDF13 ori; CamR; *Ma*tRNA^{Pyl}(8)$_{UCA}$.

TTACGCTTTGAGGAATCCCATATGGTGGTCAAGTTTACTGATTCCCAAATTCAACACCTTATGGAGTATGGCGATAATGATTG
GTCAGAGGCCGAATTCGAAGACGCTGCTGCACGCGACAAAGAGTTCTCCAGTCAATTTTCCAAACTGAAGTCTGCAAATGATA
AAGGGCTGAAAGACGTTATTGCCAACCCTCGCAACGATTTAACCGACCTTGAAAACAAGATCCGTGAAAAACTTGCAGCGCGC
GGTTTTATTGAGGTGCACACTCCCATCTTTGTTAGTAAGTCAGCCTTAGCGAAAATGACTATTACCGAGGACCACCCGCTTTT
TAAGCAAGTTTTTTGGATCGATGATAAACGTGCGTTGCGTCCGATGATGGCTATGAATATCTTTAAAGTTGCTCGCGAGTTAC
GCGATCACACCAAAGGCCCGGTTAAGATTTTTGAGATCGGTTCGTGCTTTCGCAAAGAGTCCAAGTCATCTACCCATTTGGAG
GAGTTTACAATGTTAAATCTGTTTGAAATGGGGCCAGACGGGGATCCTATGGAGCATCTTAAAATGTACATTGGGGACATCAT
GGACGCGGTAGGCGTTGAATACACGACCTCCCGCGAAGAAAGTGATGTATATGTTGAAACCTTGGATGTGGAAATCAATGGTA
CAGAGGTAGCCAGTGGCGCTGTCGGCCCCCACAAATTAGACCCCGCGCACGACGTGCATGAGCCTTGGGCAGGTATCGGTTTC
GGGCTTGAGCGCTTACTTATGCTTAAAAATGGAAAATCAAATGCGCGTAAGACCGGGAAATCTATCACTTATCTTAACGGATA
CAAGTTAGACTAAAAGGTGGCACTTTTCGCTGCAGTTTCAAACGGGTACCATATGGGAATTCGAAGCTTGGGCCCGAACAAAA
ACTCATCTCAGAAGAGGATCTGAATAGCGCCGTCGACCATCATCATCATCATCATTGAGTTTAAACGGTCTCCAGCTTGGCTG
TTTTGGCGGATGAGAGAAGATTTTCAGCCTGATACAGATTAAATCAGAACGCAGAAGCGGTCTGATAAAACAGAATTTGCCTG
GCGGCAGTAGCGCGGTGGTCCCACCTGACCCCATGCCGAACTCAGAAGTGAAACGCCGTAGCGCCGATGGTAGTGTGGGGTCT
CCCCATGCGAGAGTAGGGAACTGCCAGGCATCAAATAAAACGAAAGGCTCAGTCGAAAGACTGGGCCTTTCGTTTTATCTGTT
GTTTGTCGGTGAACGCTCTCCTGAGTAGGACAAATCCGCCGGGAGCTGTCCCTCCTGTTCAGCTACTGACGGGGTGGTGCGTA

ACGGCAAAAGCACCGCCGGACATCAGCGCTAGCGGAGTGTATACTGGCTTACTATGTTGGCACTGATGAGGGTGTCAGTGAAG
TGCTTCATGTGGCAGGAGAAAAAAGGCTGCACCGGTGCGTCAGCAGAATATGTGATACAGGATATATTCCGCTTCCTCGCTCA
CTGACTCGCTACGCTCGGTCGTTCGACTGCGGCGAGCGGAAATGGCTTACGAACGGGGCGGAGATTTCCTGGAAGATGCCAGG
AAGATACTTAACAGGGAAGTGAGAGGGCCACTCATTAGGCACCGGGATCTCGACCGATGCCCTTGAGAGCCTTCAACCCAGTC
AGCTCCTTCCGGTGGGCGCGGGGCATGACTAACATGAGAATTACAACTTATATCGTATGGGGCTGACTTCAGGTGCTACATTT
GAAGAGATAAATTGCACTGAAATCTAGAGCGGTTCAGTAGAAAAGATCAAAGGATCTTCTTGAGATCCTTTTTTTCTGCGCGT
AATCTTTTGCCCTGTAAACGAAAAAACCACCTGGGGAGGTGGTTTGATCGAAGGTTAAGTCAGTTGGGGAACTGCTTAACCGT
GGTAACTGGCTTTCGCAGAGCACAGCAACCAAATCTGTCCTTCCAGTGTAGCCGGACTTTGGCGCACACTTCAAGAGCAACCG
CGTGTTTAGCTAAACAAATCCTCTGCGAACTCCCAGTTACCAATGGCTGCTGCCAGTGGCGTTTTACCGTGCTTTTCCGGGTT
GGACTCAAGTGAACAGTTACCGGATAAGGCGCAGCAGTCGGGCTGAACGGGGAGTTCTTGCTTACAGCCCAGCTTGGAGCGAA
CGACCTACACCGAGCCGAGATACCAGTGTGTGAGCTATGAGAAAGCGCCACACTTCCCGTAAGGGAGAAAGGCGGAACAGGTA
TCCGGTAAACGGCAGGGTCGGAACAGGAGAGCGCAAGAGGGAGCGACCCGCCGGAAACGGTGGGGATCTTTAAGTCCTGTCGG
GTTTCGCCCGTACTGTCAGATTCATGGTTGAGCCTCACGGCTCCCACAGATGCACCGGAAAAGCGTCTGTTTATGTGAACTCT
GGCAGGAGGGCGGAGCCTATGGAAAAACGCCACCGGCGCGGCCCTGCTGTTTTGCCTCACATGTTAGTCCCCTGCTTATCCAC
GGAATCTGTGGGTAACTTTGTATGTGTCCGCAGCGCCCGCCGCAGTCTCACGCCCGGAGCGTAGCGACCGAGTGAGCTAGCTA
TGATCGGCACGTAAGAGGTTCCAACTTTCACCATAATGAAATAAGATCACTACCGGGCGTATTTTTTGAGTTATCGAGATTTT
CAGGAGCTAAGGAAGCTAAAATGGAGAAAAAAATCACTGGATATACCACCGTTGATATATCCCAATGGCATCGTAAAGAACAT
TTTGAGGCATTTCAGTCAGTTGCTCAATGTACCTATAACCAGACCGTTCAGCTGGATATTACGGCCTTTTTAAAGACCGTAAA
GAAAAATAAGCACAAGTTTTATCCGGCCTTTATTCACATTCTTGCCCGCCTGATGAATGCTCATCCGGAATTCCGTATGGCAA
TGAAAGACGGTGAGCTGGTGATATGGGATAGTGTTCACCCTTGTTACACCGTTTTCCATGAGCAAACTGAAACGTTTTCATCG
CTCTGGAGTGAATACCACGACGATTTCCGGCAGTTTCTACACATATATTCGCAAGATGTGGCGTGTTACGGTGAAAACCTGGC
CTATTTCCCTAAAGGGTTTATTGAGAATATGTTTTTCGTCTCAGCCAATCCCTGGGTGAGTTTCACCAGTTTTGATTTAAACG
TGGCCAATATGGACAACTTCTTCGCCCCCGTTTTCACCATGGGCAAATATTATACGCAAGGCGACAAGGTGCTGATGCCGCTG
GCGATTCAGGTTCATCATGCCGTTTGTGATGGCTTCCATGTCGGCAGAATGCTTAATGAATTACAACAGTACTGCGATGAGTG
GCAGGGCGGGGCGTAATGTCTAACAATTCGTTCAAGCCGAGGGGCCGCAAGATCCGGCCACGATGACCCATGGTTAGCAAAGG
TGAACGGCACCTCGCTAACGGAAACCTACTACTGGGCTGCTTCCTAATGCAGGAGTCGCATAAGGGAGAGCGTCTGGCGAAAA
AAAAGCCTGCTCGTTGAGCAGGCTTTTCGAATTTGGCGAGAGACCGGGGAGTCGAACCCCGCTCCTGCTGAGTTTAGAGACC
CGCTGGTCGCCGGACCGTCCCCCAATGCGGGGCGCTTGTTATCCGCTCACAATTATCTTACTGCGCAGATACGCCCTCGTCAA
TCCCTTAATAGCAAAATGCCTCCTGCACCATTATGTTCCGGATCTGCATCGCAGGATGCTGCTGGCTACCCTGTGGAACACCT
ACATCTGTATTAACGAAGCGCTAACCGTTTTTATCATGCTCTGGGAGGCAGAATAAATGATCATATCGTCAATTATTACCTCC
ACGGGGAGAGCCTGAGCAAACTGGCCTCAGGCATTTGAGAAGCACACGGTCACACTGCTTCCGGTAGTCAATAAACCGGTAAA
CCAGCAATAGACATAAGCGGCTATTTAACGACCCTGCCCTGAACCGACGACCGGGTCGAATTTGCTTTCGAATTTCTGCCATT
CATCCGCTTATTATCACTTATTCAGGCGTAGCAACCAGGCGTTTAAGGGCACCAATAACTGCCTTAAAAAAATTACGCCCCGC
CCTGCCACTCATCGCAGTTGACTGGGTCATGGCTGCGCCCCGACACCCGCCAACACCCGCTGACGCGCCCTGACGGGCTTGTC
TGCTCCCGGCATCCGCTTACAGACAAGCTGTGACCGTCTCCGGGAGCTGCATGTGTCAGAGGTTTTCACCGTCATCACCGAAA
CGCGCGAGGCAGCAGATCAATTCGCGCGCGAAGGCGAAGCGGCATGCATAATGTGCCTGTCAAATGGACGAAGCAGGGATTCT
GCAAACCCTATGCTACTCCGTCAAGCCGTCAATTGTCTGATTCGTTACCAAAAGCTGTTGTGACCGCTTGCTCTAGCCAGCTA
TCGAGTTGTGAACCGATCCATCTAGCAATTGGTCTCGATCTAGCGATAGGCTTCGATCTAGCTATGTAGAAACGCCGTGTGCT
CGATCGCTTGATAAGGTCCACGTAGCTGCTATAATTGCTTCAACAGAACATATTGACTATCCGGTATTACCCGGCCGCCGTTA
TACGTTGT

DNA sequence of pSLdT7-*Mb*(IPYE)PylRS^{4ThzA}:

The pSLdT7-Mb(IPYE)PylRS^{4ThzA} plasmid contains a pBR322 type origin of replication, a kanamycin resistance gene (KanR), and an *Mb*(IPYE)PylRS^{4ThzA} under control of a glnS promoter. The aaRS expression cassette is flanked by two T7 promoters as well as two T7 terminators, essentially as described previously[18].

Mb(IPYE)PylRS^{4ThzA}; KanR; pBR322 ori; T7 promoter; T7 terminator;

AACACCCCTTGTATTACTGTTTATGTAAGCAGACAGTTTTATTGTTCATGACCAAAATCCCTTAACGTGAGTTTTCGTTCCAC
TGAGCGTCAGACCCCGTAGAAAAGATCAAAGGATCTTCTTGAGATCCTTTTTTTCTGCGCGTAATCTGCTGCTTGCAAACAAA
AAAACCACCGCTACCAGCGGTGGTTTGTTTGCCGGATCAAGAGCTACCAACTCTTTTTCCGAAGGTAACTGGCTTCAGCAGAG
CGCAGATACCAAATACTGTCCTTCTAGTGTAGCCGTAGTTAGGCCACCACTTCAAGAACTCTGTAGCACCGCCTACATACCTC
GCTCTGCTAATCCTGTTACCAGTGGCTGCTGCCAGTGGCGATAAGTCGTGTCTTACCGGGTTGGACTCAAGACGATAGTTACC
GGATAAGGCGCAGCGGTCGGGCTGAACGGGGGGTTCGTGCACACAGCCCAGCTTGGAGCGAACGACCTACACCGAACTGAGAT
ACCTACAGCGTGAGCTATGAGAAAGCGCCACGCTTCCCGAAGGGAGAAAGGCGGACAGGTATCCGGTAAGCGGCAGGGTCGGA
ACAGGAGAGCGCACGAGGGAGCTTCCAGGGGGAAACGCCTGGTATCTTTATAGTCCTGTCGGGTTTCGCCACCTCTGACTTGA
GCGTCGATTTTTGTGATGCTCGTCAGGGGGGCGGAGCCTATGGAAAAACGCCAGCAACGCGGCCTTTTTACGGTTCCTGGCCT
TTTGCTGGCCTTTTGCTCACATGTTCTTTCCTGCGTTATCCCCTGATTCTGTGGATAACCGTATTACCGCCTTTGAGTGAGCT
GATACCGCTCGCCGCAGCCGAACGACCGAGCGCAGCGAGTCAGTGAGCGAGGAAGCGGAAGAGTAGTTCCTCCTTTCAGCAAA
AAACCCCTCAAGACCCGTTTAGAGGCCCCAAGGGGTTATGCTAGTTATTGCTCAGCGGTGGATGCGTCCGGCGTAGAGGATCG
AGATCTCGATCCCGCGAAATTAATACGACTCACTATAGGAACTTTTGCTGAGTTGAAGGATCCTCGGGTTGTCAGCCTGTCCC
GCTTATAAGATCATACGCCGTTATACGTTGTTTACGCTTTGAGGAATCCCATATGGATAAGAAGCCGCTGGATGTTCTGATCT
CTGCGACCGGTCTGTGGATGTCCCGTACCGGCACGCTGCACAAGATCAAGCACTATGAGATTTCTCGTTCTAAAATCTACATC
GAAATGGCGTGTGGTGACCATCTGGTTGTGAACAACTCTCGTTCTTGTCGTCCCGCACGTGCATTCCGTTATCATAAATACCG

TAAAACCTGCAAACGTTGTCGTGTTTCTGACGAAGATATCAACAACTTCCTGACCCGTTCTACCGAAGGCAAAACCTCTGTTA
AAGTTAAAGTTGTTTCTGAGCCGAAAGTGAAAAAAGCGATGCCGAAATCTGTTTCTCGTGCGCCGAAACCGCTGGAAAATCCG
GTTTCTGCGAAAGCGTCTACCGACACCTCTCGTTCTGTTCCGTCTCCGGCGAAATCTACCCCGAACTCTCCGGTTCCGACCTC
TGCGCCGGCACCGAGCCTGACCCGCAGCCAGCTGGATCGTGTGGAAGCGCTGCTGTCTCCGGAAGATAAAATTAGCCTGAACA
TGGCGAAACCGTTTCGTGAACTGGAACCGGAACTGGTGACCCGTCGTAAAAACGATTTTCAGCGCCTGTATACCAACGATCGT
GAAGATTATCTGGGCAAACTGGAACGTGATATCACCAAATTTTTTGTGGATCGCGGCTTTCTGGAAATTAAAAGCCCGATTCT
GATTCCGGCGGAATATGTGGAACGTATGGGCATTAACAACGACACCGAACTGAGCAAACAAATTTTCCGCGTGGATAAAAACC
TGTGCCTGCGTCCGATGCTGCAGCCGACCCTGTATAACTATCTGCGTAAACTGGATCGTATTCTGCCGGGTCCGATCAAAATT
TTTGAAGTGGGCCCGTGCTATCGCAAAGAAAGCGATGGCAAAGAACACCTGGAAGAATTCACCATGGTTAGCTTTTGGCAAAT
GGGCAGCGGCTGCACCCGTGAAAACCTGGAAGCGCTGATCAAAGAATTCCTGGATTATCTGGAAATCGACTTCGAAATTGTGG
GCGATAGCTGCATGGTGTATGGCGATACCCTGGATATTATGCATGGCGATCTGGAACTGAGCAGCGCGGTGGTGGGTCCGGTT
AGCCTGGATCGTGAATGGGGCATTGATAAACCGTGGATTGGCGCGGGTTTTGGCCTGGAACGTCTGCTGAAAGTGATGCATGG
CTTCAAAAACATTAAACGTGCGAGCCGTAGCGAAAGCTACTATAACGGCATTAGCACGAACCTGTAATAAAGGTGGCACTTTT
CGGGGAAATGTGCGCGGCCTATAGTGAGTCGTATTAATTTCGCGGGATCGAGATCTCGATCCTCTACGCCGGACGCATCCACC
GCTGAGCAATAACTAGCATAACCCCTTGGGGCCTCTAAACGGGTCTTGAGGGGTTTTTTGCTGAAAGGAGGAACTAAACCCCT
ATTTGTTTATTTTTCTAAATACATTCAAATATGTATCCGCTCATGAATTAATTCTTAGAAAAACTCATCGAGCATCAAATGAA
ACTGCAATTTATTCATATCAGGATTATCAATACCATATTTTTGAAAAAGCCGTTTCTGTAATGAAGGAGAAAACTCACCGAGG
CAGTTCCATAGGATGGCAAGATCCTGGTATCGGTCTGCGATTCCGACTCGTCCAACATCAATACAACCTATTAATTTCCCCTC
GTCAAAAATAAGGTTATCAAGTGAGAAATCACCATGAGTGACGACTGAATCCGGTGAGAATGGCAAAAGTTTATGCATTTCTT
TCCAGACTTGTTCAACAGGCCAGCCATTACGCTCGTCATCAAAATCACTCGCATCAACCAAACCGTTATTCATTCGTGATTGC
GCCTGAGCGAGACGAAATACGCGATCGCTGTTAAAAGGACAATTACAAACAGGAATCGAATGCAACCGGCGCAGGAACACTGC
CAGCGCATCAACAATATTTTCACCTGAATCAGGATATTCTTCTAATACCTGGAATGCTGTTTTCCCGGGGATCGCAGTGGTGA
GTAACCATGCATCATCAGGAGTACGGATAAAATGCTTGATGGTCGGAAGAGGCATAAATTCCGTCAGCCAGTTTAGTCTGACC
ATCTCATCTGTAACATCATTGGCAACGCTACCTTTGCCATGTTTCAGAAACAACTCTGGCGCATCGGGCTTCCCATACAATCG
ATAGATTGTCGCACCTGATTGCCCGACATTATCGCGAGCCCATTTATACCCATATAAATCAGCATCCATGTTGGAATTTAATC
GCGGCCTAGAGCAAGACGTTTCCCGTTGAATATGGCTCAT

DNA sequence of pSLdT7-*Mb*(IPYE)PylRS$^{\tau MH}$:

The pSLdT7-*Mb*(IPYE)PylRS$^{\tau MH}$ plasmid contains the final PylRS variant obtained from engineering of *Mb*(IPYE)PylRS^{4ThzA} towards τMH incorporation. It contains a pBR322 type origin of replication, a kanamycin resistance gene (KanR), and a mutant *Mb*(IPYE)PylRS^{4ThzA} under control of a glnS promoter. The aaRS expression cassette is flanked by two T7 promoters as well as two T7 terminators, essentially as described previously[18].

Mb(IPYE)PylRS$^{\tau MH}$; KanR; pBR322 ori; T7 promoter; T7 terminator;

CCAAAATCCCTTAACGTGAGTTTTCGTTCCACTGAGCGTCAGACCCCGTAGAAAAGATCAAAGGATCTTCTTGAGATCCTTTT
TTTCTGCGCGTAATCTGCTGCTTGCAAACAAAAAAACCACCGCTACCAGCGGTGGTTTGTTTGCCGGATCAAGAGCTACCAAC
TCTTTTTCCGAAGGTAACTGGCTTCAGCAGAGCGCAGATACCAAATACTGTCCTTCTAGTGTAGCCGTAGTTAGGCCACCACT
TCAAGAACTCTGTAGCACCGCCTACATACCTCGCTCTGCTAATCCTGTTACCAGTGGCTGCTGCCAGTGGCGATAAGTCGTGT
CTTACCGGGTTGGACTCAAGACGATAGTTACCGGATAAGGCGCAGCGGTCGGGCTGAACGGGGGGTTCGTGCACACAGCCCAG
CTTGGAGCGAACGACCTACACCGAACTGAGATACCTACAGCGTGAGCTATGAGAAAGCGCCACGCTTCCCGAAGGGAGAAAGG
CGGACAGGTATCCGGTAAGCGGCAGGGTCGGAACAGGAGAGCGCACGAGGGAGCTTCCAGGGGGAAACGCCTGGTATCTTTAT
AGTCCTGTCGGGTTTCGCCACCTCTGACTTGAGCGTCGATTTTTGTGATGCTCGTCAGGGGGGCGGAGCCTATGGAAAAACGC
CAGCAACGCGGCCTTTTTACGGTTCCTGGCCTTTTGCTGGCCTTTTGCTCACATGTTCTTTCCTGCGTTATCCCCTGATTCTG
TGGATAACCGTATTACCGCCTTTGAGTGAGCTGATACCGCTCGCCGCAGCCGAACGACCGAGCGCAGCGAGTCAGTGAGCGAG
GAAGCGGAAGAGTAGTTCCTCCTTTCAGCAAAAAACCCCTCAAGACCCGTTTAGAGGCCCCAAGGGGTTATGCTAGTTATTGC
TCAGCGGTGGATGCGTCCGGCGTAGAGGATCGAGATCTCGATCCCGCGAAATTAATACGACTCACTATAGGAACTTTTGCTGA
GTTGAAGGATCCTCGGGTTGTCAGCCTGTCCCGCTTATGAGATCATACGCCGTTATACGTTGTTTACGCTTTAAGGAATCCCA
TATGGATAAGAAGCCGCTGGATGTTCTGATCTCTGCGACCGGTCTGTGGATGTCCCGTACCGGCACGCTGCACAAGATCAAGC
ACTATGAGATTTCTCGTTCTAAAATCTACATCGAAATGGCGTGTGGTGACCATCTGGTTGTGAACAACTCTCGTTCTTGTCGT
CCCGCACGTGCATTCCGTTATCATAAATACCGTAAAACCTGCAAACGTTGTCGTGTTTCTGACGAAGATATCAACAACTTCCT
GACCCGTTCTACCGAAGGCAAAACCTCTGTTAAAGTTAAAGTTGTTTTTGAGCCGAAAGTGAAAAAAGCGATGCCGAAATCTG
TTTCTCGTGCGCCGAAACCGCTGGAAAATCCGGTTTCTGCGAAAGCGTCTACCGACACCTCTCGTTCTGTTCCGTCTCCGGCG
AAATCTACCCCGAACTCTCCGGTTCCGACCTCTGCGCCGGCACCGAGCCTGACCCGCAGCCAGCTGGATCGTGTGGAAGCGCT
GCTGTCTCCGGAAGATAAAATTAGCCTGAACATGGCGAAACCGTTTCGTGAACTGGAACCGGAACTGGTGACCCGTCGTAAAA
ACGATTTTCAGCGCCTGTATATCAACGATCGTGAAGATTATCTGGGCAAACTGGAACGTGATATCACCAAATTTTTTGTGGAT
CGCGGCTTTCTAGAAATTAAAAGCCCGATTCTGATTCCGGCGGAATATGTGGAACGTATGGGCATTAACAACGACACCGAACT
GAGCAAACAAATTTTCCGCGTGGATAAAAACCTGTGCCTGCGTCCGATGCTGCAGCCGACCCTGTATAACTATCTGCGTAAAC
TGGATCGTATTTTGCCAGGTCCGATCAAAATTTTTGAAGTGGGCCCGTGCTATCGCAAAGAAAGCGATGGCAAAGAACACCTG
GAAGAATTCACCATGGTTAGCTTTTGGCAAATGGGCAGCGGCTGCACCCGTGAAAACCTGGAAGCGCTGATCAAAGAATTCCT
GGATTATCTGGAAATCGACTTCGAAATTGTGGGCAATAGCTGCATGGTGTATGGCGATACCCTGGATATTATGCATGGCGATC
TAGAACTGAGCACCGCGGTGGTGGGTCCGGTTAGCCTGGATCGTGAATGGGGCATTGATAAACCGTGGATTGGCGCGGGTTTT
GGCCTGGAACGTCTGCTGAAAGTGATGCATGGCTTCAAAAACATTAAACGTGCGAGCCGTAGCGAAAGCTACTATAACGGCAT
TAGCACCAACCTGTAATAAAGGTGGCACTTTTCGGGGAAATGTGCGCGGCCTATAGTGAGTCGTATTAATTTCGCGGGATCGA

GATCTCGATCCTCTACGCCGGACGCATCCACCGCTGAGCAATAACTAGCATAACCCCTTGGGGCCTCTAAACGGGTCTTGAGG
GGTTTTTTGCTGAAAGGAGGAACTAAACCCCTATTTGTTTATTTTTCTAAATACATTCAAATATGTATCCGCTCATGAATTAA
TTCTTAGAAAAACTCATCGAGCATCAAATGAAACTGCAATTTATTCATATCAGGATTATCAATACCATATTTTTGAAAAAGCC
GTTTCTGTAATGAAGGAGAAAACTCACCGAGGCAGTTCCATAGGATGGCAAGATCCTGGTATCGGTCTGCGATTCCGACTCGT
CCAACATCAATACAACCTATTAATTTCCCCTCGTCAAAAATAAGGTTATCAAGTGAGAAATCACCATGAGTGACGACTGAATC
CGGTGAGAATGGCAAAAGTTTATGCATTTCTTTCCAGACTTGTTCAACAGGCCAGCCATTACGCTCGTCATCAAAATCACTCG
CATCAACCAAACCGTTATTCATTCGTGATTGCGCCTGAGCGAGACGAAATACGCGATCGCTGTTAAAAGGACAATTACAAACA
GGAATCGAATGCAACCGGCGCAGGAACACTGCCAGCGCATCAACAATATTTTCACCTGAATCAGGATATTCTTCTAATACCTG
GAATGCTGTTTTCCCGGGGATCGCAGTGGTGAGTAACCATGCATCATCAGGAGTACGGATAAAATGCTTGATGGTCGGAAGAG
GCATAAATTCCGTCAGCCAGTTTAGTCTGACCATCTCATCTGTAACATCATTGGCAACGCTACCTTTGCCATGTTTCAGAAAC
AACTCTGGCGCATCGGGCTTCCCATACAATCGATAGATTGTCGCACCTGATTGCCCGACATTATCGCGAGCCCATTTATACCC
ATATAAATCAGCATCCATGTTGGAATTTAATCGCGGCCTAGAGCAAGACGTTTCCCGTTGAATATGGCTCATAACACCCCTTG
TATTACTGTTTATGTAAGCAGACAGTTTTATTGTTCATGA

DNA sequence of pDae079so:

The pDae079so is derived from pDae079, a gift from Seokhee Kim (Addgene plasmid # 187622) and contains an oriV type origin of replication (oriV/trfA), a spectinomycin resistance gene (SpecR), and the eMutaT7[transition] mutator system[18].

SpecR; oriV/trfA;

ATGGGGAGAGCCTGAGCAAACTGGCCTCAGGCATTTGAGAAGCACAAGGGTCCCTAAGTCTCCTCAGCAAAACGAAAGGCCCA
GTCTTTCGACTGAGCCTTTCGTTTTATTTGACCGGATGTCCTCTTACTGACAGATGAGGGGCGGACGTTGACACTTGAGGGGC
CGACTCACCCGGCGCGGCGTTGACAGATGAGGGGCAGGCTCGATTTCGGCCGGCGACGTGGAGCTGGCCAGCCTCGCAAATCG
GCGAAAACGCCTGATTTTACGCGAGTTTCCCACAGATGATGTGGACAAGCCTGGGGATAAGTGCCCTGCGGTATTGACACTTG
AGGGGCGCGACTACTGACAGATGAGGGGCGCGATCCTTGACACTTGAGGGGCAGAGTGCTGACAGATGAGGGGCGCACCTATT
GACATTTGAGGGGCTGTCCACAGGCAGAAAATCCAGCATTTGCAAGGGTTTCCGCCCGTTTTTCGGCCACCGCTAACCTGTCT
TTTAACCTGCTTTTAAACCAATATTTATAAACCTTGTTTTTAACCAGGGCTGCGCCCTGTGCGCGTGACCGCGCACGCCGAAG
GGGGGTGCCCCCCCTTCTCGAACCCTCCCGGCCCGCTAACGCGGGCCTCCCATCCCCCCAGGGGCTGCGCCCCTCGGCCGCGA
ACGGCCTCACCCCAAAAATGGAGGTTTCTCTTCTTTCACTTTACGGCTAGCTCAGCCCTAGGTATTATGCTAGCAGCCCCATA
CGATATAAGGAGGGTGAATGAATCGGACGTTTGACCGGAAGGCATACAGGCAAGAACTGATCGACGCGGGGTTTTCCGCCGAG
GATGCCGAAACCATCGCAAGCCGCACCGTCATGCGTGCGCCCCGCGAAACCTTCCAGTCCGTCGGCTCGATGGTCCAGCAAGC
TACGGCCAAGATCGAGCGCGACAGCGTGCAACTGGCTCCCCCTGCCCTGCCCGCGCCATCGGCCGCCGTGGAGCGTTCGCGTC
GTCTCGAACAGGAGGCGGCAGGTTTGGCGAAGTCGATGACCATCGACACGCGAGGAACTATGACGACCAAGAAGCGAAAAACC
GCCGGCGAGGACCTGGCAAAACAGGTCAGCGAGGCCAAGCAGGCCGCGTTGCTGAAACACACGAAGCAGCAGATCAAGGAAAT
GCAGCTTTCCTTGTTCGATATTGCGCCGTGGCCGGACACGATGCGAGCGATGCCAAACGACACGGCCCGCTCTGCCCTGTTCA
CCACGCGCAACAAGAAAATCCCGCGCGAGGCGCTGCAAAACAAGGTCATTTTCCACGTCAACAAGGACGTGAAGATCACCTAC
ACCGGCGTCGAGCTGCGGGCCGACGATGACGAACTGGTGTGGCAGCAGGTGTTGGAGTACGCGAAGCGCACCCCTATCGGCGA
GCCGATCACCTTCACGTTCTACGAGCTTTGCCAGGACCTGGGCTGGTCGATCAATGGCCGGTATTACACGAAGGCCGAGGAAT
GCCTGTCGCGCCTACAGGCGACGGCGATGGGCTTCACGTCCGACCGCGTTGGGCACCTGGAATCGGTGTCGCTGCTGCACCGC
TTCCGCGTCCTGGACCGTGGCAAGAAAACGTCCCGTTGCCAGGTCCTGATCGACGAGGAAATCGTCGTGCTGTTTGCTGGCGA
CCACTACACGAAATTCATATGGGAGAAGTACCGCAAGCTGTCGCCGACGGCCCGACGGATGTTCGACTATTTCAGCTCGCACC
GGGAGCCGTACCCGCTCAAGCTGGAAACCTTCCGCCTCATGTGCGGATCGGATTCCACCCGCGTGAAGAAGTGGCGCGAGCAG
GTCGGCGAAGCCTGCGAAGAGTTGCGAGGCAGCGGCCTGGTGGAACACGCCTGGGTCAATGATGACCTGGTGCATTGCAAACG
CTAGACGGCCTATTTGGCCTATTTTTCTAAATACATTCAAATATGTATCCGCTCATGAGACAATAACCCTGATAAATGCTTCA
ATAATATTGAAAAAGGAAGAGTATGAGGGAAGCGGTGATCGCCGAAGTATCGACTCAACTATCAGAGGTAGTTGGCGTCATCG
AGCGCCATCTCGAACCGACGTTGCTGGCCGTACATTTGTACGGCTCCGCAGTGGATGGCGGCCTGAAGCCACACAGTGATATT
GATTTGCTGGTTACGGTGACCGTAAGGCTTGATGAAACAACGCGGCGAGCTTTGATCAACGACCTTTTGGAAACTTCGGCTTC
CCCTGGAGAGAGCGAGATTCTCCGCGCTGTAGAAGTCACCATTGTTGTGCACGACGACATCATTCCGTGGCGTTATCCAGCTA
AGCGCGAACTGCAATTTGGAGAATGGCAGCGCAATGACATTCTTGCAGGTATCTTCGAGCCAGCCACGATCGACATTGATCTG
GCTATCTTGCTGACAAAAGCAAGAGAACATAGCGTTGCCTTGGTAGGTCCAGCGGCGGAGGAACTCTTTGATCCGGTTCCTGA
ACAGGATCTATTTGAGGCGCTAAATGAAACCTTAACGCTATGGAACTCGCCGCCCGACTGGGCTGGCGATGAGCGAAATGTAG
TGCTTACGTTGTCCCGCATTTGGTACAGCGCAGTAACCGGCAAAATCGCGCCGAAGGATGTCGCTGCCGACTGGGCAATGGAG
CGCCTGCCGGCCCAGTATCAGCCCGTCATACTTGAAGCTAGACAGGCTTATCTTGGACAAGAAGAAGATCGCTTGGCCTCGCG
CGCAGATCAGTTGGAAGAATTTGTCCACTACGTGAAAGGCGAGATCACCAAGGTAGTCGGCAAATAAACGCCATGGGCATTAA
AAAATGCCCTCTTGGGTTATCAAGAGGGTCATTATATTTGCTGAGGAGTGCCGTTATTAAACTGATGCAGCGTAAAATCAATC
TAAAGTATATATGAGTAAACTTGGTCTGACAGTTACCAATGCTTAATCAGTGAGGCACCTATCTCAGCGATCTGTCTATTTCG
TTCATCCATAGTTGCCTGACTCCCCGTCGTGTAGATAACTACGATACGGGAGGGCTTACCATCTGGCCCCAGTGCTGCAATGA
TACCGCGAGACCCACGCTCACCGGCTCCAGATTTATCAGCAATAAACCAGCCAGCCGGAAGGGCCGAGCGCAGAAGTGGTCCT
GCAACTTTATCCGCCTCCATCCAGTCTATTAATTGTTGCCGGGAAGCTAGAGTAAGTAGTTCGCCAGTTAATAGTTTGCTGCA
CCCAACTGATCTTCAGCATCTTTTACTTTCACCAGCGTTTCTGGGTGAGCAAAAACAGGAAGGCAAAATGCCGCAAAAAAGGG
AATAAGGGCGACACGGAAATGTTGAATACTCATACTCTTCCTTTTTCAATATTATTGAAGCATTTATCAGGGTTATTGTCTCA
TGAGCGGATACATATTTGAATGTATTTAGAAAAATAAACAAAAGAGTTTGTAGAAACGCAAAAAGGCCATCCGTCAGGATGGC
CTTCTGCTTAATTTGATGCCTGGCAGTTTATGGCGGGCGTCCTGCCCGCCACCCTCCGGGCCGTTGCTTCGCAACGTTCAAAT
CCGCTCCCGGCGGATTTGTCCTACTCAGGAGAGCGTTCACCGACAAACAACAGATAAAACGAAAGGCCCAGTCTTTCGACTGA
GCCTTTCGTTTTATTTGATGCCTGGCAGTTCCCTACTCTCGCATGGGGAGACCCCACACTACCATCGGCGCTACGGCGTTTCA

CTTCTGAGTTCGGCATGGGGTCAGGTGGGACCACCGCGCTACTGCCGCCAGGCAAATTCTGTTTTATCAGACCGCTTCTGCGT
TCTGATTTAATCTGTATCAGGCTGAAAATCTTCTCTCATCCGCCAAAACAGCCAAGCTTTTACAGCATCTTGATCTTGTTCTC
TCCATTAGAGTCCTGAATCACCAGAGCCCAGGGTTTGTACTCTGGGGCATCTGACGTGAGCAGCATCACGTTCTCATCGGTGC
TCTCATCATACGCAGTGTGTACGAGAATGTCACTTTCCGGCTTATTCCCAATGACCTCCTCAACTTCTTCAGGAAGCATGAGT
ATCGACTCTTGGATGACCAACTGTTTGCCTGTTTCCTTCTCTATGATGTCGGAAAGGTTGGTCATAAAAAATCCTCCTTATTT
ATTGCTAGTACTCAAACAGAGCGCGCTCTGTTAGGATCCCCGGGCTGCAGTTACGCGAACGCGAAGTCCGACTCTAAGATGTC
ACGGAGGTTCAAGTTACCTTTAGCCGGAAGTGCTGGCATTTTGTCCAATTGAGACTCGTGCAACTGGTCAGCGAACTGGTCGT
AGAAATCAGCCAGTACATCACAAGACTCATAGGTGTCAACCATAGTTTCGCGCACTGCTTTGAACAGGTTCGCAGCGTCAGCC
GGAATGGTACCGAAGGAGTCGTGAATCAGTGCAAAAGATTCGATTCCGTACTTCTCGTGTGCCCACACTACAGTCTTACGAAG
GTGGCTACCGTCTTGGCTGTGTACAAAGTTAGGAGCGATACCAGACTCCTGTTTGTGTGCATCAATCTCGCTATCTTTGTTGG
TGTTAATGGTAGGCTGTAAGCGGAACTGACCGAGGAACATCAGGTTCAAGCGCGTCTGAATAGGCTTCTTGTATTCCTGCCAC
ACAGGGAAACCATCAGGAGTTACCCAATGCACAGCGCAACGCTTGCGAAGAATCTCTCCAGTCTTCTTATCTTTGACCTCAGC
AGCCAGCAGCTTAGCAGCAGACTTAAGCCAGTTCATTGCTTCAACCGCAGCTACCACCGTCACGCTCACAGATTCCCAAATCA
GCTTAGCCATGTATCCAGCAGCCTGATTCGGCTGAGTGAACATCAGACCCTTGCCGGAATCAATAGCTGGCTGAATGGTATCT
TCCAGCACTTGTTGACGGAAGCCGAACTCTTTGGACCCGTAAGCCAGCGTCATGACTGAACGCTTAGTCACACTGCGAGTAAC
ACCGTAAGCCAGCCATTGACCAGCCAGTGCCTTAGTGCCCAGCTTGACTTTCTCAGAGATTTCACCAGTGTTCTCATCGGTCA
CGGTAACTACTTCGTTATCGGTCCCATTGATTGCGTCTGCTTGTAGAATCTCGTTGACTTTCTTAGCAACAATCCCGTAGATG
TCCTGAACGGTTTCACTAGGAAGCAAGTTAACCGCGCGACCACCTACCTCATCTCGGAGCATCGCGGAGAAGTGCTGGATGCC
AGAGCAAGACCCGTCAAACGCCAGCGGAAGGGAGCAGTTATAGCTCAGGCCGTGGTGCTGTACCCCAGCGTACTCAAAGCAGA
ACGCAAGGAAGCAGAACGGAGAATCTTGCTCAGCCCACCAAGTGTTCTCCAGTGGAGACTTAGCGCAAGCCATGATGTTCTCG
TGGTTTTCCTCAATGAACTTGATGCGCTCAGGGAACGGAACCTTATCGACACCCGCACAGTTTGCACCGTGGATTTTCAGCCA
GTAGTAACCTTCCTTACCGATTGGTTTACCTTTCGCCAGCGTAAGCAGTCCTTTGGTCATATCGTTACCTTGCGGGTTGAACA
TTGACACAGCGTAAACACGACCGCGCCAGTCCATGTTGTAAGGGAACCAGATGGCCTTATGGTTAGCAAACTTATTGGCTTGC
TCAAGCATGAACTCAAGGCTGATACGGCGAGACTTGCGAGCCTTGTCCTTGCGGTACACAGCAGCGGCAGCACGTTTCCACGC
GGTGAGAGCCTCAGGATTCATGTCGATGTCTTCCGGTTTCATCGGGAGTTCTTCACGCTCAATCGCAGGGATGTCCTCGACCG
GACAATGCTTCCACTTGGTGATTACGTTGGCGACCGCTAGGACTTTCTTGTTGATTTTCCATGCGGTGTTTTGCGCAATGTTA
ATCGCTTTGTACACCTCAGGCATGTAAACGTCTTCGTAGCGCATCAGTGCTTTCTTACTGTGAGTACGCACCAGCGCCAGAGG
ACGACGACCGTTAGCCCAATAGCCACCACCAGTAATGCCAGTCCACGGCTTAGGAGGAACTACGCAAGGTTGGAACATCGGAG
AGATGCCAGCCAGCGCACCTGCACGGGTTGCGATAGCCTCAGCGTATTCAGGTGCGAGTTCGATAGTCTCAGAGTCTTGACCT
ACTACGCCAGCATTTTGGCGGTGTAAGCTAACCATTCCGGTTGACTCAATGAGCATCTCGATGCAGCGTACTCCTACATGAAT
AGAGTCTTCCTTATGCCACGAAGACCACGCCTCGCCACCGAGTAGACCCTTAGAGAGCATGTCAGCCTCGACAACTTGCATAA
ATGCTTTCTTGTAGACGTGCCCTACGCGCTTGTTGAGTTGTTCCTCAACGTTTTTCTTGAAGTGCTTAGCTTCAAGGTCACGG
ATACGACCGAAGCGAGCCTCGTCCTCAATGGCCCGACCGATTGCGCTTGCTACAGCCTGAACGGTTGTATTGTCAGCACTGGT
TAGGCAAGCCAGAGTGGTCTTAATGGTGATGTACGCTACGGCTTCCGGCTTGATTTCTTGCAGGAACTGGAAGGCTGTCGGGC
GCTTGCCGCGCTTAGCTTTCACTTCCTCAAACCAGTCGTTGATGCGTGCAATCATCTTAGGGAGTAGGGTAGTGATGAGAGGC
TTGGCGGCAGCGTTATCCGCAACCTCACCAGCTTTAAGTTGACGCTCAAACATCTTGCGGAAGCGTGCTTCACCCATCTCGTA
AGACTCATGCTCAAGGGCCAACTGTTCGCGAGCTAAACGCTCACCGTAATGGTCAGCCAGAGTGTTGAACGGGATAGCAGCCA
GTTCGATGTCAGAGAAGTCGTTCTTAGCGATGTTAATCGTGTTACTTTCGGGTGTGGCGGACTCTGAGGTCCCGGGAGTCTCG
CTAACGGCTGGAGACTTAGTGGTATGCAGAATCTTGACCTGGATCATGATGCTCAGCTCGGATCTCCACTTCTCCGCACGTTT
CAGTGTTTTCTCCAACCAGCGGTTTTCATTGAGCTGGTTATGAGAGCTTTGGATGAAAATCTTCCGACAACACTGATAGTGCT
CGGAAACCATCACGTTCAGGCCTACACCATTGTCCCTCAGATTCCAGAGTCCTATCTGGTTCCGGGCATTCTTCTCGTAGTAC
AGCTTGCAGGCCCAGATTTTCAATGTGTGCCCGTTTCCCCGAAGTTCCTGATTATACCATTCCAGAATTTTCTCGGCACAATC
AGCGCAAGGACTCCAGCTGGAATACCAGTTGATCGTAAACTGCCCAGGATTATCCCGAAGATACTCTTCCACCTTTCGGATAC
TGAATATCTCAGCGTGTATTCCTCTCTCGGTGCCACTTTGGGGCTTGTTGACGGCATAGCCCCAAAAGCAAGCCCTTCTTTCG
CCCCTTCGCTTCAACTCAAACAGCACGTAGCAGCGGTGTGACACAGATTTCTTGTTGTTGAAGAACTGCTTCTTGAAGGTGTA
AATATCCAGTTTCTCATGAATGCGCACGTACTCGGCGTCTGTCATCATATGATAATGCCATGGTGAATTCCTCCTGAGCTCGA
ATTCGCTAGCCCAAAAAAACGGGTATGGAGAAACAGTAGAGAGTTGCGATAAAAAGCGTCAGGTAGGATCCGCTAATCTTATG
GATAAAAATGCTATGGCATAGCAAAGTGTGACGCCGTGCAAATAATCAATGTGGACTTTTCTGCCGTGATTATAGACACTTTT
GTTACGCGTTTTTGTCATGGCTTTGGTCCCGCTTTGTTACAGAATGCTTTTAATAAGCGGGGTTACCGGTTTGGTTAGCGAGA
AGAGCCAGTAAAAGACGCAGTGACGGCAATGTCTGATGCAATATGGACAATTGGTTTCTTCTCTGAATGGCGGGAGTATGAAA
AGTCTGCCGCCAGGCAAATTCTGTTTTATCAGACCGCTTCTGCGTTCTGATTTAATCTGTATCAGGCTGAAAATCTTCTCTCA
TCCGCCAAAACAGCCAAGCTTATACTGCAGTTACGCGAACGCGAAGTCCGACTCTAAGATGTCACGGAGGTTCAAGTTACCTT
TAGCCGGAAGTGCTGGCATTTTGTCCAATTGAGACTCGTGCAACTGGTCAGCGAACTGGTCGTAGAAATCAGCCAGTACATCA
CAAGACTCATAGGTGTCAACCATAGTTTCGCGCACTGCTTTGAACAGGTTCGCAGCGTCAGCCGGAATGGTACCGAAGGAGTC
GTGAATCAGTGCAAAAGATTCGATTCCGTACTTCTCGTGTGCCCACACTACAGTCTTACGAAGGTGGCTACCGTCTTGGCTGT
GTACAAAGTTAGGAGCGATACCAGACTCCTGTTTGTGTGCATCAATCTCGCTATCTTTGTTGGTGTTAATGGTAGGCTGTAAG
CGGAACTGACCGAGGAACATCAGGTTCAAGCGCGTCTGAATAGGCTTCTTGTATTCCTGCCACACAGGGAAACCATCAGGAGT
TACCCAATGCACAGCGCAACGCTTGCGAAGAATCTCTCCAGTCTTCTTATCTTTGACCTCAGCAGCCAGCAGCTTAGCAGCAG
ACTTAAGCCAGTTCATTGCTTCAACCGCAGCTACCACCGTCACGCTCACAGATTCCCAAATCAGCTTAGCCATGTATCCAGCA
GCCTGATTCGGCTGAGTGAACATCAGACCCTTGCCGGAATCAATAGCTGGCTGAATGGTATCTTCCAGCACTTGTTGACGGAA
GCCGAACTCTTTGGACCCGTAAGCCAGCGTCATGACTGAACGCTTAGTCACACTGCGAGTAACACCGTAAGCCAGCCATTGAC
CAGCCAGTGCCTTAGTGCCCAGCTTGACTTTCTCAGAGATTTCACCAGTGTTCTCATCGGTCACGGTAACTACTTCGTTATCG
GTCCCATTGATTGCGTCTGCTTGTAGAATCTCGTTGACTTTCTTAGCAACAATCCCGTAGATGTCCTGAACGGTTTCACTAGG
AAGCAAGTTAACCGCGCGACCACCTACCTCATCTCGGAGCATCGCGGAGAAGTGCTGGATGCCAGAGCAAGACCCGTCAAACG
CCAGCGGAAGGGAGCAGTTATAGCTCAGGCCGTGGTGCTGTACCCCAGCGTACTCAAAGCAGAACGCAAGGAAGCAGAACGGA
GAATCTTGCTCAGCCCACCAAGTGTTCTCCAGTGGAGACTTAGCGCAAGCCATGATGTTCTCGTGGTTTTCCTCAATGAACTT
GATGCGCTCAGGGAACGGAACCTTATCGACACCCGCACAGTTTGCACCGTGGATTTTCAGCCAGTAGTAACCTTCCTTACCGA
TTGGTTTACCTTTCGCCAGCGTAAGCAGTCCTTTGGTCATATCGTTACCTTGCGGGTTGAACATTGACACAGCGTAAACACGA

CCGCGCCAGTCCATGTTGTAAGGGAACCAGATGGCCTTATGGTTAGCAAACTTATTGGCTTGCTCAAGCATGAACTCAAGGCT
GATACGGCGAGACTTGCGAGCCTTGTCCTTGCGGTACACAGCAGCGGCAGCACGTTTCCACGCGGTGAGAGCCTCAGGATTCA
TGTCGATGTCTTCCGGTTTCATCGGGAGTTCTTCACGCTCAATCGCAGGGATGTCCTCGACCGGACAATGCTTCCACTTGGTG
ATTACGTTGGCGACCGCTAGGACTTTCTTGTTGATTTTCCATGCGGTGTTTTGCGCAATGTTAATCGCTTTGTACACCTCAGG
CATGTAAACGTCTTCGTAGCGCATCAGTGCTTTCTTACTGTGAGTACGCACCAGCGCCAGAGGACGACGACCGTTAGCCCAAT
AGCCACCACCAGTAATGCCAGTCCACGGCTTAGGAGGAACTACGCAAGGTTGGAACATCGGAGAGATGCCAGCCAGCGCACCT
GCACGGGTTGCGATAGCCTCAGCGTATTCAGGTGCGAGTTCGATAGTCTCAGAGTCTTGACCTACTACGCCAGCATTTTGGCG
GTGTAAGCTAACCATTCCGGTTGACTCAATGAGCATCTCGATGCAGCGTACTCCTACATGAATAGAGTCTTCCTTATGCCACG
AAGACCACGCCTCGCCACCGAGTAGACCCTTAGAGAGCATGTCAGCCTCGACAACTTGCATAAATGCTTTCTTGTAGACGTGC
CCTACGCGCTTGTTGAGTTGTTCCTCAACGTTTTTCTTGAAGTGCTTAGCTTCAAGGTCACGGATACGACCGAAGCGAGCCTC
GTCCTCAATGGCCCGACCGATTGCGCTTGCTACAGCCTGAACGGTTGTATTGTCAGCACTGGTTAGGCAAGCCAGAGTGGTCT
TAATGGTGATGTACGCTACGGCTTCCGGCTTGATTTCTTGCAGGAACTGGAAGGCTGTCGGGCGCTTGCCGCGCTTAGCTTTC
ACTTCCTCAAACCAGTCGTTGATGCGTGCAATCATCTTAGGGAGTAGGGTAGTGATGAGAGGCTTGGCGGCAGCGTTATCCGC
AACCTCACCAGCTTTAAGTTGACGCTCAAACATCTTGCGGAAGCGTGCTTCACCCATCTCGTAAGACTCATGCTCAAGGGCCA
ACTGTTCGCGAGCTAAACGCTCACCGTAATGGTCAGCCAGAGTGTTGAACGGGATAGCAGCCAGTTCGATGTCAGAGAAGTCG
TTCTTAGCGATGTTAATCGTGTTACTTTCGGGTGTGGCGGACTCTGAGGTCCCGGGAGTCTCGCTGCCGCTATTAATGCTGCT
CTGTGCTTTCTTTTGTGCATTAAACACTTGACGCGGCATGCGATAAAAATCGCACAGCAGTGCGGCACATTCATCGGCCAGAA
TACCCTCGGTAATTTCCACGCGATGATTCATGCCAGGATAATTCAGAACATTCATCAGGCTGCCGGCGGCACCGCGCTTGCTA
TTACGAACGCCAAACACAACACGGCCAATACGACTGTGAATCATTGCACCCGCACACATCACACAAGGTTCAAAGGTCACATA
CAGGGTGGCATCAATCAGGCGATAATTTTGCATAACCAGACCACCCTGACGCAGGGCCATGATTTCTGCATGTGCGGTAGGAT
CATGCAGACCAATCGCACGATTCCAGCCTTCGCCAATCACACGATTATTTAAAACCAGCACCGCACCAACCGGCACTTCACGT
TCATCGCGGGCACGTTTTGCCAGGGTCAGGGCATGACGCATCCAATATTCATGGCTGAATTCAACTTCACTCATATGCCATGG
TGAATTCCTCCTGAGCTCGAATTCGCTAGCCCAAAAAAACGGGTATGGAGAAACAGTAGAGAGTTGCGATAAAAAGCGTCAGG
TAGGATCCGCTAATCTTATGGATAAAAATGCTATGGCATAGCAAAGTGTGACGCCGTGCAAATAATCAATGTGGACTTTTCTG
CCGTGATTATAGACACTTTTGTTACGCGTTTTTGTCATGGCTTTGGTCCCGCTTTGTTACAGAATGCTTTTAATAAGCGGGGT
TACCGGTTTGGTTAGCGAGAAGAGCCAGTAAAAGACGCAGTGACGGCAATGTCTGATGCAATATGGACAATTGGTTTCTTCTC
TGAATGGCGGGAGTATGAAAAGTATGGCTGAAGCGCAAAATGATCCCCTGCTGCCGGGATACTCGTTTAATGCCCATCTGGTG
GCGGGTTTAACGCCGATTGAGGCCAACGGTTATCTCGATTTTTTTATCGACCGACCGCTGGGAATGAAAGGTTATATTCTCAA
TCTCACCATTCGCGGTCAGGGGGTGGTGAAAAATCAGGGACGAGAATTTGTTTGCCGACCGGGTGATATTTTGCTGTTCCCGC
CAGGAGAGATTCATCACTACGGTCGTCATCCGGAGGCTCGCGAATGGTATCACCAGTGGGTTTACTTTCGTCCGCGCGCCTAC
TGGCATGAATGGCTTAACTGGCCGTCAATATTTGCCAATACGGGGTTCTTTCGCCCGGATGAAGCGCACCAGCCGCATTTCAG
CGACCTGTTTGGGCAAATCATTAACGCCGGGCAAGGGGAAGGGCGCTATTCGGAGCTGCTGGCGATAAATCTGCTTGAGCAAT
TGTTACTGCGGCGCATGGAAGCGATTAACGAGTCGCTCCATCCACCGATGGATAATCGGGTACGCGAGGCTTGTCAGTACATC
AGCGATCACCTGGCAGACAGCAATTTTGATATCGCCAGCGTCGCACAGCATGTTTGCTTGTCGCCGTCGCGTCTGTCACATCT
TTTCCGCCAGCAGTTAGGGATTAGCGTCTTAAGCTGGCGCGAGGACCAACGTATCAGCCAGGCGAAGCTGCTTTTGAGCACCA
CCCGGATGCCTATCGCCACCGTCGGTCGCAATGTTGGTTTTGACGATCAACTCTATTTCTCGCGGGTATTTAAAAAATGCACC
GGGGCCAGCCCGAGCGAGTTCCGTGCCGGTTGTGAAGAAAAAGTGAATGATGTAGCCGTCAAGTTGTCATAATTGGTAACGAA
TCAGACAATTGACGGCTTGACGGAGTAGCATAGGGTTTGCAGAATCCCTGCTTCGTCCATTTGTTCACCTTTGCTAACC

VI. References

(1) Vatansever, E. C.; Yang, K. S.; Geng, Z. Z.; Qiao, Y.; Li, P.; Xu, S.; Liu, W. R. A Designed, Highly Efficient Pyrrolysyl-tRNA Synthetase Mutant Binds o-Chlorophenylalanine Using Two Halogen Bonds. *J Mol Biol* **2022**, *434* (8), 167534. https://doi.org/10.1016/j.jmb.2022.167534.

(2) Tharp, J. M.; Wang, Y.-S.; Lee, Y.-J.; Yang, Y.; Liu, W. R. Genetic Incorporation of Seven Ortho-Substituted Phenylalanine Derivatives. *ACS Chem Biol* **2014**, *9* (4), 884–890. https://doi.org/10.1021/cb400917a.

(3) Passaro, S.; Corso, G.; Wohlwend, J.; Reveiz, M.; Thaler, S.; Somnath, V. R.; Getz, N.; Portnoi, T.; Roy, J.; Stark, H.; Kwabi-Addo, D.; Beaini, D.; Jaakkola, T.; Barzilay, R. Boltz-2: Towards Accurate and Efficient Binding Affinity Prediction. bioRxiv June 18, 2025, p 2025.06.14.659707. https://doi.org/10.1101/2025.06.14.659707. (accessed 2025-09-16)

(4) McNutt, A. T.; Li, Y.; Meli, R.; Aggarwal, R.; Koes, D. R. GNINA 1.3: The next Increment in Molecular Docking with Deep Learning. *Journal of Cheminformatics* **2025**, *17* (1), 28. https://doi.org/10.1186/s13321-025-00973-x.

(5) Gottfried-Lee, I.; Perona, J. J.; Karplus, P. A.; Mehl, R. A.; Cooley, R. B. Structures of Methanomethylophilus Alvus Pyrrolysine tRNA-Synthetases Support the Need for De Novo Selections When Altering the Substrate Specificity. *ACS Chem. Biol.* **2022**, *17* (12), 3470–3477. https://doi.org/10.1021/acschembio.2c00640.

(6) Beattie, A. T.; Dunkelmann, D. L.; Chin, J. W. Quintuply Orthogonal Pyrrolysyl-tRNA Synthetase/tRNAPyl Pairs. *Nat. Chem.* **2023**, *15* (7), 948–959. https://doi.org/10.1038/s41557-023-01232-y.
(7) Jones, R. G.; Ainsworth, C. 1,2,4-Triazole-3-Alanine. *J. Am. Chem. Soc.* **1955**, *77* (6), 1538–1540. https://doi.org/10.1021/ja01611a040.
(8) Brinkevich, S. D.; Maliborskii, A. Ya.; Melnichuk, M. E.; Sverdlov, R. L.; Grigor’ev, Yu. V.; Shadyro, O. I. Effects of Imidazole and Its Derivatives on Radiation-Induced Dephosphorylation of Glycero-1-Phosphate in Deaerated Aqueous Solutions. *High Energy Chem* **2021**, *55* (2), 155–164. https://doi.org/10.1134/S0018143921020053.
(9) Xue, X.; Zhang, Y.; Wang, C.; Zhang, M.; Xiang, Q.; Wang, J.; Wang, A.; Li, C.; Zhang, C.; Zou, L.; Wang, R.; Wu, S.; Lu, Y.; Chen, H.; Ding, K.; Li, G.; Xu, Y. Benzoxazinone-Containing 3,5-Dimethylisoxazole Derivatives as BET Bromodomain Inhibitors for Treatment of Castration-Resistant Prostate Cancer. *European Journal of Medicinal Chemistry* **2018**, *152*, 542–559. https://doi.org/10.1016/j.ejmech.2018.04.034.
(10) Kou, Q.; Wang, T.; Zou, F.; Zhang, S.; Chen, Q.; Yang, Y. Design, Synthesis and Biological Evaluation of C(4) Substituted Monobactams as Antibacterial Agents against Multidrug-Resistant Gram-Negative Bacteria. *European Journal of Medicinal Chemistry* **2018**, *151*, 98–109. https://doi.org/10.1016/j.ejmech.2018.03.058.
(11) De Luca, L.; Giacomelli, G. An Easy Microwave-Assisted Synthesis of Sulfonamides Directly from Sulfonic Acids. *J. Org. Chem.* **2008**, *73* (10), 3967–3969. https://doi.org/10.1021/jo800424g.
(12) Van der Louw, J.; Teerhuis, N. M.; Lommerse, J. P. M.; Stock, H. T.; Hermkens, P. H. H. Novel Androgens. WO2005102998A1, November 3, 2005.
(13) Lee, Y. J.; Kim, Y.; Kim, H.; Choi, J.; Noh, G. H.; Lee, K.-S.; Lee, J.; Choi, C. H.; Kim, S. H.; Seo, J. Unlocking Cu(I)-Mediated Catalytic Pathways for Efficient ROS Generation by Incorporating an Oxazole-Based Histidine Surrogate into Cu(II)–ATCUN Complexes. *Inorg. Chem.* **2023**, *62* (26), 10279–10290. https://doi.org/10.1021/acs.inorgchem.3c01084.
(14) Kenworthy, M. N.; Kilburn, J. P.; Taylor, R. J. K. Highly Functionalized Organolithium Reagents for Enantiomerically Pure α-Amino Acid Synthesis. *Org. Lett.* **2004**, *6* (1), 19–22. https://doi.org/10.1021/ol0360039.
(15) Zhao, J.; Posa, D. K.; Kumar, V.; Hoetker, D.; Kumar, A.; Ganesan, S.; Riggs, D. W.; Bhatnagar, A.; Wempe, M. F.; Baba, S. P. Carnosine Protects Cardiac Myocytes against Lipid Peroxidation Products. *Amino Acids* **2019**, *51* (1), 123–138. https://doi.org/10.1007/s00726-018-2676-6.
(16) Miyake-Stoner, S. J.; Refakis, C. A.; Hammill, J. T.; Lusic, H.; Hazen, J. L.; Deiters, A.; Mehl, R. A. Generating Permissive Site-Specific Unnatural Aminoacyl-tRNA Synthetases. *Biochemistry* **2010**, *49* (8), 1667–1677. https://doi.org/10.1021/bi901947r.
(17) Bryson, D. I.; Fan, C.; Guo, L.-T.; Miller, C.; Söll, D.; Liu, D. R. Continuous Directed Evolution of Aminoacyl-tRNA Synthetases. *Nat Chem Biol* **2017**, *13* (12), 1253–1260. https://doi.org/10.1038/nchembio.2474.
(18) Park, H.; Kim, S. Gene-Specific Mutagenesis Enables Rapid Continuous Evolution of Enzymes in Vivo. *Nucleic Acids Res* **2021**, *49* (6), e32. https://doi.org/10.1093/nar/gkaa1231.
